# A genetic screen in combination with biochemical analysis in *Saccharomyces cerevisiae* indicates that phenazine-1-carboxylic acid is harmful to vesicular trafficking and autophagy

**DOI:** 10.1038/s41598-017-01452-6

**Published:** 2017-05-16

**Authors:** Xiaolong Zhu, Yan Zeng, Xiu Zhao, Shenshen Zou, Ya-Wen He, Yongheng Liang

**Affiliations:** 1 College of Life Sciences, Key Laboratory of Agricultural Environmental Microbiology of Ministry of Agriculture, Nanjing Agricultural University, Nanjing, 210095 China; 2 State Key Laboratory of Microbial Metabolism, School of Life Sciences and Biotechnology, Shanghai Jiao Tong University, Shanghai, 200240 China

## Abstract

The environmentally friendly antibiotic phenazine-1-carboxylic acid (PCA) protects plants, mammals and humans effectively against various fungal pathogens. However, the mechanism by which PCA inhibits or kills fungal pathogens is not fully understood. We analyzed the effects of PCA on the growth of two fungal model organisms, *Saccharomyces cerevisiae* and *Candida albicans*, and found that PCA inhibited yeast growth in a dose-dependent manner which was inversely dependent on pH. In contrast, the commonly used antibiotic hygromycin B acted in a dose-dependent manner as pH increased. We then screened a yeast mutant library to identify genes whose mutation or deletion conferred resistance or sensitivity to PCA. We isolated 193 PCA-resistant or PCA-sensitive mutants in clusters, including vesicle-trafficking- and autophagy-defective mutants. Further analysis showed that unlike hygromycin B, PCA significantly altered intracellular vesicular trafficking under growth conditions and blocked autophagy under starvation conditions. These results suggest that PCA inhibits or kills pathogenic fungi in a complex way, in part by disrupting vesicular trafficking and autophagy.

## Introduction

Phenazine is a nitrogen-containing heterocyclic molecule with broad-spectrum antimicrobial activity. Phenazine-1-carboxylic acid (PCA), an intermediate in the synthesis of different phenazines, is a novel antibiotic with strong antifungal activity which is used in both agriculture^1^ and medicine^2^. Of the bacterial genera that produce PCA, *Pseudomonas* and *Streptomyces* have been the most frequently studied^1, 3–5^. PCA-producing *Pseudomonas* and its PCA product exert a curative effect on many crop fungal diseases, including wheat take-all disease^3^, potato common scab^6, 7^, ginger rhizome rot disease^8^, pepper *Phytophthora* disease and cucumber anthracnose^9^. PCA and PCA-producing strains are used as biological control agents because their fungicidal efficiency is high, their toxicity to people and livestock is low, and they are biodegradable^10^.

The molecular mechanisms underlying the action of PCA in inhibiting or killing pathogenic fungi have been studied in a fragmentary way as the foci of individual research laboratories. The effects of PCA on growth and biofilm formation are thought to be responsible for its antifungal function against some species of pathogens^11^. Biocontrol by PCA has also been attributed to the accumulation of high levels of intracellular toxic reactive oxygen species (ROS) in the target cells^12^. However, the molecular mechanisms underlying the action of PCA in inhibiting or killing pathogenic fungi are still not fully understood, especially at the genomic level.

*Saccharomyces cerevisiae*, a unicellular laboratory model organism with evolutionarily conserved properties, has been widely used to investigate the molecular mechanisms of drug action in higher eukaryotic cells by screening drug effects on genome-wide yeast mutant libraries. Investigating the inhibitory effects of PCA on this organism and its mutants, and their response to PCA treatment could provide insight into the mechanism of PCA action, which may be generalized to pathogenic fungi that are highly homologous to yeast.

In this study, we examined the inhibitory effect of PCA on *S*. *cerevisiae* and a common opportunistic pathogenic yeast, *Candida albicans*. We further examined the resistance or sensitivity to PCA of a *S*. *cerevisiae* mutant library. Our results indicate that PCA broadly affects the metabolism of *S*. *cerevisiae* by disrupting vesicular trafficking and altering autophagy. These changes in response to PCA treatment contribute, at least in part, to the growth inhibition or death of the fungus.

## Results

### PCA inhibits the growth of wild-type *S*. *cerevisiae* and *C*. *albicans* in a dose- and pH-dependent manner

**Figure 1:**
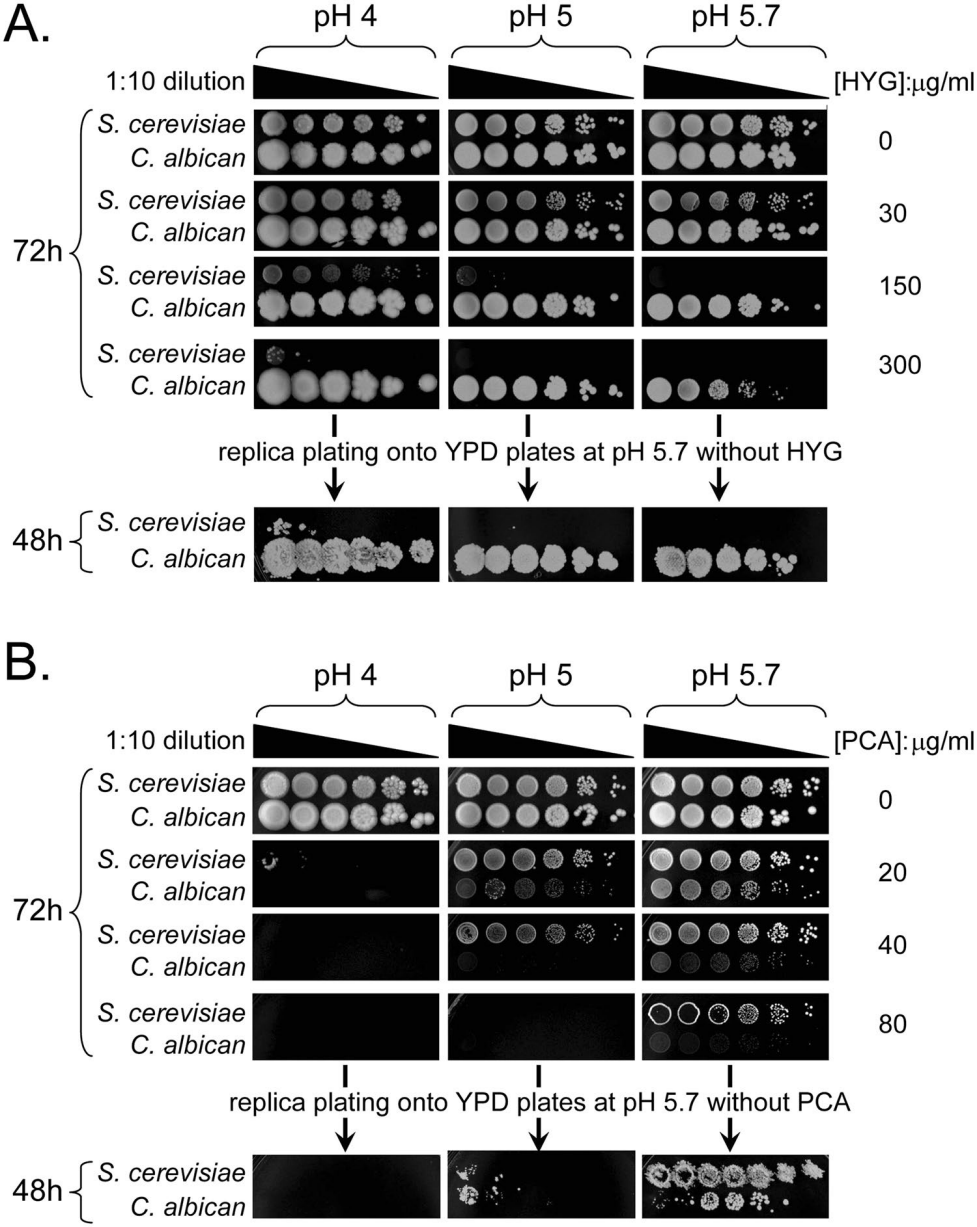
Hygromycin B and PCA inhibited the growth of *S*. *cerevisiae* and *C*. *albicans* in a dose-dependent but inversely pH-dependent manner. (**A**) Growth of *S*. *cerevisiae* and *C*. *albicans* cells on YPD plates containing different concentrations of hygromycin B (HYG) and at different pHs. Overnight-cultured cells were adjusted to OD_600_ = 1 in the first row, then were serially diluted at 1:10 and spotted onto solid YPD with the indicated concentrations of hygromycin B at pH 4, 5 or 5.7, using a 48-pin manipulator. Plates were incubated at 26 °C and photographed at 72 h. Cells on plates with 300 μg/ml HYG at 72 h were replica-plated onto YPD plates at pH 5.7 grown at 26 °C for 48 h and photographed. (**B**) Cells were grown and treated as in panel A, except with different concentrations of phenazine-1-carboxylic acid (PCA). The cells on plates containing 80 μg/ml PCA were replica-plated after 72 h.

To evaluate the inhibitory effect of PCA on yeast growth, we used hygromycin B as the reference antibiotic because yeast cells are generally sensitive to this drug. Wild-type *S*. *cerevisiae* and *C*. *albicans* cells were spotted onto yeast-peptone-dextrose (YPD) plates containing different concentrations of hygromycin B (Fig. 1A) or PCA (Fig. 1B) at different pHs. Wild-type *S*. *cerevisiae* (BY4741) was dose- and pH-dependently sensitive to hygromycin B. Stronger growth inhibition was observed at higher concentrations of hygromycin B and at the higher pH. After 72 h on YPD plates containing 300 μg/ml hygromycin B at pH 5.7, most *S*. *cerevisiae* cells were dead because they ceased to grow after they were replica-plated on YPD plates without hygromycin B at pH 5.7 for 48 h (Fig. 1A). Under the same conditions, *C*. *albicans* cells were more resistant than *S*. *cerevisiae* cells to hygromycin B treatment (Fig. 1A). In contrast, the inhibitory effect of PCA on both yeasts was dose-dependent but was inversely related to pH, and increased as pH values decreased (Fig. 1B). Both yeasts died on YPD medium containing 80 μg/ml PCA at pH 4 for 72 h because they ceased to grow when replica-plated on YPD plates without PCA at pH 5.7 for 48 h. Unexpectedly, *S*. *cerevisiae* was more resistant to PCA than was *C*. *albicans* under the same conditions. These results indicate that both hygromycin B and PCA inhibit the growth of *S*. *cerevisiae* and *C*. *albicans* in a dose- and pH-dependent manner, but the inhibitory effect of hygromycin B on yeast growth is more effective under alkaline conditions, whereas the inhibitory effect of PCA on yeast growth is more effective under acidic conditions.

### Transcriptional response of wild-type *S*. *cerevisiae* to PCA

**Figure 2:**
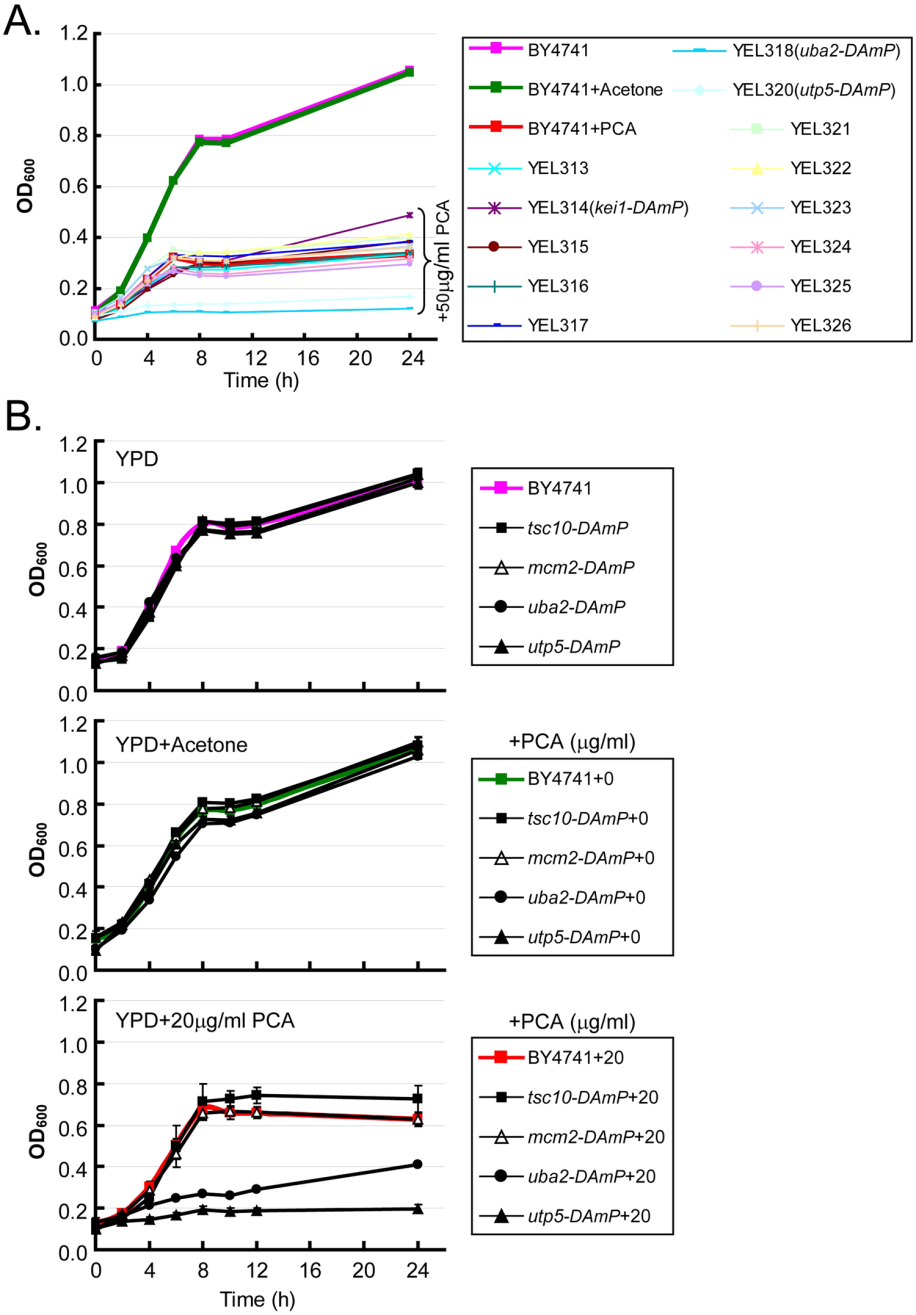
Determining and confirming the sensitivity of *S*. *cerevisiae* cells to PCA. (**A**) Determining the sensitivity of *S*. *cerevisiae* cells to 50 μg/ml PCA. Cellular OD_600_ was measured with an iMark microplate reader for cells at initial OD_600_ = 0.1 in YPD treated with or without 50 μg/ml PCA at the indicated times (intervals of 2 h, to 12 and 24 h). Cells in YPD without additive (−) or with acetone only (+0) were used as controls. YEL, yeast essential library. The full names of specific mutants with obvious sensitivity changes are given on the figure. (**B**) Confirming the sensitivity of *S*. *cerevisiae* cells to 20 μg/ml PCA. The screened mutants with obvious changes in resistance or sensitivity to PCA, determined with the method shown in panel A, were cultured in YPD to log phase and further tested in YPD without additive (upper), YPD with acetone only (middle) and YPD with 20 μg/ml PCA (bottom) to confirm their responses to PCA.

The global transcriptional response to a given stimulus indicates that biological systems use highly complex, interrelated metabolic and signaling pathways based on interacting gene networks^13^. Dynamic changes in key regulatory elements of molecular networks govern the response of an organism to a given stimulus^14^. To explore the potential molecular network and key regulatory elements involved in the inhibition or killing of yeasts by PCA, we used a microarray analysis to determine and compare the levels of different mRNAs in wild-type cells treated with or without 50 μg/ml PCA, the half-maximal inhibitory concentration (IC_50_, as reported previously^15^) at pH 5.7. Among the 10,715 mRNA fragments detected, the expression of 760 mRNAs was clearly upregulated and expression of 385 mRNAs was downregulated (Figure S1A and Table S2). We tested the sensitivity to PCA of three mutants without expression of the most strongly upregulated genes and two mutants without expression of the most strongly downregulated genes. We compared them with a known PCA-resistant control yeast, *tsc10-DAmP*, and a PCA-sensitive control yeast, *uba2-DAmP*, as shown in Fig. 2B, during culture on YPD plates containing different concentrations of PCA at pH 5. Unlike *tsc10-DAmP* and *uba2-DAmP*, the selected mutants showed no marked growth defects compared with wild-type BY4741 (Figure S1B). Therefore, the changes in the mRNA expression of these genes did not correlate with the sensitivity to PCA of their mutants, so we did not pursue this line of investigation.

### Growth sensitivity of yeast mutants to PCA

**Figure 3:**
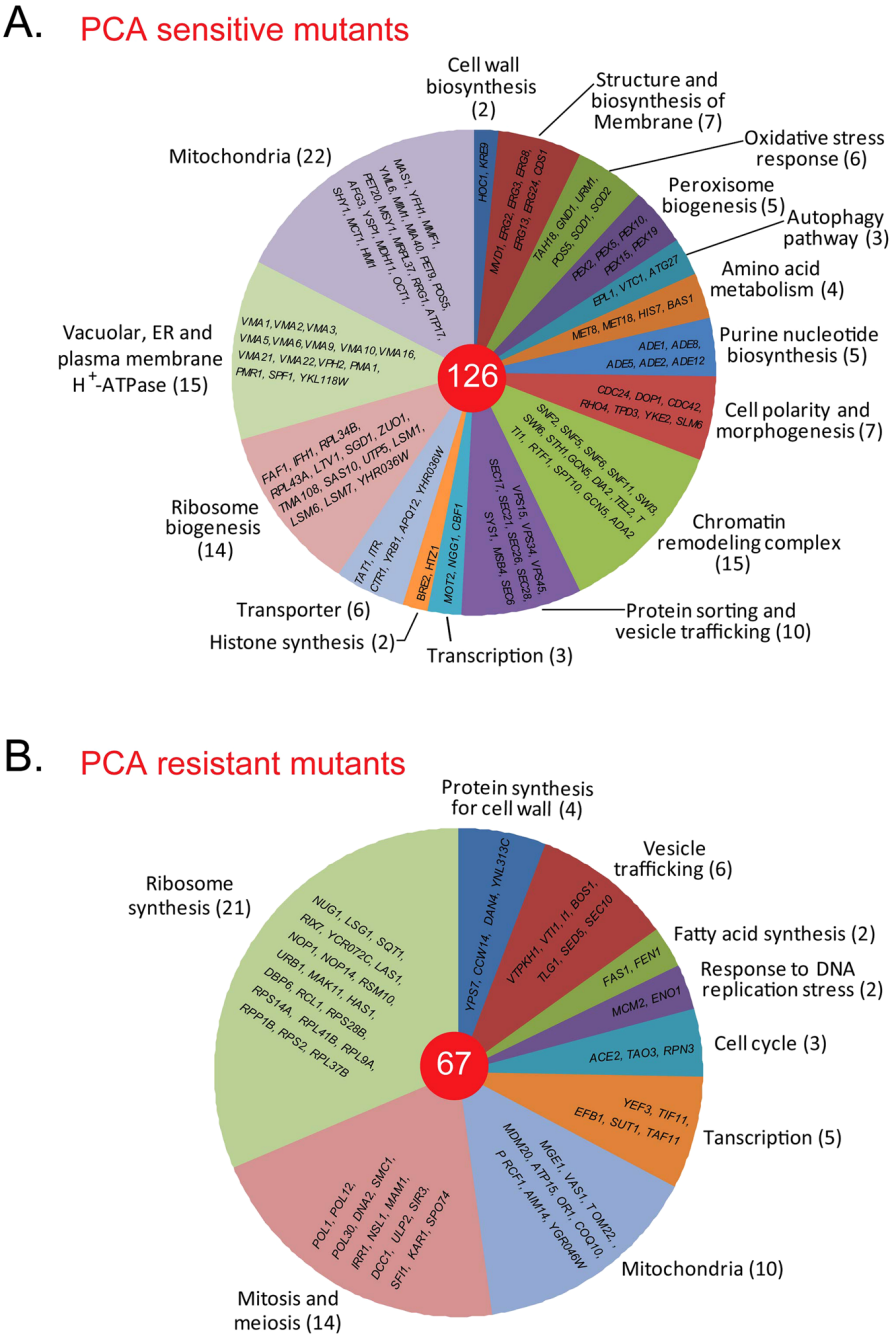
Summary of PCA-sensitive and -resistant mutants detected by screening a *S*. *cerevisiae* mutant library. (**A**) *Saccharomyces cerevisiae* mutants with increased sensitivity to PCA. The categories of metabolism are presented as a pie chart, with the number of genes in parentheses. Detailed genes are listed on the pie. Total numbers of genes are listed in the red circle in the middle. (**B**) *Saccharomyces cerevisiae* mutants with increased resistance to PCA. Data are presented as in panel A.

Yeast mutant collections have been used to investigate drug targets^16^. To identify the potential cellular target(s) of PCA and to explore the physiological processes that are inhibited by PCA, we screened yeast mutant libraries from two collections (YSC1053, Yeast MATa Collection of 5154 nonessential genes; YSC5095, Yeast DAmP Library of 842 essential genes) from Thermo Scientific Open Biosystems (Waltham, MA, USA). We tested the mutant strains for their sensitivity to 50 μg/ml PCA (the IC_50_ for PCA on wild-type BY4741 cells) after they were grown to stationary phase and inoculated at 0.1 initial optical density at 600 nm (OD_600_), with the goal of screening the whole yeast mutant library in batches as described in Materials and methods. The growth curves for mutant cells between 0 and 24 h were compared with the growth curve for wild type BY4741 treated with 50 μg/ml PCA set as the reference. The mutants that grew more rapidly than wild-type cells were considered PCA-resistant, while strains that grew more slowly were considered PCA-sensitive. Mutant cell cultures with an OD_600_ that was 10% higher or lower than that of the wild-type after 10 h were selected. By screening two representative batches, we identified *uba2-DAmP* and *utp5-DAmP* as PCA-sensitive mutants in one batch (Fig. 2A), and *tsc10-DAmP* as a PCA-resistant mutant in the other batch (data not shown). To confirm the PCA sensitivity of these mutants, we compared their growth curves with that of wild-type BY4741 cells grown under three conditions: in YPD alone, in YPD + acetone, and in YPD + 20 μg/ml PCA. In this assay, we intentionally reduced the concentration of PCA from 50 to 20 μg/ml so that the growth difference between the wild-type and PCA-sensitive mutant cells could be seen more clearly. We confirmed by this analysis that *uba2-DAmP* and *utp5-DAmP* were PCA-sensitive, whereas *tsc10-DAmP* was PCA-resistant (Fig. 2B). The resistance or sensitivity to PCA of mutants in the *S*. *cerevisiae* mutant library was then determined in a preliminary screen using only PCA as described in Fig. 2A, and verified in sensitivity-confirming experiments performed under the three conditions described in Fig. 2B. The genes affected in the mutants which appeared to confer resistance or sensitivity to PCA were grouped and clustered according to their functions and sensitivity to PCA. A cluster includes at least two genes functioning in the same metabolic pathway and their mutants showing the same sensitivity to PCA. The total of 126 PCA-sensitive mutants from 16 clusters and 67 PCA-resistant mutants from 9 clusters were identified in various metabolic pathways, including the vesicular trafficking and autophagy pathways (Fig. 3). These results indicate that multiple pathways are affected by PCA in *S*. *cerevisiae*.

### Mutants defective in vacuolar ATPase and protein sorting have increased sensitivity to PCA

**Figure 4:**
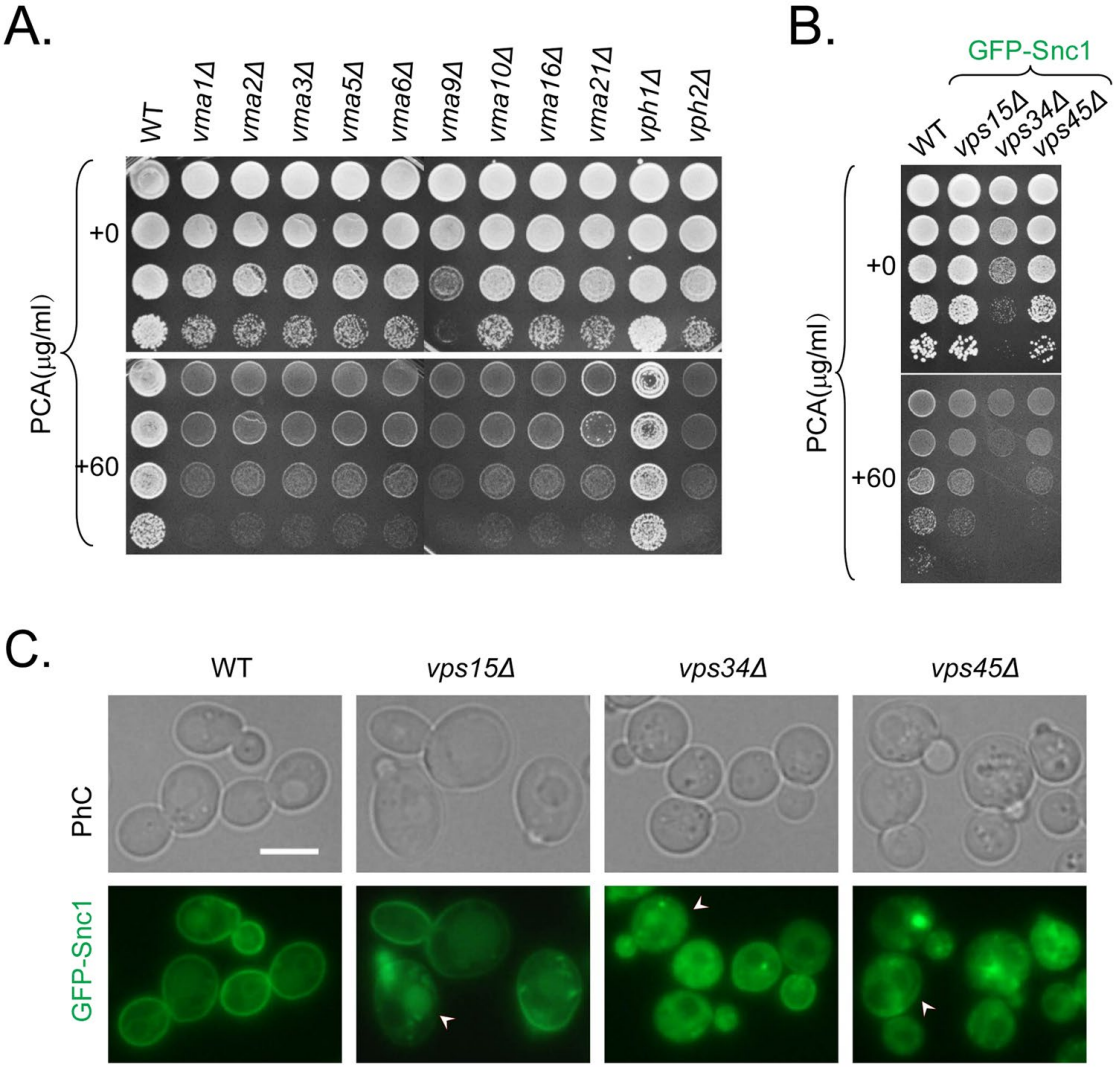
Mutants defective in vacuolar ATPase and protein sorting show increased sensitivity to PCA. (**A**) Overnight YPD-cultured wild-type and vacuolar ATPase-mutant cells were spotted onto YPD plates containing 0 or 60 μg/ml PCA and incubated for 2 days at 26 °C. (**B**) Overnight YPD-cultured wild-type and protein sorting-mutant cells tagged with GFP-Snc1 on the chromosome were spotted onto YPD plates containing 0 or 60 μg/ml PCA and incubated for 2 days at 26 °C. (**C**) Wild-type and protein sorting-mutant cells tagged with GFP-Snc1 on the chromosome were grown to mid-log phase in YPD for 5 h to examine GFP-Snc1 localization. White arrowheads indicate cells in which the polar distribution of GFP-Snc1 was lost. The bar represents 5 μm.

As shown in Fig. 3, the mutant library screening results showed that some mutants defective in vacuolar ATPase or protein sorting had increased sensitivity to PCA. To confirm these results, wild-type and related deletion mutant cells were grown on YPD plates with 0 or 60 μg/ml PCA for 2 days and then photographed. Mutants other than *vph1∆* showed increased sensitivity to PCA, and cells tagged with GFP-Snc1 were more sensitive to PCA (Fig. 4A,B). Stv1, the yeast isoform of Vph1, complements the absence of Vph1^17^, which may be why *vph1∆* is insensitive to PCA. To further validate the requirement for vacuolar protein sorting in protein trafficking, we examined the localization of a representative marker of protein trafficking, Snc1, in wild-type and mutant cells with live-cell fluorescence microscopy. Snc1 is a v-SNARE protein that is transported from the endoplasmic reticulum (ER) to the Golgi and plays a role in the fusion of trans-Golgi vesicles with the plasma membrane (PM). After multiple rounds of vesicle fusion, it is recycled from the PM back to the Golgi via endosomes^18^. Green fluorescent protein (GFP)-Snc1 is blocked in different compartments depending on the regulation step at which a mutation occurs. Therefore, this construct is widely used for monitoring vesicular transport processes in living cells^19^. In the *vps34∆* and *vps45∆* mutants, GFP-Snc1 accumulated as intracellular puncta in the cytoplasm, in contrast to the polar distribution of GFP-Snc1 in the buds and necks of wild-type cells (Fig. 4C). The trafficking defect of GFP-Snc1 in *vps15∆* was very weak, but nonetheless increased intracellular GFP-Snc1 puncta in the cytoplasm could be seen. A previous study showed that V-ATPase is required for protein endocytic recycling and autophagic processes^20, 21^. Our results, combined with these reports, show that some mutants with increased sensitivity to PCA are defective in protein sorting and endocytic recycling.

### PCA disrupts protein transport in *S*. *cerevisiae* under normal growth conditions

**Figure 5:**
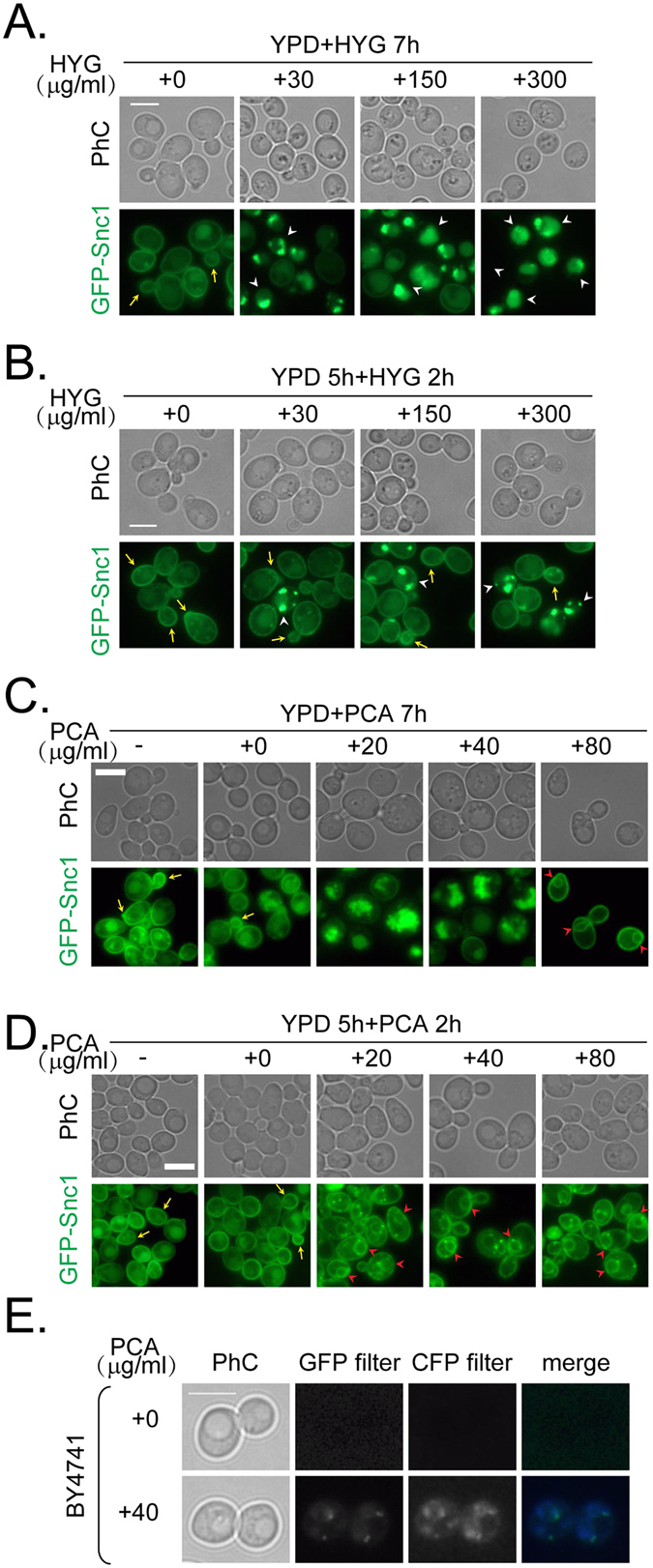
Treatment with PCA disrupts the trafficking of Snc1 differently than hygromycin B. (**A**) Wild-type cells tagged with GFP-Snc1 were grown in YPD to an initial OD_600_ = 0.1 with the indicated concentrations of HYG for 7 h. (**B**) Wild-type cells tagged with GFP-Snc1 were grown in YPD to mid-log phase for 5 h and treated with the indicated concentrations of HYG in YPD for 2 h. Arrows indicate cells with polarized Snc1 at the bud or bud tip. White arrowheads indicate intracellular GFP-Snc1 puncta (**A** and **B**). (**C** and **D**) Cells were grown and treated as in panels A and B, except that PCA was used at the indicated concentrations. Red arrowheads indicate the ER ring inside the cell (**C** and **D**). (**E**) Wild-type cells without fluorescently tagged protein were grown to mid-log phase in YPD and treated with or without 40 μg/ml PCA in YPD for 2 h. Cells were observed under a microscope with a phase-contrast set and GFP or CFP filters. The bar represents 5 μm. Results shown in this figure represent two independent experiments.

The yeast mutants hypersensitive to hygromycin B are reported to be defective in vesicular and vacuolar trafficking^22^. Therefore, we examined whether vesicular traffic in wild-type *S*. *cerevisiae*is was disrupted by hygromycin B or PCA using Snc1 as a marker of vesicular trafficking. When we examined how hygromycin B affects the localization of GFP-Snc1 under the conditions shown in Fig. 5A, we found that polar transport of GFP-Snc1 to the PM at the cellular bud or neck was almost totally lost. Instead, GFP-Snc1 appeared as intracellular puncta in a dose-dependent manner (Fig. 5A). In contrast, a period of growth for 5 h (allowing the cells to reach the mid-log phase), followed by treatment with hygromycin B for 2 h impaired GFP-Snc1 transport less markedly. Some polar staining for GFP-Snc1 was retained, but with fewer intracellular puncta, even at the lethal concentration of hygromycin B (300 μg/ml; Fig. 5B). When 20–40 μg/ml PCA was added to the growing yeast cells at an initial OD_600_ of 0.1 and incubated for 7 h in YPD medium, GFP-Snc1 accumulated in the vacuoles (seen in phase-contrast [PhC] images; Fig. 5C). At 80 μg/ml PCA, GFP-Snc1 showed a clear ER distribution, and polar transport of GFP-Snc1 to the plasma membrane was disrupted at the bud or neck (Fig. 5C). However, if the cells were grown to mid-log phase and then treated with different concentrations of PCA for 2 h, GFP-Snc1 appeared as dots around the ER, and polar transport of GFP-Snc1 to the plasma membrane was gradually lost in a dose-dependent manner (Fig. 5D). These fluorescent dots appeared in the cells after PCA treatment regardless of the tagged proteins used (data not shown), so it seemed likely that the dots corresponded to the intrinsic fluorescence of PCA. As a confirmation of this possibility, when BY4741 yeast (with no fluorescently tagged protein) was treated with or without PCA and then observed under a GFP or cyan fluorescent protein (CFP) filter, fluorescent dots appeared in the PCA-treated cells (Fig. 5E). Therefore, we concluded that the dots were attributable to PCA autofluorescence and disregarded them in subsequent experiments.

**Figure 6:**
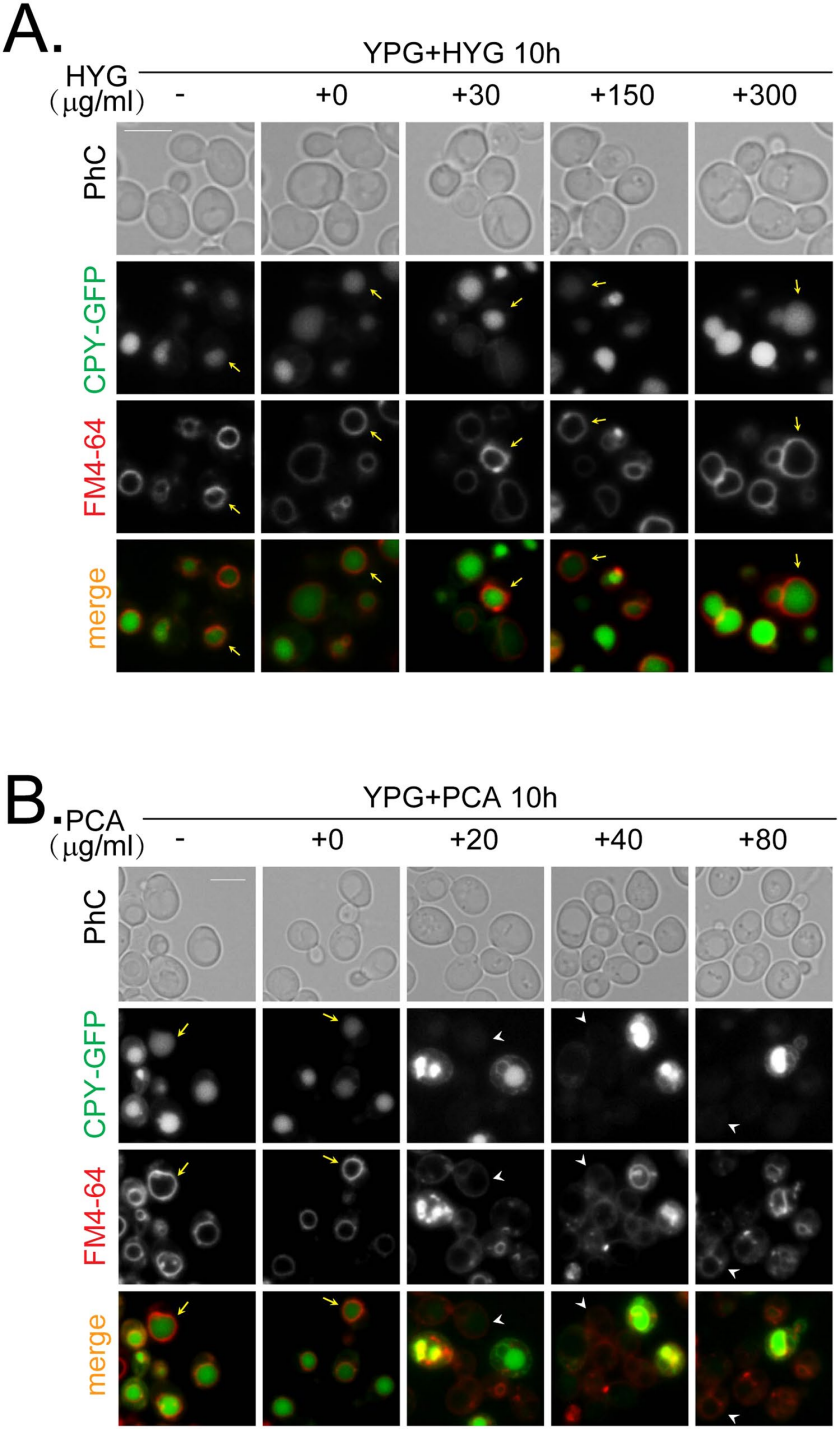
Treatment with PCA disrupts the trafficking of CPY differently than hygromycin B. (**A**) Wild-type cells tagged with CPY-GFP were grown in YPG at an initial OD_600_ = 0.1 with different concentrations of HYG in YPD for 10 h. FM4-64 was added in the last hour to stain the vacuoles. (**B**) Cells were grown and treated as in panel A, except that PCA was used at the indicated concentrations. The bar represents 5 μm. Arrows indicate internalized CPY in the vacuoles. Arrowheads indicate disrupted CPY internalization. Results shown in this figure represent two independent experiments.

To determine if general protein trafficking was disrupted by PCA, we analyzed the localization of another vesicular trafficking marker, carboxy-peptidase Y (CPY), in *S*. *cerevisiae*. CPY is a vacuolar glycoprotein that is transported from the ER to the vacuoles via the Golgi, and along the way, it undergoes a series of characteristic modifications that can be used to monitor its trafficking^23^. Hygromycin B did not block the transport of CPY-GFP to the vacuoles (Fig. 6A) because CPY-GFP signals localized to the vacuoles, which were stained with the lipophilic dye FM4-64. However, PCA disrupted the transport of CPY-GFP to the vacuoles in a dose-dependent manner (Fig. 6B), since negligible CPY-GFP signals were seen in the vacuoles. At the same time, FM4-64 did not completely reach the vacuolar membrane.

Taken together, our data indicate that PCA disrupts the polar transport of GFP-Snc1 to the cellular bud or neck and transport of CPY-GFP to the vacuoles, suggesting that PCA inhibits fungal growth by a different mechanism than hygromycin B.

### PCA alters autophagic flux in *S*. *cerevisiae* under starvation

Autophagy is a key pathway mediating stress-induced metabolic adaptation or damage, allowing eukaryotic cells to survive^24^. The autophagic process is highly dependent on vesicular trafficking^25^. Because PCA disrupts vesicular trafficking in yeast, we wondered whether PCA affects autophagy.

Atg8 is one of the core autophagy machinery proteins in *S*. *cerevisiae* and can be used to observe the status of autophagy by monitoring the localization and degradation of GFP-Atg8^26^. *S*. *cerevisiae* proaminopeptidase I (prApe1) is also processed by vacuolar hydrolases into the mature form (mApe1)^27^. Both prApe1 and mApe1 can be distinguished because of their different size^27^.

**Figure 7:**
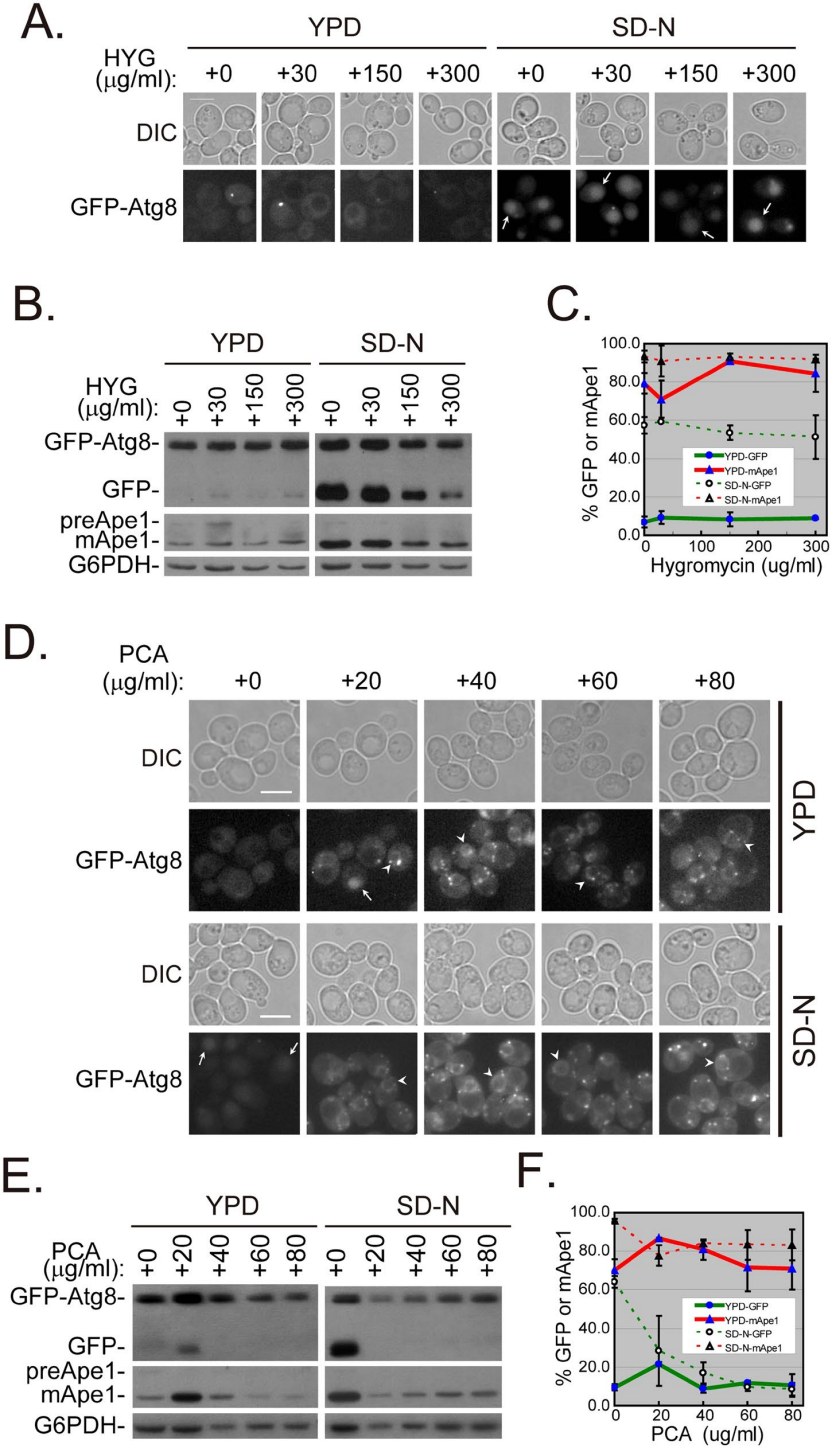
Treatment with PCA alters the autophagy process differently than hygromycin B. (**A**) HYG treatment did not disrupt the localization of GFP-Atg8. (**B**) HYG treatment slightly retarded GFP-Atg8 processing in a dose-dependent manner in SD-N. (**C**) Quantification of blots in panel B. (**D**) PCA treatment altered the localization of GFP-Atg8 in YPD and SD-N differently. (**E**) PCA treatment induced slight autophagy at low PCA concentrations in YPD and impaired autophagy in SD-N. (**F**) Quantification of blots in panel E. PhC, phase contrast. The bar represents 5 μm. Arrows indicate cells with GFP inside the vacuoles. Arrowheads indicate the ER ring inside the cell. The results shown represent two independent experiments.

We monitored the localization and degradation of GFP-Atg8 in cells after treatment with hygromycin B or PCA at various concentrations in rich (YPD) or starvation medium (SD-N). In YPD, increasing concentrations of hygromycin B did not accelerate the entry of GFP-Atg8 into the vacuoles, and 30 μg/ml hygromycin B only slightly reduced the maturation of aminopeptidase 1 (Ape1) (Fig. 7A–C). When the cells were further treated in SD-N for 2 h, the delivery of GFP-Atg8 to the vacuoles decreased as the hygromycin concentration increased (Fig. 7A–C).

Even though GFP-Atg8 was distributed in the cytosol of cells in YPD, a small proportion was delivered to the vacuole after treatment with 20 μg/ml PCA, indicating that autophagy was slightly induced. However, as the PCA concentration increased, GFP-Atg8 remained in the cytosol (Fig. 7D upper). The immunoblotting results showed that GFP-Atg8 degradation and prApe1 maturation increased transiently after treatment with 20 μg/ml PCA, but did not change at higher concentrations of PCA under growth conditions (YPD; Fig. 7E left and 7F). When cells in log phase were transferred into SD-N, the delivery of GFP-Atg8 to the vacuoles was blocked by the addition of PCA (20–80 μg/ml; Fig. 7D bottom). Consistent with this, GFP-Atg8 degradation was inhibited by the addition of PCA. Although the prApe1 could mature, the proportion of mApe1 decreased significantly with the addition of PCA in SD-N (Fig. 7E right and 7F).

Taken together, our data indicate that PCA significantly inhibits GFP-Atg8 processing under conditions that induce autophagy (i.e., SD-N). This activity differs from the action of hygromycin B.

## Discussion

Understanding the mechanism underlying the inhibitory and killing effects of PCA on fungi will extend its application and allow its formulation to be improved. We found that PCA inhibits the growth of both *S*. *cerevisiae* and *C*. *albicans*, especially at low pH, and that PCA disrupts vesicular trafficking and autophagy in *S*. *cerevisiae*. These results extend our understanding of the cellular effects of PCA on fungi, and should guide its application in medicine and agriculture.

We found the inhibitory effect of PCA on yeasts was not only dose-dependent, but was inversely dependent on pH, different from pH-dependent hygromycin B. Most likely it is because PCA needs acidic conditions to maintain its acidic toxic state. This inhibitory effect is consistent with the report that the acidification of growth medium by *P*. *aeruginosa* activates the toxicity of PCA toward *C*. *elegans* ^28^. This finding also underscores the fact that pH should be considered when PCA is used in medicine or agriculture.

What is the underlying molecular mechanism for the inhibitory effects of PCA on pathogenic microorganisms? Although the mechanism has been widely examined, it is still puzzling because the effects are so diverse. It has been reported that phenazines from *P*. *aeruginosa* act as virulence factors during opportunistic infection of host cells^29^. It has also been reported that *P*. *aeruginosa* alters host cell functions by secreting PCA, which acts in part to increase the formation of oxidants^30^. Furthermore, increased ROS and reduced ROS-scavenging enzyme activities were reported to be responsible for the biological effects of PCA on the pathogenic bacterium *Xanthomonas oryzae* pv. *oryzae* ^31^. Our finding that PCA disturbs vesicular trafficking and autophagy extends our understanding of the mechanisms by which PCA inhibits and kills *S*. *cerevisiae* (Figs 4–7). The blockage of autophagy by PCA under starvation conditions is intriguing because it could increase the susceptibility of the yeast to stress conditions and reduce its survival. Similar examples exist in macrophages where vancomycin blocks autophagy and increases the inflammatory responses^32^. However, whether other actions are involved in the inhibitory effect of PCA, which effects are dominant and the relationship between different effects warrant further investigation.

Vesicular trafficking and autophagy are two major pathways in living cells which maintain cellular functions. Although vesicular- and vacuolar-trafficking mutants are generally sensitive to hygromycin B^22^ and PCA (Fig. 4A,B), the different changes to vesicular trafficking and autophagy induced in yeast by hygromycin B and PCA imply that the actions of PCA in these pathways are not general antibiotic effects. We conservatively postulate that PCA treatment directly disrupts general intracellular trafficking or the characteristics of trafficking markers, including aggregation of proteins in response to misfolding or other intracellular toxicity responses.

Although it is known that the autophagic process is highly dependent on vesicular trafficking^27^, it is still not clear how vesicular trafficking exactly contributes to autophagy or how autophagy regulates vesicular trafficking. In this study, we found PCA affected both vesicular trafficking and autophagy, but we are unclear about their contributions and interconnection to the inhibitory effect of PCA. Although some mutants in vesicular trafficking or autophagy did show increased sensitivity or resistance to PCA, our data from the growth sensitivity tests on the yeast mutant library and the microarray analysis did not specifically point to vesicular trafficking and autophagy (Figs 2 and 3). The yeast microarray analysis also did not identify the target(s) of PCA, but our analysis of the yeast mutant library showed that mutants involving cellular structure, functional organelles or basic metabolism were more sensitive to PCA than wild-type cells, suggesting that PCA affects homeostasis, transport functions and metabolism of cells in a complex way. In the future, we hope we can streamline how PCA inhibits autophagy and contributes to cell death through autophagic processes.

In summary, our results indicate that PCA exerts broad effects on yeast, including the alteration of vesicular trafficking and autophagy. These effects are inferred to be conserved across fungal pathogens based on the overall evolutionary conservation of this taxon. The greater inhibition at lower pH and its blocking of autophagy under starvation conditions are relevant to the application of PCA in both medicine and agriculture.

## Materials and Methods

### Strains and reagents

The yeast strains used in this study are listed in Table S1.

The reagents and drugs used were as follows: acetone (cat. no. 31025, Shanghai Lingfeng Chemical Reagent Co. Ltd., Shanghai, China), geneticin (cat. no. A1720, Sigma-Aldrich, St. Louis, MO, USA), 100 mg/ml hygromycin B (cat. no. H 8080, Beijing Solarbio Science & Technology, Beijing, China), and phenazine-1-carboxylic acid (PCA, prepared from *Pseudomonas* sp. and purified with high-performance liquid chromatography; Shanghai Jiao Tong University). PCA (purity > 99%) was prepared as a 2 mg/ml stock solution in acetone.

### Growth of yeast for drug sensitivity testing and fluorescence analysis

Cells were grown in YPD without antibiotics unless stated. The experiments with PCA were also performed in parallel with hygromycin B except that different concentrations of drugs were used. For hygromycin B or PCA inhibition of growth assays on plates, overnight cultures of cells in the first row of wells in 96-well plates were adjusted to about OD_600_ = 1, and were further diluted 1:10 with sterile distilled H_2_O. They were spotted onto solid YPD with the indicated concentrations of hygromycin B or PCA at pH 4, 5 or 5.7 using a 48-pin manipulator. The plates were incubated at 26 °C and photographed at 72 h. The cells on the plates containing 300 μg/ml hygromycin B or 80 μg/ml PCA were replica-plated at 72 h on YPD plates (pH 5.7), grown at 26 °C for 48 h and photographed.

For hygromycin B or PCA inhibition of growth assays in liquid, samples were prepared as described below. GFP-Snc1-tagged cells were grown in 5 ml of YPD in 100 ml flasks at 26 °C with rotation at 200 rpm with hygromycin B or PCA added at different times: (1) Cells grown in YPD containing different concentrations of hygromycin B for 7 h to reach log phase; or (2) cells grown in YPD without hygromycin B or PCA for 5 h to reach mid-log phase, then in YPD with different concentrations of hygromycin B or PCA for 2 h to observe their fluorescence. CPY-GFP-tagged cells were grown and treated similarly, except that YPG (containing galactose instead of dextrose to induce CPY-GFP expression) was used and the cells were grown for 10 h instead of 7 h to log phase (cells grew slower in YPG than in YPD) to observe their fluorescence. GFP-Atg8-tagged cells were grown in 5 ml of YPD in 100 ml flasks at 26 °C with rotation at 200 rpm with hygromycin B or PCA added at different times: (1) Cells grown in YPD with different concentrations of hygromycin B or PCA for 7 h to reach log phase; or (2) cells grown in YPD without hygromycin B or PCA for 5 h to mid-log phase, then shifted immediately to SD-N medium (0.17% yeast nitrogen base without amino acids plus ammonium sulfate with 2% glucose) containing different concentrations of hygromycin B or PCA and incubated for 2 h at 26 °C. Fluorescent images were taken with a Nikon Eclipse Ti inverted microscope (Tokyo, Japan). Five fields were visualized for each sample. At least two independent experiments were performed.

We established the IC_50_ of PCA in the yeast cells and used it to screen the yeast mutant library or in the microarray assay. The sensitivity of wild-type yeast BY4741 was tested with a series of PCA concentrations in 96-well plates, using an iMark microplate reader (Bio-Rad, Hercules, CA, USA) as described previously^15^. The IC_50_ of PCA was used to treat the yeast mutant library and wild-type cells, and their growth was recorded on growth curves to determine the sensitivity of the mutant yeast cells. The mutant yeast cells were streaked on YPD + G418 plates and grown at 26 °C for 3–4 days, and wild-type cells were streaked on YPD plates without antibiotic. The extremely small colonies on the YPD + G418 plates and/or the very slowly growing cells in liquid YPD under the same conditions were eliminated before PCA screening. Wild-type BY4741 and mutant cells were inoculated at OD_600_ = 0.1 in YPD containing PCA to a final volume of 100 μl in 96-well plates. In the first round of screening, mutant cells were examined with YPD + 50 µg/ml PCA, whereas wild-type cells were always examined under two additional conditions, with YPD or YPD + acetone. The OD_600_ of the cells was measured with an iMark microplate reader (Bio-Rad) at the indicated time points (intervals of 2 h up to 12 and 24 h). Cells in YPD without additive (−) or with acetone only (+0) were used as controls. The growth curve of BY4741 cells treated with 50 μg/ml PCA was used as the reference. When the growth curves of mutants were located above that of BY4741, they were considered more resistant to PCA. In contrast, mutants with growth curves located below those of BY4741 were considered more sensitive to PCA. In confirmation experiments, all wild-type and mutant cells were tested with YPD, YPD + acetone and YPD + 20 µg/ml PCA. The IC_50_ of PCA was also used to treat wild-type yeast cells in the microarray assay according to the manufacturer’s instructions (Bioassay Laboratory of Capital Bio Corporation, Beijing, China). Differences in mRNA levels in the control (acetone) and PCA-treated (PCA dissolved in acetone) samples were compared for each gene.

### Immunoblotting analysis of Atg8 and Ape1

The effects of drug treatment on autophagic processes were determined with immunoblotting analyses of Atg8 and Ape1. Half the GFP-Atg8-tagged cells were observed for fluorescence, and the rest were subjected to an immunoblotting analysis to determine the effect of PCA treatment on the autophagy process. The cells were lysed as previously described^33^. The blots were probed with an anti-GFP antibody (cat. no. sc-9996, Santa Cruz Biotechnology, Dallas, TX, USA) to monitor the processing of GFP-Atg8 to GFP, with anti-Ape1 antibody (a gift from Dr. Y. Ohsumi, Tokyo Institute of Technology) to monitor the processing of Ape1 from prApe1 to mApe1, and an anti-glucose-6-phosphate dehydrogenase (G6PDH) antibody (cat. no. A9521, Sigma-Aldrich) to analyze G6PDH as the loading control. The percentage of processed GFP was calculated as (GFP/[GFP−Atg8 + GFP]) × 100 and the percentage of mApe1 was calculated as (mApe1/[prApe1 + mApe1]) × 100. The data presented were the means ± standard deviation of two independent experiments.

## Electronic supplementary material

suppl figure and tables

## References

[1] Puopolo G (2013). Insights on the susceptibility of plant pathogenic fungi to phenazine-1-carboxylic acid and its chemical derivatives. Nat Prod Res.

[2] Gorantla JN (2014). Purification and characterization of antifungal phenazines from a fluorescent *Pseudomonas* strain FPO4 against medically important fungi. J Mycol Med.

[3] Park GK, Lim JH, Kim SD, Shim SH (2012). Elucidation of antifungal metabolites produced by *Pseudomonas aurantiaca* IB5-10 with broad-spectrum antifungal activity. J Microbiol Biotechnol.

[4] Karnetova J, Tax J, Stajner K, Vanek Z, Krumphanzl V (1983). Production of phenazines by *Streptomyces cinnamonensis*. Folia Microbiol (Praha).

[5] Geiger A, Keller-Schierlein W, Brandl M, Zahner H (1988). Metabolites of microorganisms. 247. Phenazines from *Streptomyces antibioticus*, strain Tu 2706. J Antibiot (Tokyo).

[6] Arseneault T, Goyer C, Filion M (2013). Phenazine production by *Pseudomonas* sp. LBUM223 contributes to the biological control of potato common scab. Phytopathology.

[7] St-Onge R, Gadkar VJ, Arseneault T, Goyer C, Filion M (2011). The ability of *Pseudomonas* sp. LBUM 223 to produce phenazine-1-carboxylic acid affects the growth of *Streptomyces scabies*, the expression of thaxtomin biosynthesis genes and the biological control potential against common scab of potato. FEMS Microbiol Ecol.

[8] Jasim B (2014). Phenazine carboxylic acid production and rhizome protective effect of endophytic *Pseudomonas aeruginosa* isolated from *Zingiber officinale*. World J Microbiol Biotechnol.

[9] Lee JY, Moon SS, Hwang BK (2003). Isolation and *in vitro* and *in vivo* activity against *Phytophthora capsici* and *Colletotrichum orbiculare* of phenazine-1-carboxylic acid from *Pseudomonas aeruginosa* strain GC-B26. Pest Manag Sci.

[10] D’Aes J (2011). Biological control of Rhizoctonia root rot on bean by phenazine- and cyclic lipopeptide-producing *Pseudomonas* CMR12a. Phytopathology.

[11] Morales DK (2010). Antifungal mechanisms by which a novel *Pseudomonas aeruginosa* phenazine toxin kills *Candida albicans* in biofilms. Mol Microbiol.

[12] Huang H, Sun L, Bi K, Zhong G, Hu M (2016). The effect of phenazine-1-carboxylic acid on the morphological, physiological, and molecular characteristics of *Phellinus noxius*. Molecules.

[13] Nanduri B, Shack LA, Burgess SC, Lawrence ML (2009). The transcriptional response of *Pasteurella multocida* to three classes of antibiotics. BMC Genomics.

[14] Weston AD, Hood L (2004). Systems biology, proteomics, and the future of health care: toward predictive, preventative, and personalized medicine. J Proteome Res.

[15] Zhao X, Zhu X, He Y, Liang Y (2015). *CDS1* is required for proper vacuole morphology but not for autophagy in *Saccharomyces cerevisae*. Journal of Nanjing Agricultural University.

[16] Scherens B, Goffeau A (2004). The uses of genome-wide yeast mutant collections. Genome Biol.

[17] Manolson MF (1994). *STV1* gene encodes functional homologue of 95-kDa yeast vacuolar H(+)-ATPase subunit Vph1p. J Biol Chem.

[18] Lewis MJ, Nichols BJ, Prescianotto-Baschong C, Riezman H, Pelham HRB (2000). Specific retrieval of the exocytic SNARE Snc1p from early yeast endosomes. Mol Biol Cell.

[19] Zou S (2012). Modular TRAPP complexes regulate intracellular protein trafficking through multiple Ypt/Rab GTPases in *Saccharomyces cerevisiae*. Genetics.

[20] Ueno K (2014). V-ATPase-dependent luminal acidification is required for endocytic recycling of a yeast cell wall stress sensor, Wsc1p. Biochem Biophys Res Commun.

[21] Mijaljica D, Prescott M, Devenish RJ (2011). V-ATPase engagement in autophagic processes. Autophagy.

[22] Banuelos MG (2010). Genomic analysis of severe hypersensitivity to hygromycin B reveals linkage to vacuolar defects and new vacuolar gene functions in *Saccharomyces cerevisiae*. Curr Genet.

[23] Stevens T, Esmon B, Schekman R (1982). Early stages in the yeast secretory pathway are required for transport of carboxypeptidase Y to the vacuole. Cell.

[24] Kroemer G, Marino G, Levine B (2010). Autophagy and the integrated stress response. Mol Cell.

[25] Lamb CA, Longatti A, Tooze SA (2016). Rabs and GAPs in starvation-induced autophagy. Small GTPases.

[26] Suzuki K (2001). The pre-autophagosomal structure organized by concerted functions of *APG* genes is essential for autophagosome formation. EMBO J.

[27] Klionsky DJ, Cueva R, Yaver DS (1992). Aminopeptidase I of *Saccharomyces cerevisiae* is localized to the vacuole independent of the secretory pathway. J Cell Biol.

[28] Cezairliyan B (2013). Identification of *Pseudomonas aeruginosa* phenazines that kill *Caenorhabditis elegans*. PLoS Pathog.

[29] Recinos DA (2012). Redundant phenazine operons in *Pseudomonas aeruginosa* exhibit environment-dependent expression and differential roles in pathogenicity. Proc Natl Acad Sci USA.

[30] Look DC (2005). Pyocyanin and its precursor phenazine-1-carboxylic acid increase IL-8 and intercellular adhesion molecule-1 expression in human airway epithelial cells by oxidant-dependent mechanisms. J Immunol.

[31] Xu S (2015). Effects of phenazine-1-carboxylic acid on the biology of the plant-pathogenic bacterium *Xanthomonas oryzae* pv. oryzae. Pestic Biochem Physiol.

[32] Ha YE (2015). Vancomycin blocks autophagy and induces interleukin-1beta release in macrophages. . J Antibiot (Tokyo).

[33] Chen Y (2014). A Vps21 endocytic module regulates autophagy. Mol Biol Cell.

