# Supplementary material for: A genetic screen in combination with biochemical analysis in Saccharomyces cerevisiae indicates that phenazine-1-carboxylic acid is harmful to vesicular trafficking and autophagy
Source: Sci Rep. 2017 May 16;7:1967. doi: 10.1038/s41598-017-01452-6 (PMC5434042; doi:10.1038/s41598-017-01452-6)
Supplement: Supplementary file 1 — suppl figure and tables [file 41598_2017_1452_MOESM1_ESM.pdf]

SUPPORTING INFORMATION

A genetic screen in combination with biochemical analysis in *Saccharomyces cerevisiae* indicates that phenazine-1-carboxylic acid is harmful to vesicular trafficking and autophagy

Xiaolong Zhu[1], Yan Zeng[1], Xiu Zhao[1], Shenshen Zou[1], Ya-Wen He[2] & Yongheng Liang[1]

Figure S1. Microarray analysis and growth phenotypes of PCA-treated yeast. A. A scatter plot of mRNA levels in PCA-treated yeast cells against those in non-PCA-treated (control) yeast cells. Red dots represent increasing mRNA expression and green dots represent decreasing mRNA expression. Names of the genes with extraordinarily increased or reduced mRNA levels are shown. **B.** Mutations in genes with extraordinarily increased or reduced mRNA levels after treatment with PCA caused no altered growth sensitivity to PCA. *tsc10-DamP* was shown to be PCA-resistant and *uba2-DAmP* to be PCA-sensitive in the experiments illustrated in Figure 2. Both were used as controls.

Figure S1

A.

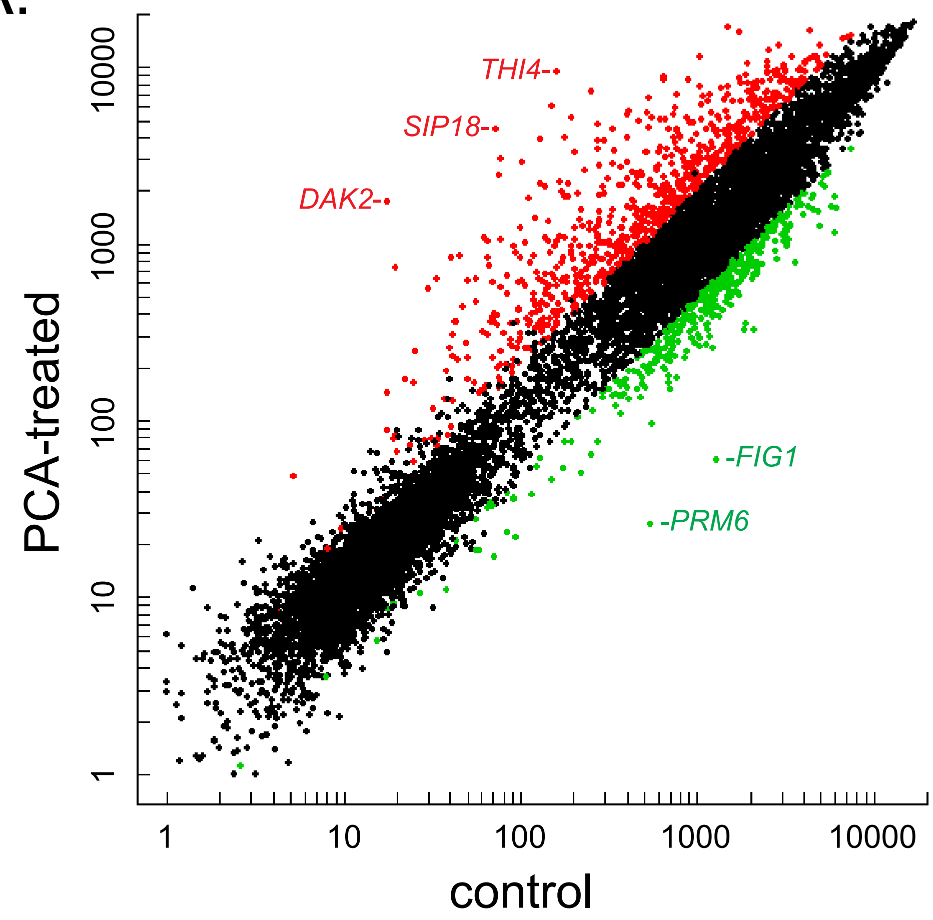
PCA-treated
control
THI4
SIP18
DAK2
FIG1
PRM6
1
10
100
1000
10000

B.

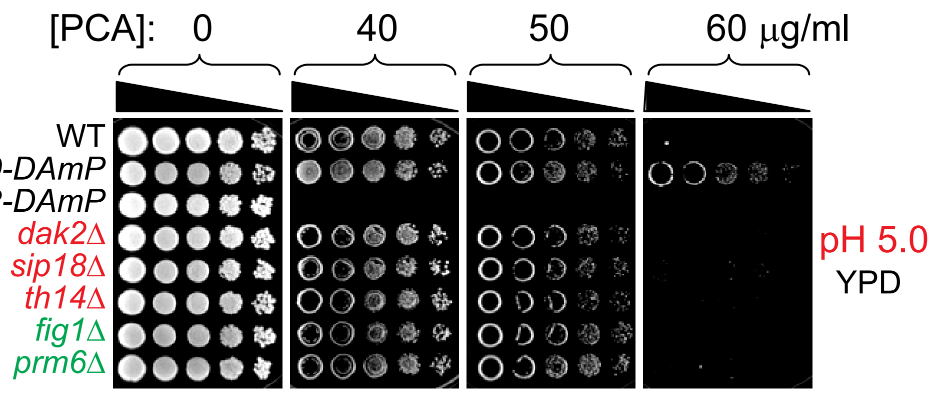
[PCA]: 0
40
50
60 µg/ml
WT
tsc10-DAmP
uba2-DAmP
dak2Δ
sip18Δ
th14Δ
fig1Δ
prm6Δ
pH 5.0
YPD

Table S1. Strains used in this study

Strain	Alias	Genotype	Source
YLY548	BY4741	*MAT a his3-1 leu2Δ met15Δ ura3Δ*	[1]
	Candida. albicans		Nanjing institute of skin disease, Chinese academy of sciences
YLY40	NSY128	MAT α *ade2 his3-Δ200 leu2-3,112 lys2-801 ura3-52 gal*	[2]
YLY130	GFP-Snc1	NSY128, *ura3::GFP-Snc1-URA3*	[3]
YLY915	SEY6210	*MATα ura3-52 leu2-3,112 his3-Δ200 trp1-Δ901 lys2-801 suc2-Δ9 mel GAL*	[4]
YLY3977		MAT a *ura3-52 leu2-3,112 his3-Δ200 ura3::CPY-GFP-URA3*	This study
YLY2422	GFP-Atg8	SEY6210*, ura3::GFP-Atg8-URA3*	[5]
YLY1770		Mat a *his3-Δ200 lys2-801 leu2-3,112* GFP-Snc1::*URA3*	This study
YLY5352		Mat a *his3-Δ200 lys2-801 leu2-3,112* GFP-Snc1::*URA3 vps15::KanMX*	This study
YLY5353		Mat a *his3-Δ200 lys2-801 leu2-3,112* GFP-Snc1::*URA3 vps34::KanMX*	This study
YLY5354		Mat a *his3-Δ200 lys2-801 leu2-3,112* GFP-Snc1::*URA3 vps45::KanMX*	This study
	YSC1053	Yeast MATa Collection for 5154 nonessential genes	Thermo Scientific Open Biosystems
	YSC5095	Yeast DAmP Library for 842 essential genes	Thermo Scientific Open Biosystems

REFERENCES

1 Brachmann, C. B. *et al.* Designer deletion strains derived from *Saccharomyces cerevisiae* S288C: a useful set of strains and plasmids for PCR-mediated gene disruption and other applications. *Yeast* **14**, 115-132, (1998).

2 Jedd, G., Mulholland, J. & Segev, N. Two new Ypt GTPases are required for exit from the yeast trans-Golgi compartment. *J Cell Biol* **137**, 563-580 (1997).

3 Zou, S. *et al.* Modular TRAPP complexes regulate intracellular protein trafficking through multiple Ypt/Rab GTPases in *Saccharomyces cerevisiae*. *Genetics* **191**, 451-460, (2012).

4 Robinson, J. S., Klionsky, D. J., Banta, L. M. & Emr, S. D. Protein sorting in *Saccharomyces cerevisiae*: isolation of mutants defective in the delivery and processing of multiple vacuolar hydrolases. *Mol Cell Biol* **8**, 4936-4948 (1988).

5 Chen, Y. *et al.* A Vps21 endocytic module regulates autophagy. *Mol Biol Cell* **25**, 3166-3177, (2014).

Table S2. Changes in mRNA levels in *S. cerevisiae* treated with 50 μg/ml PCA or no PCA. Red-labeled genes are upregulated; green-labeled genes are downregulated; the other 9570 mRNA fragments/genes, which showed no obvious changes, were omitted from this table.

Table S2. Changes in mRNA levels in *S. cerevisiae* treated with 50 μg/mL PCA or no PCA. Red-labeled genes are upregulated; green-labeled genes are downregulated;the other 9570 mRNA fragments/genes, which showed no obvious changes, were omitted from this table.

ProbeSetID	PCA01	PCA01.call	2	02.call	Ratio	Ratio.call	Transcript ID(Array Design)	Target Description	Representative Public ID	Alignments	Gene Title	Gene Symbol	Chromosomal Location	Ensembl	Entrez Gene	SwissProt	EC	RefSeq Protein ID	RefSeq Transcript ID	Gene Ontology Biological Process	Gene Ontology Cellular Component	Gene Ontology Molecular Function	InterPro	Trans Membrane	Annotation Description	Annotation Transcript Cluster
1771192_at	117.06	P	31.99	P	3.6593	P	YAL067W-A	S. cerevisiae	YAL067W-A	chr10:9451-	---	---	chr1	YAL067W-A	---	Q8TGK6	---	---	---	---	---	---	---	---	This probe s	S000028593(11),YAL067W-A(11)
1769997_at	639.16	P	33.24	P	19.2286	P	YAL067C	S. cerevisiae	YAL067C.S	chr1:7235-9	Putative per	SEO1	chr1	YAL067C	---	P39709	---	---	---	0006810 // t	0016020 // r	---	---	S00000006	This probe s	S000000062(11),GENSCAN00000003288(11),YAL067C(11)
1779679_s_at	1701.06	P	652.59	P	2.6066	P	YAL063C	S. cerevisiae	YAL063C.S	chr1:203507	Lectin-like p	FLO1 /// FL	chr1 /// chr8	YAL063C ///	---	A1E497 /// A	---	---	---	0000128 // f	0005886 // r	0005537 // r	---	S00000005	This probe s	S000000059(11),S000000084(11),S000001254(11),GENEFINDER00000000134(11),GENSCAN00000003322(11),GENEFINDER00000000147(11),GENSCAN00000003304(11),GENEFINDER00000000583(11),GENSCAN00000003886(11),YAL063C(11),YAR050W(11),YHR211W(11)
1772549_at	325.53	P	152.13	P	2.1398	P	YAL062W	S. cerevisiae	YAL062W.S	chr1:31567-	NADP(+)-de	GDH3	chr1	YAL062W	---	P39708	---	---	---	0006520 // a	0005625 // s	0003824 // c	---	---	This probe s	S000000058(11),GENEFINDER00000000104(11),GENSCAN00000003310(11),YAL062W(11)
1776156_at	3281.13	P	523.87	P	6.2633	P	YAL061W	S. cerevisiae	YAL061W.S	chr1:33448-	Putative me	BDH2	chr1	YAL061W	---	P39713	---	---	---	0008152 // r	0005634 // r	0003824 // c	---	---	This probe s	S000000057(11),GENEFINDER00000000131(11),GENSCAN00000003245(11),YAL061W(11)
1777350_at	9631.3	P	3137.04	P	3.0702	P	YAL060W	S. cerevisiae	YAL060W.S	chr1:35155-	NAD-depend	BDH1	chr1	YAL060W	---	P39714 /// C	---	---	---	0006066 // c	0005737 // c	0000721 // (	---	---	This probe s	S000000056(11),GENEFINDER00000000113(11),GENSCAN00000003317(11),YAL060W(11)
1772915_at	1076.1	P	406.54	P	2.647	P	YAL054C	S. cerevisiae	YAL054C.S	chr1:42880-	Acetyl-coA s	ACS1	chr1	YAL054C	---	Q01574 /// C	---	---	---	0006085 // a	0005739 // r	0003824 // c	---	---	This probe s	S000000050(11),GENEFINDER00000000103(11),GENSCAN00000003308(11),YAL054C(11)
1777171_at	2186.18	P	1066.86	P	2.0492	P	YAL044W-A	S. cerevisiae	YAL044W-A	chr1:57519-	---	---	chr1	YAL044W-A	---	Q3E793	---	---	---	---	---	---	---	---	This probe s	S000007586(11),YAL044W-A(11)
1769551_at	4773.32	P	1213.44	P	3.9337	P	YAL034C	S. cerevisiae	YAL034C.S	chr1:80711-	Non-essenti	FUN19	chr1	YAL034C	---	P28003	---	---	---	---	---	---	---	---	This probe s	S000002134(11),GENEFINDER00000000106(11),GENSCAN00000003259(11),YAL034C(11)
1772848_at	2055.77	P	659.99	P	3.1149	P	YAL028W	S. cerevisiae	YAL028W.S	chr1:92901-	Tail-anchore	FRT2	chr1	YAL028W	---	P39734	---	---	---	0006950 // r	0005783 // e	0005515 // r	---	S00000002	This probe s	S000000026(11),GENEFINDER00000000099(11),GENSCAN00000003311(11),YAL028W(11)
1775227_at	2196.21	P	965.71	P	2.2742	P	YAL008W	S. cerevisiae	YAL008W.S	chr1:136915	Mitochondri	FUN14	chr1	YAL008W	---	P18411	---	---	---	---	0005739 // r	0005515 // r	---	S00000000	This probe s	S000000006(11),YAL008W(11)
1772576_x_at	672.67	P	150.71	P	4.4633	P	YAR020C	S. cerevisiae	YAR020C.S	chr1:176853	Part of 23-m	PAU7	chr1	YAR020C	---	P39545	---	---	---	0006950 // r	0016020 // r	---	IPR000992	S00000007	This probe s	S000000073(11),YAR020C(11)
1779844_at	443.94	P	200.71	P	2.2118	P	YAR023C	S. cerevisiae	YAR023C.S	chr1:179278	---	---	chr1	YAR023C	---	P39546	---	---	---	---	0016020 // r	---	---	S00000007	This probe s	S000000074(11),YAR023C(11)
1778830_at	380.31	P	147.71	P	2.5747	P	YAR050W	S. cerevisiae	YAR050W.S	chr1:203393	Lectin-like p	FLO1	chr1	YAR050W	---	A1E497 /// A	---	---	---	0000128 // f	0005886 // r	0005537 // r	---	S00000008	This probe s	S000000084(11),GENEFINDER00000000147(11),GENSCAN00000003304(11),YAR050W(11)
1774060_at	1990.07	P	712.36	P	2.7936	P	YBL091C-A	S. cerevisiae	YBL091C-A	chr2:46558-	Protein invol	SCS22	chr2	YBL091C-A	---	Q6Q595	---	---	---	0006348 // c	0016020 // r	0005198 // s	---	S00000722	This probe s	S000007228(11),GENEFINDER00000001402(10),YBL091C-A(11)
1778374_at	914.37	P	392.8	P	2.3278	P	YBL086C	S. cerevisiae	YBL086C.S	chr2:61195-	---	---	chr2	YBL086C	---	P38177 /// C	---	---	---	---	---	---	---	---	This probe s	S000000182(11),GENEFINDER00000001510(11),GENSCAN00000005037(11),YBL086C(11)
1772778_at	6592.66	P	2525.71	P	2.6102	P	YBL078C	S. cerevisiae	YBL078C.S	chr2:80371-	Component	ATG8	chr2	YBL078C	---	P38182	---	---	---	0000045 // a	0000407 // r	0005515 // r	---	---	This probe s	S000000174(11),GENSCAN00000005468(11),YBL078C(11)
1779158_at	1797.74	P	890.76	P	2.0182	P	YBL075C	S. cerevisiae	YBL075C.S	chr2:84493-	ATPase invo	SSA3	chr2	YBL075C	---	P09435	---	---	---	0006457 // r	0005737 // c	0000166 // r	---	---	This probe s	S000000171(11),GENEFINDER00000001442(11),GENSCAN00000004881(11),YBL075C(11)
1775303_at	8358.76	P	2954.79	P	2.8289	P	YBL064C	S. cerevisiae	YBL064C.S	chr2:100331	Mitochondri	PRX1	chr2	YBL064C	---	P34227	---	---	---	0006979 // r	0005739 // r	0004601 // r	---	---	This probe s	S000000160(11),GENEFINDER00000001393(11),GENSCAN00000005474(11),YBL064C(11)
1771375_at	2463.95	P	75.69	P	32.5532	P	YBL049W	S. cerevisiae	YBL049W.S	chr2:126789	Protein of u	MOH1	chr2	YBL049W	---	P38191	---	---	---	0034605 // c	---	---	---	---	This probe s	S000000145(11),YBL049W(11)
1776434_at	3870.8	P	300.59	P	12.8773	P	YBL043W	S. cerevisiae	YBL043W.S	chr2:136649	Non-essenti	ECM13	chr2	YBL043W	---	P38195	---	---	---	0007047 // c	---	0005515 // r	---	---	This probe s	S000000139(11),GENEFINDER00000001458(11),GENSCAN00000004907(11),YBL043W(11)
1776351_at	3002.25	P	1256.98	P	2.3885	P	YBL029C-A	S. cerevisiae	YBL029C-A	chr2:164449	---	---	chr2	YBL029C-A	---	Q3E756	---	---	---	---	0005886 // r	0005515 // r	---	---	This probe s	S000007591(11),YBL029C-A(11)
1777284_at	1287.41	P	623	P	2.0665	P	YBL029W	S. cerevisiae	YBL029W.S	chr2:166095	---	---	chr2	YBL029W	---	P38201	---	---	---	---	0005634 // r	---	---	---	This probe s	S000000125(11),GENSCAN00000005163(11),YBL029W(11)
1776046_at	2572.71	P	1148.74	P	2.2396	P	YBR001C	S. cerevisiae	YBR001C.S	chr2:238901	Putative neu	NTH2	chr2	YBR001C	---	P35172	---	---	---	0005991 // t	0005737 // c	0003824 // c	---	---	This probe s	S000000205(11),GENEFINDER00000001455(11),GENSCAN00000004941(11),YBR001C(11)
1775744_at	3861.74	P	923.75	P	4.1805	P	YBR006W	S. cerevisiae	YBR006W.S	chr2:246970	Succinate se	UGA2	chr2	YBR006W	---	O42833 /// P	---	---	---	0006540 // c	0005737 // c	0005515 // r	---	---	This probe s	S000000210(11),GENEFINDER00000001342(11),GENSCAN00000005139(11),YBR006W(11)
1780188_at	1449.4	P	707.34	P	2.0491	P	YBR008C	S. cerevisiae	YBR008C.S	chr2:252522	Plasma men	FLR1	chr2	YBR008C	---	P38124	---	---	---	0006810 // t	0005886 // r	0015238 // c	---	S00000021	This probe s	S000000212(11),GENSCAN00000005418(11),YBR008C(11)
1776949_at	1456.65	P	450.29	P	3.2349	P	YBR013C	S. cerevisiae	YBR013C.S	chr2:265448	---	---	chr2	YBR013C	---	P38215	---	---	---	---	---	---	---	---	This probe s	S000000217(11),YBR013C(11)
1775628_at	347.07	P	73.84	P	4.7003	P	YBR018C	S. cerevisiae	YBR018C.S	chr2:274385	Galactose-1	GAL7	chr2	YBR018C	---	P08431	---	---	---	0005975 // c	0005737 // c	0003824 // c	---	---	This probe s	S000000222(11),GENEFINDER00000001457(11),GENSCAN00000005272(11),YBR018C(11)
1779098_at	556.58	P	211.08	P	2.6368	P	YBR020W	S. cerevisiae	YBR020W.S	chr2:278979	Galactokina	GAL1	chr2	YBR020W	---	P04385	---	---	---	0000411 // r	0005737 // c	0000166 // r	---	---	This probe s	S000000224(11),GENEFINDER00000001443(11),GENSCAN00000005503(11),YBR020W(11)
1772852_at	267.84	P	87.9	P	3.0471	P	YBR033W	S. cerevisiae	YBR033W.S	chr2:301902	Putative zinc	EDS1	chr2	YBR033W	---	P38073	---	---	---	0006350 // t	0005634 // r	0003677 // I	---	---	This probe s	S000000237(11),GENEFINDER00000001444(11),GENSCAN00000005368(11),YBR033W(11)
1773332_at	2246.6	P	1038.41	P	2.1635	P	YBR037C	S. cerevisiae	YBR037C.S	chr2:310522	Copper-bind	SCO1	chr2	YBR037C	---	P23833	---	---	---	0006461 // r	0005739 // r	0005507 // c	---	---	This probe s	S000000241(11),GENSCAN00000005252(11),YBR037C(11)
1777808_at	206.04	P	78.18	P	2.6355	P	YBR045C	S. cerevisiae	YBR045C.S	chr2:328329	Meiosis-spe	GIP1	chr2	YBR045C	---	P38229	---	---	---	0030476 // a	0000164 // r	0005515 // r	---	---	This probe s	S000000249(11),GENSCAN00000005243(11),YBR045C(11)
1777707_at	1632.99	P	538.21	P	3.0341	P	YBR047W	S. cerevisiae	YBR047W.S	chr2:331791	Putative pro	FMP23	chr2	YBR047W	---	P38231	---	---	---	---	0005739 // r	---	---	---	This probe s	S000000251(11),GENSCAN00000005473(11),YBR047W(11)
1770656_at	5223.15	P	2383.94	P	2.191	P	YBR053C	S. cerevisiae	YBR053C.S	chr2:339633	---	---	chr2	YBR053C	---	P38235	---	---	---	---	---	0005515 // r	---	---	This probe s	S000000257(11),GENSCAN00000004984(11),YBR053C(11)
1778053_at	15014.78	P	7495.05	P	2.0033	P	YBR054W	S. cerevisiae	YBR054W.S	chr2:343059	Putative pro	YRO2	chr2	YBR054W	---	P38079	---	---	---	0006811 // i	0005739 // r	0005216 // i	IPR001425	S00000025	This probe s	S000000258(11),GENEFINDER00000001451(11),GENSCAN00000005021(11),YBR054W(11)
1777569_at	16101.39	P	4402.23	P	3.6576	P	YBR072W	S. cerevisiae	YBR072W.S	chr2:381988	Small heat s	HSP26	chr2	YBR072W	---	P15992	---	---	---	0006457 // r	0005634 // r	0005515 // r	---	---	This probe s	S000000276(11),GENEFINDER00000001375(11),GENSCAN00000005035(11),YBR072W(11)
1769982_at	611.2	P	180.8	P	3.3805	P	YBR076W	S. cerevisiae	YBR076W.S	chr2:390330	Non-essenti	ECM8	chr2	YBR076W	---	P38246	---	---	---	0007047 // c	---	---	---	---	This probe s	S000000280(11),GENSCAN00000005296(11),YBR076W(11)
1771977_at	8195.72	P	2096.98	P	3.9083	P	YBR085C-A	S. cerevisiae	YBR085C-A	chr2:418862	---	---	chr2	YBR085C-A	---	O43137	---	---	---	---	0005634 // r	---	---	---	This probe s	S000007522(11),YBR085C-A(11)
1779016_at	522.39	P	220.57	P	2.3684	P	YBR098W	S. cerevisiae	YBR098W.S	chr2:441470	Subunit of th	MMS4	chr2	YBR098W	---	P38257	---	---	---	0006259 // I	0005634 // r	0000287 // r	---	---	This probe s	S000000302(11),GENEFINDER00000001407(11),GENSCAN00000005390(11),YBR098W(11)
1773681_at	6873.73	P	2086.54	P	3.2943	P	YBR105C	S. cerevisiae	YBR105C.S	chr2:450836	Peripheral m	VID24	chr2	YBR105C	---	P38263	---	---	---	0006623 // r	0016020 // r	0005515 // r	---	---	This probe s	S000000309(11),GENEFINDER00000001524(11),GENSCAN00000005029(11),YBR105C(11)
1775590_at	881.03	P	431.86	P	2.0401	P	YBR107C	S. cerevisiae	YBR107C.S	chr2:453748	Protein with	IML3	chr2	YBR107C	---	P38265	---	---	---	0007049 // c	0000777 // c	---	---	---	This probe s	S000000311(11),GENSCAN00000005231(11),YBR107C(11)
1770379_at	674.06	P	316.61	P	2.129	P	YBR108W	S. cerevisiae	YBR108W.S	chr2:454777	Protein inter	AIM3	chr2	YBR108W	---	P38266	---	---	---	---	0045121 // r	0005515 // r	---	---	This probe s	S000000312(10),GENEFINDER00000001411(10),GENSCAN00000005005(10),YBR108W(10)
1771955_at	10408	P	4122.74	P	2.5245	P	YBR111C	S. cerevisiae	YBR111C.S	chr2:461132	Nudix hydrol	YSA1	chr2	YBR111C	---	Q01976	---	---	---	0019693 // r	0005634 // r	0000287 // r	---	---	This probe s	S000000315(11),GENSCAN00000005358(11),YBR111C(11)
1778689_at	1259.72	P	618.02	P	2.0383	P	YBR111W-A	S. cerevisiae	YBR111W-A	chr2:462093	Protein invol	SUS1	chr2	YBR111W-A	---	Q6WNK7	---	NP_878049	---	0006350 // t	0000124 // S	0005515 // r	---	---	This probe s	AY278445(11)
1773804_at	1698.88	P	837.21	P	2.0292	P	YBR114W	S. cerevisiae	YBR114W.S	chr2:467202	Protein that	RAD16	chr2	YBR114W	---	P31244	---	---	---	0000715 // r	0000108 // r	0000166 // r	---	---	This probe s	S000000318(11),GENEFINDER00000001530(11),GENSCAN00000005109(11),YBR114W(11)
1778142_at	2101.65	P	139.02	P	15.1176	P	YBR117C	S. cerevisiae	YBR117C.S	chr2:474346	Transketolas	TKL2	chr2	YBR117C	---	P33315	---	---	---	0006098 // r	0005634 // r	0003824 // c	---	---	This probe s	S000000321(11),GENEFINDER00000001513(11),GENSCAN00000005354(11),YBR117C(11)
1769900_at	507.39	P	205.55	P	2.4685	P	YBR128C	S. cerevisiae	YBR128C.S	chr2:493035	Autophagy-s	ATG14	chr2	YBR128C	---	P38270	---	---	---	0006810 // t	0000300 // r	0005515 // r	---	---	This probe s	S000000332(11),GENSCAN00000005103(11),YBR128C(11)
1773348_at	2788.79	P	1213.45	P	2.2982	P	YBR137W	S. cerevisiae	YBR137W.S	chr2:512998	---	---	chr2	YBR137W	---	P38276	---	---	---	0034605 // c	0005737 // c	0005515 // r	---	---	This probe s	S000000341(11),GENSCAN00000005391(11),YBR137W(11)
1779364_at	2741.92	P	848.19	P	3.2327	P	YBR147W	S. cerevisiae	YBR147W.S	chr2:536529	Protein of ur	RTC2	chr2	YBR147W	---	P38279	---	---	---	---	0005739 // r	---	---	S00000035	This probe s	S000000351(11),YBR147W(11)
1777222_at	147.81	P	59.89	P	2.468	P	YBR148W	S. cerevisiae	YBR148W.S	chr2:537830	Protein expr	YSW1	chr2	YBR148W	---	P38280	---	---	---	---	---	---	---	---	This probe s	S000000352(11),GENSCAN00000005000(11),YBR148W(11)
1769411_at	7906.34	P	3761.37	P	2.102	P	YBR149W	S. cerevisiae	YBR149W.S	chr2:539941	NADP+ depe	ARA1	chr2	YBR149W	---	P38115	---	---	---	0005975 // c	0005737 // c	0016491 // c	---	---	This probe s	S000000353(11),GENEFINDER00000001371(11),GENSCAN00000005491(11),YBR149W(11)
1771395_at	5650.92	P	2365.22	P	2.3892	P	YBR151W	S. cerevisiae	YBR151W.S	chr2:544982	Protein of ur	APD1	chr2	YBR151W	---	P38281	---	---	---	---	0005634 // r	0005515 // r	---	---	This probe s	S000000355(11),GENEFINDER00000001543(10),GENSCAN00000005132(11),YBR151W(11)
1769643_at	8014.88	P	2087.28	P	3.8399	P	YBR169C	S. cerevisiae	YBR169C.S	chr2:573869	Member of t	SSE2	chr2	YBR169C	---	P32590	---	NP_009728	---	0006950 // r	0005737 // c	0000166 // r	---	---	This probe s	D38369(11)
1779307_at	6630.3	P	2863.57	P	2.3154	P	YBR177C	S. cerevisiae	YBR177C.S	chr2:584761	Acyl-coenzy	EHT1	chr2	YBR177C	---	P38295 /// C	---	---	---	0006629 // I	0005739 // r	0004026 // a	---	---	This probe s	S000000381(11),GENEFINDER00000001485(11),GENSCAN00000005079(11),YBR177C(11)
1779277_at	372.58	P	131.05	P	2.843	P	YBR182C	S. cerevisiae	YBR182C.S	chr2:593460	Putative tran	SMP1	chr2	YBR182C	---	P38128	---	---	---	0006350 // t	0005634 // r	0003677 // I	---	---	This probe s	S000000386(11),GENEFINDER00000001429(10),GENSCAN00000005499(11),YBR182C(11)
1773097_at	6547.65	P	1975.01	P	3.3152	P	YBR183W	S. cerevisiae	YBR183W.S	chr2:596069	Alkaline cera	YPC1	chr2	YBR183W	---	P38298	---	---	---	0006672 // c	0000139 // G	0005515 // r	---	S00000038	This probe s	S000000387(11),GENSCAN00000004998(11),YBR183W(11)
1773976_at	920.43	P	354.34	P	2.5976	P	YBR203W	S. cerevisiae	YBR203W.S	chr2:629122	Protein regu	COS111	chr2	YBR203W	---	P38308	---	---	---	0007165 // s	0005739 // r	0005515 // r	IPR001810	---	This probe s	S000000407(11),GENSCAN00000005238(11),YBR203W(11)
1778857_at	1361.08	P	388.86	P	3.5002	P	YBR208C	S. cerevisiae	YBR208C.S	chr2:636657	Urea amidol	DUR1,2	chr2	YBR208C	---	P32528	---	---	---	0000256 // a	0005737 // c	0000166 // r	IPR003135	---	This probe s	S000000412(11),GENEFINDER00000001477(11),GENSCAN00000005217(11),YBR208C(11)
1772959_at	648.79	P	300.76	P	2.1572	P	YBR213W	S. cerevisiae	YBR213W.S	chr2:650322	Bifunctional	MET8	chr2	YBR213W	---	A0SXK9 ///	---	---	---	0000103 // s	---	0003824 // c	---	---	This probe s	S000000417(11),GENEFINDER00000001401(11),GENSCAN00000005260(11),YBR213W(11)
1774926_at	6597.78	P	2128.37	P	3.0999	P	YBR214W	S. cerevisiae	YBR214W.S	chr2:651369	One of two	SDS24	chr2	YBR214W	---	P38314	---	---	---	0000920 // c	0005737 // c	0005515 // r	---	---	This probe s	S000000418(11),GENEFINDER00000001516(11),GENSCAN00000004995(11),YBR214W(11)
1772945_at	1816.01	P	691.06	P	2.6279	P	YBR230W-A	S. cerevisiae	YBR230W-A	chr2:680316	---	---	chr2	YBR230W-A	---	Q3E762	---	---	---	---	---	---	---	---	This probe s	S000029722(11),YBR230W-A(11)
1770288_at	450.11	P	121.44	P	3.7064	P	YBR240C	S. cerevisiae	YBR240C.S	chr2:700444	Zinc finger n	THI2	chr2	YBR240C	---	P38141	---	---	---	0006350 // t	0005634 // r	0003677 // I	---	---	This probe s	S000000444(11),GENEFINDER00000001501(11),GENSCAN00000005290(11),YBR240C(11)
1774729_at	3519.23	P	623.02	P	5.6487	P	YBR241C	S. cerevisiae	YBR241C.S	chr2:702543	Putative trans	---	chr2	YBR241C	---	P38142 /// C	---	---	---	0006810 // t	0000329 // f	0005215 // t	---	S00000044	This probe s	S000000445(11),GENEFINDER00000001351(11),GENSCAN00000005113(11),YBR241C(11)
1773816_at	7349.27	P	2031.89	P	3.617	P	YBR244W	S. cerevisiae	YBR244W.S	chr2:707482	Phospholipid	GPX2	chr2	YBR244W	---	P38143	---	---	---	0006979 // r	0005634 // r	0004601 // r	IPR013740	---	This probe s	S000000448(11),YBR244W(11)
1773522_at	199.9	P	74.82	P	2.6717	P	YBR250W	S. cerevisiae	YBR250W.S	chr2:718987	Protein of ur	SPO23	chr2	YBR250W	---	P33757	---	---	---	0007126 // r	---	---	---	---	This probe s	S000000454(11),GENSCAN00000005153(11),GENEFINDER00000001517(11),YBR250W(11)
1777486_at	5901.49	P	2722.67	P	2.1675	P	YBR256C	S. cerevisiae	YBR256C.S	chr2:727340	Riboflavin sy	RIB5	chr2	YBR256C	---	P38145	---	---	---	0009231 // r	0005625 // s	0004746 // r	---	---	This probe s	S000000460(11),GENSCAN00000005032(11),YBR256C(11)
1772934_at	5966.82	P	2349.37	P	2.5398	P	YBR269C	S. cerevisiae	YBR269C.S	chr2:742114	Putative pro	FMP21	chr2	YBR269C	---	P38345	---	---	---	---	0005739 // r	---	---	---	This probe s	S000000473(11),GENSCAN00000004921(11),YBR269C(11)
1770427_at	772.22	P	373.37	P	2.0682	P	YBR270C	S. cerevisiae	YBR270C.S	chr2:742713	Subunit of T	BIT2	chr2	YBR270C	---	P38346	---	---	---	---	---	0005515 // r	---	---	This probe s	S000000474(11),GENSCAN00000005241(11),YBR270C(11)
1776425_at	1631.16	P	438.89	P	3.7166	P	YBR280C	S. cerevisiae	YBR280C.S	chr2:762737	F-Box protei	SAF1	chr2	YBR280C	---	P38352	---	---	---	0019941 // r	0019005 // S	0004842 // u	---	---	This probe s	S000000484(11),GENEFINDER00000001459(11),GENSCAN00000005043(11),YBR280C(11)
1778076_at	632.94	P	125.78	P	5.0321	P	YBR284W	S. cerevisiae	YBR284W.S	chr2:771192	---	---	chr2	YBR284W	---	P38150	---	---	---	0006144 // r	0016020 // r	0005515 // r	---	GENEFINDE	This probe s	S000000488(11),GENEFINDER00000001497(9),GENSCAN00000005443(11),YBR284W(11)
1769585_at	585.38	P	222.9	P	2.6262	P	YBR285W	S. cerevisiae	YBR285W.S	chr2:773875	---	---	chr2	YBR285W	---	P38354 /// C	---	---	---	---	---	---	---	GENEFINDE	This probe s	S000000489(11),GENEFINDER00000001497(9),YBR285W(11)
1770303_at	731.23	P	19.47	A	37.5568	P	YBR294W	S. cerevisiae	YBR294W.S	chr2:789187	High affinity	SUL1	chr2	YBR294W	---	P38359	---	NP_009853	---	0006810 // t	0005886 // r	0005215 // t	---	S00000049	This probe s	X82013(11)
1773594_at	2160.1	P	797.67	P	2.708	P	YBR298C	S. cerevisiae	YBR298C.S	chr2:802582	Maltose per	MAL31	chr2	YBR298C	---	O93979 /// F	---	---	---	0000023 // r	0016020 // r	0005215 // t	---	S00000050	This probe s	S000000502(11),GENEFINDER00000001418(11),GENSCAN00000005111(11),YBR298C(11)
1770162_x_at	335.49	P	122.6	P	2.7365	P	YBR301W	S. cerevisiae	YBR301W.S	chr4:152322	Cell wall ma	DAN3	chr2	YBR301W	---	P38155 /// C	---	---	---	0006950 // r	0005576 // e	---	---	---	This probe s	S000000505(11),GENEFINDER00000001395(11),GENSCAN00000005376(11),YBR301W(11)
1775991_s_at	204.49	P	89.81	P	2.2769	P	YCL073C	S. cerevisiae	YCL073C.S	chr11:66107	---	---	chr11 /// chr	YCL073C ///	---	P25596 /// F	---	---	---	---	0016020 // r	---	---	S00000057	This probe s	S000000575(11),S000001814(11),GENSCAN00000003736(11),GENSCAN00000004534(11),GENEFINDER00000001790(11),YCL073C(11),YKR106W(11)
1776457_at	3398.42	P	1657	P	2.0509	P	YCL052C	S. cerevisiae	YCL052C.S	chr3:34142-	Essential co	PBN1	chr3	YCL052C	---	P25580	---	---	---	0006506 // C	0005783 // e	0000030 // r	---	S00000055	This probe s	S000000557(11),GENSCAN00000003610(11),YCL052C(11)
1773907_at	4517.05	P	1698.14	P	2.66	P	YCL049C	S. cerevisiae	YCL049C.S	chr3:39785-	---	---	chr3	YCL049C	---	P25577	---	---	---	---	0005624 // r	---	---	---	This probe s	S000000554(11),GENEFINDER00000000048(11),GENSCAN00000003708(11),YCL049C(11)
1772421_at	8603.36	P	3923.45	P	2.1928	P	YCL035C	S. cerevisiae	YCL035C.S	chr3:60840-	Hydroperoxi	GRX1	chr3	YCL035C	---	P25373	---	---	---	0006810 // t	0005634 // r	0004364 // c	---	---	This probe s	S000000540(11),GENSCAN00000003629(11),YCL035C(11)
1777941_at	2051.9	P	958.53	P	2.1407	P	YCL033C	S. cerevisiae	YCL033C.S	chr3:62775-	Methionine-F	MXR2	chr3	YCL033C	---	P25566	---	---	---	0006979 // r	---	0008113 // r	---	---	This probe s	S000000538(11),GENEFINDER00000000028(11),YCL033C(11)
1776829_at	1481.07	P	505.02	P	2.9327	P	YCL026C-B	S. cerevisiae	YCL026C-B	chr3:73404-	Putative pro	HBN1	chr3	YCL026C-B	---	Q96VH4	---	---	---	0055114 // c	0005634 // r	0016491 // c	---	---	This probe s	S000007548(11),GENEFINDER00000000056(11),GENSCAN00000003699(11),YCL026C-B(11)
1778141_at	8293.89	P	2188.29	P	3.7901	P	YCL025C	S. cerevisiae	YCL025C.S	chr3:76130-	Low-affinity	AGP1	chr3	YCL025C	---	P25376	---	---	---	0006810 // t	0005886 // r	0005515 // r	---	S00000053	This probe s	S000000530(11),GENEFINDER00000000005(11),GENSCAN00000003703(11),YCL025C(11)
1770347_at	610.25	P	205.71	P	2.9666	P	YCR007C	S. cerevisiae	YCR007C.S	chr3:126004	---	---	chr3	YCR007C	---	P25354	---	---	---	---	0016020 // r	---	---	S00000060	This probe s	S000000600(11),GENSCAN00000003643(11),YCR007C(11)
1774805_at	1156.47	P	335.43	P	3.4477	P	YCR020C	S. cerevisiae	YCR020C.S	chr3:154005	Protein regu	PET18	chr3	YCR020C	---	P25362	---	---	---	0006772 // t	0005829 // c	---	---	---	This probe s	S000000613(11),YCR020C(11)
1774184_at	3075.1	P	951.74	P	3.231	P	YCR061W	S. cerevisiae	YCR061W.S	chr3:225554	---	---	chr3	YCR061W	---	P25639	---	---	---	0008361 // r	0005737 // c	---	---	S00000065	This probe s	S000000657(11),GENSCAN00000003670(11),GENEFINDER00000000058(11),YCR061W(11)
1770695_at	3530.8	P	1630.33	P	2.1657	P	YCR083W	S. cerevisiae	YCR083W.S	chr3:259570	Mitochondria	TRX3	chr3	YCR083W	---	P25372	---	---	---	0006810 // t	0005739 // r	0015036 // c	---	---	This probe s	S000000679(11),GENSCAN00000003691(10),YCR083W(11)
1770900_at	988.94	P	373.29	P	2.6493	P	YCR091W	S. cerevisiae	YCR091W.S	chr3:274396	Putative seri	KIN82	chr3	YCR091W	---	P25341	---	---	---	0006468 // r	---	0000166 // r	IPR001245	---	This probe s	S000000687(11),GENSCAN00000003681(11),YCR091W(11)
1770353_at	153.12	P	75.44	P	2.0297	P	YCR098C	S. cerevisiae	YCR098C.S	chr3:297041	Plasma men	GIT1	chr3	YCR098C	---	P25346	---	---	---	0001407 // c	0005886 // r	0001406 // c	---	S00000069	This probe s	S000000695(11),GENEFINDER00000000012(11),GENSCAN00000003607(11),YCR098C(11)
1772983_at	595.02	P	141.64	P	4.2009	P	YCR099C	S. cerevisiae	YCR099C.S	chr3:300824	---	---	chr3	YCR099C	---	P25657	---	---	---	---	---	---	---	---	This probe s	S000000696(11),YCR099C(11)
1776145_at	732.78	P	161.21	P	4.5455	P	YCR100C	S. cerevisiae	YCR100C.S	chr3:301263	---	---	chr3	YCR100C	---	P25606	---	---	---	---	---	---	---	---	This probe s	S000000697(11),GENSCAN00000003667(11),YCR100C(11)
1772198_at	411.32	P	157.91	P	2.6048	P	YCR102C	S. cerevisiae	YCR102C.S	chr3:304353	---	---	chr3	YCR102C	---	P25608	---	---	---	0008152 // r	---	0003824 // c	IPR013154	---	This probe s	S000000699(11),GENEFINDER00000000052(11),GENSCAN00000003593(11),YCR102C(11)
1778121_s_at	791.16	P	172.79	P	4.5787	P	YCR104W	S. cerevisiae	YCR104W.S	chr9:433924	Putative pro	PAU15 /// P	chr11 /// chr	YCR104W /	---	P25610 /// F	---	---	---	0006950 // r	0016020 // r	---	---	S00000070	This probe s	S000000701(11),S000001480(11),S000001707(11),S000004944(11),S000005921(11),S000006203(11),GENEFINDER00000000004(11),GENSCAN00000003718(11),GENEFINDER00000000470(11),GENSCAN00000003413(11),GENEFINDER00000001652(11),GENSCAN00000004497(11),GENEFINDER00000001950(11),GENSCAN00000006
1773829_at	190.23	P	38.51	P	4.9398	P	YDL247W	S. cerevisiae	YDL247W.S	chr4:5984-7	Alpha-glucos	MPH2	chr4	YDL247W	---	P47186	---	---	---	0000017 // a	0016020 // r	0005215 // t	IPR011701	S00000240	This probe s	S000002406(11),GENEFINDER00000002737(11),GENSCAN00000008194(11),YDL247W(11)
1774944_s_at	404.72	P	115.72	P	3.4974	P	YDL247W	S. cerevisiae	YDL247W.S	chr4:5984-7	Alpha-glucos	MPH2 /// MF	chr10 /// chr	YDL247W //	---	P47186	---	---	---	0000017 // a	0016020 // r	0005215 // t	IPR011701	S00000240	This probe s	S000002406(11),S000003921(11),GENEFINDER00000001218(11),GENSCAN00000004387(11),GENEFINDER00000002737(11),GENSCAN00000008194(11),YJR160C(11),YDL247W(11)
1777143_s_at	604.82	P	297.94	P	2.03	P	YDL246C	S. cerevisiae	YDL246C.S	chr10:73573	Sorbitol deh	SOR1 /// SC	chr10 /// chr	YDL246C //	---	P35497 /// C	---	---	---	0008152 // r	---	0003824 // c	---	---	This probe s	S000002405(11),S000003920(11),GENEFINDER00000001262(11),GENSCAN00000004220(11),GENEFINDER00000002943(11),GENSCAN00000008292(11),YJR159W(11),YDL246C(11)
1769913_s_at	579.12	P	147.28	P	3.9321	P	YDL244W	S. cerevisiae	YDL244W.S	chr14:14831	Protein invol	THI11 /// TH	chr10 /// chr	YDL244W //	---	P42883 /// F	---	---	---	0009228 // t	---	0005515 // r	---	---	This probe s	S000002403(11),S000001836(11),S000003917(11),S000005276(11),GENEFINDER00000000164(11),GENSCAN00000003366(11),GENEFINDER00000001324(11),GENSCAN00000004397(11),GENEFINDER00000000674(11),GENSCAN00000005082(11),GENEFINDER00000002631(11),GENSCAN00000008066(11),YFL058W(11),YJR156C(1
1774511_at	657.34	P	154.71	P	4.2489	P	YDL243C	S. cerevisiae	YDL243C.S	chr4:17576-	Putative aryl	AAD4	chr4	YDL243C	---	Q07747	---	---	---	0006950 // r	---	0016491 // c	---	---	This probe s	S000002402(11),GENEFINDER00000002865(11),GENSCAN00000008136(11),YDL243C(11)
1771716_s_at	4441.61	P	1121.89	P	3.959	P	YDL243C	S. cerevisiae	YDL243C.S	chr4:17576-	Putative aryl	AAD16 /// A	chr4 /// chr6	YDL243C //	---	P43546 /// C	---	---	---	0006950 // r	---	0005515 // r	IPR001395	---	This probe s	S000002402(11),S000001837(11),GENEFINDER00000000170(11),GENSCAN00000003565(11),GENEFINDER00000002865(11),GENSCAN00000008136(11),YFL057C(11),YDL243C(11)
1774087_at	1020.82	P	275.31	P	3.7079	P	YDL239C	S. cerevisiae	YDL239C.S	chr4:26402-	Protein regu	ADY3	chr4	YDL239C	---	Q07732	---	---	---	0000001 // r	0005628 // r	0005515 // r	---	---	This probe s	S000002398(11),GENSCAN00000007733(11),YDL239C(11)
1777816_at	520.94	P	118.12	P	4.4103	P	YDL238C	S. cerevisiae	YDL238C.S	chr4:28984-	Guanine dea	GUD1	chr4	YDL238C	---	Q07729	---	---	---	---	0005737 // c	0005515 // r	---	---	This probe s	S000002397(11),GENEFINDER00000002965(11),GENSCAN00000007983(11),YDL238C(11)
1769986_at	620.49	P	102.13	P	6.0755	P	YDL223C	S. cerevisiae	YDL223C.S	chr4:57265-	Substrate of	HBT1	chr4	YDL223C	---	Q07653	---	---	---	0000746 // c	0005737 // c	0005515 // r	---	---	This probe s	S000002382(11),GENEFINDER00000002908(11),GENSCAN00000007739(11),YDL223C(11)
1771052_at	690.58	P	123.44	P	5.5945	P	YDL222C	S. cerevisiae	YDL222C.S	chr4:60872-	Integral mer	FMP45	chr4	YDL222C	---	Q07651	---	---	---	0007047 // c	0005739 // r	0005515 // r	---	S00000238	This probe s	S000002381(11),GENEFINDER00000002794(11),GENSCAN00000007817(10),YDL222C(11)
1770125_at	283.25	P	88.6	P	3.197	P	YDL218W	S. cerevisiae	YDL218W.S	chr4:66493-	---	---	chr4	YDL218W	---	Q07629	---	---	---	---	0005737 // c	0005515 // r	---	S00000237	This probe s	S000002377(11),GENSCAN00000008044(11),GENEFINDER00000002961(11),YDL218W(11)
1774699_at	128.59	P	41.3	P	3.1136	P	YDL214C	S. cerevisiae	YDL214C.S	chr4:74446-	Serine/threo	PRR2	chr4	YDL214C	---	Q12310	---	---	---	0006468 // r	---	0000166 // r	---	---	This probe s	S000002373(11),GENSCAN00000007956(11),YDL214C(11)
1778622_at	417.78	P	201.4	P	2.0744	P	YDL210W	S. cerevisiae	YDL210W.S	chr4:84270-	Permease th	UGA4	chr4	YDL210W	---	P32837 /// C	---	---	---	0006810 // t	0000329 // f	0015171 // a	---	S00000236	This probe s	S000002369(11),GENEFINDER00000002885(11),GENSCAN00000007661(11),YDL210W(11)
1775740_at	983.56	P	381.05	P	2.5812	P	YDL206W	S. cerevisiae	YDL206W.S	chr4:90176-	---	---	chr4	YDL206W	---	Q12424	---	---	---	0006810 // t	0016020 // r	---	---	S00000236	This probe s	S000002365(11),GENSCAN00000008139(11),YDL206W(11)
1779266_at	8397.4	P	892.82	P	9.4055	P	YDL204W	S. cerevisiae	YDL204W.S	chr4:94605-	Protein of ur	RTN2	chr4	YDL204W	---	Q12443	---	NP_010077	---	---	0005635 // r	0005515 // r	---	S00000236	This probe s	AY164797(11)
1770764_at	978.64	P	247.48	P	3.9544	P	YDL199C	S. cerevisiae	YDL199C.S	chr4:101290	---	---	chr4	YDL199C	---	Q12407	---	---	---	0006810 // t	0016020 // r	0005215 // t	IPR005828	S00000235	This probe s	S000002358(11),GENEFINDER00000002872(11),GENSCAN00000007818(11),YDL199C(11)
1779030_at	4188.11	P	1538.35	P	2.7225	P	YDL174C	S. cerevisiae	YDL174C.S	chr4:145826	D-lactate de	DLD1	chr4	YDL174C	---	P32891	---	---	---	0005975 // c	0005739 // r	0003824 // c	---	---	This probe s	S000002333(11),GENEFINDER00000002878(11),GENSCAN00000007865(11),YDL174C(11)
1773191_at	1478.37	P	614.62	P	2.4053	P	YDL170W	S. cerevisiae	YDL170W.S	chr4:156318	Transcriptio	UGA3	chr4	YDL170W	---	P26370 /// C	---	---	---	0006350 // t	0005634 // r	0003677 // I	---	---	This probe s	S000002329(11),GENSCAN00000008229(11),YDL170W(11)
1775340_at	1102.65	P	167.34	P	6.5893	P	YDL169C	S. cerevisiae	YDL169C.S	chr4:158064	Protein of ur	UGX2	chr4	YDL169C	---	P32772	---	---	---	---	---	0005515 // r	---	---	This probe s	S000002328(11),GENSCAN00000007781(11),YDL169C(11)
1774287_at	1844.23	P	850.87	P	2.1675	P	YDL142C	S. cerevisiae	YDL142C.S	chr4:201719	Cardiolipin s	CRD1	chr4	YDL142C	---	Q07560	---	---	---	0006873 // c	0005739 // r	0005515 // r	---	S00000230	This probe s	S000002301(11),YDL142C(11)
1773541_at	988.83	P	348.14	P	2.8403	P	YDL138W	S. cerevisiae	YDL138W.S	chr4:213351	Plasma men	RGT2	chr4	YDL138W	---	Q12300	---	---	---	0006810 // t	0005886 // r	0004872 // r	---	S00000229	This probe s	S000002297(11),GENSCAN00000007774(11),GENEFINDER00000002825(11),YDL138W(11)
1771782_at	5138.04	P	2253.89	P	2.2796	P	YDL130W-A	S. cerevisiae	YDL130W-A	chr4:229170	Protein invol	STF1	chr4	YDL130W-A	---	P01098	---	---	---	0032780 // r	0005739 // r	0004857 // e	---	---	This probe s	S000007232(11),YDL130W-A(11)
1776343_at	1310.84	P	615.11	P	2.1311	P	YDL113C	S. cerevisiae	YDL113C.S	chr4:256631	Sorting nexi	ATG20	chr4	YDL113C	---	Q07528	---	---	---	0006810 // t	0000300 // r	0005515 // r	---	---	This probe s	S000002271(11),GENEFINDER00000002646(11),GENSCAN00000008290(11),YDL113C(11)
1778304_at	1306.37	P	568.48	P	2.298	P	YDL091C	S. cerevisiae	YDL091C.S	chr4:293390	UBX (ubiquit	UBX3	chr4	YDL091C	---	Q12229	---	---	---	0019941 // r	0005737 // c	0005515 // r	---	S00000224	This probe s	S000002249(11),GENSCAN00000008160(11),YDL091C(11)
1769959_at	662.4	P	84.43	P	7.8456	P	YDL085W	S. cerevisiae	YDL085W.S	chr4:303209	Mitochondria	NDE2	chr4	YDL085W	---	Q07500	---	---	---	0006116 // N	0005739 // r	0005515 // r	---	---	This probe s	S000002243(11),GENEFINDER00000002827(11),GENSCAN00000008047(11),YDL085W(11)
1778227_at	852.03	P	343.95	P	2.4772	P	YDL079C	S. cerevisiae	YDL079C.S	chr4:312949	Glycogen sy	MRK1	chr4	YDL079C	---	P50873	---	---	---	0006468 // r	---	0000166 // r	IPR000534	---	This probe s	S000002237(11),GENEFINDER00000002823(11),GENSCAN00000007714(11),YDL079C(11)
1775757_at	2114.01	P	292.52	P	7.2269	P	YDL059C	S. cerevisiae	YDL059C.S	chr4:344235	Protein invol	RAD59	chr4	YDL059C	---	Q12223	---	---	---	0000722 // t	0005634 // r	0000739 // I	---	---	This probe s	S000002217(11),GENSCAN00000008174(11),YDL059C(11)
1770549_at	531.42	P	210.76	P	2.5214	P	YDL057W	S. cerevisiae	YDL057W.S	chr4:351432	---	---	chr4	YDL057W	---	Q07379	---	---	---	0034605 // c	---	---	---	---	This probe s	S000002215(11),GENSCAN00000007968(11),YDL057W(11)
1771404_at	3350.92	P	1531.61	P	2.1878	P	YDL025C	S. cerevisiae	YDL025C.S	chr4:405339	---	---	chr4	YDL025C	---	Q12100	---	---	---	0006468 // r	---	0000166 // r	IPR001245	---	This probe s	S000002183(11),GENSCAN00000007896(11),YDL025C(11)
1773875_at	898.02	P	94.02	P	9.5514	P	YDL024C	S. cerevisiae	YDL024C.S	chr4:408446	Protein of ur	DIA3	chr4	YDL024C	---	P52290	---	---	---	0001403 // i	0009277 // f	0003993 // a	---	---	This probe s	S000002182(11),GENSCAN00000007812(11),YDL024C(11)
1776528_at	2849.13	P	713.89	P	3.991	P	YDL021W	S. cerevisiae	YDL021W.S	chr4:413948	Homolog of	GPM2	chr4	YDL021W	---	Q12008	---	---	---	0006096 // c	0005737 // c	0003824 // c	---	---	This probe s	S000002179(11),GENSCAN00000008235(11),YDL021W(11)
1775378_at	5260	P	2480.58	P	2.1205	P	YDL020C	S. cerevisiae	YDL020C.S	chr4:415108	Transcriptio	RPN4	chr4	YDL020C	---	Q03465 /// C	---	---	---	0000086 // C	0005622 // i	0003677 // I	---	---	This probe s	S000002178(11),GENEFINDER00000002869(11),GENSCAN00000007748(11),YDL020C(11)
1770456_at	5099.13	P	2125	P	2.3996	P	YDR001C	S. cerevisiae	YDR001C.S	chr4:450215	Neutral treha	NTH1	chr4	YDR001C	---	P32356 /// C	---	---	---	0005991 // t	0005737 // c	0003824 // c	---	---	This probe s	S000002408(11),GENEFINDER00000002671(11),GENSCAN00000008067(11),YDR001C(11)
1778728_at	4242.39	P	1276.31	P	3.3239	P	YDR003W	S. cerevisiae	YDR003W.S	chr4:454117	Vacuolar pro	RCR2	chr4	YDR003W	---	Q03446	---	---	---	0016192 // v	0000324 // f	0005515 // r	---	S00000241	This probe s	S000002410(11),YDR003W(11)
1774645_at	4237.95	P	1838.49	P	2.3051	P	YDR003W-A	S. cerevisiae	YDR003W-A	chr4:454777	---	---	chr4	YDR003W-A	---	Q3E7Z7	---	---	---	---	---	---	---	---	This probe s	S000028819(11),YDR003W-A(11)
1776773_at	5570.96	P	1828.89	P	3.0461	P	YDR011W	S. cerevisiae	YDR011W.S	chr4:465914	Plasma men	SNQ2	chr4	YDR011W	---	P32568	---	---	---	0000304 // r	0005624 // r	0000166 // r	---	S00000241	This probe s	S000002418(11),GENEFINDER00000002728(11),GENSCAN00000008181(11),YDR011W(11)
1772647_at	2220.07	P	168.99	P	13.1373	P	YDR018C	S. cerevisiae	YDR018C.S	chr4:482664	Probable mer	---	chr4	YDR018C	---	Q12185	---	---	---	0008152 // r	0016020 // r	0008415 // a	---	S00000242	This probe s	S000002425(11),GENSCAN00000008013(11),YDR018C(11)
1778545_at	1351.23	P	513.71	P	2.6303	P	YDR022C	S. cerevisiae	YDR022C.S	chr4:488066	Autophagy-s	CIS1	chr4	YDR022C	---	Q12421	---	---	---	0000741 // k	0000407 // r	0005515 // r	---	---	This probe s	S000002429(11),GENEFINDER00000002754(11),GENSCAN00000007888(11),YDR022C(11)
1778149_at	4430.49	P	689.65	P	6.4243	P	YDR034W-B	S. cerevisiae	YDR034W-B	chr4:521307	---	---	chr4	YDR034W-B	---	Q6Q5X2	---	---	---	---	0005737 // c	0005515 // r	---	---	This probe s	S000007234(11),YDR034W-B(11)
1771435_at	4318.24	P	1266.26	P	3.4102	P	YDR043C	S. cerevisiae	YDR043C.S	chr4:542668	Transcriptio	NRG1	chr4	YDR043C	---	Q03125	---	---	---	0001403 // i	0005622 // i	0003676 // r	---	---	This probe s	S000002450(11),GENSCAN00000008082(11),YDR043C(11)
1770307_at	9693.67	P	3715.19	P	2.6092	P	YDR055W	S. cerevisiae	YDR055W.S	chr4:563521	Cell wall pro	PST1	chr4	YDR055W	---	Q12355	---	---	---	0001101 // r	0005576 // e	---	IPR000494	---	This probe s	S000002462(11),GENEFINDER00000002837(11),GENSCAN00000008126(11),YDR055W(11)

1777617_at	1174.76	P	507.64	P	2.3142	P	YDR058C	S. cerevisiae	YDR058C.S	chr4:567717	Protein with	TGL2	chr4	YDR058C	---	P54857 /// (	---	---	---	0016042 // l	---	0004806 // t	---	---	This probe s	S000002465(11),GENSCAN00000008306(11),YDR058C(11)
1779563_at	6981.65	P	766.93	P	9.1034	P	YDR070C	S. cerevisiae	YDR070C.S	chr4:588092	Putative pro	FMP16	chr4	YDR070C	---	Q12497	---	---	---	---	0005739 // r	---	---	---	This probe s	S000002477(11),YDR070C(11)
1775412_at	1168.84	P	520.9	P	2.2439	P	YDR078C	S. cerevisiae	YDR078C.S	chr4:602191	Protein invol	SHU2	chr4	YDR078C	---	P38957	---	---	---	0000725 // r	---	0005515 // r	---	---	This probe s	S000002485(11),YDR078C(11)
1778437_at	2307.9	P	547.79	P	4.2131	P	YDR085C	S. cerevisiae	YDR085C.S	chr4:614280	Protein regu	AFR1	chr4	YDR085C	---	P33304	---	---	---	0000750 // r	0001400 // r	0005057 // r	---	---	This probe s	S000002492(11),GENEFINDER00000002832(11),GENSCAN00000008182(11),YDR085C(11)
1772516_at	2966.36	P	1446.2	P	2.0511	P	YDR096W	S. cerevisiae	YDR096W.S	chr4:637131	JmjC domain	GIS1	chr4	YDR096W	---	Q03833	---	---	---	0006350 // t	0005622 // i	0003677 // [	---	---	This probe s	S000002503(11),GENSCAN00000008201(11),YDR096W(11)
1776178_at	1207.12	P	503.62	P	2.3969	P	YDR132C	S. cerevisiae	YDR132C.S	chr4:718808	---	---	chr4	YDR132C	---	Q03900	---	---	---	---	0005634 // r	0005515 // r	---	---	This probe s	S000002539(11),GENSCAN00000007804(11),YDR132C(11)
1776550_at	10623.51	P	4848.93	P	2.1909	P	YDR171W	S. cerevisiae	YDR171W.S	chr4:806613	Small heat s	HSP42	chr4	YDR171W	---	Q12329	---	---	---	0006950 // r	0005737 // c	0005515 // r	---	---	This probe s	S000002578(11),GENEFINDER00000002810(11),GENSCAN00000008080(11),YDR171W(11)
1774353_at	2061.9	P	1000.46	P	2.061	P	YDR185C	S. cerevisiae	YDR185C.S	chr4:831925	---	---	chr4	YDR185C	---	Q04006	---	---	---	---	0005739 // r	---	---	---	This probe s	S000002593(11),GENSCAN00000008014(11),YDR185C(11)
1772224_at	2430.68	P	1120.63	P	2.169	P	YDR202C	S. cerevisiae	YDR202C.S	chr4:856889	Subunit of R	RAV2	chr4	YDR202C	---	Q03956	---	---	---	0006810 // t	0005737 // c	0005515 // r	---	---	This probe s	S000002610(11),GENSCAN00000008041(11),YDR202C(11)
1776216_at	3508.29	P	1113.77	P	3.1499	P	YDR204W	S. cerevisiae	YDR204W.S	chr4:858128	Protein with	COQ4	chr4	YDR204W	---	O13525	---	---	---	0006744 // u	0005739 // r	---	---	---	This probe s	S000002612(11),GENEFINDER00000002951(11),GENSCAN00000008144(11),YDR204W(11)
1776183_at	917.93	P	192.59	P	4.7662	P	YDR216W	S. cerevisiae	YDR216W.S	chr4:895025	Carbon sour	ADR1	chr4	YDR216W	---	P07248	---	---	---	0000433 // r	0005622 // i	0003676 // r	---	---	This probe s	S000002624(11),GENEFINDER00000002899(11),GENSCAN00000007953(11),YDR216W(11)
1769854_at	302.74	P	41.33	P	7.3249	P	YDR223W	S. cerevisiae	YDR223W.S	chr4:912089	Transcriptio	CRF1	chr4	YDR223W	---	Q04930	---	---	---	0006350 // t	0005634 // r	0003714 // t	---	---	This probe s	S000002631(11),GENEFINDER00000002697(11),GENSCAN00000007807(11),YDR223W(11)
1779950_at	1212.88	P	428	P	2.8338	P	YDR242W	S. cerevisiae	YDR242W.S	chr4:946797	Putative ami	AMD2	chr4	YDR242W	---	P22580	---	---	---	---	---	0004040 // a	---	---	This probe s	S000002650(11),GENSCAN00000008153(11),YDR242W(11)
1772055_at	3507.21	P	1483.96	P	2.3634	P	YDR247W	S. cerevisiae	YDR247W.S	chr4:956003	Cytoplasmic	VHS1	chr4	YDR247W	---	Q03785	---	---	---	0000082 // (	0005737 // c	0000166 // r	---	---	This probe s	S000002655(11),GENSCAN00000008274(11),YDR247W(11)
1769750_at	2176.45	P	130.04	P	16.7368	P	YDR253C	S. cerevisiae	YDR253C.S	chr4:963980	Zinc-finger D	MET32	chr4	YDR253C	---	Q12041	---	---	---	0000096 // s	0005622 // i	0003677 // [	---	---	This probe s	S000002661(11),GENSCAN00000008254(11),YDR253C(11)
1776304_at	607.57	P	69.87	P	8.6957	P	YDR256C	S. cerevisiae	YDR256C.S	chr4:968123	Catalase A,	CTA1	chr4	YDR256C	---	P15202 /// C	---	---	---	0000302 // r	0005759 // r	0004096 // c	---	---	This probe s	S000002664(11),GENEFINDER00000002900(11),GENSCAN00000008027(11),YDR256C(11)
1778899_at	8409.78	P	2579.29	P	3.2605	P	YDR258C	S. cerevisiae	YDR258C.S	chr4:971798	Oligomeric r	HSP78	chr4	YDR258C	---	P33416	---	---	---	0000002 // r	0005739 // r	0000166 // r	---	---	This probe s	S000002666(11),GENEFINDER00000002842(11),GENSCAN00000008150(11),YDR258C(11)
1773511_at	832.93	P	254.25	P	3.276	P	YDR259C	S. cerevisiae	YDR259C.S	chr4:974621	Putative bas	YAP6	chr4	YDR259C	---	Q03935	---	---	---	0006350 // t	0005634 // r	0003677 // [	---	---	This probe s	S000002667(11),GENSCAN00000008049(11),YDR259C(11)
1771632_at	829.95	P	329.59	P	2.5181	P	YDR273W	S. cerevisiae	YDR273W.S	chr4:101016	Meiosis-spe	DON1	chr4	YDR273W	---	Q05610	---	---	---	0007049 // c	0005628 // r	0005515 // r	---	---	This probe s	S000002681(11),GENEFINDER00000002752(11),GENSCAN00000007870(11),YDR273W(11)
1773597_at	1745.91	P	716.02	P	2.4384	P	YDR313C	S. cerevisiae	YDR313C.S	chr4:108921	RING-type u	PIB1	chr4	YDR313C	---	Q06651	---	---	---	0016567 // r	0000329 // f	0004842 // u	IPR000306 ,	---	This probe s	S000002721(11),GENEFINDER00000002916(10),GENSCAN00000007676(11),YDR313C(11)
1776161_at	1914.51	P	740.07	P	2.5869	P	YDR358W	S. cerevisiae	YDR358W.S	chr4:119004	Golgi-localiz	GGA1	chr4	YDR358W	---	Q06336	---	---	---	0006810 // t	0005622 // i	0005515 // r	IPR004152 ,	---	This probe s	S000002766(11),GENSCAN00000007898(11),YDR358W(11)
1771990_at	2691.3	P	888.48	P	3.0291	P	YDR379C-A	S. cerevisiae	YDR379C-A	chr4:123326	---	---	chr4	YDR379C-A	---	Q3E785	---	---	---	---	0005739 // r	---	---	---	This probe s	S000007605(11),YDR379C-A(11)
1770879_at	1582.42	P	765.46	P	2.0673	P	YDR381C-A	S. cerevisiae	YDR381C-A	chr4:123829	---	---	chr4	YDR381C-A	---	Q3E6R5	---	---	---	---	0005739 // r	0005515 // r	---	S00000765(	This probe s	S000007650(11),YDR381C-A(11)
1778440_at	3926.1	P	1773.77	P	2.2134	P	YDR391C	S. cerevisiae	YDR391C.S	chr4:125734	---	---	chr4	YDR391C	---	Q04170	---	---	---	---	0005634 // r	0005515 // r	IPR000594 ,	---	This probe s	S000002799(11),GENEFINDER00000002830(11),GENSCAN00000008173(11),YDR391C(11)
1774988_at	58.69	P	24.92	M	2.3551	P	YDR402C	S. cerevisiae	YDR402C.S	chr4:127222	N-formyltyro	DIT2	chr4	YDR402C	---	P21595	---	---	---	0030435 // s	---	0004497 // r	---	S00000281(	This probe s	S000002810(11),GENSCAN00000008069(11),YDR402C(11)
1779681_at	621.25	P	50.04	P	12.4151	P	YDR403W	S. cerevisiae	YDR403W.S	chr4:127458	Sporulation-	DIT1	chr4	YDR403W	---	P21623	---	---	---	0030435 // s	---	0003824 // c	---	---	This probe s	S000002811(11),GENSCAN00000007987(11),GENEFINDER00000002576(9),YDR403W(11)
1778973_at	1570.34	P	617.65	P	2.5424	P	YDR406W	S. cerevisiae	YDR406W.S	chr4:127919	Plasma men	PDR15	chr4	YDR406W	---	Q04182	---	---	---	0006810 // t	0016020 // r	0000166 // r	---	S00000281	This probe s	S000002814(11),GENEFINDER00000002566(11),GENSCAN00000007777(11),YDR406W(11)
1774409_at	2270.34	P	835.07	P	2.7187	P	YDR423C	S. cerevisiae	YDR423C.S	chr4:131803	AP-1-like ba	CAD1	chr4	YDR423C	---	P24813	---	NP_010711	---	0006350 // t	0005634 // r	0003677 // [	NULL // NUL	---	This probe s	X69106(11)
1780130_at	4450.61	P	1624.65	P	2.7394	P	YDR435C	S. cerevisiae	YDR435C.S	chr4:133297	Carboxyl me	PPM1	chr4	YDR435C	---	Q04081	---	---	---	0006481 // (	---	0003880 // (	---	---	This probe s	S000002843(11),GENSCAN00000008222(11),YDR435C(11)
1774315_at	984.08	P	327.51	P	3.0047	P	YDR436W	S. cerevisiae	YDR436W.S	chr4:133480	Serine/threo	PPZ2	chr4	YDR436W	---	P33329	---	---	---	0006883 // c	---	0004721 // r	---	GENEFINDE	This probe s	S000002844(11),GENEFINDER00000002946(11),GENSCAN00000008137(11),YDR436W(11)
1774998_at	1061.42	P	381.37	P	2.7832	P	YDR438W	S. cerevisiae	YDR438W.S	chr4:133826	Mitochondria	THI74	chr4	YDR438W	---	Q04083	---	---	---	---	0005739 // r	---	IPR000620 ,	S00000284(	This probe s	S000002846(11),GENSCAN00000008100(11),YDR438W(11)
1771624_at	8886.5	P	641.95	P	13.843	P	YDR453C	S. cerevisiae	YDR453C.S	chr4:136505	Stress induc	TSA2	chr4	YDR453C	---	Q04120	---	---	---	0006979 // r	0005737 // c	0004601 // r	---	---	This probe s	S000002861(11),GENEFINDER00000002853(11),GENSCAN00000007902(11),YDR453C(11)
1776152_at	3384.56	P	1164.18	P	2.9072	P	YDR475C	S. cerevisiae	YDR475C.S	chr4:140745	Protein of ur	JIP4	chr4	YDR475C	---	Q03361	---	---	---	---	---	---	---	---	This probe s	S000002883(11),GENEFINDER00000002849(11),GENSCAN00000007985(11),YDR475C(11)
1769599_at	2169.27	P	650.07	P	3.337	P	YDR479C	S. cerevisiae	YDR479C.S	chr4:141519	Peroxisomal	PEX29	chr4	YDR479C	---	Q03370	---	---	---	0007031 // r	0005777 // r	0005515 // r	---	S00000288	This probe s	S000002887(11),GENEFINDER00000002735(11),GENSCAN00000007724(11),YDR479C(11)
1769443_at	279.91	P	136.3	P	2.0536	P	YDR501W	S. cerevisiae	YDR501W.S	chr4:145134	Forkhead As	PLM2	chr4	YDR501W	---	Q04383	---	---	---	0034984 // c	0000790 // r	0003682 // c	---	---	This probe s	S000002909(11),GENEFINDER00000002729(11),GENSCAN00000008210(11),YDR501W(11)
1771402_at	9380.75	P	2498.93	P	3.7539	P	YDR502C	S. cerevisiae	YDR502C.S	chr4:145329	S-adenosylm	SAM2	chr4	YDR502C	---	P19358 /// C	---	---	---	0006555 // r	---	0000166 // r	---	---	This probe s	S000002910(11),GENEFINDER00000002941(11),GENSCAN00000007963(11),YDR502C(11)
1773023_at	1317.2	P	595.63	P	2.2114	P	YDR512C	S. cerevisiae	YDR512C.S	chr4:147048	Non-essentia	EMI1	chr4	YDR512C	---	Q04406	---	---	---	0007126 // r	---	---	---	---	This probe s	S000002920(11),GENSCAN00000008068(11),YDR512C(11)
1777042_at	11574.73	P	5418.06	P	2.1363	P	YDR513W	S. cerevisiae	YDR513W.S	chr4:147100	Cytoplasmic	GRX2	chr4	YDR513W	---	P17695	---	---	---	0006810 // t	0005737 // c	0004364 // c	---	S00000292	This probe s	S000002921(11),YDR513W(11)
1770001_at	6599.51	P	3268.24	P	2.0193	P	YDR516C	S. cerevisiae	YDR516C.S	chr4:147496	Non-essentia	EMI2	chr4	YDR516C	---	Q04409	---	---	---	0005975 // c	0005737 // c	0000166 // r	---	---	This probe s	S000002924(11),GENEFINDER00000002745(11),GENSCAN00000008188(11),YDR516C(11)
1771657_at	6008.32	P	2121.53	P	2.8321	P	YDR525W-A	S. cerevisiae	YDR525W-A	chr4:149058	Protein of ur	SNA2	chr4	YDR525W-A	---	P56508	---	---	---	---	0005737 // c	---	---	S00000723	This probe s	S000007236(11),YDR525W-A(11)
1770402_at	2973.63	P	1406.33	P	2.1145	P	YDR530C	S. cerevisiae	YDR530C.S	chr4:149677	Diadenosine	APA2	chr4	YDR530C	---	P22108	---	---	---	0009117 // r	0005634 // r	0003824 // c	---	---	This probe s	S000002938(11),GENEFINDER00000002551(11),GENSCAN00000008219(11),YDR530C(11)
1774696_at	12368.11	P	2569.33	P	4.8137	P	YDR533C	S. cerevisiae	YDR533C.S	chr4:150143	Possible cha	HSP31	chr4	YDR533C	---	Q04432 /// (	---	---	---	0006950 // r	0005625 // s	0005515 // r	---	---	This probe s	S000002941(11),GENEFINDER00000002736(9),GENSCAN00000008214(11),YDR533C(11)
1771286_at	1713.17	P	721.79	P	2.3735	P	YDR541C	S. cerevisiae	YDR541C.S	chr4:151964	---	---	chr4	YDR541C	---	Q03049	---	---	---	0008152 // r	---	0003824 // c	IPR002225 ,	---	This probe s	S000002949(11),GENSCAN00000007949(11),YDR541C(11)
1779620_at	5226.22	P	195.79	P	26.6930	P	YEL073C	S. cerevisiae	YEL073C.S	chr5:7229-7	Putative prot	---	chr5	YEL073C	---	P39974	---	---	---	---	---	---	---	---	This probe s	S000000799(11),YEL073C(11)
1777421_at	1208.02	P	244.44	P	4.942	P	YEL072W	S. cerevisiae	YEL072W.S	chr5:13719-	Protein regu	RMD6	chr5	YEL072W	---	P39975	---	---	---	0007126 // r	---	---	---	---	This probe s	S000000798(11),GENSCAN00000004267(11),YEL072W(11)
1777667_s_at	375.02	P	109.81	P	3.4152	P	YEL070W	S. cerevisiae	YEL070W.S	chr5:19588-	Deletion sup	DSF1	chr14 /// chr	YEL070W //	---	P39941	---	---	---	0008152 // r	---	0003824 // c	---	GENEFINDE	This probe s	S000000796(11),S000005356(11),GENEFINDER00000000376(11),GENSCAN00000004099(11),GENEFINDER00000000821(11),GENSCAN00000005209(11),YEL070W(11),YNR073C(11)
1778254_at	145.1	P	58.63	P	2.4748	P	YEL057C	S. cerevisiae	YEL057C.S	chr5:45019-	---	---	chr5	YEL057C	---	P39983	---	---	---	---	---	---	---	---	This probe s	S000000783(11),YEL057C(11)
1778969_x_at	430.85	P	97.66	P	4.4117	P	YEL049W	S. cerevisiae	YEL049W.S	chr4:152322	Part of 23-m	PAU2	chr5	YEL049W	---	P32612 /// (	---	---	---	0006950 // r	0016020 // r	0005515 // r	---	---	This probe s	S000000775(11),GENEFINDER00000000269(11),GENSCAN00000004248(11),YEL049W(11)
1780079_at	454.77	P	129.15	P	3.5213	P	YEL041W	S. cerevisiae	YEL041W.S	chr5:75943-	ATP-NADH k	YEF1	chr5	YEL041W	---	P32622	---	---	---	0006741 // N	---	0003951 // N	---	---	This probe s	S000000767(11),GENSCAN00000004211(11),YEL041W(11)
1774137_at	7800.4	P	1505.53	P	5.1812	P	YEL039C	S. cerevisiae	YEL039C.S	chr5:79635-	Cytochrome	CYC7	chr5	YEL039C	---	P00045 /// C	---	---	---	0006122 // r	0005739 // r	0005506 // i	---	---	This probe s	S000000765(11),GENSCAN00000004197(11),YEL039C(11)
1775308_at	171.25	P	22.48	P	7.6179	P	YEL035C	S. cerevisiae	YEL035C.S	chr5:85044-	Protein of ur	UTR5	chr5	YEL035C	---	P32630	---	---	---	---	---	---	---	---	This probe s	S000000761(11),S66120(10),YEL035C(11)
1780036_at	2327.82	P	964.56	P	2.4133	P	YEL012W	S. cerevisiae	YEL012W.S	chr5:131897	Ubiquitin-cor	UBC8	chr5	YEL012W	---	P28263	---	---	---	0000209 // r	0005737 // c	0004842 // u	---	---	This probe s	S000000738(11),GENSCAN00000004240(11),GENEFINDER00000000257(11),YEL012W(11)
1777933_at	1373.98	P	629.98	P	2.181	P	YEL005C	S. cerevisiae	YEL005C.S	chr5:145905	Protein with	VAB2	chr5	YEL005C	---	P40003	---	---	---	---	0005737 // c	0005515 // r	---	---	This probe s	S000000731(11),GENSCAN00000004228(11),YEL005C(11)
1771757_at	473.03	P	200.38	P	2.3607	P	YER024W	S. cerevisiae	YER024W.S	chr5:202190	Carnitine ac	YAT2	chr5	YER024W	---	P40017	---	---	---	0006066 // c	0005737 // c	0004092 // c	---	---	This probe s	S000000826(11),GENEFINDER00000000325(10),GENSCAN00000004265(11),YER024W(11)
1772540_at	1537.45	P	278.74	P	5.5157	P	YER028C	S. cerevisiae	YER028C.S	chr5:210690	Probable tra	MIG3	chr5	YER028C	---	P39943	---	---	---	0000122 // r	0005622 // i	0003676 // r	---	---	This probe s	S000000830(11),GENSCAN00000004012(11),YER028C(11)
1778130_at	1580.42	P	421.55	P	3.7491	P	YER033C	S. cerevisiae	YER033C.S	chr5:218055	Protein of ur	ZRG8	chr5	YER033C	---	P40021	---	---	---	---	0005737 // c	0005515 // r	---	---	This probe s	S000000835(11),GENEFINDER00000000310(11),GENSCAN00000004021(11),YER033C(11)
1769450_at	4596.77	P	1750.97	P	2.6253	P	YER035W	S. cerevisiae	YER035W.S	chr5:222637	RNA-binding	EDC2	chr5	YER035W	---	P40023	---	---	---	0000184 // r	0005634 // r	0003723 // F	---	---	This probe s	S000000837(11),GENEFINDER00000000382(11),GENSCAN00000004000(11),YER035W(11)
1772785_at	5045.82	P	658.5	P	7.6626	P	YER037W	S. cerevisiae	YER037W.S	chr5:225887	Protein of ur	PHM8	chr5	YER037W	---	P40025	---	---	---	0008152 // r	0005634 // r	0003824 // c	---	---	This probe s	S000000839(11),GENSCAN00000004157(11),YER037W(11)
1779736_at	292.51	P	144.08	P	2.0302	P	YER039C-A	S. cerevisiae	YER039C-A	chr5:229261	---	---	chr5	YER039C-A	---	---	---	---	---	---	---	---	---	S00000722	This probe s	S000007226(11),YER039C-A(11)
1773675_at	6427.82	P	1150.02	P	5.5893	P	YER042W	S. cerevisiae	YER042W.S	chr5:234935	Methionine-S	MXR1	chr5	YER042W	---	P40029	---	---	---	0006979 // r	0005634 // r	0005515 // r	---	---	This probe s	S000000844(11),GENSCAN00000004040(11),YER042W(11)
1774685_at	2185.72	P	978.8	P	2.2331	P	YER045C	S. cerevisiae	YER045C.S	chr5:240030	Basic leucine	ACA1	chr5	YER045C	---	P39970	---	---	---	0006350 // t	0005634 // r	0003677 // [	IPR011700 ,	---	This probe s	S000000847(11),GENEFINDER00000000337(11),GENSCAN00000004123(11),YER045C(11)
1769679_at	14591.92	P	6697.74	P	2.1786	P	YER053C	S. cerevisiae	YER053C.S	chr5:258735	Mitochondria	PIC2	chr5	YER053C	---	P40035	---	---	---	0006810 // t	0005739 // r	0005315 // i	---	S00000085	This probe s	S000000855(11),GENEFINDER00000000255(9),GENSCAN00000004008(11),YER053C(11)
1779924_at	9228.72	P	2521.62	P	3.6598	P	YER053C-A	S. cerevisiae	YER053C-A	chr5:260931	---	---	chr5	YER053C-A	---	Q3E7B0	---	---	---	---	0005783 // e	0005515 // r	---	S00000752	This probe s	S000007523(11),YER053C-A(11)
1771802_at	1332.87	P	143.8	P	9.2689	P	YER054C	S. cerevisiae	YER054C.S	chr5:262050	Putative reg	GIP2	chr5	YER054C	---	P40036	---	---	---	0005977 // c	0000164 // r	0005515 // r	---	---	This probe s	S000000856(11),GENSCAN00000004054(11),YER054C(11)
1779235_at	1082.86	P	176.93	P	6.1203	P	YER060W	S. cerevisiae	YER060W.S	chr5:274564	Putative puri	FCY21	chr5	YER060W	---	P40039	---	---	---	0006810 // t	0016020 // r	0005515 // r	---	S00000086	This probe s	S000000862(11),GENEFINDER00000000333(9),GENSCAN00000004192(11),YER060W(11)
1775529_at	133.59	P	37.39	P	3.5729	P	YER060W-A	S. cerevisiae	YER060W-A	chr5:276569	Putative puri	FCY22	chr5	YER060W-A	---	Q12119	---	---	---	0006810 // t	0016020 // r	0005515 // r	---	S00000295	This probe s	S000002958(11),GENEFINDER00000000333(11),GENSCAN00000004098(11),YER060W-A(11)
1771712_at	1726.42	P	699.97	P	2.4664	P	YER066W	S. cerevisiae	YER066W.S	chr5:290239	Putative pro	RRT13	chr5	YER066W	---	P40042	---	---	---	---	---	---	---	---	This probe s	S000000868(11),GENSCAN00000004145(11),YER066W(11)
1773234_at	4867.41	P	1751.89	P	2.7784	P	YER079W	S. cerevisiae	YER079W.S	chr5:318915	---	---	chr5	YER079W	---	P40052	---	---	---	0034605 // c	0005634 // r	---	---	---	This probe s	S000000881(11),GENSCAN00000004143(11),YER079W(11)
1779031_at	2568.34	P	1126.36	P	2.2802	P	YER081W	S. cerevisiae	YER081W.S	chr5:322681	3-phosphogl	SER3	chr5	YER081W	---	P40054	---	---	---	0006564 // l	0005737 // c	0003824 // c	---	---	This probe s	S000000883(11),GENEFINDER00000000233(11),GENSCAN00000004062(11),YER081W(11)
1774859_at	9820.91	P	3797.74	P	2.586	P	YER091C	S. cerevisiae	YER091C.S	chr5:339859	Cobalamin-in	MET6	chr5	YER091C	---	P05694 /// C	---	---	---	0008652 // a	0005737 // c	0003871 // 5	---	---	This probe s	S000000893(11),GENEFINDER00000000354(11),GENSCAN00000004246(11),YER091C(11)
1779267_at	350.28	P	112.26	P	3.1203	P	YER096W	S. cerevisiae	YER096W.S	chr5:351693	Sporulation-	SHC1	chr5	YER096W	---	P39000	---	---	---	0006031 // c	0005628 // r	0005488 // b	---	---	This probe s	S000000898(11),GENEFINDER00000000383(11),GENSCAN00000004014(11),YER096W(11)
1777276_at	630.08	P	312.41	P	2.0168	P	YER098W	S. cerevisiae	YER098W.S	chr5:355461	Ubiquitin car	UBP9	chr5	YER098W	---	P39967	---	---	---	0006511 // u	0005737 // c	0004221 // u	---	---	This probe s	S000000900(11),GENEFINDER00000000228(10),GENSCAN00000004147(11),YER098W(11)
1770202_at	10605.84	P	1936.62	P	5.4765	P	YER103W	S. cerevisiae	YER103W.S	chr5:364584	Heat shock	SSA4	chr5	YER103W	---	P22202	---	---	---	0006457 // r	0005634 // r	0000166 // r	---	---	This probe s	S000000905(11),GENEFINDER00000000290(11),GENSCAN00000004084(11),YER103W(11)
1779959_at	3068.44	P	1155.12	P	2.6564	P	YER119C	S. cerevisiae	YER119C.S	chr5:399491	Vacuolar am	AVT6	chr5	YER119C	---	P40074	---	---	---	0006810 // t	0000324 // f	0005313 // L	---	S00000092	This probe s	S000000921(11),GENEFINDER00000000326(11),GENSCAN00000004101(11),YER119C(11)
1777510_at	4783.47	P	2352.43	P	2.0334	P	YER130C	S. cerevisiae	YER130C.S	chr5:421110	---	---	chr5	YER130C	---	P39959	---	---	---	---	0005622 // i	0003676 // r	---	---	This probe s	S000000932(11),GENSCAN00000004111(11),YER130C(11)
1771705_at	14822.41	P	7083.89	P	2.0924	P	YER150W	S. cerevisiae	YER150W.S	chr5:468364	GPI-anchore	SPI1	chr5	YER150W	---	P40092	---	---	---	0001101 // r	0009277 // f	---	---	---	This probe s	S000000952(11),GENEFINDER00000000287(10),GENSCAN00000004030(10),YER150W(11)
1779403_at	3447.45	P	1680.21	P	2.0518	P	YER158C	S. cerevisiae	YER158C.S	chr5:488851	---	---	chr5	YER158C	---	P40095	---	---	---	---	---	0005515 // r	---	---	This probe s	S000000960(11),GENEFINDER00000000338(11),GENSCAN00000004132(11),YER158C(11)
1778246_at	2552.33	P	747.45	P	3.4147	P	YER175C	S. cerevisiae	YER175C.S	chr5:539458	Trans-aconit	TMT1	chr5	YER175C	---	P32643	---	---	---	---	0005737 // c	0008168 // r	IPR003358 ,	---	This probe s	S000000977(11),GENEFINDER00000000334(11),GENSCAN00000004130(11),YER175C(11)
1772062_s_at	345.94	P	145.68	P	2.3747	P	YFL061W	S. cerevisiae	YFL061W.S	chr14:11451	Protein of ur	DDI2 /// DDI	chr14 /// chr	YFL061W //	---	P43543	---	---	---	0006974 // r	---	0003824 // c	---	---	This probe s	S000001833(11),S000005279(11),GENEFINDER00000000209(11),GENSCAN00000003566(11),GENEFINDER00000000810(11),GENSCAN00000005415(11),YFL061W(11),YNL335W(11)
1770343_s_at	702.1	P	292.98	P	2.3964	P	YFL059W	S. cerevisiae	YFL059W.S	chr14:13266	Member of a	SNZ2 /// SN	chr14 /// chr	YFL059W //	---	P43545 /// P	---	---	---	0008152 // r	---	0003824 // c	---	---	This probe s	S000001835(11),S000005277(11),GENSCAN00000003549(11),GENEFINDER00000000148(11),GENSCAN00000004926(11),GENEFINDER00000000724(11),YFL059W(11),YNL333W(11)
1773315_at	777.82	P	114.75	P	6.7784	P	YFL056C	S. cerevisiae	YFL056C.S	chr6:14792-	Putative aryl	AAD6	chr6	YFL056C	---	P43547 /// C	---	---	---	0009636 // r	---	0016491 // c	---	---	This probe s	S000001838(11),GENEFINDER00000000170(10),YFL056C(11)
1773737_at	364.28	P	42.8	P	8.5112	P	YFL055W	S. cerevisiae	YFL055W.S	chr6:17003-	Low-affinity	AGP3	chr6	YFL055W	---	P43548	---	---	---	0006810 // t	0016020 // r	0015171 // a	---	S00000183	This probe s	S000001839(11),GENEFINDER00000000202(11),GENSCAN00000003560(11),YFL055W(11)
1772292_at	2341.24	P	399.13	P	5.8659	P	YFL054C	S. cerevisiae	YFL054C.S	chr6:20846-	---	---	chr6	YFL054C	---	P43549	---	---	---	0006810 // t	0016020 // r	0005215 // t	---	S00000184	This probe s	S000001840(11),GENEFINDER00000000154(9),GENSCAN00000003414(11),YFL054C(11)
1776664_at	1751.18	P	17.71	A	98.8809	P	YFL053W	S. cerevisiae	YFL053W.S	chr6:23422-	Dihydroxyac	DAK2	chr6	YFL053W	---	P43550	---	---	---	0006071 // c	---	0004371 // c	---	---	This probe s	S000001841(11),GENEFINDER00000000186(11),GENSCAN00000003422(11),YFL053W(11)
1771045_at	648.86	P	182.03	P	3.5646	P	YFL050C	S. cerevisiae	YFL050C.S	chr6:33271-	Probable Mg	ALR2	chr6	YFL050C	---	P43553	---	---	---	0006810 // t	0005886 // p	0000287 // r	---	S00000184	This probe s	S000001844(11),GENSCAN00000003522(11),YFL050C(11)
1774767_at	1270.06	P	471.4	P	2.6942	P	YFL044C	S. cerevisiae	YFL044C.S	chr6:44654-	Deubiquityla	OTU1	chr6	YFL044C	---	P43558	---	---	---	0016579 // r	0005622 // i	0004843 // u	---	---	This probe s	S000001850(11),GENEFINDER00000000204(11),GENSCAN00000003480(11),YFL044C(11)
1776670_at	2457.58	P	905.55	P	2.7139	P	YFL042C	S. cerevisiae	YFL042C.S	chr6:45719-	---	---	chr6	YFL042C	---	P43560	---	---	---	---	0016020 // r	---	---	S00000185	This probe s	S000001852(11),GENEFINDER00000000185(11),GENSCAN00000003403(11),YFL042C(11)
1780131_at	1324.16	P	487.67	P	2.7153	P	YFL041W-A	S. cerevisiae	YFL041W-A	chr6:48733-	---	---	chr6	YFL041W-A	---	Q3E817	---	---	---	---	---	---	---	---	This probe s	S000028547(11),YFL041W-A(11)
1770767_at	2710.41	P	370.45	P	7.3165	P	YFL030W	S. cerevisiae	YFL030W.S	chr6:76828-	Alanine:glyo	AGX1	chr6	YFL030W	---	P43567	---	---	---	0008152 // r	0005739 // r	0003824 // c	---	---	This probe s	S000001864(11),GENEFINDER00000000150(11),GENSCAN00000003416(11),YFL030W(11)
1776687_at	3216.88	P	1278.66	P	2.5158	P	YFL021W	S. cerevisiae	YFL021W.S	chr6:95963-	Transcriptio	GAT1	chr6	YFL021W	---	P43574	---	---	---	0006350 // t	0005634 // r	0003677 // [	---	---	This probe s	S000001873(11),GENEFINDER00000000191(11),GENSCAN00000003516(11),YFL021W(11)
1771323_at	557.27	P	141.25	P	3.9453	P	YFL020C	S. cerevisiae	YFL020C.S	chr1:176652	Part of 23-m	PAU5	chr6	YFL020C	---	P39545 /// P	---	---	---	0006950 // r	0000299 // i	---	---	---	This probe s	S000001874(11),GENEFINDER00000000195(11),GENSCAN00000003458(11),YFL020C(11)
1770897_x_at	580.6	P	106.35	P	5.4593	P	YFL020C	S. cerevisiae	YFL020C.S	chr1:176652	Part of 23-m	PAU5	chr6	YFL020C	---	P39545 /// P	---	---	---	0006950 // r	0000299 // i	---	---	---	This probe s	S000001874(11),GENEFINDER00000000195(11),GENSCAN00000003458(11),YFL020C(11)
1776246_at	5573.41	P	2163.89	P	2.5756	P	YFL016C	S. cerevisiae	YFL016C.S	chr6:104694	Co-chaperon	MDJ1	chr6	YFL016C	---	P35191	---	---	---	0000002 // r	0005739 // r	0001671 // A	---	---	This probe s	S000001878(11),GENEFINDER00000000210(11),GENSCAN00000003380(11),YFL016C(11)
1779754_at	15655.88	P	1731.84	P	9.04	P	YFL014W	S. cerevisiae	YFL014W.S	chr6:107249	Plasma men	HSP12	chr6	YFL014W	---	P22943	---	NP_116640	---	0006950 // r	0005634 // r	0005515 // r	---	---	This probe s	X55785(11)
1774565_at	1346.31	P	631.73	P	2.1311	P	YFR007W	S. cerevisiae	YFR007W.S	chr6:159292	Putative kina	YFH7	chr6	YFR007W	---	P43591	---	---	---	---	---	0005515 // r	---	---	This probe s	S000001903(11),GENSCAN00000003475(11),YFR007W(11)
1773415_at	5425.13	P	1913.34	P	2.8354	P	YFR017C	S. cerevisiae	YFR017C.S	chr6:182261	---	---	chr6	YFR017C	---	P43598	---	---	---	---	0005737 // c	0005515 // r	---	---	This probe s	S000001913(11),GENEFINDER00000000181(11),GENSCAN00000003517(11),YFR017C(11)
1772831_at	4557.17	P	528.82	P	8.6176	P	YFR022W	S. cerevisiae	YFR022W.S	chr6:196820	Protein that	ROG3	chr6	YFR022W	---	P43602	---	---	---	---	---	0005515 // r	IPR011021 ,	---	This probe s	S000001918(11),GENSCAN00000003372(11),GENEFINDER00000000178(11),YFR022W(11)
1770319_at	3378	P	340.11	P	9.9321	P	YFR030W	S. cerevisiae	YFR030W.S	chr6:213299	Subunit alph	MET10	chr6	YFR030W	---	A0SXI4 /// A	---	---	---	0000103 // s	0009337 // s	0003824 // c	---	---	This probe s	S000001926(11),GENEFINDER00000000189(11),GENSCAN00000003502(11),YFR030W(11)
1775539_at	76.71	P	29.08	A	2.6379	P	YGL262W	S. cerevisiae	YGL262W.S	chr7:5311-5	---	---	chr7	YGL262W	---	P53054	---	---	---	---	---	0005515 // r	---	---	This probe s	S000003231(11),YGL262W(11)
1771083_at	435.16	P	47.69	P	9.1248	P	YGL259W	S. cerevisiae	YGL259W.S	chr7:8469-8	Protein with	YPS5	chr7	YGL259W	---	P53057	---	---	---	0006508 // r	---	0004190 // a	---	---	This probe s	S000003228(11),GENSCAN00000006564(11),YGL259W(11)
1777239_at	836.21	P	40.91	P	20.4402	P	YGL258W-A	S. cerevisiae	YGL258W-A	chr7:9161-9	---	---	chr7	YGL258W-A	---	Q3E740	---	---	---	0006508 // r	---	0004190 // a	---	---	This probe s	S000007607(11),YGL258W-A(11)
1777854_at	4366.28	P	1433.02	P	3.0469	P	YGL248W	S. cerevisiae	YGL248W.S	chr7:35652-	Low-affinity	PDE1	chr7	YGL248W	---	P22434	---	---	---	0006198 // c	---	0004114 // 3	---	---	This probe s	S000003217(11),GENSCAN00000005708(11),YGL248W(11)
1772343_at	1524.55	P	457.99	P	3.3288	P	YGL229C	S. cerevisiae	YGL229C.S	chr7:64501-	Protein regu	SAP4	chr7	YGL229C	---	P53036	---	---	---	0000082 // (	0005739 // r	---	---	---	This probe s	S000003198(11),GENEFINDER00000002344(11),GENSCAN00000005623(11),YGL229C(11)
1777109_at	1007.9	P	469.13	P	2.1484	P	YGL227W	S. cerevisiae	YGL227W.S	chr7:69669-	Protein invol	VID30	chr7	YGL227W	---	P53076	---	---	---	0006808 // r	0005634 // r	0005515 // r	IPR013720 ,	---	This probe s	S000003196(11),GENSCAN00000005607(11),GENEFINDER00000002411(11),YGL227W(11)
1769379_at	1494.4	P	673.36	P	2.2193	P	YGL222C	S. cerevisiae	YGL222C.S	chr7:80656-	RNA-binding	EDC1	chr7	YGL222C	---	P53080	---	---	---	0000184 // r	0005737 // c	0003723 // F	---	---	This probe s	S000003190(11),YGL222C(11)
1776136_at	1187.25	P	481.61	P	2.4652	P	YGL208W	S. cerevisiae	YGL208W.S	chr7:97339-	One of three	SIP2	chr7	YGL208W	---	P34164	---	---	---	0001302 // r	0005737 // c	0004679 // A	---	---	This probe s	S000003176(11),GENSCAN00000005892(11),GENEFINDER00000002453(11),YGL208W(11)
1771997_at	767.18	P	248.03	P	3.0931	P	YGL185C	S. cerevisiae	YGL185C.S	chr7:153170	---	---	chr7	YGL185C	---	P53100	---	---	---	0008152 // r	0005737 // c	0003824 // c	---	---	This probe s	S000003153(11),GENSCAN00000005879(11),YGL185C(11)
1777388_at	6061.69	P	151.2	P	40.0905	P	YGL184C	S. cerevisiae	YGL184C.S	chr7:154618	Cystathionin	STR3	chr7	YGL184C	---	P53101	---	---	---	0006520 // a	0005634 // r	0003824 // c	---	GENEFINDE	This probe s	S000003152(11),GENEFINDER00000002485(9),GENSCAN00000006144(11),YGL184C(11)
1776041_at	1109.47	P	379.43	P	2.924	P	YGL180W	S. cerevisiae	YGL180W.S	chr7:160068	Protein ser/t	ATG1	chr7	YGL180W	---	P53104	---	---	---	0000045 // a	0000407 // r	0000166 // r	---	---	This probe s	S000003148(11),GENEFINDER00000002318(11),GENSCAN00000005676(11),YGL180W(11)
1769837_at	2826.54	P	905.43	P	3.1218	P	YGL179C	S. cerevisiae	YGL179C.S	chr7:163412	Protein kina	TOS3	chr7	YGL179C	---	P43637	---	---	---	0006006 // c	0005634 // r	0000166 // r	IPR001245 ,	---	This probe s	S000003147(11),GENSCAN00000005907(11),YGL179C(11)
1777697_at	5367.63	P	1900.5	P	2.8243	P	YGL157W	S. cerevisiae	YGL157W.S	chr7:209008	---	---	chr7	YGL157W	---	P53111	---	---	---	0008152 // r	0005634 // r	0003824 // c	IPR002225 ,	---	This probe s	S000003125(11),GENEFINDER00000002368(11),GENSCAN00000005866(11),YGL157W(11)
1771498_at	1380.85	P	279.18	P	4.9461	P	YGL156W	S. cerevisiae	YGL156W.S	chr7:210418	Vacuolar alp	AMS1	chr7	YGL156W	---	P22855	---	---	---	0005975 // c	0005773 // v	0003824 // c	---	---	This probe s	S000003124(11),GENEFINDER00000002524(9),GENSCAN00000006487(11),YGL156W(11)
1779032_at	2902.07	P	945.86	P	3.0682	P	YGL154C	S. cerevisiae	YGL154C.S	chr7:215280	Phosphopan	LYS5	chr7	YGL154C	---	P50113	---	---	---	0009059 // r	0005737 // c	0000287 // r	---	---	This probe s	S000003122(11),YGL154C(11)
1775996_at	346.48	P	159.78	P	2.1685	P	YGL146C	S. cerevisiae	YGL146C.S	chr7:228753	Putative pro	RRT6	chr7	YGL146C	---	P53117	---	---	---	---	0016020 // r	---	---	S00000311	This probe s	S000003114(11),GENEFINDER00000002276(9),GENSCAN00000006083(11),YGL146C(11)
1774207_at	1565.43	P	739.87	P	2.1158	P	YGL141W	S. cerevisiae	YGL141W.S	chr7:238355	Ubiquitin-cor	HUL5	chr7	YGL141W	---	P53119	---	---	---	0000209 // r	0000502 // r	0005515 // r	---	---	This probe s	S000003109(11),GENSCAN00000006381(11),YGL141W(11)
1778144_at	1892.47	P	280.67	P	6.7427	P	YGL125W	S. cerevisiae	YGL125W.S	chr7:272523	Major isozyr	MET13	chr7	YGL125W	---	P53128	---	---	---	0006555 // r	0005739 // r	0004489 // r	---	---	This probe s	S000003093(11),GENEFINDER00000002433(11),GENSCAN00000005841(11),YGL125W(11)
1769579_at	5539.78	P	517.56	P	10.7036	P	YGL121C	S. cerevisiae	YGL121C.S	chr7:280780	Proposed ga	GPG1	chr7	YGL121C	---	P53130	---	---	---	0001403 // i	0005737 // c	0004871 // s	---	---	This probe s	S000003089(11),GENEFINDER00000002410(11),YGL121C(11)
1769804_at	2029.38	P	879.36	P	2.3078	P	YGL104C	S. cerevisiae	YGL104C.S	chr7:308713	Mitochondria	VPS73	chr7	YGL104C	---	P53142	---	---	---	0006623 // r	0005739 // r	0005215 // t	---	S00000307	This probe s	S000003072(11),YGL104C(11)
1771222_at	4978.21	P	468.96	P	10.6154	P	YGL096W	S. cerevisiae	YGL096W.S	chr7:325331	Homeodoma	TOS8	chr7	YGL096W	---	P53147	---	---	---	0006355 // r	0000790 // r	0003677 // [	IPR001356 ,	---	This probe s	S000003064(11),GENSCAN00000006160(11),YGL096W(11)
1770381_at	246.28	P	119.69	P	2.0576	P	YGL089C	S. cerevisiae	YGL089C.S	chr7:344791	Mating phero	MF(ALPHA)	chr7	YGL089C	---	P32435	---	---	---	0000750 // r	0005576 // e	0000772 // r	---	---	This probe s	S000003057(11),GENEFINDER00000002376(11),YGL089C(11)
1769313_at	1490.92	P	632.73	P	2.3563	P	YGL059W	S. cerevisiae	YGL059W.S	chr7:392225	Mitochondria	PKP2	chr7	YGL059W	---	P53170	---	---	---	0015976 // c	0005739 // r	0004672 // r	IPR003594 ,	---	This probe s	S000003027(11),GENSCAN00000005600(11),YGL059W(11)
1775255_at	1958.1	P	711.18	P	2.7533	P	YGL053W	S. cerevisiae	YGL053W.S	chr7:402589	Pheromone-	PRM8	chr7	YGL053W	---	P53174 /// C	---	---	---	0000747 // c	0005783 // e	0005515 // r	---	S00000302	This probe s	S000003021(11),GENSCAN00000005698(11),YGL053W(11)
1777414_at	3970.43	P	735.44	P	5.3987	P	YGL045W	S. cerevisiae	YGL045W.S	chr7:414103	Protein of ur	RIM8	chr7	YGL045W	---	P53179	---	---	---	0001403 // i	---	---	---	---	This probe s	S000003013(11),GENSCAN00000005869(11),GENEFINDER00000002367(10),YGL045W(11)
1777374_at	8545.44	P	3471.04	P	2.4619	P	YGL009C	S. cerevisiae	YGL009C.S	chr7:476315	Isopropylma	LEU1	chr7	YGL009C	---	P07264	---	---	---	0008152 // r	0005625 // s	0003861 // 3	---	---	This probe s	S000002977(11),GENEFINDER00000002491(11),GENSCAN00000006047(11),YGL009C(11)
1770017_at	3058.88	P	1325.77	P	2.3072	P	YGL006W	S. cerevisiae	YGL006W.S	chr7:485922	Vacuolar Ca	PMC1	chr7	YGL006W	---	P38929	---	---	---	0006754 // A	0000329 // f	0000166 // r	---	S00000297	This probe s	S000002974(11),GENSCAN00000006153(11),YGL006W(11)
1773255_at	10573.56	P	4937.47	P	2.1415	P	YGR008C	S. cerevisiae	YGR008C.S	chr7:508110	Protein invol	STF2	chr7	YGR008C	---	P16965	---	---	---	0006970 // r	0005739 // r	0005515 // r	---	---	This probe s	S000003240(11),GENSCAN00000005871(11),YGR008C(11)
1774010_at	4945.84	P	1817.72	P	2.7209	P	YGR019W	S. cerevisiae	YGR019W.S	chr7:525229	Gamma-ami	UGA1	chr7	YGR019W	---	A5H0J7 /// F	---	---	---	0009448 // c	0005622 // i	0003824 // c	---	---	This probe s	S000003251(11),GENEFINDER00000002450(11),GENSCAN00000005930(11),YGR019W(11)
1779035_at	1443.22	P	562.24	P	2.5669	P	YGR023W	S. cerevisiae	YGR023W.S	chr7:529264	Protein with	MTL1	chr7	YGR023W	---	P53214	---	---	---	0006950 // r	0016020 // r	---	---	S00000325	This probe s	S000003255(11),YGR023W(11)
1778735_at	688.8	P	221.87	P	3.1045	P	YGR035C	S. cerevisiae	YGR035C.S	chr7:557072	---	---	chr7	YGR035C	---	P53222 /// C	---	---	---	---	---	---	---	---	This probe s	S000003267(11),GENSCAN00000005992(11),YGR035C(11)
1774777_at	4465.51	P	334.88	P	13.3347	P	YGR043C	S. cerevisiae	YGR043C.S	chr7:580435	Transaldolas	NQM1	chr7	YGR043C	---	P53228 /// C	---	---	---	0005975 // c	0005634 // r	0003824 // c	---	---	This probe s	S000003275(11),GENEFINDER00000002472(11),GENSCAN00000006543(11),YGR043C(11)
1780221_at	4102.39	P	2027.71	P	2.0232	P	YGR044C	S. cerevisiae	YGR044C.S	chr7:582990	Zinc finger p	RME1	chr7	YGR044C	---	P32338 /// C	---	---	---	0000122 // r	0005622 // i	0003677 // [	---	---	This probe s	S000003276(11),GENSCAN00000005813(11),YGR044C(11)
1773998_at	490.55	P	150.85	P	3.2519	P	YGR045C	S. cerevisiae	YGR045C.S	chr7:583934	---	---	chr7	YGR045C	---	P53229	---	---	---	---	0016020 // r	---	---	S00000327	This probe s	S000003277(11),YGR045C(11)
1772958_at	5061.66	P	625.6	P	8.0909	P	YGR052W	S. cerevisiae	YGR052W.S	chr7:593596	Putative pro	FMP48	chr7	YGR052W	---	P53233 /// C	---	---	---	0006468 // r	0005739 // r	0000166 // r	IPR001245 ,	---	This probe s	S000003284(11),GENSCAN00000006515(11),GENEFINDER00000002545(11),YGR052W(11)
1771288_at	764.76	P	355.18	P	2.1532	P	YGR053C	S. cerevisiae	YGR053C.S	chr7:594986	---	---	chr7	YGR053C	---	P53234 /// C	---	---	---	---	---	---	---	---	This probe s	S000003285(11),GENSCAN00000006316(11),YGR053C(11)
1774026_at	10036.66	P	2483.7	P	4.041	P	YGR055W	S. cerevisiae	YGR055W.S	chr7:599417	High affinity	MUP1	chr7	YGR055W	---	P50276	---	---	---	0000101 // s	0016020 // r	0000102 // L	IPR004841 ,	S00000328	This probe s	S000003287(11),GENEFINDER00000002513(11),GENSCAN00000006324(11),YGR055W(11)
1773662_at	743.53	P	67.16	P	11.071	P	YGR066C	S. cerevisiae	YGR066C.S	chr7:620921	---	---	chr7	YGR066C	---	P53242 /// C	---	---	---	---	---	0005515 // r	---	---	This probe s	S000003298(11),GENSCAN00000006156(11),YGR066C(11)
1780215_at	1019.7	P	299.61	P	3.4034	P	YGR070W	S. cerevisiae	YGR070W.S	chr7:627806	GDP/GTP e	ROM1	chr7	YGR070W	---	P53046 /// C	---	---	---	0007015 // a	0005622 // i	0004871 // s	---	---	This probe s	S000003302(11),GENSCAN00000006394(11),YGR070W(11)
1771692_at	365.09	P	42.96	P	8.4984	P	YGR087C	S. cerevisiae	YGR087C.S	chr7:651290	Minor isofor	PDC6	chr7	YGR087C	---	P26263	---	---	---	0000949 // a	0005737 // c	0000287 // r	---	---	This probe s	S000003319(11),GENEFINDER00000002537(11),GENSCAN00000006530(11),YGR087C(11)

1769955_at	8400.53	P	645.3	P	13.018	P	YGR088W	S. cerevisiae	YGR088W S	chr7:654601	Cytosolic ca	CTT1	chr7	YGR088W	---	P06115	---	---	---	0000302 // r	0005737 // c	0004096 // c	---	---	This probe s	S000003320(11),GENEFINDER00000002338(11),GENSCAN00000006327(11),YGR088W(11)
1769609_at	1346.8	P	278.69	P	4.8326	P	YGR110W	S. cerevisiae	YGR110W S	chr7:713709	Mitochondria	CLD1	chr7	YGR110W	---	P53264	---	---	---	0032048 // c	0005739 // r	0004623 // r	---	---	This probe s	S000003342(11),GENSCAN00000005933(11),YGR110W(11)
1777175_at	410.3	P	154.4	P	2.6574	P	YGR121C	S. cerevisiae	YGR121C S	chr7:731450	Ammonium r	MEP1	chr7	YGR121C	---	P40260	---	---	---	0006810 // t	0005886 // r	0005515 // r	---	S000003353	This probe s	S000003353(11),GENEFINDER00000002407(11),GENSCAN00000005948(11),YGR121C(11)
1774571_at	256.27	P	111.29	P	2.3027	P	YGR126W	S. cerevisiae	YGR126W S	chr7:745836	---	---	chr7	YGR126W	---	P53274	---	---	---	---	0005634 // r	---	---	---	This probe s	S000003358(11),GENEFINDER00000002299(11),GENSCAN00000006092(11),YGR126W(11)
1777223_at	5554.25	P	1200.13	P	4.628	P	YGR127W	S. cerevisiae	YGR127W S	chr7:746799	---	---	chr7	YGR127W	---	P53275	---	---	---	---	---	---	IPR013101 ,	---	This probe s	S000003359(11),GENSCAN00000005741(11),YGR127W(11)
1776565_at	461.15	P	164.18	P	2.8088	P	YGR131W	S. cerevisiae	YGR131W S	chr7:754727	---	---	chr7	YGR131W	---	P53279	---	---	---	---	0016020 // r	---	---	S000003363	This probe s	S000003363(11),YGR131W(11)
1770071_at	9340.99	P	160.5	P	58.1993	P	YGR144W	S. cerevisiae	YGR144W S	chr7:780400	Thiazole syn	THI4	chr7	YGR144W	---	P32318	---	---	---	0000002 // r	0005739 // r	0005515 // r	IPR002218 ,	S000003376	This probe s	S000003376(11),GENEFINDER00000002470(11),GENSCAN00000005959(11),YGR144W(11)
1771432_at	3036.95	P	832.62	P	3.6475	P	YGR149W	S. cerevisiae	YGR149W S	chr7:789032	---	---	chr7	YGR149W	---	P48236	---	---	---	---	0016020 // r	---	---	S000003381	This probe s	S000003381(11),YGR149W(11)
1776630_at	970.7	P	155.88	P	6.2272	P	YGR154C	S. cerevisiae	YGR154C S	chr7:796799	Omega-clas	GTO1	chr7	YGR154C	---	P48239	---	---	---	---	0005777 // r	0004364 // c	---	---	This probe s	S000003386(11),GENSCAN00000005771(11),YGR154C(11)
1780217_at	3394.36	P	1573.22	P	2.1576	P	YGR161C	S. cerevisiae	YGR161C S	chr7:808630	Putative con	RTS3	chr7	YGR161C	---	P53289 /// C	---	---	---	0006470 // r	0005634 // r	---	---	---	This probe s	S000003393(11),GENEFINDER00000002396(11),GENSCAN00000005859(11),YGR161C(11)
1769723_at	257.64	P	40.17	P	6.4137	P	YGR174W-A	S. cerevisiae	YGR174W-A	chr7:846656	---	---	chr7	YGR174W-A	---	Q8TGT8	---	---	---	---	---	---	---	---	This probe s	S000028639(11),YGR174W-A(11)
1777740_x_at	1246.36	P	225.41	P	5.5293	P	YGR174W-A	S. cerevisiae	YGR174W-A	chr7:846656	---	---	chr7	YGR174W-A	---	Q8TGT8	---	---	---	---	---	---	---	---	This probe s	S000028639(11),YGR174W-A(11)
1778604_at	2865.06	P	1286.24	P	2.2275	P	YGR194C	S. cerevisiae	YGR194C S	chr7:886074	Xylulokinase	XKS1	chr7	YGR194C	---	P42826 /// C	---	---	---	0005975 // c	0005737 // c	0004856 // x	IPR000577 ,	---	This probe s	S000003426(11),GENSCAN00000006351(11),YGR194C(11)
1771117_at	6667.69	P	528	P	12.6282	P	YGR197C	S. cerevisiae	YGR197C S	chr7:892498	Protein invol	SNG1	chr7	YGR197C	---	P46950	---	---	---	0042493 // r	0016020 // r	---	---	S000003429	This probe s	S000003429(11),GENEFINDER00000002445(11),GENSCAN00000006489(11),YGR197C(11)
1771986_at	3958.76	P	808.08	P	4.899	P	YGR201C	S. cerevisiae	YGR201C S	chr7:902521	---	---	chr7	YGR201C	---	P42936	---	---	---	---	---	---	---	---	This probe s	S000003433(11),GENEFINDER00000002399(10),GENSCAN00000005834(11),YGR201C(11)
1771363_at	222.79	P	92.01	P	2.4214	P	YGR213C	S. cerevisiae	YGR213C S	chr7:918513	Protein invol	RTA1	chr7	YGR213C	---	P53047 /// C	---	---	---	0006950 // r	0016020 // r	---	---	S000003445	This probe s	S000003445(11),YGR213C(11)
1776680_at	388.07	P	69.68	P	5.5693	P	YGR236C	S. cerevisiae	YGR236C S	chr7:962429	Protein regu	SPG1	chr7	YGR236C	---	P50088	---	---	---	---	0005739 // r	0000166 // r	---	S000003468	This probe s	S000003468,GENEFINDER00000002456,YGR236C
1777017_at	1722.65	P	777.24	P	2.2164	P	YGR237C	S. cerevisiae	YGR237C S	chr7:963300	---	---	chr7	YGR237C	---	P50089	---	---	---	---	0005737 // c	---	---	GENEFINDE	This probe s	S000003469(11),GENEFINDER00000002456(11),GENSCAN00000006282(11),YGR237C(11)
1777561_at	935.57	P	388.9	P	2.4057	P	YGR243W	S. cerevisiae	YGR243W S	chr7:977338	Putative pro	FMP43	chr7	YGR243W	---	P53311	---	---	---	---	0005739 // r	---	---	---	This probe s	S000003475(11),YGR243W(11)
1774540_at	8113.21	P	1785.13	P	4.5449	P	YGR248W	S. cerevisiae	YGR248W S	chr7:985974	6-phosphogl	SOL4	chr7	YGR248W	---	P53315	---	---	---	0006098 // r	0005634 // r	0016787 // h	---	---	This probe s	S000003480(11),GENEFINDER00000002297(11),GENSCAN00000006128(11),YGR248W(11)
1776257_at	8261.59	P	2353.28	P	3.5107	P	YGR250C	S. cerevisiae	YGR250C S	chr7:991178	---	---	chr7	YGR250C	---	P53316	---	---	---	---	0005737 // c	0000166 // r	---	---	This probe s	S000003482(11),GENEFINDER00000002469(11),GENSCAN00000005837(11),YGR250C(11)
1773961_at	4003.06	P	180.27	P	22.2059	P	YGR256W	S. cerevisiae	YGR256W S	chr7:100462	6-phosphogl	GND2	chr7	YGR256W	---	P53319	---	---	---	0006098 // r	0005829 // c	0003824 // c	---	---	This probe s	S000003488(11),GENEFINDER00000002288(11),GENSCAN00000006523(11),YGR256W(11)
1770859_at	257.3	P	96.22	P	2.6741	P	YGR287C	S. cerevisiae	YGR287C S	chr7:106722	---	---	chr7	YGR287C	---	P53051 /// C	---	---	---	0000023 // r	0005739 // r	0003824 // c	---	---	This probe s	S000003519(11),AB109221(11),GENEFINDER00000002471(11),GENSCAN00000005829(11),YGR287C(11)
1772873_at	813.04	P	293.03	P	2.7746	P	YGR288W	S. cerevisiae	YGR288W S	chr7:107029	MAL-activat	MAL13	chr7	YGR288W	---	P53338 /// C	---	---	---	0000023 // r	0005634 // r	0003677 // [	---	---	This probe s	S000003520(11),GENSCAN00000006332(11),YGR288W(11)
1776944_at	1310.45	P	320.14	P	4.0934	P	YGR289C	S. cerevisiae	YGR289C S	chr7:107396	Inducible hig	MAL11	chr7	YGR289C	---	A6N8U8 /// A	---	---	---	0000017 // a	0016020 // r	0005215 // t	---	S000003521	This probe s	S000003521(11),GENEFINDER00000002435(11),GENSCAN00000006463(11),YGR289C(11)
1771691_at	3905.94	P	129.82	P	30.0874	P	YHL048C-A	S. cerevisiae	YHL048C-A	chr10:74430	---	---	chr8	YHL048C-A	---	Q3E758	---	---	---	---	---	---	---	---	This probe s	S000028829(11),YHL048C-A(11)
1776679_at	1116.02	P	461.98	P	2.4157	P	YHL047C	S. cerevisiae	YHL047C S	chr8:8297-1	Transporter	ARN2	chr8	YHL047C	---	P38724	---	---	---	0006810 // t	0005768 // e	0005506 // i	---	S000001039	This probe s	S000001039(11),GENEFINDER00000000524(11),GENSCAN00000003859(11),YHL047C(11)
1779914_at	4807.6	P	917.24	P	5.2414	P	YHL044W	S. cerevisiae	YHL044W S	chr8:13562-	---	---	chr8	YHL044W	---	P38727	---	---	---	---	0005886 // r	0005515 // r	---	S000001036	This probe s	S000001036(11),YHL044W(11)
1772536_at	563.45	P	77.6	P	7.261	P	YHL043W	S. cerevisiae	YHL043W S	chr8:14898-	Putative pro	ECM34	chr8	YHL043W	---	P38728	---	---	---	0007047 // c	0016020 // r	---	---	S000001035	This probe s	S000001035(11),GENSCAN00000003865(11),YHL043W(11)
1772066_at	676.65	P	138.94	P	4.8701	P	YHL042W	S. cerevisiae	YHL042W S	chr8:15664-	---	---	chr8	YHL042W	---	P38729	---	---	---	---	---	0005515 // r	---	S000001034	This probe s	S000001034(11),YHL042W(11)
1773748_at	7797.57	P	1040.78	P	7.492	P	YHL036W	S. cerevisiae	YHL036W S	chr8:26238-	Low affinity	MUP3	chr8	YHL036W	---	P38734	---	---	---	0006810 // t	0016020 // r	0015171 // a	IPR004841 ,	S000001028	This probe s	S000001028(11),GENSCAN00000003776(11),YHL036W(11)
1769845_at	1856.52	P	708.53	P	2.6202	P	YHL032C	S. cerevisiae	YHL032C S	chr8:36376-	Glycerol kin	GUT1	chr8	YHL032C	---	P32190	---	---	---	0005975 // c	0005737 // c	0000166 // r	---	---	This probe s	S000001024(11),GENEFINDER00000000551(11),GENSCAN00000003791(11),YHL032C(11)
1775842_at	6365.15	P	1789.38	P	3.5572	P	YHL021C	S. cerevisiae	YHL021C S	chr8:64457-	Putative pro	AIM17	chr8	YHL021C	---	P23180	---	---	---	0034605 // c	0005739 // r	0005506 // i	---	---	This probe s	S000001013(11),GENEFINDER00000000598(11),GENSCAN00000003867(11),YHL021C(11)
1778128_at	976.77	P	92.55	P	10.554	P	YHL016C	S. cerevisiae	YHL016C S	chr8:72032-	Plasma mem	DUR3	chr8	YHL016C	---	P33413	---	---	---	0006810 // t	0005886 // r	0005215 // t	IPR001734 ,	S000001008	This probe s	S000001008(11),GENEFINDER00000000663(9),GENSCAN00000003930(11),YHL016C(11)
1775410_at	1845.23	P	752.01	P	2.4537	P	YHL008C	S. cerevisiae	YHL008C S	chr8:92621-	---	---	chr8	YHL008C	---	P38750	---	---	---	0006810 // t	0000324 // f	0005215 // t	IPR000292 ,	S000001000	This probe s	S000001000(11),GENEFINDER00000000624(11),GENSCAN00000003941(11),YHL008C(11)
1771693_at	275.65	P	131.5	P	2.0962	P	YHL006C	S. cerevisiae	YHL006C S	chr8:98337-	Protein invol	SHU1	chr8	YHL006C	---	P38751	---	---	---	0000725 // r	0005634 // r	0005515 // r	---	---	This probe s	S000000998(11),GENEFINDER00000000567(11),YHL006C(11)
1769451_at	1290.63	P	640.01	P	2.0166	P	YHR002W	S. cerevisiae	YHR002W S	chr8:108804	Mitochondria	LEU5	chr8	YHR002W	---	P38702	---	---	---	0006810 // t	0005739 // r	0005488 // b	---	S000001044	This probe s	S000001044(11),YHR002W(11)
1770203_at	1558.25	P	685.49	P	2.2732	P	YHR006W	S. cerevisiae	YHR006W S	chr8:117806	Transcriptio	STP2	chr8	YHR006W	---	P38704 /// C	---	---	---	0008033 // t	0005622 // i	0003704 // s	---	---	This probe s	S000001048(11),GENEFINDER00000000582(11),GENSCAN00000003828(11),YHR006W(11)
1770189_at	365.55	P	70.38	P	5.1939	P	YHR007C-A	S. cerevisiae	YHR007C-A	chr8:122542	---	---	chr8	YHR007C-A	---	Q07074	---	---	---	---	0016020 // r	---	---	S000028830	This probe s	S000028830(11),YHR007C-A(11)
1778006_at	4197.35	P	984.64	P	4.2628	P	YHR016C	S. cerevisiae	YHR016C S	chr8:136871	Actin-binding	YSC84	chr8	YHR016C	---	P32793	---	---	---	0006897 // e	0005737 // c	0005515 // r	---	---	This probe s	S000001058(11),GENEFINDER00000000623(11),GENSCAN00000003945(11),YHR016C(11)
1778124_at	2645	P	1119.12	P	2.3635	P	YHR017W	S. cerevisiae	YHR017W S	chr8:138684	Non-essenti	YSC83	chr8	YHR017W	---	P32792	---	---	---	---	0005739 // r	---	---	---	This probe s	S000001059(11),YHR017W(11)
1776087_at	211.94	P	98.98	P	2.1412	P	YHR021W-A	S. cerevisiae	YHR021W-A	chr8:149215	Non-essenti	ECM12	chr8	YHR021W-A	---	O13529	---	---	---	0007047 // c	0016020 // r	---	---	S000003531	This probe s	S000003531(11),YHR021W-A(11)
1778153_at	313.58	P	99.55	P	3.15	P	YHR022C	S. cerevisiae	YHR022C S	chr8:149565	---	---	chr8	YHR022C	---	P38763	---	---	---	0007264 // s	---	0000166 // r	---	---	This probe s	S000001064(11),GENSCAN00000003864(11),YHR022C(11)
1772139_at	4491.35	P	1744.2	P	2.575	P	YHR030C	S. cerevisiae	YHR030C S	chr8:168880	Serine/threo	SLT2	chr8	YHR030C	---	Q00772	---	---	---	0001101 // r	0005634 // r	0000166 // r	IPR001245 ,	---	This probe s	S000001072(11),GENEFINDER00000000665(10),GENSCAN00000003747(11),YHR030C(11)
1778668_at	390.11	P	162.13	P	2.4062	P	YHR044C	S. cerevisiae	YHR044C S	chr8:194058	2-deoxygluc	DOG1	chr8	YHR044C	---	P38774	---	---	---	0008152 // r	---	0003824 // c	---	---	This probe s	S000001086(11),GENSCAN00000003975(11),YHR044C(11)
1779904_at	409.62	P	124.33	P	3.2946	P	YHR048W	S. cerevisiae	YHR048W S	chr8:204597	Presumed a	YHK8	chr8	YHR048W	---	P38776	---	---	---	0006810 // t	0016020 // r	0015238 // c	---	S000001090	This probe s	S000001090(11),GENSCAN00000003770(11),YHR048W(11)
1770925_at	13210.86	P	4937.89	P	2.6754	P	YHR049W	S. cerevisiae	YHR049W S	chr8:206452	Putative seri	FSH1	chr8	YHR049W	---	P38777	---	---	---	---	0005634 // r	0004091 // c	---	---	This probe s	S000001091(11),GENEFINDER00000000570(11),GENSCAN00000003746(11),YHR049W(11)
1778501_at	1336.07	P	610.08	P	2.19	P	YHR075C	S. cerevisiae	YHR075C S	chr8:248439	Protein with	PPE1	chr8	YHR075C	---	P38796	---	---	---	0006464 // r	0005625 // s	0004091 // c	---	---	This probe s	S000001117(11),GENSCAN00000003787(11),YHR075C(11)
1776820_at	1548.15	P	501.25	P	3.0886	P	YHR080C	S. cerevisiae	YHR080C S	chr8:262801	---	---	chr8	YHR080C	---	P38800	---	---	---	---	0005739 // r	0005515 // r	---	---	This probe s	S000001122(11),GENEFINDER00000000534(11),GENSCAN00000003870(11),YHR080C(11)
1770302_at	10192.37	P	3104.94	P	3.2826	P	YHR087W	S. cerevisiae	YHR087W S	chr8:280820	Protein of ur	RTC3	chr8	YHR087W	---	P38804	---	---	---	0016070 // R	0005634 // r	---	---	---	This probe s	S000001129(11),GENEFINDER00000000573(11),YHR087W(11)
1776721_at	4097.43	P	798.04	P	5.1344	P	YHR094C	S. cerevisiae	YHR094C S	chr8:290914	Low-affinity	HXT1	chr8	YHR094C	---	P32465	---	---	---	0006810 // t	0005624 // r	0005215 // t	---	S000001136	This probe s	S000001136(11),GENEFINDER00000000541(11),GENSCAN00000003837(11),YHR094C(11)
1778666_at	526.85	P	138.38	P	3.8073	P	YHR096C	S. cerevisiae	YHR096C S	chr8:294670	Hexose tran	HXT5	chr8	YHR096C	---	P38695	---	---	---	0006810 // t	0005624 // r	0005215 // t	---	S000001138	This probe s	S000001138(11),GENEFINDER00000000637(11),GENSCAN00000003998(11),YHR096C(11)
1779275_at	11393.11	P	3443.16	P	3.3089	P	YHR104W	S. cerevisiae	YHR104W S	chr8:323410	Aldose redu	GRE3	chr8	YHR104W	---	P38715	---	---	---	0006950 // r	0005634 // r	0004032 // a	---	---	This probe s	S000001146(11),GENSCAN00000003807(11),GENEFINDER00000000639(11),YHR104W(11)
1776805_at	2330.44	P	999.36	P	2.3319	P	YHR106W	S. cerevisiae	YHR106W S	chr8:325599	Mitochondria	TRR2	chr8	YHR106W	---	P38816	---	---	---	0006979 // r	0005737 // c	0004791 // t	---	---	This probe s	S000001148(11),GENEFINDER00000000552(11),GENSCAN00000003931(11),YHR106W(11)
1775219_at	6606.98	P	1425.87	P	4.6336	P	YHR112C	S. cerevisiae	YHR112C S	chr8:334528	---	---	chr8	YHR112C	---	P38716	---	---	---	0006520 // a	0005737 // c	0003824 // c	---	---	This probe s	S000001154(11),GENSCAN00000003814(11),YHR112C(11)
1769796_at	184.66	P	89.09	P	2.0727	P	YHR124W	S. cerevisiae	YHR124W S	chr8:356562	Meiosis-spe	NDT80	chr8	YHR124W	---	P38830	---	---	---	0006350 // t	0000228 // r	0003677 // [	---	---	This probe s	S000001166(11),GENSCAN00000003963(11),YHR124W(11)
1775421_at	5235.56	P	1876.17	P	2.7906	P	YHR138C	S. cerevisiae	YHR138C S	chr8:377355	---	---	chr8	YHR138C	---	P38841	---	---	---	0034605 // c	---	0004866 // e	---	S000001180	This probe s	S000001180(11),GENSCAN00000003895(9),YHR138C(11)
1771373_at	1052.44	P	77.43	P	13.5921	P	YHR139C	S. cerevisiae	YHR139C S	chr8:378219	Protein regu	SPS100	chr8	YHR139C	---	P13130	---	---	---	0006520 // a	---	---	---	---	This probe s	S000001181(11),GENSCAN00000003817(11),YHR139C(11)
1771364_at	1558.55	P	222.35	P	7.0094	P	YHR140W	S. cerevisiae	YHR140W S	chr8:380572	---	---	chr8	YHR140W	---	P38842	---	---	---	---	0005783 // e	---	---	S000001182	This probe s	S000001182(11),YHR140W(11)
1770182_at	340.31	P	135.59	P	2.5098	P	YHR157W	S. cerevisiae	YHR157W S	chr8:412907	Protein invol	REC104	chr8	YHR157W	---	P33323	---	---	---	0006350 // t	0000794 // c	---	---	---	This probe s	S000001200(11),GENSCAN00000003982(11),YHR157W(11)
1773454_at	986.42	P	471.08	P	2.094	P	YHR171W	S. cerevisiae	YHR171W S	chr8:445710	Autophagy-r	ATG7	chr8	YHR171W	---	P38862	---	---	---	0006501 // c	0000407 // r	0003824 // c	---	---	This probe s	S000001214(11),GENSCAN00000004005(11),GENEFINDER00000000652(9),YHR171W(11)
1772738_at	2191.88	P	446.54	P	4.9086	P	YHR176W	S. cerevisiae	YHR176W S	chr8:454226	Flavin-conta	FMO1	chr8	YHR176W	---	P38866	---	---	---	0006457 // r	0005789 // e	0003824 // c	IPR013027 ,	---	This probe s	S000001219(11),GENEFINDER00000000579(11),GENSCAN00000003788(11),YHR176W(11)
1775114_at	92.11	P	40.29	A	2.2862	P	YHR177W	S. cerevisiae	YHR177W S	chr8:456586	---	---	chr8	YHR177W	---	P38867	---	---	---	---	---	0005515 // r	---	---	This probe s	S000001220(11),GENSCAN00000003751(11),GENEFINDER00000000531(11),YHR177W(11)
1773739_at	1686.41	P	829.03	P	2.0342	P	YHR195W	S. cerevisiae	YHR195W S	chr8:490742	Nuclear enve	NVJ1	chr8	YHR195W	---	P38881	---	---	---	0006914 // a	0005634 // r	0005515 // r	---	---	This probe s	S000001238(11),GENSCAN00000003856(11),YHR195W(11)
1779601_at	5525.57	P	2410.01	P	2.2928	P	YHR199C	S. cerevisiae	YHR199C S	chr8:497485	Putative pro	AIM46	chr8	YHR199C	---	P38885	---	---	---	0009813 // f	0005739 // r	0005515 // r	---	---	This probe s	S000001242(11),GENEFINDER00000000669(11),GENSCAN00000003765(11),YHR199C(11)
1772935_at	290.51	P	99.21	P	2.9282	P	YHR199C-A	S. cerevisiae	YHR199C-A	chr8:498566	Subunit of th	NBL1	chr8	YHR199C-A	---	Q3E7Y6	---	---	---	0007059 // c	0032133 // c	0030674 // r	---	---	This probe s	S000029704(11),YHR199C-A(11)
1771963_at	1374.73	P	214.76	P	6.4012	P	YHR209W	S. cerevisiae	YHR209W S	chr8:519432	Putative S-a	CRG1	chr8	YHR209W	---	P38892 /// C	---	---	---	0008152 // r	---	0008168 // r	---	---	This probe s	S000001252(11),GENSCAN00000003881(11),YHR209W(11)
1777802_at	343.92	P	138.15	P	2.4895	P	YHR210C	S. cerevisiae	YHR210C S	chr8:520707	---	---	chr8	YHR210C	---	P38893	---	---	---	0005975 // c	---	0003824 // c	---	---	This probe s	S000001253(11),GENEFINDER00000000626(11),GENSCAN00000003769(11),YHR210C(11)
1775391_s_at	361.05	P	113.76	P	3.1738	P	YIL172C	S. cerevisiae	YIL172C S1	chr15:22523	Protein of ur	FSP2	chr10 /// chr	YIL172C ///	---	P40439 /// C	---	---	---	0000023 // r	0005737 // c	0003824 // c	---	---	This probe s	S000001434(11),S000003757(11),S000005517(11),GENEFINDER00000000457(11),GENSCAN00000003449(11),GENEFINDER00000001271(11),GENSCAN00000004309(11),GENEFINDER00000002027(11),GENSCAN00000006795(11),YIL172C(11),YJL221C(11),YOL157C(11)
1779400_at	3405.3	P	855.06	P	3.9825	P	YIL165C	S. cerevisiae	YIL165C S1	chr9:33717-	Nitrilase, me	NIT1	chr9	YIL165C	---	P40446 /// P	---	---	---	0006807 // r	---	0016810 // h	---	---	This probe s	S000001427(11),X66132(10),GENEFINDER00000000404(9),GENSCAN00000003489(9),YIL165C(11)
1769342_at	81.95	P	19.44	P	4.2155	P	YIL160C	S. cerevisiae	YIL160C S1	chr9:40190-	3-ketoacyl-C	POT1	chr9	YIL160C	---	P27796	---	---	---	0006629 // l	0005777 // r	0003824 // c	---	---	This probe s	S000001422(11),GENEFINDER00000000389(11),GENSCAN00000003402(11),YIL160C(11)
1774613_at	1986.35	P	858.08	P	2.3149	P	YIL154C	S. cerevisiae	YIL154C S1	chr9:53980-	Transcriptio	IMP2'	chr9	YIL154C	---	P32351	---	---	---	0005975 // c	0005737 // c	0003713 // t	---	---	This probe s	S000001416(11),GENEFINDER00000000439(11),GENSCAN00000003339(11),YIL154C(11)
1775925_at	3579.13	P	1034.07	P	3.4612	P	YIL136W	S. cerevisiae	YIL136W S1	chr9:93618-	Protein of ur	OM45	chr9	YIL136W	---	P16547	---	---	---	---	0005739 // r	---	---	S000001398	This probe s	S000001398(11),GENEFINDER00000000422(11),GENSCAN00000003608(11),YIL136W(11)
1770981_at	6823.36	P	2943.97	P	2.3177	P	YIL121W	S. cerevisiae	YIL121W S1	chr9:132240	Multidrug tra	QDR2	chr9	YIL121W	---	P40474	---	---	---	0006810 // t	0005887 // i	0005515 // r	---	S000001383	This probe s	S000001383(11),GENEFINDER00000000436(11),GENSCAN00000003518(11),YIL121W(11)
1779976_at	1459.61	P	524.36	P	2.7836	P	YIL120W	S. cerevisiae	YIL120W S1	chr9:134413	Multidrug tra	QDR1	chr9	YIL120W	---	P40475	---	---	---	0006810 // t	0005886 // r	0015559 // r	IPR005828 ,	S000001382	This probe s	S000001382(11),GENSCAN00000003614(11),YIL120W(11)
1774545_at	2140.57	P	379.95	P	5.6338	P	YIL113W	S. cerevisiae	YIL113W S1	chr9:150555	Stress-induc	SDP1	chr9	YIL113W	---	P40479	---	---	---	0000196 // M	0005634 // r	0004721 // r	---	---	This probe s	S000001375(11),GENSCAN00000003338(11),YIL113W(11)
1774011_at	3040.41	P	1068.3	P	2.846	P	YIL107C	S. cerevisiae	YIL107C S1	chr9:163274	6-phosphofr	PFK26	chr9	YIL107C	---	P40433	---	---	---	0006000 // f	---	0000166 // r	---	---	This probe s	S000001369(11),GENEFINDER00000000473(11),GENSCAN00000003383(11),YIL107C(11)
1777690_at	1035.76	P	160.98	P	6.4341	P	YIL101C	S. cerevisiae	YIL101C.S1	chr9:175303	Transcriptio	XBP1	chr9	YIL101C	---	P40489	---	---	---	0006350 // t	0005634 // r	0003677 // [	---	---	This probe s	S000001363(11),GENEFINDER00000000511(11),GENSCAN00000003540(11),YIL101C(11)
1775874_at	1045.86	P	145.02	P	7.2118	P	YIL099W	S. cerevisiae	YIL099W S1	chr9:178000	Intracellular	SGA1	chr9	YIL099W	---	P08019	---	---	---	0000272 // r	0000324 // f	0003824 // c	---	S000001361	This probe s	S000001361(11),GENEFINDER00000000464(11),GENSCAN00000003386(11),YIL099W(11)
1773792_at	1405.36	P	474.85	P	2.9596	P	YIL097W	S. cerevisiae	YIL097W S1	chr9:180423	Protein of ur	FYV10	chr9	YIL097W	---	P40492	---	---	---	0006916 // a	0005634 // r	0005515 // r	---	---	This probe s	S000001359(11),GENSCAN00000003406(11),YIL097W(11)
1778263_at	5322.48	P	1303.62	P	4.0828	P	YIL087C	S. cerevisiae	YIL087C S1	chr9:199642	Putative pro	AIM19	chr9	YIL087C	---	P40502	---	---	---	---	0005739 // r	---	---	---	This probe s	S000001349(11),YIL087C(11)
1775892_at	1871.62	P	598.96	P	3.1248	P	YIL077C	S. cerevisiae	YIL077C S1	chr9:214987	---	---	chr9	YIL077C	---	P40508	---	---	---	0042493 // r	0005739 // r	---	---	S000001339	This probe s	S000001339(11),GENSCAN00000003334(11),YIL077C(11)
1778823_at	280.66	P	98.29	P	2.8554	P	YIL072W	S. cerevisiae	YIL072W S1	chr9:226598	Meiosis-spe	HOP1	chr9	YIL072W	---	P20050	---	---	---	0007067 // r	0000794 // c	0000400 // f	---	---	This probe s	S000001334(11),GENSCAN00000003342(11),YIL072W(11)
1777336_at	2146.12	P	987.61	P	2.173	P	YIL065C	S. cerevisiae	YIL065C S1	chr9:241304	Protein invol	FIS1	chr9	YIL065C	---	P40515	---	---	---	0000266 // r	0005739 // r	0005488 // b	---	S000001327	This probe s	S000001327(11),GENEFINDER00000000484(11),GENSCAN00000003609(11),YIL065C(11)
1779583_at	626.17	P	156.36	P	4.0047	P	YIL060W	S. cerevisiae	YIL060W.S1	chr9:246388	---	---	chr9	YIL060W	---	P40519	---	---	---	---	0016020 // r	---	---	---	This probe s	S000001322(11),YIL060W(11)
1776054_at	2949.06	P	1304.09	P	2.2614	P	YIL056W	S. cerevisiae	YIL056W S1	chr9:249988	Transcriptio	VHR1	chr9	YIL056W	---	P40522	---	---	---	0006357 // r	0005634 // r	0003677 // [	---	---	This probe s	S000001318(11),GENEFINDER00000000401(11),GENSCAN00000003376(11),YIL056W(11)
1773936_at	851.39	P	270.95	P	3.1422	P	YIL055C	S. cerevisiae	YIL055C S1	chr9:252039	---	---	chr9	YIL055C	---	P40523	---	---	---	---	---	---	---	---	This probe s	S000001317(11),GENEFINDER00000000410(11),GENSCAN00000003328(11),YIL055C(11)
1776174_at	4353.13	P	1313.75	P	3.3135	P	YIL050W	S. cerevisiae	YIL050W S1	chr9:258911	Pho85p cycl	PCL7	chr9	YIL050W	---	P40186	---	---	---	0000079 // r	0000307 // c	0005515 // r	---	---	This probe s	S000001312(11),GENEFINDER00000000400(11),GENSCAN00000003601(11),YIL050W(11)
1777366_at	2126.7	P	780.34	P	2.7254	P	YIL045W	S. cerevisiae	YIL045W S1	chr9:271159	Putative type	PIG2	chr9	YIL045W	---	P40187 /// C	---	---	---	0005979 // r	0005737 // c	0005515 // r	---	---	This probe s	S000001307(11),GENSCAN00000003340(11),YIL045W(11)
1772007_at	1079.14	P	274.2	P	3.9356	P	YIL029C	S. cerevisiae	YIL029C S1	chr9:300826	---	---	chr9	YIL029C	---	P40538	---	---	---	0042493 // r	0016020 // r	---	---	S000001291	This probe s	S000001291(11),YIL029C(11)
1775401_at	895.57	P	381.47	P	2.3477	P	YIL024C	S. cerevisiae	YIL024C S1	chr9:308599	---	---	chr9	YIL024C	---	P40543	---	---	---	---	---	---	---	---	This probe s	S000001286(11),GENEFINDER00000000407(11),GENSCAN00000003420(11),YIL024C(11)
1772670_at	1022.56	P	468.3	P	2.1836	P	YIL017C	S. cerevisiae	YIL017C.S1	chr9:318197	Protein invol	VID28	chr9	YIL017C	---	P40547	---	---	---	0007039 // v	0005634 // r	0005488 // b	---	---	This probe s	S000001279(11),GENEFINDER00000000420(11),GENSCAN00000003586(11),YIL017C(11)
1771944_at	448.86	P	139.68	P	3.2135	P	YIL013C	S. cerevisiae	YIL013C S1	chr9:328204	ATP-binding	PDR11	chr9	YIL013C	---	P40550	---	---	---	0006810 // t	0016020 // r	0000166 // r	---	S000001275	This probe s	S000001275(11),GENEFINDER00000000446(11),GENSCAN00000003581(11),YIL013C(11)
1777842_at	1298.04	P	633.46	P	2.0491	P	YIL007C	S. cerevisiae	YIL007C S1	chr9:342990	Protein with	NAS2	chr9	YIL007C	---	P40555	---	---	---	---	0005737 // c	0005515 // r	---	---	This probe s	S000001269(11),GENSCAN00000003388(11),YIL007C(11)
1773698_at	3509.02	P	1663.12	P	2.1099	P	YIR016W	S. cerevisiae	YIR016W S	chr9:382624	---	---	chr9	YIR016W	---	P40572	---	---	---	---	---	0000166 // r	---	---	This probe s	S000001455(11),GENEFINDER00000000450(11),GENSCAN00000003352(11),YIR016W(11)
1780163_at	1395.54	P	121.14	P	11.5201	P	YIR017C	S. cerevisiae	YIR017C S1	chr9:383552	Basic leucine	MET28	chr9	YIR017C	---	P40573	---	---	---	0000097 // s	0005634 // r	0003677 // [	---	---	This probe s	S000001456(11),GENSCAN00000003597(11),YIR017C(11)
1774717_at	672.73	P	314.22	P	2.141	P	YIR018W	S. cerevisiae	YIR018W S	chr9:384605	Basic leucine	YAP5	chr9	YIR018W	---	P40574	---	---	---	0006350 // t	0000790 // r	0003677 // [	---	---	This probe s	S000001457(11),GENEFINDER00000000437(11),GENSCAN00000003434(11),YIR018W(11)
1773183_at	548.48	P	245.07	P	2.2381	P	YIR019C	S. cerevisiae	YIR019C S1	chr9:389568	GPI-anchore	MUC1	chr9	YIR019C	---	P08640	---	---	---	0001403 // i	0005886 // r	---	---	S000001458	This probe s	S000001458(11),GENEFINDER00000000443(11),YIR019C(11)
1774321_at	316.64	P	131.11	P	2.4151	P	YIR032C	S. cerevisiae	YIR032C.S1	chr9:415026	Ureidoglycol	DAL3	chr9	YIR032C	---	P32459	---	---	---	0000256 // a	0005886 // r	0004848 // u	---	---	This probe s	S000001471(11),GENSCAN00000003451(11),YIR032C(11)
1776749_at	6545.24	P	2773.66	P	2.3598	P	YIR038C	S. cerevisiae	YIR038C S1	chr9:423805	ER associat	GTT1	chr9	YIR038C	---	P40582	---	---	---	0006749 // c	0005739 // r	0004364 // c	---	---	This probe s	S000001477(11),GENEFINDER00000000503(11),GENSCAN00000003439(11),YIR038C(11)
1769659_at	3826.69	P	276.78	P	13.8257	P	YIR039C	S. cerevisiae	YIR039C S1	chr9:430493	Putative GP	YPS6	chr9	YIR039C	---	P40583 /// C	---	---	---	0006508 // r	0005886 // r	0004190 // a	---	---	This probe s	S000001478(11),GENEFINDER00000000403(10),GENSCAN00000003584(11),YIR039C(11)
1778832_at	797.35	P	228.69	P	3.4866	P	YIR042C	S. cerevisiae	YIR042C S1	chr9:435269	---	---	chr9	YIR042C	---	P40586	---	---	---	0008152 // r	---	0008080 // N	---	---	This probe s	S000001481(11),GENSCAN00000003468(11),YIR042C(11)
1778617_at	423.98	P	130.67	P	3.2447	P	YJL216C	S. cerevisiae	YJL216C S1	chr10:24340	---	---	chr10	YJL216C	---	P40884	---	---	---	0000023 // r	---	0003824 // c	---	---	This probe s	S000003752(11),GENEFINDER00000001288(11),GENSCAN00000004784(11),YJL216C(11)
1775215_at	1033.93	P	320.06	P	3.2304	P	YJL213W	S. cerevisiae	YJL213W S	chr10:32162	---	---	chr10	YJL213W	---	P40896	---	---	---	0044237 // c	0005840 // r	0016787 // h	---	GENEFINDE	This probe s	S000003749(11),GENEFINDER00000001244(11),GENSCAN00000004182(11),YJL213W(11)
1773613_at	3520.34	P	1224	P	2.8761	P	YJL212C	S. cerevisiae	YJL212C S1	chr10:33849	Proton-coup	OPT1	chr10	YJL212C	---	P40897	---	---	---	0006790 // s	0005783 // e	0005427 // r	---	S000003748	This probe s	S000003748(11),GENEFINDER00000001199(11),GENSCAN00000004383(11),YJL212C(11)
1775797_at	5227.17	P	1476.46	P	3.5403	P	YJL210W	S. cerevisiae	YJL210W S	chr10:36918	RING-finger	PEX2	chr10	YJL210W	---	P32800	---	---	---	0007031 // r	0005777 // r	0005515 // r	IPR006845 ,	---	This probe s	S000003746(11),GENSCAN00000004771(11),YJL210W(11)
1772973_at	417.99	P	169.28	P	2.4692	P	YJL206C	S. cerevisiae	YJL206C S1	chr10:47658	---	---	chr10	YJL206C	---	P39529	---	---	---	0006350 // t	0005634 // r	0003677 // [	---	---	This probe s	S000003741(11),GENSCAN00000004569(11),YJL206C(11)
1770240_at	3478.03	P	1602.68	P	2.1701	P	YJL205C	S. cerevisiae	YJL205C S1	chr10:50138	Protein of ur	NCE101	chr10	YJL205C	---	Q02820	---	NP_012330	---	0006810 // t	---	---	---	S000003742	This probe s	U41658(11)
1769460_at	922.36	P	424.99	P	2.1703	P	YJL185C	S. cerevisiae	YJL185C S1	chr10:82092	---	---	chr10	YJL185C	---	P46983	---	---	---	---	---	0005515 // r	---	---	This probe s	S000003721(11),GENSCAN00000004179(11),YJL185C(11)
1769514_at	4025.84	P	1684.97	P	2.3893	P	YJL165C	S. cerevisiae	YJL165C S1	chr10:10688	Putative pro	HAL5	chr10	YJL165C	---	P38970	---	---	---	0006468 // r	---	0000166 // r	IPR001245 ,	---	This probe s	S000003701(11),U15300(11),GENSCAN00000004461(11),YJL165C(11)
1771925_at	4645.68	P	1669.86	P	2.7821	P	YJL164C	S. cerevisiae	YJL164C S1	chr10:10995	cAMP-depen	TPK1	chr10	YJL164C	---	P06244	---	---	---	0006468 // r	0005634 // r	0000166 // r	IPR001245 ,	---	This probe s	S000003700(11),GENEFINDER00000001268(11),GENSCAN00000004298(11),YJL164C(11)
1772692_at	1460.12	P	450.44	P	3.2415	P	YJL163C	S. cerevisiae	YJL163C S1	chr10:11165	---	---	chr10	YJL163C	---	P46996	---	---	---	---	0016020 // r	---	---	S000003699	This probe s	S000003699(11),GENSCAN00000004679(11),YJL163C(11)
1774435_at	2902.33	P	402.5	P	7.2108	P	YJL161W	S. cerevisiae	YJL161W S	chr10:11723	Putative pro	FMP33	chr10	YJL161W	---	P46998	---	---	---	---	0005739 // r	---	---	---	This probe s	S000003697(11),YJL161W(11)
1778914_at	439.62	P	208.09	P	2.1126	P	YJL160C	S. cerevisiae	YJL160C S1	chr10:11795	---	---	chr10	YJL160C	---	P46999	---	---	---	---	0005576 // e	---	---	---	This probe s	S000003696(11),GENEFINDER00000001195(11),GENSCAN00000004687(11),YJL160C(11)
1774340_at	2403.14	P	896.91	P	2.6794	P	YJL155C	S. cerevisiae	YJL155C S1	chr10:12898	Fructose-2,6	FBP26	chr10	YJL155C	---	P32604	---	---	---	0006000 // f	---	0000166 // r	---	---	This probe s	S000003691(11),GENEFINDER00000001217(10),GENSCAN00000004161(11),YJL155C(11)
1779129_at	844.31	P	172.95	P	4.8818	P	YJL153C	S. cerevisiae	YJL153C S1	chr10:13402	Inositol 1-ph	INO1	chr10	YJL153C	---	P11986	---	---	---	0006021 // i	0005737 // c	0003824 // c	---	---	This probe s	S000003689(11),GENEFINDER00000001267(11),YJL153C(11)
1771951_at	2086.19	P	582.3	P	3.5827	P	YJL144W	S. cerevisiae	YJL144W S	chr10:14605	---	---	chr10	YJL144W	---	P47009	---	---	---	0006970 // r	0005737 // c	---	---	---	This probe s	S000003680(11),YJL144W(11)
1773607_at	5908.87	P	2598.2	P	2.2742	P	YJL141C	S. cerevisiae	YJL141C S1	chr10:14766	Serine-threo	YAK1	chr10	YJL141C	---	P14680	---	---	---	0006468 // r	0005634 // r	0000166 // r	---	---	This probe s	S000003677(11),GENEFINDER00000001243(11),GENSCAN00000004377(11),YJL141C(11)
1779784_at	3658.24	P	1752.24	P	2.0878	P	YJL133C-A	S. cerevisiae	YJL133C-A	chr10:15932	---	---	chr10	YJL133C-A	---	Q3E7A3	---	---	---	---	0005739 // r	---	---	---	This probe s	S000028805(11),YJL133C-A(11)
1775363_at	810.34	P	284.99	P	2.8434	P	YJL132W	S. cerevisiae	YJL132W S	chr10:16161	---	---	chr10	YJL132W	---	P47014	---	---	---	---	0005624 // r	---	---	S000003668	This probe s	S000003668(11),GENEFINDER00000001236(11),GENSCAN00000004149(11),YJL132W(11)
1780101_at	965.83	P	192.59	P	5.015	P	YJL106W	S. cerevisiae	YJL106W S	chr10:22108	Serine/threo	IME2	chr10	YJL106W	---	P32581	---	---	---	0006468 // r	0005634 // r	0000166 // r	IPR001245 ,	---	This probe s	S000003642(11),GENSCAN00000004185(11),YJL106W(11)
1777080_at	6643.98	P	2019.32	P	3.2902	P	YJL101C	S. cerevisiae	YJL101C S1	chr10:23401	Gamma glut	GSH1	chr10	YJL101C	---	A6N9K8 /// F	---	---	---	0006750 // c	0005622 // i	0004357 // c	---	---	This probe s	S000003637(11),GENSCAN00000004646(11),YJL101C(11)
1770534_at	1781.27	P	433.31	P	4.1108	P	YJL100W	S. cerevisiae	YJL100W S	chr10:23695	Type II phos	LSB6	chr10	YJL100W	---	P42951	---	---	---	0046854 // r	0000329 // f	0000287 // r	---	---	This probe s	S000003636(11),GENEFINDER00000001149(11),GENSCAN00000004414(11),YJL100W(11)
1770592_at	150.45	P	62.3	P	2.4149	P	YJL089W	S. cerevisiae	YJL089W S	chr10:26562	C6 zinc clust	SIP4	chr10	YJL089W	---	P46954	---	---	---	0001403 // i	0005634 // r	0003677 // [	---	---	This probe s	S000003625(11),GENSCAN00000004659(11),YJL089W(11)
1769951_at	3730.88	P	1209.62	P	3.0843	P	YJL082W	S. cerevisiae	YJL082W S	chr10:28088	Protein of ur	IML2	chr10	YJL082W	---	P47031	---	---	---	---	0005634 // r	0005515 // r	---	---	This probe s	S000003618(11),GENEFINDER00000001259(11),GENSCAN00000004198(11),YJL082W(11)
1774985_at	1206.14	P	456.08	P	2.6446	P	YJL070C	S. cerevisiae	YJL070C S1	chr10:30766	---	---	chr10	YJL070C	---	P40361	---	---	---	0006144 // r	0005737 // c	0005515 // r	---	---	This probe s	S000003606(11),GENEFINDER00000001194(11),GENSCAN00000004264(11),YJL070C(11)
1774485_at	3308.58	P	1043.98	P	3.1692	P	YJL066C	S. cerevisiae	YJL066C.S1	chr10:31380	Mitochondria	MPM1	chr10	YJL066C	---	P40364	---	---	---	---	0005624 // r	---	---	---	This probe s	S000003602(11),GENEFINDER00000001155(11),GENSCAN00000004141(11),YJL066C(11)
1775384_at	8215.65	P	3257.8	P	2.5218	P	YJL060W	S. cerevisiae	YJL060W S	chr10:32308	Kynurenine a	BNA3	chr10	YJL060W	---	P47039	---	---	---	0009058 // b	0005737 // c	0003824 // c	---	---	This probe s	S000003596(11),GENSCAN00000004680(11),GENEFINDER00000001212(11),YJL060W(11)
1779079_at	2391.06	P	1043.04	P	2.2924	P	YJL059W	S. cerevisiae	YJL059W S	chr10:32465	Vacuolar me	YHC3	chr10	YJL059W	---	P47040	---	---	---	0006810 // t	0000324 // f	---	---	S000003595	This probe s	S000003595(11),YJL059W(11)
1772974_at	2034.81	P	624.63	P	3.2576	P	YJL057C	S. cerevisiae	YJL057C S1	chr10:32781	Putative seri	IKS1	chr10	YJL057C	---	P47042	---	---	---	0006468 // r	---	0000166 // r	IPR001245 ,	---	This probe s	S000003593(11),GENSCAN00000004245(11),YJL057C(11)
1772371_at	3719.82	P	749.46	P	4.9633	P	YJL048C	S. cerevisiae	YJL048C.S1	chr10:34714	UBX (ubiquit	UBX6	chr10	YJL048C	---	P47049	---	---	---	0006511 // u	0005634 // r	0005515 // r	---	---	This probe s	S000003584(11),GENEFINDER00000001335(11),GENSCAN00000004411(11),YJL048C(11)
1774888_at	468.16	P	56.57	P	8.2758	P	YJL045W	S. cerevisiae	YJL045W S	chr10:35571	---	---	chr10	YJL045W	---	P47052	---	---	---	0006099 // t	0005739 // r	0008177 // s	IPR002218 ,	---	This probe s	S000003581(11),GENSCAN00000004579(11),GENEFINDER00000001221(11),YJL045W(11)
1770120_at	6480.05	P	2458.59	P	2.6357	P	YJL016W	S. cerevisiae	YJL016W S	chr10:40528	---	---	chr10	YJL016W	---	P47072	---	---	---	---	0005737 // c	0005515 // r	---	---	This probe s	S000003553(11),GENSCAN00000004678(11),YJL016W(11)
1772850_at	5210.1	P	866.43	P	6.0133	P	YJR008W	S. cerevisiae	YJR008W.S	chr10:45212	---	---	chr10	YJR008W	---	P47085	---	---	---	---	0005634 // r	0005515 // r	---	---	This probe s	S000003768(11),GENSCAN00000004573(11),YJR008W(11)
1773498_at	3964.47	P	402.16	P	9.8579	P	YJR010W	S. cerevisiae	YJR010W S	chr10:45593	ATP sulfuryl	MET3	chr10	YJR010W	---	P08536	---	---	---	0000103 // s	0005737 // c	0000166 // r	---	---	This probe s	S000003771(11),GENEFINDER00000001278(11),GENSCAN00000004398(11),YJR010W(11)
1772228_at	1263.17	P	273.5	P	4.6185	P	YJR039W	S. cerevisiae	YJR039W S	chr10:50362	---	---	chr10	YJR039W	---	P47107	---	---	---	---	0005739 // r	---	---	---	This probe s	S000003800(11),GENEFINDER00000001178(11),GENSCAN00000004205(11),YJR039W(11)
1779001_at	1477.29	P	686.4	P	2.1522	P	YJR052W	S. cerevisiae	YJR052W S	chr10:53175	Protein that	RAD7	chr10	YJR052W	---	P06779	---	---	---	0000715 // r	0000108 // r	0003684 // c	---	---	This probe s	S000003813(11),GENEFINDER00000001311(11),GENSCAN00000004417(11),YJR052W(11)
1772327_at	2894.58	P	1408.7	P	2.0548	P	YJR059W	S. cerevisiae	YJR059W S	chr10:54548	Putative seri	PTK2	chr10	YJR059W	---	P47116 /// C	---	---	---	0000082 // c	0005634 // r	0000166 // r	IPR001245 ,	---	This probe s	S000003820(11),GENSCAN00000004581(11),GENEFINDER00000001294(11),YJR059W(11)

1773329_at	9777.14	P	3109.77	P	3.144	P	YJR073C	S. cerevisiae	YJR073C S	chr10:5720	Phospholipic	OPI3	chr10	YJR073C	---	P05375	---	---	---	0006644 // r	0005739 // r	0004481 // r	---	---	This probe s	S000003834(11),GENEFINDER00000001237(11),GENSCAN00000004328(11),YJR073C(11)
1772566_at	528.05	P	227.75	P	2.3186	P	YJR094C	S. cerevisiae	YJR094C S	chr10:60426	Master regu	IME1	chr10	YJR094C	---	P21190	---	---	---	0006350 // t	0005634 // r	0005515 // r	---	---	This probe s	S000003854(11),GENEFINDER00000001183(11),GENSCAN00000004629(11),YJR094C(11)
1778931_at	8163.81	P	1940.35	P	4.2074	P	YJR096W	S. cerevisiae	YJR096W S	chr10:61089	---	---	chr10	YJR096W	---	P47137	---	---	---	0006979 // r	0005634 // r	0004032 // a	---	---	This probe s	S000003857(11),GENEFINDER00000001207(11),GENSCAN00000004694(11),YJR096W(11)
1779018_at	4193.66	P	2039.72	P	2.056	P	YJR103W	S. cerevisiae	YJR103W S	chr10:62044	Minor CTP s	URA8	chr10	YJR103W	---	P38627	---	---	---	0006221 // p	0005829 // c	0003824 // c	---	---	This probe s	S000003864(11),GENEFINDER00000001187(11),GENSCAN00000004676(11),YJR103W(11)
1774796_at	658.45	P	209.7	P	3.14	P	YJR108W	S. cerevisiae	YJR108W S	chr10:62840	Protein of ur	ABM1	chr10	YJR108W	---	P47146	---	---	---	0000226 // r	---	0005515 // r	---	---	This probe s	S000003869(11),YJR108W(11)
1777575_at	2195.48	P	793.04	P	2.7684	P	YJR115W	S. cerevisiae	YJR115W S	chr10:63963	---	---	chr10	YJR115W	---	P47152	---	---	---	---	---	---	---	---	This probe s	S000003876(11),GENSCAN00000004230(10),YJR115W(11)
1777867_at	6729.67	P	3115.33	P	2.1602	P	YJR127C	S. cerevisiae	YJR127C S	chr10:65861	Zinc-finger p	RSF2	chr10	YJR127C	---	P46974	---	---	---	0045449 // r	0005622 // i	0003676 // r	---	---	This probe s	S000003888(11),GENSCAN00000004737(11),YJR127C(11)
1775663_at	5427.11	P	735.62	P	7.3776	P	YJR137C	S. cerevisiae	YJR137C S	chr10:67865	Sulfite reduc	ECM17	chr10	YJR137C	---	A0SXK5 ///	---	---	---	0000103 // s	0005737 // c	0003824 // c	---	---	This probe s	S000003898(11),GENEFINDER00000001192(11),GENSCAN00000004194(11),YJR137C(11)
1772626_at	988.3	P	319.43	P	3.0939	P	YJR149W	S. cerevisiae	YJR149W S	chr10:70685	---	---	chr10	YJR149W	---	P47177	---	---	---	0008152 // r	0005737 // c	0003824 // c	---	---	This probe s	S000003910(11),GENEFINDER00000001235(11),GENSCAN00000004389(11),YJR149W(11)
1771702_at	899.76	P	356.4	P	2.5246	P	YJR152W	S. cerevisiae	YJR152W S	chr10:71936	Allantoin per	DAL5	chr10	YJR152W	---	P15365	---	---	---	0006810 // t	0005886 // p	0005215 // t	---	S000003913	This probe s	S000003913(11),GENSCAN00000004429(11),YJR152W(11)
1774749_at	1560.04	P	330.54	P	4.7197	P	YJR155W	S. cerevisiae	YJR155W S	chr10:72709	Putative aryl	AAD10	chr10	YJR155W	---	P47182	---	---	---	0009636 // r	---	0005515 // p	---	---	This probe s	S000003916(11),GENEFINDER00000001253(11),GENSCAN00000004431(11),YJR155W(11)
1778187_at	402.8	P	197.86	P	2.0358	P	YKL222C	S. cerevisiae	YKL222C S	chr11:3503-	---	---	chr11	YKL222C	---	P35995	---	---	---	0006350 // t	0005634 // r	0003677 // [	---	---	This probe s	S000001705(11),GENSCAN00000004623(11),YKL222C(11)
1779517_at	247.94	P	25.53	P	9.7117	P	YKL221W	S. cerevisiae	YKL221W S	chr11:6107-	Protein with	MCH2	chr11	YKL221W	---	P36032	---	---	---	0006810 // t	0016020 // r	0008028 // r	---	S000001704	This probe s	S000001704(11),GENSCAN00000004806(11),YKL221W(11)
1773370_at	3095.49	P	994.7	P	3.112	P	YKL201C	S. cerevisiae	YKL201C S	chr11:63926	Putative pos	MNN4	chr11	YKL201C	---	P36044	---	---	---	0006487 // p	0016020 // r	---	IPR001245 /	S000001684	This probe s	S000001684(11),GENEFINDER00000001615(11),GENSCAN00000004432(11),YKL201C(11)
1770378_at	508.98	P	147.39	P	3.4533	P	YKL188C	S. cerevisiae	YKL188C S	chr11:86225	Subunit of a	PXA2	chr11	YKL188C	---	P34230	---	---	---	0006810 // t	0005777 // r	0000166 // r	---	---	This probe s	S000001671(11),GENSCAN00000004268(11),YKL188C(11)
1777919_at	766.58	P	362.83	P	2.1128	P	YKL171W	S. cerevisiae	YKL171W S	chr11:12747	---	---	chr11	YKL171W	---	P36003	---	---	---	0006468 // r	0005737 // c	0000166 // r	---	---	This probe s	S000001654(11),GENEFINDER00000001606(11),GENSCAN00000004727(11),YKL171W(11)
1772229_at	11418.42	P	1031.59	P	11.0688	P	YKL163W	S. cerevisiae	YKL163W S	chr11:14440	O-glycosylat	PIR3	chr11	YKL163W	---	Q03180	---	---	---	0007047 // c	0005576 // e	0005199 // s	---	---	This probe s	S000001646(11),GENEFINDER00000001743(11),GENSCAN00000004793(11),YKL163W(11)
1770795_at	818.57	P	399.38	P	2.0496	P	YKL162C	S. cerevisiae	YKL162C S	chr11:14763	---	---	chr11	YKL162C	---	P36052	---	---	---	---	0005739 // r	---	---	---	This probe s	S000001645(11),GENSCAN00000004749(11),YKL162C(11)
1771580_at	525.19	P	178.84	P	2.9366	P	YKL161C	S. cerevisiae	YKL161C S	chr11:14938	---	---	chr11	YKL161C	---	P36005	---	---	---	0006468 // r	---	0000166 // r	IPR001245 /	---	This probe s	S000001644(11),GENSCAN00000004296(11),YKL161C(11)
1776204_at	6471.08	P	2043.28	P	3.167	P	YKL151C	S. cerevisiae	YKL151C S	chr11:16491	---	---	chr11	YKL151C	---	P36059 /// C	---	---	---	0006950 // r	0005737 // c	---	---	---	This probe s	S000001634(11),GENEFINDER00000001603(11),GENSCAN00000004358(11),YKL151C(11)
1778599_at	7372.43	P	3617.22	P	2.0381	P	YKL150W	S. cerevisiae	YKL150W S	chr11:16654	Mitochondria	MCR1	chr11	YKL150W	---	P36060	---	---	---	0006696 // e	0005739 // r	0004128 // c	---	S000001633	This probe s	S000001633(11),GENSCAN00000004686(11),GENEFINDER00000001867(11),YKL150W(11)
1776609_at	8356.68	P	3459.81	P	2.4154	P	YKL142W	S. cerevisiae	YKL142W S	chr11:17851	Protein of ur	MRP8	chr11	YKL142W	---	P35719	---	---	---	---	0005737 // c	0005515 // r	IPR001245 /	GENEFINDE	This probe s	S000001625(11),GENEFINDER00000001598(11),GENSCAN00000004752(11),YKL142W(11)
1772181_at	780.61	P	316.29	P	2.468	P	YKL133C	S. cerevisiae	YKL133C S	chr11:19167	---	---	chr11	YKL133C	---	P36066	---	---	---	---	---	0000166 // r	---	S000001616	This probe s	S000001616(11),GENSCAN00000004765(11),YKL133C(11)
1769938_at	1245.77	P	484	P	2.5739	P	YKL132C	S. cerevisiae	YKL132C S	chr11:19357	Putative dihy	RMA1	chr11	YKL132C	---	P36001	---	---	---	0006730 // c	0005739 // r	0000166 // r	---	---	This probe s	S000001615(11),GENSCAN00000004357(11),YKL132C(11)
1774355_at	1831.49	P	901.84	P	2.0308	P	YKL129C	S. cerevisiae	YKL129C S	chr11:19634	One of two t	MYO3	chr11	YKL129C	---	P36006	---	---	---	0006887 // e	0005737 // c	0000146 // r	IPR011511 /	---	This probe s	S000001612(11),GENEFINDER00000001736(11),GENSCAN00000004591(11),YKL129C(11)
1771189_at	1452.38	P	659.54	P	2.2021	P	YKL121W	S. cerevisiae	YKL121W S	chr11:21378	Protein of ur	DGR2	chr11	YKL121W	---	P32330	---	---	---	---	---	---	---	---	This probe s	S000001604(11),GENSCAN00000004804(11),YKL121W(11)
1776759_at	219.71	P	44	P	4.9934	P	YKL107W	S. cerevisiae	YKL107W S	chr11:23578	---	---	chr11	YKL107W	---	P34251	---	---	---	0006808 // r	0016020 // r	0003824 // c	IPR008030 /	---	This probe s	S000001590(11),GENEFINDER00000001662(10),GENSCAN00000004412(11),YKL107W(11)
1771153_at	6575.09	P	3149.31	P	2.0878	P	YKL103C	S. cerevisiae	YKL103C S	chr11:24577	Vacuolar am	LAP4	chr11	YKL103C	---	P14904	---	---	---	0006508 // p	0000324 // f	0004177 // a	---	---	This probe s	S000001586(11),GENEFINDER00000001619(11),GENSCAN00000004505(11),YKL103C(11)
1774633_at	6009.64	P	2923.18	P	2.0559	P	YKL094W	S. cerevisiae	YKL094W S	chr11:26298	Serine hydro	YJU3	chr11	YKL094W	---	P28321	---	---	---	---	0005737 // c	0005515 // r	---	---	This probe s	S000001577(11),GENEFINDER00000001594(11),GENSCAN00000004707(11),YKL094W(11)
1776576_at	1228.25	P	373.88	P	3.2851	P	YKL093W	S. cerevisiae	YKL093W S	chr11:26442	Protein invol	MBR1	chr11	YKL093W	---	P23493	---	---	---	0009060 // a	0005739 // r	---	---	---	This probe s	S000001576(11),GENEFINDER00000001680(11),GENSCAN00000004582(11),YKL093W(11)
1772023_at	7529.92	P	1377.56	P	5.4661	P	YKL091C	S. cerevisiae	YKL091C S	chr11:26935	---	---	chr11	YKL091C	---	P33324	---	---	---	0034605 // c	0005634 // r	---	---	---	This probe s	S000001574(11),GENEFINDER00000001818(11),GENSCAN00000004810(11),YKL091C(11)
1777723_at	3590.36	P	1426.39	P	2.5171	P	YKL087C	S. cerevisiae	YKL087C S	chr11:27682	Cytochrome	CYT2	chr11	YKL087C	---	Q00873	---	---	---	0018063 // c	0005739 // r	0004408 // h	---	---	This probe s	S000001570(11),GENEFINDER00000001779(11),GENSCAN00000004452(11),YKL087C(11)
1773938_at	7223.05	P	249.53	P	28.9466	P	YKL086W	S. cerevisiae	YKL086W S	chr11:27792	Sulfiredoxin,	SRX1	chr11	YKL086W	---	P36077	---	---	---	0006979 // r	0005634 // r	0000287 // r	---	---	This probe s	S000001569(11),YKL086W(11)
1776865_at	622.42	P	133.18	P	4.6735	P	YKL071W	S. cerevisiae	YKL071W S	chr11:30475	---	---	chr11	YKL071W	---	P36086	---	---	---	0008152 // r	0005737 // c	0003824 // c	IPR002198 /	---	This probe s	S000001554(11),GENSCAN00000004761(11),YKL071W(11)
1779207_at	3949.82	P	1518.15	P	2.6017	P	YKL068W-A	S. cerevisiae	YKL068W-A	chr11:30884	---	---	chr11	YKL068W-A	---	Q3E826	---	---	---	---	---	---	---	---	This probe s	S000028524(11),YKL068W-A(11)
1772255_at	7878.92	P	3806.83	P	2.0697	P	YKL067W	S. cerevisiae	YKL067W S	chr11:31445	Nucleoside d	YNK1	chr11	YKL067W	---	P36010	---	---	---	0006165 // r	0005737 // c	0000166 // r	IPR007230 /	---	This probe s	S000001550(11),GENEFINDER00000001676(11),GENSCAN00000004559(11),YKL067W(11)
1780038_at	8242.45	P	3690.61	P	2.2334	P	YKL065C	S. cerevisiae	YKL065C S	chr11:31607	Endoplasmic	YET1	chr11	YKL065C	---	P35723	---	---	---	0006810 // t	0005783 // e	0005515 // r	IPR008417 /	S000001548	This probe s	S000001548(11),GENSCAN00000004728(10),YKL065C(11)
1778658_at	3116.01	P	1491.61	P	2.089	P	YKL064W	S. cerevisiae	YKL064W S	chr11:31740	Putative mag	MNR2	chr11	YKL064W	---	P35724	---	---	---	0030001 // r	0016020 // r	0005515 // r	---	S000001547	This probe s	S000001547(11),GENEFINDER00000001593(11),GENSCAN00000004770(11),YKL064W(11)
1777138_at	2125.67	P	842.61	P	2.5227	P	YKL052C	S. cerevisiae	YKL052C S	chr11:33930	Essential su	ASK1	chr11	YKL052C	---	P35734	---	---	---	0007049 // c	0000777 // c	0005200 // s	---	---	This probe s	S000001535(11),GENEFINDER00000001878(11),GENSCAN00000004526(11),YKL052C(11)
1778769_at	5866.4	P	2439.55	P	2.4047	P	YKL051W	S. cerevisiae	YKL051W S	chr11:34095	Plasma men	SFK1	chr11	YKL051W	---	P35735	---	---	---	0006629 // l	0005886 // p	0005515 // r	---	S000001534	This probe s	S000001534(11),GENSCAN00000004503(11),YKL051W(11)
1778312_at	9330.39	P	2706.54	P	3.4473	P	YKL043W	S. cerevisiae	YKL043W S	chr11:35638	Transcriptio	PHD1	chr11	YKL043W	---	P36093 /// C	---	---	---	0006350 // t	0005634 // r	0003677 // [	---	---	This probe s	S000001526(11),GENEFINDER00000001636(11),GENSCAN00000004587(11),YKL043W(11)
1775851_at	2625.6	P	591.16	P	4.4414	P	YKL026C	S. cerevisiae	YKL026C S	chr11:38901	Phospholipic	GPX1	chr11	YKL026C	---	P36014	---	---	---	0006979 // r	---	0004601 // r	---	---	This probe s	S000001509(11),GENSCAN00000004814(11),YKL026C(11)
1774365_at	4412.77	P	768.91	P	5.739	P	YKL001C	S. cerevisiae	YKL001C S	chr11:43841	Adenylylsulfa	MET14	chr11	YKL001C	---	Q02196	---	---	---	0000103 // s	0005622 // i	0000166 // r	---	---	This probe s	S000001484(11),GENEFINDER00000001792(11),GENSCAN00000004624(11),YKL001C(11)
1769308_at	3191.83	P	721.64	P	4.423	P	YKR009C	S. cerevisiae	YKR009C S	chr11:45398	Multifunction	FOX2	chr11	YKR009C	---	Q02207	EC:4.2.1.74	NP_012934	---	0006629 // l	0005777 // r	0003824 // c	---	---	This probe s	M86456(11)
1770480_at	866.37	P	227.52	P	3.8079	P	YKR039W	S. cerevisiae	YKR039W S	chr11:51469	General ami	GAP1	chr11	YKR039W	---	P19145	---	---	---	0006810 // t	0000328 // f	0005515 // r	---	S000001747	This probe s	S000001747(11),GENEFINDER00000001770(11),GENSCAN00000004321(11),YKR039W(11)
1774671_at	5116.18	P	1352.97	P	3.7814	P	YKR046C	S. cerevisiae	YKR046C S	chr11:52385	Protein of ur	PET10	chr11	YKR046C	---	P36139	---	---	---	0009060 // a	0005811 // l	---	---	---	This probe s	S000001754(11),GENEFINDER00000001720(11),GENSCAN00000004344(11),YKR046C(11)
1771265_at	3464.05	P	677.08	P	5.1162	P	YKR049C	S. cerevisiae	YKR049C S	chr11:52646	Putative red	FMP46	chr11	YKR049C	---	P36141	---	---	---	0034605 // c	0005739 // r	0005515 // r	---	---	This probe s	S000001757(11),YKR049C(11)
1771744_at	3118.68	P	654.1	P	4.7679	P	YKR052C	S. cerevisiae	YKR052C S	chr11:53218	Iron transpo	MRS4	chr11	YKR052C	---	P23500	---	---	---	0006397 // r	0005739 // r	0005381 // i	---	---	This probe s	S000001760(11),GENSCAN00000004583(11),YKR052C(11)
1772791_at	9363.4	P	3154.39	P	2.9684	P	YKR066C	S. cerevisiae	YKR066C S	chr11:56574	Mitochondria	CCP1	chr11	YKR066C	---	P00431	---	---	---	0006979 // r	0005739 // r	0004130 // c	---	S000001774	This probe s	S000001774(11),GENEFINDER00000001747(11),GENSCAN00000004585(11),YKR066C(11)
1772694_at	3530.19	P	1617.6	P	2.1824	P	YKR067W	S. cerevisiae	YKR067W S	chr11:56755	Glycerol-3-p	GPT2	chr11	YKR067W	---	P36148	---	---	---	0008152 // r	0005737 // c	0004366 // c	IPR002123 /	S000001775	This probe s	S000001775(11),GENEFINDER00000001728(11),GENSCAN00000004438(11),YKR067W(11)
1778632_at	2833.89	P	329.4	P	8.6032	P	YKR069W	S. cerevisiae	YKR069W S	chr11:57124	S-adenosyl-	MET1	chr11	YKR069W	---	A0SXK0 ///	---	---	---	0000103 // s	0005622 // i	0004851 // u	---	---	This probe s	S000001777(11),GENEFINDER00000001861(11),GENSCAN00000004443(11),YKR069W(11)
1772192_at	4412.5	P	1949.56	P	2.2633	P	YKR071C	S. cerevisiae	YKR071C S	chr11:57456	Protein that	DRE2	chr11	YKR071C	---	P36152	---	---	---	0007064 // r	0005737 // c	0005515 // r	---	---	This probe s	S000001779(11),GENEFINDER00000001737(9),GENSCAN00000004807(11),YKR071C(11)
1778040_at	2720.69	P	597.35	P	4.5546	P	YKR076W	S. cerevisiae	YKR076W S	chr11:58191	Omega clas	ECM4	chr11	YKR076W	---	P36156	---	---	---	0007047 // c	0005737 // c	0004364 // c	---	---	This probe s	S000001784(11),GENSCAN00000004762(11),YKR076W(11)
1779973_at	1922.24	P	643.47	P	2.9873	P	YKR091W	S. cerevisiae	YKR091W S	chr11:61144	Cytoplasmic	SRL3	chr11	YKR091W	---	P36167	---	---	---	0006139 // r	0005737 // c	---	---	---	This probe s	S000001799(11),GENSCAN00000004796(11),YKR091W(11)
1777196_at	313.54	P	110.92	P	2.8267	P	YKR097W	S. cerevisiae	YKR097W S	chr11:63078	Phosphoeno	PCK1	chr11	YKR097W	---	P10963 /// C	---	---	---	0006094 // c	0005829 // c	0000166 // r	---	---	This probe s	S000001805(11),U24234(9),GENSCAN00000004572(11),GENEFINDER00000001644(11),YKR097W(11)
1771410_at	1042.43	P	519.24	P	2.0076	P	YKR098C	S. cerevisiae	YKR098C S	chr11:63265	Ubiquitin-spe	UBP11	chr11	YKR098C	---	P36026	---	---	---	0006511 // u	---	0004221 // u	---	---	This probe s	S000001806(11),GENEFINDER00000001754(11),GENSCAN00000004370(11),YKR098C(11)
1779467_at	618.55	P	170.62	P	3.6253	P	YKR102W	S. cerevisiae	YKR102W S	chr11:64598	Lectin-like p	FLO10	chr11	YKR102W	---	P36170	---	---	---	---	0005576 // e	---	---	S000001810	This probe s	S000001810(11),GENEFINDER00000001767(11),GENSCAN00000004710(11),YKR102W(11)
1771403_at	347.43	P	132.86	P	2.615	P	YKR104W	S. cerevisiae	YKR104W S	chr11:65646	---	---	chr11	YKR104W	---	P36028	---	---	---	0006810 // t	0016020 // r	0000166 // r	---	---	This probe s	S000001812(11),GENSCAN00000004669(11),YKR104W(11)
1774003_at	347.08	P	126.28	P	2.7485	P	YLL066W-B	S. cerevisiae	YLL066W-B	chr12:5604-	---	---	chr12	YLL066W-B	---	Q8TGJ7	---	---	---	---	---	---	---	---	This probe s	S000028672(11),YLL066W-B(11)
1776349_s_at	808.79	P	386.38	P	2.0933	P	YLL066W-B	S. cerevisiae	YLL066W-B	chr12:5604-	---	---	chr12 /// chr	YBL109W //	---	P38898 /// F	---	---	---	---	---	---	---	---	This probe s	S000002150(10),S000002952(11),S000001260(11),S000028672(11),S000028686(11),S000005282(11),YHR217C(11),YNL339W(11),YBL109W(10),YLL066W-B(11),YLR466C-B(11),YDR544C(11)
1773363_x_at	599.61	P	241.55	P	2.4823	P	YLL066W-B	S. cerevisiae	YLL066W-B	chr12:5604-	---	---	chr12	YLL066W-B	---	Q8TGJ7	---	---	---	---	---	---	---	---	This probe s	S000028672(11),YLL066W-B(11)
1776767_at	3330.95	P	203.1	P	16.4005	P	YLL062C	S. cerevisiae	YLL062C S	chr12:16638	S-methylmet	MHT1	chr12	YLL062C	---	Q12525	---	---	---	0000096 // s	0005737 // c	0005515 // r	---	---	This probe s	S000003985(11),GENSCAN00000007553(11),YLL062C(11)
1778233_at	2472.84	P	458.03	P	5.3989	P	YLL061W	S. cerevisiae	YLL061W S	chr12:17955	High-affinity	MMP1	chr12	YLL061W	---	Q12372	---	---	---	0006810 // t	0005783 // e	0000100 // S	---	S000003984	This probe s	S000003984(11),GENSCAN00000007192(11),YLL061W(11)
1779613_at	3433.78	P	273.9	P	12.5366	P	YLL060C	S. cerevisiae	YLL060C S	chr12:21137	Glutathione	GTT2	chr12	YLL060C	---	Q12390	---	---	---	0006749 // c	0005739 // r	0004364 // c	---	---	This probe s	S000003983(11),GENEFINDER00000003170(11),GENSCAN00000007649(11),YLL060C(11)
1770340_at	1732.18	P	686.53	P	2.5231	P	YLL058W	S. cerevisiae	YLL058W S	chr12:23568	---	---	chr12	YLL058W	---	Q12198	---	---	---	0006520 // a	---	0003824 // c	---	---	This probe s	S000003981(11),GENSCAN00000007031(11),GENEFINDER00000003231(11),YLL058W(11)
1779211_at	306.89	P	64.73	P	4.7411	P	YLL057C	S. cerevisiae	YLL057C S	chr12:25755	Fe(II)-deper	JLP1	chr12	YLL057C	---	Q12358	---	---	---	0044273 // s	---	0000907 // s	---	---	This probe s	S000003980(11),GENEFINDER00000003211(11),GENSCAN00000007306(11),YLL057C(11)
1778834_at	2799.49	P	282.68	P	9.9034	P	YLL056C	S. cerevisiae	YLL056C S	chr12:27408	---	---	chr12	YLL056C	---	Q12177	---	---	---	0008152 // r	---	0003824 // c	IPR002225 /	---	This probe s	S000003979(11),GENEFINDER00000003024(11),GENSCAN00000007639(11),YLL056C(11)
1776384_at	2407.77	P	455.53	P	5.2856	P	YLL055W	S. cerevisiae	YLL055W S	chr12:30108	High-affinity	YCT1	chr12	YLL055W	---	Q12235	---	---	---	0042883 // c	0005783 // e	0033229 // c	---	S000003978	This probe s	S000003978(11),GENSCAN00000007125(11),YLL055W(11)
1777394_at	608.09	P	236.55	P	2.5707	P	YLL042C	S. cerevisiae	YLL042C S	chr12:52085	Conserved F	ATG10	chr12	YLL042C	---	Q07879	---	---	---	0006464 // r	0005737 // c	0019777 // A	---	---	This probe s	S000003965(11),YLL042C(11)
1780187_at	9705.25	P	4755.75	P	2.0407	P	YLL023C	S. cerevisiae	YLL023C S	chr12:97995	---	---	chr12	YLL023C	---	Q12164	---	---	---	---	0005783 // e	0005515 // r	---	S000003946	This probe s	S000003946(11),GENEFINDER00000003123(11),GENSCAN00000007652(11),YLL023C(11)
1773588_at	862.28	P	117.68	P	7.3273	P	YLR004C	S. cerevisiae	YLR004C S	chr12:15793	Putative plas	THI73	chr12	YLR004C	---	Q07904	---	---	---	---	0005783 // e	---	---	S000003994	This probe s	S000003994(11),GENSCAN00000007624(11),YLR004C(11)
1770886_at	78.43	P	34.38	P	2.2813	P	YLR030W	S. cerevisiae	YLR030W S	chr12:20329	---	---	chr12	YLR030W	---	Q07967	---	---	---	---	---	0005515 // r	---	---	This probe s	S000004020(11),GENEFINDER00000003223(10),GENSCAN00000007510(11),YLR030W(11)
1779091_at	315.65	P	61.62	P	5.1225	P	YLR031W	S. cerevisiae	YLR031W S	chr12:20422	---	---	chr12	YLR031W	---	Q07978	---	---	---	---	---	0005515 // r	---	---	This probe s	S000004021(11),GENSCAN00000007359(11),YLR031W(11)
1778088_at	2367.08	P	651.25	P	3.6347	P	YLR046C	S. cerevisiae	YLR046C S	chr12:23800	---	---	chr12	YLR046C	---	Q12253	---	---	---	0006950 // r	0016020 // r	---	---	S000004036	This probe s	S000004036(11),YLR046C(11)
1780022_at	626.51	P	56.67	P	11.0554	P	YLR053C	S. cerevisiae	YLR053C S	chr12:24810	---	---	chr12	YLR053C	---	Q12026	---	---	---	---	---	---	---	---	This probe s	S000004043(11),GENEFINDER00000003125(11),YLR053C(11)
1769520_at	5640.96	P	2499.12	P	2.2572	P	YLR064W	S. cerevisiae	YLR064W S	chr12:26545	---	---	chr12	YLR064W	---	Q12144	---	---	---	0034605 // c	0005783 // e	0005515 // r	---	S000004054	This probe s	S000004054(11),YLR064W(11)
1778656_at	788.67	P	259.98	P	3.0336	P	YLR080W	S. cerevisiae	YLR080W S	chr12:28791	Integral mer	EMP46	chr12	YLR080W	---	Q12396	---	---	---	0006810 // t	0000139 // C	0001948 // c	---	S000004070	This probe s	S000004070(11),GENEFINDER00000003003(11),GENSCAN00000007378(11),YLR080W(11)
1774952_at	2206.65	P	184.68	P	11.9485	P	YLR092W	S. cerevisiae	YLR092W S	chr12:32354	High affinity	SUL2	chr12	YLR092W	---	Q12325	---	---	---	0006810 // t	0005886 // p	0005215 // t	IPR002645 /	S000004082	This probe s	S000004082(11),GENEFINDER00000002974(11),GENSCAN00000007616(11),YLR092W(11)
1776159_at	2541.37	P	945.26	P	2.6885	P	YLR097C	S. cerevisiae	YLR097C S	chr12:33623	Putative SCI	HRT3	chr12	YLR097C	---	Q12347	---	---	---	0019941 // r	0019005 // S	---	---	---	This probe s	S000004087(11),GENSCAN00000007097(11),YLR097C(11)
1772365_at	3291.41	P	512.64	P	6.4205	P	YLR108C	S. cerevisiae	YLR108C S	chr12:36666	---	---	chr12	YLR108C	---	Q12259	---	---	---	---	0005634 // r	0005515 // r	---	---	This probe s	S000004098(11),YLR108C(11)
1769482_at	353.16	P	127.02	P	2.7803	P	YLR125W	S. cerevisiae	YLR125W S	chr12:39348	---	---	chr12	YLR125W	---	Q12138	---	---	---	---	---	0005515 // p	---	---	This probe s	S000004115(11),GENSCAN00000007385(11),YLR125W(11)
1779910_at	6115.78	P	2081.7	P	2.9379	P	YLR130C	S. cerevisiae	YLR130C S	chr12:40279	Low-affinity	ZRT2	chr12	YLR130C	---	Q12436	---	---	---	0006810 // t	0005886 // p	0000007 // l	---	S000004120	This probe s	S000004120(11),GENEFINDER00000003065(11),GENSCAN00000007230(11),YLR130C(11)
1774937_at	16982.47	P	1502.25	P	11.3047	P	YLR134W	S. cerevisiae	YLR134W S	chr12:41072	Minor isofor	PDC5	chr12	YLR134W	---	P16467	---	---	---	0000949 // a	0005634 // r	0000287 // r	---	---	This probe s	S000004124(11),GENEFINDER00000003098(11),GENSCAN00000007605(11),YLR134W(11)
1775678_at	1825.51	P	110.45	P	16.5279	P	YLR142W	S. cerevisiae	YLR142W S	chr12:42518	Proline oxida	PUT1	chr12	YLR142W	---	P09368	---	---	---	0006537 // c	0005739 // r	0004657 // r	---	GENSCAN0	This probe s	S000004132(11),GENEFINDER00000003113(11),GENSCAN00000007593(11),YLR142W(11)
1769544_at	1321.36	P	243.96	P	5.4163	P	YLR149C	S. cerevisiae	YLR149C S	chr12:43763	---	---	chr12	YLR149C	---	Q99296	---	---	---	---	---	---	---	---	This probe s	S000004139(11),GENEFINDER00000003164(11),GENSCAN00000007559(11),YLR149C(11)
1776002_s_at	1934.06	P	298.05	P	6.489	P	YLR156W	S. cerevisiae	YLR156W S	chr12:48899	---	---	chr12	YLR156W //	---	Q12478	---	---	---	---	---	0005515 // r	---	---	This probe s	S000004146(11),S000028677(11),S000004149(11),S000004151(11),YLR156W(11),YLR157W-D(11),YLR159W(11),YLR161W(11)
1775038_s_at	2326.41	P	314.9	P	7.3878	P	YLR157W-C	S. cerevisiae	YLR157W-C	chr12:48187	---	---	chr12	YLR156W //	---	Q12478	---	---	---	---	---	0005515 // r	---	---	This probe s	S000004146(11),S000028678(11),S000004149(11),S000004151(11),YLR156W(11),YLR157W-E(11),YLR159W(11),YLR161W(11)
1779688_at	1131.82	P	273.58	P	4.1371	P	YLR164W	S. cerevisiae	YLR164W S	chr12:49388	---	---	chr12	YLR164W	---	Q06236	---	---	---	0006099 // t	0005739 // r	0005506 // i	---	---	This probe s	S000004154(11),YLR164W(11)
1770891_at	1190.58	P	571.86	P	2.0819	P	YLR165C	S. cerevisiae	YLR165C S	chr12:49449	Pseudouridir	PUS5	chr12	YLR165C	---	Q06244	---	---	---	0000154 // r	0005739 // r	0003723 // F	---	---	This probe s	S000004155(11),YLR165C(11)
1773506_at	602.93	P	267.32	P	2.2555	P	YLR173W	S. cerevisiae	YLR173W S	chr12:50242	---	---	chr12	YLR173W	---	Q06247	---	---	---	---	0016020 // r	---	---	S000004163	This probe s	S000004163(11),GENEFINDER00000003061(11),GENSCAN00000007095(11),YLR173W(11)
1779660_at	1013.39	P	286.35	P	3.539	P	YLR174W	S. cerevisiae	YLR174W S	chr12:50459	Cytosolic NA	IDP2	chr12	YLR174W	---	P41939	---	---	---	0006097 // c	0005737 // c	0000287 // r	---	---	This probe s	S000004164(11),GENEFINDER00000003061(11),GENSCAN00000007050(11),YLR174W(11)
1774403_at	7061.97	P	2455.57	P	2.8759	P	YLR177W	S. cerevisiae	YLR177W S	chr12:51105	---	---	chr12	YLR177W	---	Q06251	---	---	---	---	0005737 // c	0005515 // p	---	---	This probe s	S000004167(11),GENEFINDER00000003049(11),GENSCAN00000007119(11),YLR177W(11)
1779498_at	10801.02	P	2501.96	P	4.317	P	YLR178C	S. cerevisiae	YLR178C S	chr12:51316	Carboxypep	TFS1	chr12	YLR178C	---	P14306	---	---	---	0030162 // r	0000328 // f	0004866 // e	---	---	This probe s	S000004168(11),GENEFINDER00000003168(11),GENSCAN00000007488(11),YLR178C(11)
1770792_at	4565.24	P	1756.6	P	2.5989	P	YLR237W	S. cerevisiae	YLR237W S	chr12:61236	Plasma mer	THI7	chr12	YLR237W	---	Q05998	---	---	---	0006810 // t	0005886 // p	0005515 // r	---	S000004227	This probe s	S000004227(11),GENEFINDER00000003067(11),GENSCAN00000007613(11),YLR237W(11)
1779774_at	1140.31	P	566.01	P	2.0146	P	YLR247C	S. cerevisiae	YLR247C S	chr12:62868	Putative heli	IRC20	chr12	YLR247C	---	Q06554	---	---	---	0006950 // r	0005634 // r	0000166 // r	---	---	This probe s	S000004237(11),GENSCAN00000007319(11),YLR247C(11)
1772447_at	3361.76	P	1146.04	P	2.9334	P	YLR251W	S. cerevisiae	YLR251W S	chr12:64146	Protein requ	SYM1	chr12	YLR251W	---	Q06563	---	---	---	0006067 // e	0005739 // r	---	---	---	This probe s	S000004241(11),YLR251W(11)
1771340_at	244.16	P	76.73	P	3.1821	P	YLR267W	S. cerevisiae	YLR267W S	chr12:67821	Protein of ur	BOP2	chr12	YLR267W	---	Q06150	---	---	---	---	---	0005515 // r	---	GENEFINDE	This probe s	S000004257(11),GENSCAN00000007541(11),GENEFINDER00000003124(10),YLR267W(11)
1772009_at	5015.03	P	2034.14	P	2.4654	P	YLR270W	S. cerevisiae	YLR270W S	chr12:68118	Non-essentia	DCS1	chr12	YLR270W	---	Q06151	---	NP_013372	---	0000184 // r	0000932 // c	0003824 // c	---	GENEFINDE	This probe s	AY165035(11)
1776191_at	922.42	P	266.58	P	3.4602	P	YLR281C	S. cerevisiae	YLR281C S	chr12:70449	---	---	chr12	YLR281C	---	Q05863	---	---	---	0006415 // t	0005739 // r	0003747 // t	IPR000352 /	---	This probe s	S000004271(11),GENEFINDER00000002993(11),GENSCAN00000007502(11),YLR281C(11)
1776978_at	3252.44	P	1479.41	P	2.1985	P	YLR290C	S. cerevisiae	YLR290C S	chr12:71714	---	---	chr12	YLR290C	---	Q05892	---	---	---	0008152 // r	0005739 // r	0003824 // c	IPR013120 /	---	This probe s	S000004281(11),YLR290C(11)
1772193_at	8682.78	P	3086.59	P	2.8131	P	YLR297W	S. cerevisiae	YLR297W S	chr12:72404	---	---	chr12	YLR297W	---	Q05899	---	---	---	---	0000324 // f	---	---	S000004288	This probe s	S000004288(11),GENSCAN00000007354(11),YLR297W(11)
1769870_at	2471.43	P	873.49	P	2.8294	P	YLR299W	S. cerevisiae	YLR299W S	chr12:72606	Gamma-glut	ECM38	chr12	YLR299W	---	Q05902	---	---	---	0006750 // c	0000324 // f	0003840 // c	---	S000004290	This probe s	S000004290(11),GENEFINDER00000003149(11),GENSCAN00000007312(11),YLR299W(11)
1772642_at	2531.72	P	429.16	P	5.8992	P	YLR312C	S. cerevisiae	YLR312C S	chr12:75763	---	---	chr12	YLR312C	---	Q06159	---	---	---	---	0016020 // r	---	---	---	This probe s	S000004303(11),GENSCAN00000007314(11),YLR312C(11)
1778218_at	6221.72	P	2180.01	P	2.854	P	YLR327C	S. cerevisiae	YLR327C S	chr12:78312	Protein of ur	TMA10	chr12	YLR327C	---	Q06177	---	---	---	---	0005634 // r	---	---	---	This probe s	S000004319(11),YLR327C(11)
1774057_at	3193.48	P	1267.11	P	2.5203	P	YLR345W	S. cerevisiae	YLR345W S	chr12:82051	---	---	chr12	YLR345W	---	Q06137	---	---	---	0006000 // f	0005737 // c	0000166 // r	---	---	This probe s	S000004337(11),GENEFINDER00000002971(11),GENSCAN00000007557(11),YLR345W(11)
1769998_at	841.62	P	100.44	P	8.3793	P	YLR346C	S. cerevisiae	YLR346C S	chr12:82228	---	---	chr12	YLR346C	---	Q06139	---	---	---	0009636 // r	0005739 // r	---	---	---	This probe s	S000004338(11),YLR346C(11)
1771801_at	3750.38	P	1525.76	P	2.458	P	YLR356W	S. cerevisiae	YLR356W S	chr12:84031	---	---	chr12	YLR356W	---	Q06485	---	---	---	---	0005739 // r	---	---	S000004348	This probe s	S000004348(11),GENSCAN00000007519(11),YLR356W(11)
1770687_at	970.87	P	341.44	P	2.8435	P	YLR364W	S. cerevisiae	YLR364W S	chr12:85406	Glutaredoxir	GRX8	chr12	YLR364W	---	Q05926	---	---	---	0006810 // t	0005737 // c	0004362 // c	---	---	This probe s	S000004356(11),GENSCAN00000007231(11),YLR364W(11)
1777027_at	6454.94	P	2745.94	P	2.3507	P	YLR375W	S. cerevisiae	YLR375W S	chr12:87169	Zinc-finger p	STP3	chr12	YLR375W	---	Q05937	---	---	---	---	0005622 // i	0003676 // r	---	---	This probe s	S000004367(11),GENEFINDER00000003078(11),GENSCAN00000007546(11),YLR375W(11)
1778613_at	2876.71	P	1433.59	P	2.0066	P	YLR390W	S. cerevisiae	YLR390W S	chr12:90306	Putative pro	ECM19	chr12	YLR390W	---	Q06011	---	---	---	0007047 // c	0005739 // r	---	---	S000004382	This probe s	S000004382(11),GENSCAN00000007539(11),YLR390W(11)
1775480_at	8018.85	P	3406.03	P	2.3543	P	YLR438W	S. cerevisiae	YLR438W S	chr12:10124	L-ornithine t	CAR2	chr12	YLR438W	---	P07991	---	---	---	0006527 // a	0005634 // r	0003824 // c	---	---	This probe s	S000004430(11),GENEFINDER00000003100(11),GENSCAN00000007424(11),YLR438W(11)
1773688_at	1048.47	P	520.11	P	2.0159	P	YLR446W	S. cerevisiae	YLR446W S	chr12:10252	---	---	chr12	YLR446W	---	Q06204	---	---	---	0005975 // c	---	0000166 // r	IPR001312 /	---	This probe s	S000004438(11),GENSCAN00000007110(11),YLR446W(11)
1778286_at	1479.04	P	561.38	P	2.6347	P	YLR466C-B	S. cerevisiae	YLR466C-B	chr12:5604-	---	---	chr12	YLR466C-B	---	Q8TGJ4	---	---	---	---	---	---	---	---	This probe s	S000028686(11),YLR466C-B(11)
1778724_x_at	1431.59	P	567.89	P	2.5209	P	YLR466C-B	S. cerevisiae	YLR466C-B	chr12:5604-	---	---	chr12	YLR466C-B	---	Q8TGJ4	---	---	---	---	---	---	---	---	This probe s	S000028686(11),YLR466C-B(11)
1771515_at	6997.74	P	1494.77	P	4.6815	P	YML131W	S. cerevisiae	YML131W S	chr13:10198	---	---	chr13	YML131W	---	Q03102 /// C	---	---	---	0006970 // r	0005737 // c	0003824 // c	---	S000004600	This probe s	S000004600(11),GENEFINDER00000001948(11),GENSCAN00000006960(11),YML131W(11)
1775014_at	9052.74	P	1895.03	P	4.7771	P	YML128C	S. cerevisiae	YML128C S	chr13:15135	Protein of ur	MSC1	chr13	YML128C	---	Q03104	---	---	---	0007131 // r	0005739 // r	---	---	---	This probe s	S000004597(11),GENEFINDER00000001811(11),GENSCAN00000006766(11),YML128C(11)
1774756_at	11435.72	P	4596.25	P	2.4881	P	YML100W	S. cerevisiae	YML100W S	chr13:70623	Large subur	TSL1	chr13	YML100W	---	P38427	---	---	---	0005992 // t	0005737 // c	0003824 // c	---	---	This probe s	S000004566(11),GENEFINDER00000001969(11),GENSCAN00000006764(11),YML100W(11)
1772703_at	753.86	P	362.5	P	2.0796	P	YML098W	S. cerevisiae	YML098W S	chr13:77266	TFIID subun	TAF13	chr13	YML098W	---	P11747	---	---	---	0000114 // r	0005634 // r	0003677 // [	---	---	This probe s	S000004564(11),GENEFINDER00000001952(11),GENSCAN00000006086(11),YML098W(11)
1773386_at	974.13	P	359.97	P	2.7061	P	YML087C	S. cerevisiae	YML087C S	chr13:94430	Putative pro	AIM33	chr13	YML087C	---	Q04516	---	---	---	0055114 // c	0016020 // r	0009055 // e	---	S000004552	This probe s	S000004552(11),GENSCAN00000006310(11),YML087C(11)
1770137_at	164.39	P	24.81	P	6.626	P	YML083C	S. cerevisiae	YML083C S	chr13:99793	---	---	chr13	YML083C	---	Q04526	---	---	---	---	---	---	---	---	This probe s	S000004548(11),GENSCAN00000006514(11),YML083C(11)
1772338_at	6970.61	P	3447.21	P	2.0221	P	YML070W	S. cerevisiae	YML070W S	chr13:13347	Dihydroxyac	DAK1	chr13	YML070W	---	P54838	---	---	---	0006071 // c	0005737 // c	0004371 // c	---	---	This probe s	S000004535(11),GENEFINDER00000001660(11),GENSCAN00000006056(11),YML070W(11)
1776837_at	893.39	P	147.45	P	6.0589	P	YML058W-A	S. cerevisiae	YML058W-A	chr13:15875	Protein invol	HUG1	chr13	YML058W-A	---	Q6Q5K6	---	---	---	0000075 // c	0005634 // r	0005515 // r	---	---	This probe s	S000007472(11),YML058W-A(11)
1771143_at	832.43	P	65.16	P	12.7752	P	YML054C	S. cerevisiae	YML054C S	chr13:16553	Cytochrome	CYB2	chr13	YML054C	---	P00175	---	---	---	0006089 // l	0005739 // r	0003824 // c	---	---	This probe s	S000004518(11),GENEFINDER00000001890(11),GENSCAN00000006781(11),YML054C(11)
1774377_at	1050.25	P	202.09	P	5.1969	P	YML042W	S. cerevisiae	YML042W S	chr13:19278	Carnitine ac	CAT2	chr13	YML042W	---	P32796	---	---	---	0006629 // l	0005739 // r	0004092 // c	---	---	This probe s	S000004506(11),GENSCAN00000006476(11),GENEFINDER00000001913(11),YML042W(11)
1777589_at	8051.62	P	3691.38	P	2.1812	P	YML004C	S. cerevisiae	YML004C S	chr13:26170	Monomeric g	GLO1	chr13	YML004C	---	P50107	---	---	---	0006749 // c	0005634 // r	0004462 // l	---	---	This probe s	S000004463(11),GENEFINDER00000001922(11),GENSCAN00000006757(11),YML004C(11)
1775415_at	812.04	P	374.52	P	2.1682	P	YML002W	S. cerevisiae	YML002W S	chr13:26454	---	---	chr13	YML002W	---	Q04257	---	---	---	---	---	---	---	---	This probe s	S000004461(11),GENSCAN00000006333(11),YML002W(11)
1778335_at	3424.37	P	773.27	P	4.4284	P	YMR009W	S. cerevisiae	YMR009W S	chr13:28410	Acireductone	ADI1	chr13	YMR009W	---	Q03677	---	---	---	0008652 // a	0005634 // r	0016151 // r	IPR003313 /	---	This probe s	S000004611(11),GENEFINDER00000001954(11),GENSCAN00000006012(11),YMR009W(11)
1777721_at	1655.29	P	746.39	P	2.2177	P	YMR020W	S. cerevisiae	YMR020W S	chr13:31537	Polyamine o	FMS1	chr13	YMR020W	---	P50264	---	---	---	0006598 // r	0005737 // c	0008131 // a	IPR002938 /	---	This probe s	S000004622(11),GENEFINDER00000001937(11),GENSCAN00000006646(11),YMR020W(11)
1779146_at	688.11	P	283.64	P	2.426	P	YMR030W	S. cerevisiae	YMR030W S	chr13:33079	Protein requ	RSF1	chr13	YMR030W	---	Q05043	---	---	---	0006350 // t	0005634 // r	0005515 // r	---	---	This probe s	S000004632(11),GENSCAN00000006221(11),GENEFINDER00000001675(11),YMR030W(11)
1775362_at	1107	P	441.31	P	2.5084	P	YMR040W	S. cerevisiae	YMR040W S	chr13:35037	Protein of ur	YET2	chr13	YMR040W	---	Q04210	---	---	---	0006810 // t	0005783 // e	---	---	S000004643	This probe s	S000004643(11),YMR040W(11)
1775922_at	874.83	P	419.02	P	2.0878	P	YMR041C	S. cerevisiae	YMR041C S	chr13:35096	NAD-depenc	ARA2	chr13	YMR041C	---	Q04212	---	---	---	0019853 // L	---	0005515 // p	---	---	This probe s	S000004644(11),GENSCAN00000006133(11),YMR041C(11)
1772863_at	384.59	P	182.17	P	2.1112	P	YMR053C	S. cerevisiae	YMR053C S	chr13:38034	Protein that	STB2	chr13	YMR053C	---	P46679	---	---	---	0016575 // h	0005634 // r	0016564 // t	---	---	This probe s	S000004657(11),GENEFINDER00000001574(11),GENSCAN00000006965(11),YMR053C(11)
1775433_at	3267.37	P	818.86	P	3.9901	P	YMR056C	S. cerevisiae	YMR056C S	chr13:38731	Mitochondria	AAC1	chr13	YMR056C	---	P04710	---	---	---	0006783 // h	0005739 // r	0005215 // t	---	S000004660	This probe s	S000004660(11),GENEFINDER00000001778(11),GENSCAN00000006816(11),YMR056C(11)
1776272_at	4747.93	P	2279.56	P	2.0828	P	YMR062C	S. cerevisiae	YMR062C S	chr13:39505	Mitochondria	ECM40	chr13	YMR062C	---	Q04728	---	---	---	0006526 // a	0005739 // r	0003824 // c	---	---	This probe s	S000004666(11),GENEFINDER00000001642(10),GENSCAN00000006546(11),YMR062C(11)
1779232_at	498.06	P	235.75	P	2.1127	P	YMR068W	S. cerevisiae	YMR068W S	chr13:40630	Component	AVO2	chr13	YMR068W	---	Q04749	---	---	---	0001558 // r	0005737 // c	0005515 // r	---	---	This probe s	S000004672(11),GENSCAN00000006747(11),GENEFINDER00000001881(11),YMR068W(11)
1774501_at	78.42	P	19.02	P	4.123	P	YMR084W	S. cerevisiae	YMR084W S	chr13:43662	---	---	chr13	YMR084W	---	A2P2R3 /// C	---	---	---	0005975 // c	---	0004360 // c	---	---	This probe s	S000004689(11),GENSCAN00000006390(11),YMR084W(11)
1778475_at	1019.46	P	107.44	P	9.4886	P	YMR085W	S. cerevisiae	YMR085W S	chr13:43748	---	---	chr13	YMR085W	---	Q6B308	---	---	---	0005975 // c	---	0004360 // c	---	---	This probe s	S000004690(11),GENEFINDER00000001872(9),GENSCAN00000006587(11),YMR085W(11)
1770335_at	2033.61	P	651.94	P	3.1193	P	YMR087W	S. cerevisiae	YMR087W S	chr13:44252	---	---	chr13	YMR087W	---	Q04299	---	---	---	0006388 // t	---	---	---	---	This probe s	S000004693(11),YMR087W(11)

1775890_at	7569.68	P	2107.61	P	3.5916	P	YMR090W	S. cerevisiae	YMR090W S	chr13:44924	---	---	chr13	YMR090W	---	Q04304	---	---	---	0008152 //	0005737 //	0003824 //	IPR000534	---	This probe s	S000004696(11),GENSCAN00000006060(11),YMR090W(11)
1776127_at	2526.3	P	982.81	P	2.5705	P	YMR102C	S. cerevisiae	YMR102C S	chr13:46984	---	---	chr13	YMR102C	---	Q03177	---	---	---	---	---	0005515 //	---	---	This probe s	S000004708(11),GENSCAN00000006505(11),YMR102C(11)
1775390_at	1899.28	P	602.55	P	3.1521	P	YMR104C	S. cerevisiae	YMR104C S	chr13:47341	Protein kina	YPK2	chr13	YMR104C	---	P18961	---	---	---	0006468 //	0005634 //	0000166 //	IPR001245	---	This probe s	S000004710(11),GENSCAN00000006025(11),YMR104C(11)
1778907_at	7743.05	P	3734.91	P	2.0732	P	YMR105C	S. cerevisiae	YMR105C S	chr13:47589	Phosphogluc	PGM2	chr13	YMR105C	---	P37012	---	---	---	0005975 //	0005737 //	0000287 //	IPR005844	---	This probe s	S000004711(11),GENEFINDER00000001942(11),GENSCAN00000006808(11),YMR105C(11)
1773545_at	169.95	P	49.94	P	3.4031	P	YMR107W	S. cerevisiae	YMR107W S	chr13:48301	Protein regu	SPG4	chr13	YMR107W	---	Q04438	---	---	---	---	---	---	---	---	This probe s	S000004713(11),GENEFINDER00000001836(11),GENSCAN00000006748(11),YMR107W(11)
1774440_at	7122.07	P	2831.24	P	2.5155	P	YMR110C	S. cerevisiae	YMR110C S	chr13:49039	Putative fatt	HFD1	chr13	YMR110C	---	Q04458	---	---	---	0006081 //	0005739 //	0004030 //	---	S000004716	This probe s	S000004716(11),GENEFINDER00000001909(11),GENSCAN00000006725(11),YMR110C(11)
1773831_at	501.69	P	204.39	P	2.4546	P	YMR114C	S. cerevisiae	YMR114C S	chr13:49634	---	---	chr13	YMR114C	---	Q04471	---	---	---	---	0005634 //	---	---	---	This probe s	S000004720(11),GENEFINDER00000001923(11),GENSCAN00000006753(11),YMR114C(11)
1773702_at	1079.29	P	61.86	P	17.4473	P	YMR118C	S. cerevisiae	YMR118C S	chr13:50214	---	---	chr13	YMR118C	---	Q04487	---	---	---	0006099 //	0005739 //	0000104 //	---	S000004724	This probe s	S000004724(11),YMR118C(11)
1773376_at	361.04	P	68.28	P	5.2876	P	YMR133W	S. cerevisiae	YMR133W S	chr13:53620	Protein invol	REC114	chr13	YMR133W	---	P32841	---	---	---	0006310 //	0000794 //	0005515 //	---	---	This probe s	S000004740(11),GENSCAN00000006251(11),YMR133W(11)
1779962_at	3563.87	P	1672.84	P	2.1304	P	YMR139W	S. cerevisiae	YMR139W S	chr13:54612	Protein kina	RIM11	chr13	YMR139W	---	P38615	---	---	---	0006468 //	0005737 //	0000166 //	IPR001245	---	This probe s	S000004747(11),GENSCAN00000006349(11),YMR139W(11)
1780234_at	376.87	P	134.26	P	2.807	P	YMR154C	S. cerevisiae	YMR154C S	chr13:56599	Calpain-like	RIM13	chr13	YMR154C	---	Q03792	---	---	---	0006508 //	0005622 //	0004197 //	---	---	This probe s	S000004763(11),GENSCAN00000006886(11),YMR154C(11)
1769805_at	5221.39	P	554.32	P	9.4195	P	YMR169C	S. cerevisiae	YMR169C S	chr13:59935	Cytoplasmic	ALD3	chr13	YMR169C	---	P54114	---	---	---	0006598 //	---	0004029 //	---	---	This probe s	S000004779(11),GENEFINDER00000001677(11),GENSCAN00000006536(11),YMR169C(11)
1771982_at	1481.45	P	523.67	P	2.829	P	YMR170C	S. cerevisiae	YMR170C S	chr13:60156	Cytoplasmic	ALD2	chr13	YMR170C	---	P47771	---	---	---	0006598 //	0005737 //	0004029 //	---	---	This probe s	S000004780(11),GENEFINDER00000001713(11),GENSCAN00000006398(11),YMR170C(11)
1777444_at	143.84	P	17.7	P	8.1266	P	YMR174C	S. cerevisiae	YMR174C S	chr13:61015	Cytoplasmic	PAI3	chr13	YMR174C	---	P01094	---	---	---	0007039 //	0005737 //	0004866 //	---	---	This probe s	S000004786(11),YMR174C(11)
1774019_at	4440.28	P	72.16	P	61.5338	P	YMR175W	S. cerevisiae	YMR175W S	chr13:61101	Protein of ur	SIP18	chr13	YMR175W	---	P50263	---	---	---	0006970 //	0005625 //	0005543 //	---	---	This probe s	S000004787(11),GENEFINDER00000001858(11),GENSCAN00000006312(11),YMR175W(11)
1779297_at	65.9	P	19.97	P	3.2999	P	YMR175W-	S. cerevisiae	YMR175W-	chr13:61131	---	---	chr13	YMR175W-	---	Q3E766	---	---	---	---	---	---	---	---	This probe s	S000028848(11),YMR175W-A(11)
1771719_at	1035.14	P	490.42	P	2.1107	P	YMR180C	S. cerevisiae	YMR180C S	chr13:62224	RNA 5'-triph	CTL1	chr13	YMR180C	---	Q03220	---	---	---	0006370 //	0005634 //	0004651 //	---	---	This probe s	S000004792(11),GENSCAN00000006628(11),YMR180C(11)
1776402_at	7126.44	P	2700.86	P	2.6386	P	YMR181C	S. cerevisiae	YMR181C S	chr13:62361	---	---	chr13	YMR181C	---	Q03231	---	---	---	---	---	---	---	---	This probe s	S000004793(11),YMR181C(11)
1774631_at	1274.84	P	448.48	P	2.8426	P	YMR182W-	S. cerevisiae	YMR182W-	chr13:62580	---	---	chr13	YMR182W-	---	Q8TGS7	---	---	---	---	0016020 //	---	---	S000028693	This probe s	S000028693(11),YMR182W-A(11)
1771949_at	2320.46	P	902.17	P	2.5721	P	YMR196W	S. cerevisiae	YMR196W S	chr13:65507	---	---	chr13	YMR196W	---	Q04336	---	---	---	---	0005737 //	0003824 //	---	---	This probe s	S000004809(11),GENEFINDER00000001756(11),GENSCAN00000006325(11),YMR196W(11)
1776153_at	1044.63	P	65.67	P	15.9073	P	YMR206W	S. cerevisiae	YMR206W S	chr13:67589	---	---	chr13	YMR206W	---	Q03695	---	---	---	---	---	---	---	---	This probe s	S000004819(11),GENSCAN00000006588(11),YMR206W(11)
1774495_at	9734.28	P	2868.12	P	3.394	P	YMR250W	S. cerevisiae	YMR250W S	chr13:77079	Glutamate d	GAD1	chr13	YMR250W	---	Q04792	---	---	---	0006536 //	0005737 //	0003824 //	---	---	This probe s	S000004862(11),GENEFINDER00000001914(11),GENSCAN00000006142(11),YMR250W(11)
1779968_at	1083.77	P	513.61	P	2.1101	P	YMR253C	S. cerevisiae	YMR253C S	chr13:77594	---	---	chr13	YMR253C	---	Q04835 ///	---	---	---	---	0005737 //	---	---	S000004866	This probe s	S000004866(11),GENSCAN00000006685(11),YMR253C(11)
1777158_at	2786.89	P	1236.45	P	2.2539	P	YMR258C	S. cerevisiae	YMR258C S	chr13:78261	---	---	chr13	YMR258C	---	Q04847	---	---	---	---	0005634 //	---	---	---	This probe s	S000004871(11),GENSCAN00000006392(11),YMR258C(11)
1773683_at	5312.51	P	2463.93	P	2.1561	P	YMR261C	S. cerevisiae	YMR261C S	chr13:79020	Regulatory s	TPS3	chr13	YMR261C	---	P38426	---	---	---	0005992 //	0005737 //	0003824 //	---	---	This probe s	S000004874(11),GENEFINDER00000001972(11),GENSCAN00000006697(11),YMR261C(11)
1780011_at	1622.63	P	401.14	P	4.045	P	YMR262W	S. cerevisiae	YMR262W S	chr13:79372	---	---	chr13	YMR262W	---	P38430 ///	---	---	---	---	---	0016888 //	---	---	This probe s	S000004875(11),GENSCAN00000006666(11),YMR262W(11)
1770593_at	1391.89	P	609.81	P	2.2825	P	YMR265C	S. cerevisiae	YMR265C S	chr13:79653	---	---	chr13	YMR265C	---	Q03508	---	---	---	---	---	---	---	---	This probe s	S000004878(11),GENSCAN00000006624(11),YMR265C(11)
1774157_at	5610.4	P	413.47	P	13.5691	P	YMR271C	S. cerevisiae	YMR271C S	chr13:80686	Minor orotat	URA10	chr13	YMR271C	---	P30402 ///	---	---	---	0006207 //	0005737 //	0004588 //	---	---	This probe s	S000004884(11),GENEFINDER00000001798(11),GENSCAN00000006278(11),YMR271C(11)
1770055_at	254.66	P	107.12	P	2.3773	P	YMR306W	S. cerevisiae	YMR306W S	chr13:88115	Protein invol	FKS3	chr13	YMR306W	---	Q04952	---	---	---	0006075 //	0000148 //	0003843 //	---	S000004923	This probe s	S000004923(11),GENSCAN00000006554(11),YMR306W(11)
1771664_at	8125.22	P	3908.17	P	2.079	P	YMR315W	S. cerevisiae	YMR315W S	chr13:90279	---	---	chr13	YMR315W	---	Q04869	---	---	---	0008152 //	0005634 //	0003824 //	---	---	This probe s	S000004932(11),GENEFINDER00000001618(11),GENSCAN00000006074(11),YMR315W(11)
1769461_at	173.43	P	75.86	P	2.2862	P	YMR317W	S. cerevisiae	YMR317W S	chr13:90736	---	---	chr13	YMR317W	---	Q04893	---	---	---	---	---	---	---	---	This probe s	S000004936(11),GENSCAN00000006548(11),GENEFINDER00000001851(11),YMR317W(11)
1770073_s_at	392.84	P	74.56	P	5.2688	P	YMR322C	S. cerevisiae	YMR322C S	chr16:11886	Possible cha	HSP32 /// H	chr13 /// chr	YMR322C /	---	Q04902 ///	---	---	---	0006950 //	---	0008233 //	---	GENEFINDE	This probe s	S000004941(11),S000005918(11),S000006201(11),GENEFINDER00000001632(9),GENSCAN00000006453(11),GENSCAN00000006618(11),YMR322C(11),YPL280W(11),YOR391C(11)
1776354_s_at	1211.47	P	439.44	P	2.7568	P	YMR323W	S. cerevisiae	YMR323W S	chr13:92008	Protein of ur	ERR1 /// ER	chr13 /// chr	YMR323W /	---	P42222 ///	---	---	---	0006096 //	0000015 //	0000287 //	---	---	This probe s	S000004942(11),S000005920(11),S000006202(11),GENEFINDER00000001612(11),GENSCAN00000006884(11),GENEFINDER00000000915(11),GENSCAN00000006358(11),GENEFINDER00000002221(11),GENSCAN00000007132(11),YMR323W(11),YPL281C(11),YOR393W(11)
1780166_at	450.76	P	205.55	P	2.1929	P	YNL331C	S. cerevisiae	YNL331C S	chr14:16117	Putative aryl	AAD14	chr14	YNL331C	---	P42884	---	---	---	0009636 //	---	0005515 //	---	---	This probe s	S000005275(11),GENEFINDER00000000879(11),GENSCAN00000005269(11),YNL331C(11)
1776883_at	6712.62	P	2684.58	P	2.5004	P	YNL305C	S. cerevisiae	YNL305C S	chr14:58897	---	---	chr14	YNL305C	---	P48558 ///	---	---	---	0034605 //	0000324 //	---	---	S000005249	This probe s	S000005249(11),GENEFINDER00000000855(11),GENSCAN00000005116(11),YNL305C(11)
1778953_at	1828.03	P	806.99	P	2.2652	P	YNL294C	S. cerevisiae	YNL294C S	chr14:78656	Component	RIM21	chr14	YNL294C	---	P48565	---	---	---	0001403 //	0005886 //	---	---	S000005238	This probe s	S000005238(11),GENSCAN00000005429(11),YNL294C(11)
1779413_at	2926.92	P	103	P	28.4167	P	YNL277W	S. cerevisiae	YNL277W S	chr14:11734	L-homoserin	MET2	chr14	YNL277W	---	P08465	---	---	---	0008652 //	0005737 //	0004414 //	---	---	This probe s	S000005221(11),GENEFINDER00000000786(11),GENSCAN00000005091(11),YNL277W(11)
1777106_at	4634.94	P	1229.04	P	3.7712	P	YNL274C	S. cerevisiae	YNL274C S	chr14:12111	Glyoxylate r	GOR1	chr14	YNL274C	---	P53839	---	---	---	0008152 //	0005634 //	0003824 //	---	---	This probe s	S000005218(11),GENEFINDER00000000728(11),GENSCAN00000005399(11),YNL274C(11)
1771738_at	418.4	P	97.38	P	4.2966	P	YNL270C	S. cerevisiae	YNL270C S	chr14:13593	Arginine trar	ALP1	chr14	YNL270C	---	P38971	---	---	---	0006810 //	0016020 //	0015171 //	---	S000005214	This probe s	S000005214(11),GENEFINDER00000000856(11),GENSCAN00000004832(11),YNL270C(11)
1776427_at	9098.71	P	2806.19	P	3.2424	P	YNL241C	S. cerevisiae	YNL241C S	chr14:19642	Glucose-6-p	ZWF1	chr14	YNL241C	---	P11412 ///	---	---	---	0005975 //	0005737 //	0003824 //	---	---	This probe s	S000005185(11),GENEFINDER00000000731(11),GENSCAN00000005275(11),YNL241C(11)
1780105_at	1388.77	P	470.36	P	2.9526	P	YNL237W	S. cerevisiae	YNL237W S	chr14:20518	Probable tyr	YTP1	chr14	YNL237W	---	P53584	---	---	---	0034605 //	0016020 //	---	---	S000005181	This probe s	S000005181(11),YNL237W(11)
1778789_at	2759.06	P	603.04	P	4.5753	P	YNL234W	S. cerevisiae	YNL234W S	chr14:21023	---	---	chr14	YNL234W	---	P53857	---	---	---	0006950 //	0005737 //	0005506 //	---	---	This probe s	S000005178(11),GENSCAN00000005412(11),YNL234W(11)
1777260_at	10269.65	P	5042.29	P	2.0367	P	YNL208W	S. cerevisiae	YNL208W S	chr14:25441	---	---	chr14	YNL208W	---	P40159	---	---	---	---	0005739 //	---	---	---	This probe s	S000005152(11),GENEFINDER00000000797(11),GENSCAN00000004978(11),YNL208W(11)
1777343_at	503.26	P	149.67	P	3.3625	P	YNL202W	S. cerevisiae	YNL202W S	chr14:25956	Peroxisomal	SPS19	chr14	YNL202W	---	P32573	---	---	---	0008152 //	0005777 //	0003824 //	---	---	This probe s	S000005146(11),GENEFINDER00000000854(11),GENSCAN00000005425(11),YNL202W(11)
1774614_at	7668.24	P	2177.02	P	3.5224	P	YNL200C	S. cerevisiae	YNL200C S	chr14:26370	---	---	chr14	YNL200C	---	P40165	---	---	---	0034605 //	0005737 //	---	---	---	This probe s	S000005144(11),GENSCAN00000004960(11),YNL200C(11)
1773046_at	155.86	P	63.23	P	2.465	P	YNL196C	S. cerevisiae	YNL196C S	chr14:27027	Sporulation-	SLZ1	chr14	YNL196C	---	P40167	---	---	---	---	---	---	---	---	This probe s	S000005140(11),GENEFINDER00000000726(11),GENSCAN00000005026(11),YNL196C(11)
1774129_at	4711.39	P	274.92	P	17.1373	P	YNL195C	S. cerevisiae	YNL195C S	chr14:27151	---	---	chr14	YNL195C	---	P40168	---	---	---	---	0005739 //	---	---	GENEFINDE	This probe s	S000005139(11),GENEFINDER00000000816(11),GENSCAN00000005259(11),YNL195C(11)
1775810_at	5362.33	P	399.55	P	13.4209	P	YNL194C	S. cerevisiae	YNL194C S	chr14:27270	---	---	chr14	YNL194C	---	P40169	---	---	---	0030437 //	0005737 //	---	---	S000005138	This probe s	S000005138(11),GENEFINDER00000000816(10),YNL194C(11)
1775916_at	1649.74	P	761.64	P	2.166	P	YNL193W	S. cerevisiae	YNL193W S	chr14:27436	---	---	chr14	YNL193W	---	P53870	---	---	---	---	---	---	---	---	This probe s	S000005137(11),GENEFINDER00000000775(11),GENSCAN00000004935(11),YNL193W(11)
1773126_at	1216.47	P	507.45	P	2.3972	P	YNL146C-A	S. cerevisiae	YNL146C-A	chr14:35138	---	---	chr14	YNL146C-A	---	Q3E7Z1	---	---	---	---	---	---	---	---	This probe s	S000028851(11),YNL146C-A(11)
1774177_at	2040.47	P	993.74	P	2.0533	P	YNL144C	S. cerevisiae	YNL144C S	chr14:35281	---	---	chr14	YNL144C	---	P53907	---	---	---	---	0005739 //	0005515 //	---	---	This probe s	S000005088(11),GENEFINDER00000000886(11),GENSCAN00000005395(11),YNL144C(11)
1777219_at	9939.27	P	4877.79	P	2.0377	P	YNL134C	S. cerevisiae	YNL134C S	chr14:37244	---	---	chr14	YNL134C	---	P53912	---	---	---	0008152 //	0005634 //	0003824 //	IPR013154	---	This probe s	S000005078(11),GENEFINDER00000000713(11),GENSCAN00000004940(11),YNL134C(11)
1775729_at	164.38	P	77.59	P	2.1186	P	YNL117W	S. cerevisiae	YNL117W S	chr14:40635	Malate synth	MLS1	chr14	YNL117W	---	P30952 ///	---	---	---	0006097 //	0005737 //	0003824 //	---	---	This probe s	S000005061(11),GENEFINDER00000000891(11),GENSCAN00000005251(11),YNL117W(11)
1776930_at	3007.24	P	1150.66	P	2.6135	P	YNL115C	S. cerevisiae	YNL115C S	chr14:41011	---	---	chr14	YNL115C	---	P53925 ///	---	---	---	---	0000324 //	---	---	S000005059	This probe s	S000005059(11),GENSCAN00000004885(11),YNL115C(11)
1773966_at	420.43	P	204.65	P	2.0544	P	YNL093W	S. cerevisiae	YNL093W S	chr14:44986	GTPase, sir	YPT53	chr14	YNL093W	---	P36019 ///	---	---	---	0006623 //	0005770 //	0000166 //	---	---	This probe s	S000005037(11),GENSCAN00000005152(11),YNL093W(11)
1778105_at	810.25	P	200.36	P	4.044	P	YNL092W	S. cerevisiae	YNL092W S	chr14:45086	---	---	chr14	YNL092W	---	P53934	---	---	---	---	---	0005515 //	---	---	This probe s	S000005036(11),GENEFINDER00000000736(11),GENSCAN00000004864(11),YNL092W(11)
1773890_at	2320.32	P	859.66	P	2.6991	P	YNL077W	S. cerevisiae	YNL077W S	chr14:48136	Putative cha	APJ1	chr14	YNL077W	---	P53940	---	---	---	0006457 //	0005634 //	0008270 //	IPR001305	---	This probe s	S000005021(11),GENEFINDER00000000684(10),GENSCAN00000005326(11),YNL077W(11)
1773150_at	2264.93	P	787.61	P	2.8757	P	YNL063W	S. cerevisiae	YNL063W S	chr14:50775	S-adenosylm	MTQ1	chr14	YNL063W	---	P53944	---	---	---	0006451 //	0005739 //	0005515 //	IPR007848	---	This probe s	S000005007(11),GENSCAN00000005224(11),YNL063W(11)
1776356_at	1569.01	P	625.17	P	2.5097	P	YNL042W	S. cerevisiae	YNL042W S	chr14:54809	Protein of ur	BOP3	chr14	YNL042W	---	P53958	---	---	---	0046689 //	0005634 //	0005515 //	---	---	This probe s	S000004987(11),GENSCAN00000005134(11),GENEFINDER00000000782(11),YNL042W(11)
1776673_at	8660.27	P	1726.99	P	5.0147	P	YNL036W	S. cerevisiae	YNL036W S	chr14:55980	Carbonic an	NCE103	chr14	YNL036W	---	P53615	---	---	---	0006979 //	0005634 //	0004089 //	---	---	This probe s	S000004981(11),U52369(10),GENSCAN00000005417(11),YNL036W(11)
1775837_at	984.03	P	450.98	P	2.182	P	YNL025C	S. cerevisiae	YNL025C S	chr14:58431	Cyclin-like c	SSN8	chr14	YNL025C	---	P47821	---	---	---	0000122 //	0005634 //	0016251 //	---	---	This probe s	S000004970(11),GENSCAN00000005327(11),YNL025C(11)
1772689_at	7506.06	P	3246.63	P	2.312	P	YNL015W	S. cerevisiae	YNL015W S	chr14:60537	Cytosolic inf	PBI2	chr14	YNL015W	---	P01095	---	---	---	0006810 //	0000324 //	0004866 //	---	---	This probe s	S000004960(11),GENEFINDER00000000780(11),GENSCAN00000005402(11),YNL015W(11)
1778884_at	586.1	P	161.03	P	3.6397	P	YNL014W	S. cerevisiae	YNL014W S	chr14:60631	Translationa	HEF3	chr14	YNL014W	---	P53978	---	---	---	0006412 //	0022626 //	0000166 //	IPR000357	---	This probe s	S000004959(11),GENEFINDER00000000680(11),GENSCAN00000005207(11),YNL014W(11)
1778518_at	162.82	P	55.25	P	2.947	P	YNL012W	S. cerevisiae	YNL012W S	chr14:60968	Meiosis-spe	SPO1	chr14	YNL012W	---	P53541	---	---	---	0007126 //	0005634 //	0004620 //	---	---	This probe s	S000004957(11),GENSCAN00000005073(11),YNL012W(11)
1770248_at	2118.94	P	932.78	P	2.2716	P	YNL011C	S. cerevisiae	YNL011C S	chr14:61183	---	---	chr14	YNL011C	---	P53980	---	---	---	---	---	---	IPR002882	GENSCAN0	This probe s	S000004956(11),GENSCAN00000004924(11),YNL011C(11)
1778706_at	1957.63	P	171.27	P	11.4301	P	YNR002C	S. cerevisiae	YNR002C S	chr14:63300	Putative trar	ATO2	chr14	YNR002C	---	P32907	---	---	---	0015696 //	0005739 //	0005515 //	---	S000005285	This probe s	S000005285(11),GENEFINDER00000000718(11),GENSCAN00000005076(11),YNR002C(11)
1779450_at	3055.5	P	1209.06	P	2.5272	P	YNR014W	S. cerevisiae	YNR014W S	chr14:65246	---	---	chr14	YNR014W	---	P53719	---	---	---	---	0005737 //	---	---	---	This probe s	S000005297(11),GENSCAN00000004847(11),YNR014W(11)
1772907_at	1175.51	P	465.32	P	2.5262	P	YNR034W	S. cerevisiae	YNR034W S	chr14:69031	Protein with	SOL1	chr14	YNR034W	---	P50278	---	---	---	0005975 //	0005634 //	0005515 //	IPR006148	---	This probe s	S000005317(11),GENEFINDER00000000828(11),GENSCAN00000005015(11),YNR034W(11)
1779897_at	10625.17	P	4126.02	P	2.5752	P	YNR034W-/	S. cerevisiae	YNR034W-/	chr14:69255	---	---	chr14	YNR034W-/	---	Q3E841	---	---	---	---	0005737 //	---	---	---	This probe s	S000007525(11),YNR034W-A(11)
1772199_at	640.69	P	271.87	P	2.3566	P	YNR058W	S. cerevisiae	YNR058W S	chr14:73428	7,8-diamino-	BIO3	chr14	YNR058W	---	P50277 ///	---	---	---	0009102 //	---	0003824 //	---	---	This probe s	S000005341(11),GENEFINDER00000000765(11),GENSCAN00000004825(11),YNR058W(11)
1779825_at	3285.6	P	603.01	P	5.4487	P	YNR065C	S. cerevisiae	YNR065C S	chr14:75034	---	---	chr14	YNR065C	---	P53751	---	---	---	---	0016020 //	0005515 //	---	S000005348	This probe s	S000005348(11),GENSCAN00000004855(11),YNR065C(11)
1769988_at	4282.06	P	1737.67	P	2.4643	P	YNR067C	S. cerevisiae	YNR067C S	chr14:75574	Daughter ce	DSE4	chr14	YNR067C	---	P53753	---	---	---	0007109 //	0005576 //	0016787 //	---	---	This probe s	S000005350(11),GENSCAN00000005199(11),YNR067C(11)
1775742_at	1915.01	P	213.48	P	8.9704	P	YNR068C	S. cerevisiae	YNR068C S	chr14:76006	---	---	chr14	YNR068C	---	P53754	---	---	---	---	---	0005515 //	---	---	This probe s	S000005351(11),GENSCAN00000004859(11),YNR068C(11)
1777389_at	232.99	P	41.98	P	5.55	P	YNR069C	S. cerevisiae	YNR069C S	chr14:76111	Protein of ur	BSC5	chr14	YNR069C	---	P53755	---	---	---	0019941 //	---	0005515 //	---	---	This probe s	S000005352(11),GENSCAN00000004977(11),YNR069C(11)
1779355_at	332.09	P	133.51	P	2.4874	P	YNR070W	S. cerevisiae	YNR070W S	chr14:76536	---	---	chr14	YNR070W	---	P53756	---	---	---	0006810 //	0005739 //	0000166 //	---	S000005353	This probe s	S000005353(11),GENSCAN00000004969(11),YNR070W(11)
1775478_at	366.09	P	96.62	P	3.789	P	YNR071C	S. cerevisiae	YNR071C S	chr14:77043	---	---	chr14	YNR071C	---	P53757	---	---	---	0005975 //	---	0003824 //	---	---	This probe s	S000005354(11),GENEFINDER00000000768(11),GENSCAN00000005140(11),YNR071C(11)
1777472_at	226.25	P	50.63	P	4.4687	P	YOL164W-/	S. cerevisiae	YOL164W-/	chr15:4129-	---	---	chr15	YOL164W-/	---	Q3E7A5	---	---	---	---	---	---	---	---	This probe s	S000028580(11),YOL164W-A(11)
1776553_at	561.23	P	30.28	P	18.5347	P	YOL163W	S. cerevisiae	YOL163W S	chr15:9595-	---	---	chr15	YOL163W	---	A2P2R4 ///	---	---	---	0006810 //	0016020 //	---	---	S000005523	This probe s	S000005523(11),YOL163W(11)
1769713_at	857.26	P	45.4	P	18.8824	P	YOL162W	S. cerevisiae	YOL162W S	chr15:10117	---	---	chr15	YOL162W	---	O08337	---	---	---	0006810 //	0016020 //	---	---	S000005522	This probe s	S000005522(11),YOL162W(11)
1777191_at	664.95	P	217.12	P	3.0626	P	YOL159C-A	S. cerevisiae	YOL159C-A	chr15:15231	---	---	chr15	YOL159C-A	---	Q3E769	---	---	---	---	0005737 //	---	---	---	This probe s	S000007627(11),YOL159C-A(11)
1777081_at	2260.27	P	778.82	P	2.9022	P	YOL159C	S. cerevisiae	YOL159C S	chr15:17279	---	---	chr15	YOL159C	---	Q08300	---	---	---	---	0005625 //	---	---	---	This probe s	S000005519(11),YOL159C(11)
1771341_at	639.62	P	243.71	P	2.6245	P	YOL154W	S. cerevisiae	YOL154W S	chr15:34656	Putative GP	ZPS1	chr15	YOL154W	---	Q12512	---	---	---	---	0000324 //	---	---	---	This probe s	S000005514(11),GENEFINDER00000002025(11),GENSCAN00000006785(11),YOL154W(11)
1772997_at	4680.68	P	1513.53	P	3.0926	P	YOL151W	S. cerevisiae	YOL151W S	chr15:43691	3-methylbuta	GRE2	chr15	YOL151W	---	Q12068	---	---	---	0006950 //	0005634 //	0003824 //	IPR002225	---	This probe s	S000005511(11),GENEFINDER00000002223(11),GENSCAN00000006640(11),YOL151W(11)
1773889_at	78.65	P	31.53	P	2.4944	P	YOL131W	S. cerevisiae	YOL131W S	chr15:73029	---	---	chr15	YOL131W	---	Q08270	---	---	---	---	---	0005515 //	---	---	This probe s	S000005491(11),YOL131W(11)
1774081_at	2892.8	P	857.27	P	3.3744	P	YOL126C	S. cerevisiae	YOL126C S	chr15:81785	Cytoplasmic	MDH2	chr15	YOL126C	---	P22133	---	---	---	0005975 //	0005737 //	0003824 //	---	---	This probe s	S000005486(11),GENSCAN00000007365(11),YOL126C(11)
1775781_at	4323.86	P	1655.61	P	2.6116	P	YOL119C	S. cerevisiae	YOL119C S	chr15:94854	Protein with	MCH4	chr15	YOL119C	---	Q08268	---	---	---	0006810 //	0000329 //	0005515 //	---	S000005479	This probe s	S000005479(11),YOL119C(11)
1772686_at	486.73	P	181.35	P	2.6839	P	YOL117W	S. cerevisiae	YOL117W S	chr15:97549	Subunit of th	RRI2	chr15	YOL117W	---	Q12348	---	---	---	0000338 //	0005634 //	0005515 //	---	---	This probe s	S000005477(11),GENEFINDER00000002137(11),GENSCAN00000007015(11),YOL117W(11)
1778184_at	504.65	P	225.88	P	2.2342	P	YOL114C	S. cerevisiae	YOL114C S	chr15:10331	---	---	chr15	YOL114C	---	Q12322	---	---	---	0006415 //	---	0003747 //	---	---	This probe s	S000005474(11),YOL114C(11)
1773699_at	875.73	P	192.65	P	4.5457	P	YOL084W	S. cerevisiae	YOL084W S	chr15:16235	Protein of ur	PHM7	chr15	YOL084W	---	Q12252	---	---	---	---	0000324 //	---	---	S000005444	This probe s	S000005444(11),GENEFINDER00000002267(11),GENSCAN00000007111(11),YOL084W(11)
1774983_at	1046.28	P	366.02	P	2.8585	P	YOL083W	S. cerevisiae	YOL083W S	chr15:16571	---	---	chr15	YOL083W	---	Q12292	---	---	---	---	---	0005515 //	---	---	This probe s	S000005443(11),GENSCAN00000007330(11),YOL083W(11)
1773230_at	1422.27	P	677.61	P	2.099	P	YOL059W	S. cerevisiae	YOL059W S	chr15:21712	NAD-depend	GPD2	chr15	YOL059W	---	P41911	---	---	---	0005975 //	0005737 //	0003824 //	---	---	This probe s	S000005420(11),GENEFINDER00000002023(11),GENSCAN00000007355(11),YOL059W(11)
1770923_at	2448.4	P	485.52	P	5.0428	P	YOL055C	S. cerevisiae	YOL055C S	chr15:22441	Multifunction	THI20	chr15	YOL055C	---	Q08224	---	---	---	0009228 //	0005829 //	0008902 //	---	---	This probe s	S000005416(11),GENEFINDER00000002222(11),GENSCAN00000007274(11),YOL055C(11)
1774354_at	13236.22	P	2872.2	P	4.6084	P	YOL052C-A	S. cerevisiae	YOL052C-A	chr15:23156	Multistress r	DDR2	chr15	YOL052C-A	---	P89113	---	---	---	0006950 //	0000324 //	---	---	---	This probe s	S000005413(11),YOL052C-A(11)
1774339_at	6452.17	P	1152.29	P	5.5994	P	YOL048C	S. cerevisiae	YOL048C S	chr15:24020	Putative pro	RRT8	chr15	YOL048C	---	Q08219	---	---	---	0034605 //	0005811 //	---	---	S000005408	This probe s	S000005408(11),YOL048C(11)
1771775_at	601.72	P	98.11	P	6.1331	P	YOL047C	S. cerevisiae	YOL047C S	chr15:24161	---	---	chr15	YOL047C	---	Q08218	---	---	---	---	0005737 //	---	---	S000005407	This probe s	S000005407(11),YOL047C(11)
1770529_at	4170.3	P	1454.84	P	2.8665	P	YOL032W	S. cerevisiae	YOL032W S	chr15:26542	Protein with	OPI10	chr15	YOL032W	---	Q08202	---	---	---	0006020 //	0005634 //	---	---	---	This probe s	S000005392(11),GENEFINDER00000002178(11),GENSCAN00000007086(11),YOL032W(11)
1770843_at	260.84	P	90.56	P	2.8803	P	YOL015W	S. cerevisiae	YOL015W S	chr15:29707	Putative pro	IRC10	chr15	YOL015W	---	Q08118	---	---	---	---	---	---	---	---	This probe s	S000005375(11),GENSCAN00000006749(11),YOL015W(11)
1777122_at	3637.05	P	1537.32	P	2.3658	P	YOL014W	S. cerevisiae	YOL014W S	chr15:29969	---	---	chr15	YOL014W	---	Q08110	---	---	---	---	---	---	---	---	This probe s	S000005374(11),YOL014W(11)
1770742_at	1196.62	P	496.25	P	2.4113	P	YOR003W	S. cerevisiae	YOR003W S	chr15:33145	Putative pre	YSP3	chr15	YOR003W	---	P25036	---	---	---	0006508 //	---	0004252 //	---	---	This probe s	S000005529(11),GENSCAN00000007118(11),YOR003W(11)
1773670_at	683.13	P	328.44	P	2.0799	P	YOR009W	S. cerevisiae	YOR009W S	chr15:34433	Cell wall ma	TIR4	chr15	YOR009W	---	Q12218	---	---	---	0006950 //	0005576 //	0005515 //	---	---	This probe s	S000005535(11),GENEFINDER00000001989(11),GENSCAN00000006948(11),YOR009W(11)
1774507_at	1604.7	P	573.97	P	2.7958	P	YOR018W	S. cerevisiae	YOR018W S	chr15:36436	Membrane r	ROD1	chr15	YOR018W	---	Q02805	---	---	---	0042493 //	0005886 //	0005515 //	---	---	This probe s	S000005544(11),GENEFINDER00000002141(11),GENSCAN00000007409(11),YOR018W(11)
1772252_at	652.3	P	221.77	P	2.9413	P	YOR019W	S. cerevisiae	YOR019W S	chr15:36812	---	---	chr15	YOR019W	---	Q99248	---	---	---	0007049 //	0005840 //	---	---	---	This probe s	S000005545(11),GENSCAN00000007087(11),YOR019W(11)
1779529_at	2972.1	P	1184.56	P	2.509	P	YOR020W-/	S. cerevisiae	YOR020W-/	chr15:37168	---	---	chr15	YOR020W-/	---	Q3E824	---	---	---	---	0005739 //	---	---	S000028526	This probe s	S000028526(11),YOR020W-A(11)
1777302_at	11519.11	P	4766.02	P	2.4169	P	YOR027W	S. cerevisiae	YOR027W S	chr15:38105	Hsp90 cocha	STI1	chr15	YOR027W	---	P15705	---	---	---	0006457 //	0005737 //	0005488 //	---	---	This probe s	S000005553(11),GENEFINDER00000002237(11),GENSCAN00000006991(11),YOR027W(11)
1770566_at	979.82	P	218.28	P	4.4888	P	YOR028C	S. cerevisiae	YOR028C S	chr15:38355	Basic leucin	CIN5	chr15	YOR028C	---	P40917	---	---	---	0006350 //	0005634 //	0003677 //	---	---	This probe s	S000005554(11),GENEFINDER00000002257(10),GENSCAN00000006775(11),YOR028C(11)
1780120_at	2637.87	P	954.09	P	2.7648	P	YOR031W	S. cerevisiae	YOR031W S	chr15:38921	Copper-bind	CRS5	chr15	YOR031W	---	P41902	---	---	---	0010038 //	---	0005507 //	---	---	This probe s	S000005557(11),YOR031W(11)
1778920_at	274.63	P	49.29	P	5.5717	P	YOR032W-/	S. cerevisiae	YOR032W-/	chr15:39217	---	---	chr15	YOR032W-/	---	Q8TGS1	---	---	---	---	0016020 //	---	---	S000028710	This probe s	S000028710(11),YOR032W-A(11)
1771460_at	1315.16	P	535.89	P	2.4542	P	YOR049C	S. cerevisiae	YOR049C S	chr15:42266	Suppressor	RSB1	chr15	YOR049C	---	Q08417	---	---	---	0006810 //	0005783 //	0004012 //	---	S000005575	This probe s	S000005575(11),GENEFINDER00000002042(10),GENSCAN00000006636(11),YOR049C(11)
1772452_at	1375.62	P	661.16	P	2.0806	P	YOR054C	S. cerevisiae	YOR054C S	chr15:42785	Functionally	VHS3	chr15	YOR054C	---	Q08438	---	---	---	0000082 //	---	0003824 //	---	---	This probe s	S000005580(11),GENEFINDER00000002263(9),YOR054C(11)
1770048_at	1749.58	P	438.24	P	3.9923	P	YOR062C	S. cerevisiae	YOR062C S	chr15:44272	---	---	chr15	YOR062C	---	P36025	---	---	---	---	0005634 //	---	---	---	This probe s	S000005588(11),GENSCAN00000006733(11),YOR062C(11)
1777383_at	3942.68	P	1839.21	P	2.1437	P	YOR092W	S. cerevisiae	YOR092W S	chr15:49512	Non-essentia	ECM3	chr15	YOR092W	---	Q99252	---	---	---	0007047 //	0005783 //	---	---	S000005618	This probe s	S000005618(11),GENEFINDER00000002095(11),GENSCAN00000007220(11),YOR092W(11)
1774778_at	469	P	131.37	P	3.5701	P	YOR100C	S. cerevisiae	YOR100C S	chr15:51329	Mitochondria	CRC1	chr15	YOR100C	---	Q12289	---	---	---	0006631 //	0005739 //	0005476 //	---	S000005626	This probe s	S000005626(11),GENSCAN00000006887(11),YOR100C(11)
1772838_at	6842.22	P	1070.07	P	6.3942	P	YOR120W	S. cerevisiae	YOR120W S	chr15:55111	Putative NAI	GCY1	chr15	YOR120W	---	P14065	---	---	---	0006979 //	0005634 //	0004032 //	---	---	This probe s	S000005646(11),GENEFINDER00000002126(11),GENSCAN00000006839(11),YOR120W(11)
1779369_at	2626.92	P	229.42	P	11.4503	P	YOR134W	S. cerevisiae	YOR134W S	chr15:57856	Rho GTPase	BAG7	chr15	YOR134W	---	Q12128	---	---	---	0007165 //	0005622 //	0005096 //	---	---	This probe s	S000005660(11),GENSCAN00000007466(11),YOR134W(11)
1771041_at	2431.87	P	761.13	P	3.1951	P	YOR152C	S. cerevisiae	YOR152C S	chr15:61751	---	---	chr15	YOR152C	---	Q99325	---	---	---	---	0005624 //	---	---	S000005678	This probe s	S000005678(11),YOR152C(11)
1778203_at	4075.51	P	1315.32	P	3.0985	P	YOR161C	S. cerevisiae	YOR161C S	chr15:63693	Protein of ur	PNS1	chr15	YOR161C	---	Q12412	---	---	---	0006810 //	0005886 //	0015220 //	---	S000005687	This probe s	S000005687(11),GENEFINDER00000002022(11),GENSCAN00000007170(11),YOR161C(11)
1775677_at	490.83	P	231.7	P	2.1184	P	YOR162C	S. cerevisiae	YOR162C S	chr15:63955	Zn2-Cys6 zi	YRR1	chr15	YOR162C	---	Q12172	---	---	---	0006350 //	0005634 //	0003677 //	---	---	This probe s	S000005688(11),GENEFINDER00000002244(11),GENSCAN00000006996(11),YOR162C(11)
1775066_at	6946.54	P	1184.2	P	5.866	P	YOR173W	S. cerevisiae	YOR173W S	chr15:65713	Non-essentia	DCS2	chr15	YOR173W	---	Q12123	---	---	---	0000184 //	0000932 //	0003824 //	---	---	This probe s	S000005699(11),GENSCAN00000006566(11),GENEFINDER00000002161(11),YOR173W(11)
1770118_at	2119.07	P	691.91	P	3.0626	P	YOR178C	S. cerevisiae	YOR178C S	chr15:66785	Regulatory s	GAC1	chr15	YOR178C	---	P28006	---	---	---	0000122 //	0000164 //	0005198 //	---	---	This probe s	S000005704(11),GENEFINDER00000002202(11),GENSCAN00000006997(11),YOR178C(11)
1778781_at	6741.53	P	2101.23	P	3.2084	P	YOR185C	S. cerevisiae	YOR185C S	chr15:68144	GTP binding	GSP2	chr15	YOR185C	---	P32836	---	---	---	0006810 //	0005634 //	0000166 //	---	---	This probe s	S000005711(11),GENEFINDER00000002138(11),GENSCAN00000006745(11),YOR185C(11)
1777402_at	407.65	P	70.96	P	5.7448	P	YOR186W	S. cerevisiae	YOR186W S	chr15:68311	---	---	chr15	YOR186W	---	Q08560	---	---	---	---	0016020 //	---	---	S000005712	This probe s	S000005712(11),GENSCAN00000006962(11),YOR186W(11)
1773923_at	475.51	P	119.49	P	3.9795	P	YOR192C	S. cerevisiae	YOR192C S	chr15:69876	Transporter	THI72	chr15	YOR192C	---	Q08579	---	---	---	0006810 //	0016020 //	0005515 //	---	S000005718	This probe s	S000005718(11),GENEFINDER00000002059(11),GENSCAN00000006649(11),YOR192C(11)
1776449_at	2082.63	P	835.57	P	2.4925	P	YOR208W	S. cerevisiae	YOR208W S	chr15:73392	Phosphotyro	PTP2	chr15	YOR208W	---	P29461	---	---	---	0000173 //	0005634 //	0004721 //	---	---	This probe s	S000005734(11),GENSCAN00000007343(11),GENEFINDER00000002007(9),YOR208W(11)
1777525_at	6485.57	P	2296.55	P	2.824	P	YOR220W	S. cerevisiae	YOR220W S	chr15:75532	Protein of ur	RCN2	chr15	YOR220W	---	Q12044	---	---	---	---	0005737 //	0005515 //	---	---	This probe s	S000005746(11),GENSCAN00000006625(11),YOR220W(11)
1769764_at	982.95	P	446.94	P	2.1993	P	YOR223W	S. cerevisiae	YOR223W S	chr15:75978	---	---	chr15	YOR223W	---	Q12015	---	---	---	---	0016020 //	---	---	S000005749	This probe s	S000005749(11),GENEFINDER00000002056(11),GENSCAN00000007178(11),YOR223W(11)
1776725_at	9035.37	P	2874.29	P	3.1435	P	YOR226C	S. cerevisiae	YOR226C S	chr15:76161	Conserved r	ISU2	chr15	YOR226C	---	Q12056	---	---	---	0006879 //	0005739 //	0005506 //	---	---	This probe s	S000005752(11),GENEFINDER00000002252(11),GENSCAN00000007128(11),YOR226C(11)
1777845_at	2160.36	P	546.01	P	3.9566	P	YOR227W	S. cerevisiae	YOR227W S	chr15:76282	Protein of ur	HER1	chr15	YOR227W	---	Q12276	---	---	---	0007029 //	0005737 //	0005515 //	---	---	This probe s	S000005753(11),GENSCAN00000007349(11),GENEFINDER00000002176(11),YOR227W(11)
1775509_at	7106.03	P	3438.95	P	2.0663	P	YOR267C	S. cerevisiae	YOR267C S	chr15:82256	Protein kina	HRK1	chr15	YOR267C	---	Q08732	---	---	---	0006468 //	0005737 //	0000166 //	---	---	This probe s	S000005793(11),GENEFINDER00000002152(11),GENSCAN00000006774(11),YOR267C(11)
1778686_at	4309.75	P	1836.53	P	2.3467	P	YOR273C	S. cerevisiae	YOR273C S	chr15:83444	Polyamine tr	TPO4	chr15	YOR273C	---	Q12256	---	---	---	0000296 //	0005774 //	0000297 //	---	S000005799	This probe s	S000005799(11),GENSCAN00000007298(11),YOR273C(11)
1778930_at	1223.9	P	375.39	P	3.2603	P	YOR289W	S. cerevisiae	YOR289W S	chr15:85335	---	---	chr15	YOR289W	---	Q12012	---	---	---	---	0005634 //	0005515 //	---	---	This probe s	S000005815(11),GENSCAN00000006661(11),YOR289W(11)
1773225_at	1974.42	P	731.46	P	2.6993	P	YOR292C	S. cerevisiae	YOR292C S	chr15:86564	---	---	chr15	YOR292C	---	Q08743	---	---	---	0034605 //	0000324 //	---	---	S000005818	This probe s	S000005818(11),YOR292C(11)
1770393_at	3380.23	P	1371.89	P	2.4639	P	YOR302W	S. cerevisiae	YOR302W S	chr15:88275	---	---	chr15	YOR302W	---	P08521	---	---	---	0006417 //	0005829 //	0005515 //	---	---	This probe s	S000005828(11),YOR302W(11)
1773073_at	2443.32	P	1016.06	P	2.4047	P	YOR306C	S. cerevisiae	YOR306C S	chr15:88986	Plasma men	MCH5	chr15	YOR306C	---	Q08777	---	---	---	0006810 //	0005886 //	0008028 //	---	S000005833	This probe s	S000005833(11),GENEFINDER00000002165(11),GENSCAN00000006773(11),YOR306C(11)
1778748_at	5202.36	P	2108.19	P	2.4677	P	YOR316C	S. cerevisiae	YOR316C S	chr15:90622	Vacuolar tra	COT1	chr15	YOR316C	---	P32798	---	---	---	0006810 //	0000324 //	0005385 //	---	S000005843	This probe s	S000005843(11),GENEFINDER00000002250(11),GENSCAN00000007322(11),YOR316C(11)
1769813_at	1209.61	P	555.51	P	2.1775	P	YOR329C	S. cerevisiae	YOR329C S	chr15:93672	Protein regu	SCD5	chr15	YOR329C	---	P34758	---	---	---	0006810 //	0000300 //	0005515 //	---	---	This probe s	S000005856(11),GENEFINDER00000002246(9),GENSCAN00000007318(11),YOR329C(11)
1769876_at	1142.25	P	473.01	P	2.4149	P	YOR338W	S. cerevisiae	YOR338W S	chr15:95689	---	---	chr15	YOR338W	---	Q99326	---	---	---	0006950 //	---	---	IPR007080	---	This probe s	S000005865(11),GENEFINDER00000002256(11),GENSCAN00000007101(11),YOR338W(11)
1771274_at	73.33	P	23.73	P	3.0902	P	YOR351C	S. cerevisiae	YOR351C S	chr15:99501	Meiosis-spe	MEK1	chr15	YOR351C	---	P24719	---	---	---	0006468 //	0005634 //	0000166 //	IPR001245	---	This probe s	S000005878(11),GENSCAN00000007451(11),YOR351C(11)
1776063_at	857.58	P	414.18	P	2.0705	P	YOR358W	S. cerevisiae	YOR358W S	chr15:10101	Subunit of th	HAP5	chr15	YOR358W	---	Q02516	---	---	---	0006109 //	0005634 //	0003677 //	---	---	This probe s	S000005885(11),GENSCAN00000006680(11),YOR358W(11)

1773280_at	887.32	P	418.47	P	2.1204	P	YOR363C	S. cerevisiae	YOR363C.S	chr15:10202	Autoregulato	PIP2	chr15	YOR363C	---	P52960	---	---	---	0006350 // t	0005634 // n	0003677 // D	---	---	This probe s	S000005890(11),GENSCAN00000007013(11),YOR363C(11)
1776624_at	7330.15	P	2470.74	P	2.9668	P	YOR374W	S. cerevisiae	YOR374W.S	chr15:10398	Mitochondria	ALD4	chr15	YOR374W	---	P46367	---	---	---	0006067 // e	0005739 // m	0004029 // a	---	---	This probe s	S000005901(11),GENSCAN00000006889(11),GENEFINDER00000002062(11),YOR374W(11)
1779593_at	1836.56	P	658.44	P	2.7893	P	YOR377W	S. cerevisiae	YOR377W.S	chr15:10462	Alcohol acet	ATF1	chr15	YOR377W	---	P40353 /// C	---	---	---	0006066 // c	0005811 // l	0004026 // a	---	---	This probe s	S000005904(11),GENSCAN00000007189(11),YOR377W(11)
1778065_at	1496.11	P	606.7	P	2.466	P	YOR380W	S. cerevisiae	YOR380W.S	chr15:10512	Transcriptio	RDR1	chr15	YOR380W	---	Q08904	---	---	---	0006350 // t	0005634 // n	0003677 // D	---	---	This probe s	S000005907(11),GENEFINDER00000002201(11),GENSCAN00000007219(11),YOR380W(11)
1773546_at	719.55	P	238.53	P	3.0166	P	YOR381W	S. cerevisiae	YOR381W.S	chr15:10555	Ferric reduc	FRE3	chr15	YOR381W	---	Q08905	---	---	---	0006810 // t	0005886 // p	0005506 // i	---	S000005908	This probe s	S000005908(11),GENEFINDER00000002122(11),GENSCAN00000007333(11),YOR381W(11)
1772681_s_at	87.88	P	17.66	P	4.9762	P	YOR394W	S. cerevisiae	YOR394W.S	chr13:92251	Putative pro	PAU21 /// P.	chr15 /// chr	YOR394W /	---	P42221	---	---	---	0006950 // r	---	---	---	S000005921	This probe s	S000005921(11),S000006203(11),GENEFINDER00000000984(11),GENSCAN00000005982(11),GENEFINDER00000002163(11),GENSCAN00000006809(11),YPL282C(11),YOR394W(11)
1772992_s_at	865.43	P	409.88	P	2.1114	P	YPL278C	S. cerevisiae	YPL278C.S	chr15:10757	---	---	chr15 /// chr	YOR389W /	---	Q08912 /// C	---	---	---	---	0005576 // e	0005515 // p	---	S000005916	This probe s	S000005916(11),S000006199(11),GENEFINDER00000001990(10),GENSCAN00000007253(11),YPL278C(11),YOR389W(11)
1774517_at	31.03	P	15.36	P	2.0202	P	YPL277C	S. cerevisiae	YPL277C.S	chr15:10742	---	---	chr16	YPL277C	---	Q08989	---	---	---	---	0005624 // m	---	---	S000006198	This probe s	S000006198(11),GENEFINDER00000000973(11),GENSCAN00000005673(11),YPL277C(11)
1775137_s_at	594.99	P	296.8	P	2.0047	P	YPL277C	S. cerevisiae	YPL277C.S	chr15:10742	---	---	chr15 /// chr	YOR389W /	---	Q08912 /// C	---	---	---	---	0005576 // e	0005515 // p	---	S000005916	This probe s	S000005916(11),S000006198(11),GENEFINDER00000000973(11),GENSCAN00000005673(11),GENEFINDER00000001990(11),GENSCAN00000007253(11),YPL277C(11),YOR389W(11)
1779399_at	5916.06	P	752.5	P	7.8619	P	YPL274W	S. cerevisiae	YPL274W.S	chr16:22937	High-affinity	SAM3	chr16	YPL274W	---	Q08986	---	---	---	0006810 // t	0005783 // e	0000095 // S	---	S000006195	This probe s	S000006195(11),GENEFINDER00000000910(9),GENSCAN00000006105(11),YPL274W(11)
1770751_at	718.99	P	330.84	P	2.1732	P	YPL272C	S. cerevisiae	YPL272C.S	chr16:26610	---	---	chr16	YPL272C	---	Q08984	---	---	---	0006066 // c	---	0004026 // a	---	---	This probe s	S000006193(11),YPL272C(11)
1777714_at	1528.56	P	737.24	P	2.0734	P	YPL265W	S. cerevisiae	YPL265W.S	chr16:41042	Dicarboxylic	DIP5	chr16	YPL265W	---	P53388	---	---	---	0006810 // t	0016020 // m	0005515 // p	IPR013216 ,	S000006186	This probe s	S000006186(11),GENEFINDER00000001120(9),GENSCAN00000005555(11),YPL265W(11)
1769474_at	968.32	P	384.41	P	2.519	P	YPL264C	S. cerevisiae	YPL264C.S	chr16:43282	---	---	chr16	YPL264C	---	Q08980	---	---	---	---	0016020 // m	---	---	S000006185	This probe s	S000006185(11),YPL264C(11)
1770700_at	1557.5	P	457.11	P	3.4073	P	YPL258C	S. cerevisiae	YPL258C.S	chr16:53497	Hydroxymetl	THI21	chr16	YPL258C	---	Q08975	---	---	---	0009228 // t	---	0005515 // p	---	---	This probe s	S000006179(11),GENEFINDER00000000933(11),GENSCAN00000005925(11),YPL258C(11)
1775201_at	869.8	P	309.73	P	2.8083	P	YPL257W	S. cerevisiae	YPL257W.S	chr16:63278	---	---	chr16	YPL257W	---	Q08974	---	---	---	0007049 // c	0016020 // m	---	---	S000006178	This probe s	S000006178(11),YPL257W(11)
1771824_at	8549.72	P	4102.41	P	2.0841	P	YPL250C	S. cerevisiae	YPL250C.S	chr16:74308	Protein of ur	ICY2	chr16	YPL250C	---	Q12048	---	---	---	---	---	---	---	---	This probe s	S000006171(11),GENEFINDER00000001015(10),GENSCAN00000006261(11),YPL250C(11)
1776302_at	5022.95	P	1065.44	P	4.7144	P	YPL247C	S. cerevisiae	YPL247C.S	chr16:82624	---	---	chr16	YPL247C	---	Q12523	---	---	---	---	0005634 // n	---	---	---	This probe s	S000006168(11),GENEFINDER00000000964(11),GENSCAN00000005534(11),YPL247C(11)
1773612_at	11136.57	P	4357.84	P	2.5555	P	YPL240C	S. cerevisiae	YPL240C.S	chr13:63235	Hsp90 chape	HSP82	chr16	YPL240C	---	P02829	---	---	---	0006457 // p	0005737 // c	0000166 // n	---	---	This probe s	S000006161(11),GENEFINDER00000001138(11),GENSCAN00000005535(11),YPL240C(11)
1775271_at	4324.09	P	767.94	P	5.6308	P	YPL230W	S. cerevisiae	YPL230W.S	chr16:11531	Putative trar	USV1	chr16	YPL230W	---	Q12132	---	---	---	0000436 // c	0005622 // i	0003704 // s	---	---	This probe s	S000006151(11),GENSCAN00000005906(11),YPL230W(11)
1775975_at	2066.71	P	808.96	P	2.5548	P	YPL224C	S. cerevisiae	YPL224C.S	chr16:12673	Putative met	MMT2	chr16	YPL224C	---	Q08970	---	---	---	0006810 // t	0005739 // m	0005506 // i	---	---	This probe s	S000006145(11),GENSCAN00000006194(11),YPL224C(11)
1769333_at	1217.59	P	99.84	P	12.1954	P	YPL223C	S. cerevisiae	YPL223C.S	chr16:12863	Hydrophilin c	GRE1	chr16	YPL223C	---	Q08969	---	---	---	0006950 // r	0005737 // c	0005515 // p	---	---	This probe s	S000006144(11),GENEFINDER00000001116(10),GENSCAN00000005867(11),YPL223C(11)
1776228_at	1242.5	P	201.8	P	6.1571	P	YPL222W	S. cerevisiae	YPL222W.S	chr16:13016	Putative pro	FMP40	chr16	YPL222W	---	Q08968	---	---	---	---	0005739 // m	0005515 // p	---	---	This probe s	S000006143(11),GENSCAN00000005762(11),GENEFINDER00000000962(11),YPL222W(11)
1773101_at	4866.8	P	957.75	P	5.0815	P	YPL214C	S. cerevisiae	YPL214C.S	chr16:14856	Bifunctional	THI6	chr16	YPL214C	---	P41835	---	---	---	0008152 // m	0005737 // c	0000166 // n	---	---	This probe s	S000006135(11),GENEFINDER00000001037(11),GENSCAN00000005965(11),YPL214C(11)
1780047_at	4477.62	P	1531.73	P	2.9232	P	YPL203W	S. cerevisiae	YPL203W.S	chr16:16625	cAMP-deper	TPK2	chr16	YPL203W	---	P06245	---	---	---	0001403 // i	0005634 // n	0000166 // n	IPR001245 ,	---	This probe s	S000006124(11),GENEFINDER00000001104(11),GENSCAN00000006006(11),YPL203W(11)
1772073_at	2513	P	741.47	P	3.3892	P	YPL196W	S. cerevisiae	YPL196W.S	chr16:17504	Protein of ur	OXR1	chr16	YPL196W	---	Q08952	---	---	---	0006979 // r	0005739 // m	---	---	---	This probe s	S000006117(11),GENSCAN00000005703(11),YPL196W(11)
1777879_at	763.76	P	360.45	P	2.1189	P	YPL191C	S. cerevisiae	YPL191C.S	chr16:18359	---	---	chr16	YPL191C	---	Q08930	---	---	---	---	0005737 // c	---	---	---	This probe s	S000006112(11),GENSCAN00000006041(11),YPL191C(11)
1774351_at	2868.14	P	1233.33	P	2.3255	P	YPL188W	S. cerevisiae	YPL188W.S	chr16:19140	Mitochondria	POS5	chr16	YPL188W	---	Q06892	---	---	---	0006741 // N	0005739 // m	0000166 // n	---	---	This probe s	S000006109(11),GENSCAN00000005988(11),YPL188W(11)
1775057_at	224.86	P	69.42	P	3.2391	P	YPL187W	S. cerevisiae	YPL187W.S	chr16:19364	Mating pher	MF(ALPHA)	chr16	YPL187W	---	P01149 /// C	---	---	---	0000750 // p	0005576 // e	0000772 // m	---	---	This probe s	S000006108(11),GENEFINDER00000001036(11),YPL187W(11)
1779057_at	2120.77	P	470.33	P	4.5091	P	YPL186C	S. cerevisiae	YPL186C.S	chr16:19451	Protein that	UIP4	chr16	YPL186C	---	Q08926	---	---	---	---	0005634 // n	0005515 // p	---	---	This probe s	S000006107(11),GENEFINDER00000000913(11),GENSCAN00000005809(11),YPL186C(11)
1775331_at	2995.6	P	76.64	P	39.0866	P	YPL171C	S. cerevisiae	YPL171C.S	chr16:22616	Conserved N	OYE3	chr16	YPL171C	---	P41816 /// C	---	---	---	0008152 // m	---	0003824 // c	---	---	This probe s	S000006092(11),GENEFINDER00000000922(11),GENSCAN00000005642(11),YPL171C(11)
1778181_at	716.91	P	202.19	P	3.5457	P	YPL166W	S. cerevisiae	YPL166W.S	chr16:23733	Autophagy-s	ATG29	chr16	YPL166W	---	Q12092	---	---	---	0006810 // t	0000407 // p	---	---	---	This probe s	S000006087(11),YPL166W(11)
1770261_at	989.64	P	370.67	P	2.6699	P	YPL165C	S. cerevisiae	YPL165C.S	chr16:23795	SET domain	SET6	chr16	YPL165C	---	Q12529	---	---	---	---	---	0016740 // t	---	---	This probe s	S000006086(11),YPL165C(11)
1776923_at	9589.75	P	3871.69	P	2.4769	P	YPL135W	S. cerevisiae	YPL135W.S	chr16:29755	Conserved p	ISU1	chr16	YPL135W	---	Q03020	---	---	---	0006879 // c	0005739 // m	0005506 // i	---	---	This probe s	S000006056(11),GENSCAN00000006051(9),YPL135W(11)
1779718_at	2534.89	P	479.14	P	5.2905	P	YPL123C	S. cerevisiae	YPL123C.S	chr16:31763	Vacuolar RN	RNY1	chr16	YPL123C	---	Q02933	---	---	---	0000902 // c	0005576 // e	0003723 // R	---	---	This probe s	S000006044(11),GENSCAN00000005964(9),YPL123C(11)
1778654_at	9606.48	P	3787.99	P	2.536	P	YPL111W	S. cerevisiae	YPL111W.S	chr16:33994	Arginase, re	CAR1	chr16	YPL111W	---	P00812	---	---	---	0006525 // a	0043332 // m	0004053 // a	---	---	This probe s	S000006032(11),GENEFINDER00000001014(11),GENSCAN00000005934(11),YPL111W(11)
1775629_at	829.1	P	411.78	P	2.0135	P	YPL109C	S. cerevisiae	YPL109C.S	chr16:34526	---	---	chr16	YPL109C	---	Q02981	---	---	---	---	0005739 // m	---	---	---	This probe s	S000006030(11),YPL109C(11)
1778500_at	2419	P	1097.34	P	2.2044	P	YPL089C	S. cerevisiae	YPL089C.S	chr16:37911	MADS-box t	RLM1	chr16	YPL089C	---	Q12224	---	---	---	0001101 // r	0005634 // n	0003677 // D	---	---	This probe s	S000006010(11),GENEFINDER00000001053(11),YPL089C(11)
1778446_at	1425.05	P	323.95	P	4.399	P	YPL088W	S. cerevisiae	YPL088W.S	chr16:38195	---	---	chr16	YPL088W	---	Q02895	---	---	---	0034605 // c	---	0005515 // p	---	---	This probe s	S000006009(11),GENEFINDER00000000953(11),GENSCAN00000006171(11),YPL088W(11)
1775990_at	6719.79	P	2799.8	P	2.4001	P	YPL087W	S. cerevisiae	YPL087W.S	chr16:38344	Alkaline dihy	YDC1	chr16	YPL087W	---	Q02896	---	---	---	0006672 // c	0000139 // G	0005515 // p	---	S000006008	This probe s	S000006008(11),YPL087W(11)
1780091_at	7253.96	P	1935.66	P	3.7475	P	YPL058C	S. cerevisiae	YPL058C.S	chr16:44583	Plasma mem	PDR12	chr16	YPL058C	---	Q02785	---	---	---	0006810 // t	0005886 // p	0000166 // n	---	S000005979	This probe s	S000005979(11),GENEFINDER00000001117(11),GENSCAN00000005683(11),YPL058C(11)
1773135_at	1088.73	P	139.16	P	7.8236	P	YPL054W	S. cerevisiae	YPL054W.S	chr16:45575	Zinc-finger p	LEE1	chr16	YPL054W	---	Q02799	---	---	---	---	---	0003676 // n	---	---	This probe s	S000005975(11),GENSCAN00000006393(11),YPL054W(11)
1770576_at	414.43	P	163.11	P	2.5408	P	YPL036W	S. cerevisiae	YPL036W.S	chr16:48283	Plasma mem	PMA2	chr16	YPL036W	---	P19657	---	---	---	0006754 // A	0005739 // m	0000166 // n	---	S000005957	This probe s	S000005957(11),GENEFINDER00000001039(11),GENSCAN00000005560(11),YPL036W(11)
1779389_at	353.43	P	88.81	P	3.9796	P	YPL018W	S. cerevisiae	YPL018W.S	chr16:51764	Outer kineto	CTF19	chr16	YPL018W	---	Q02732	---	---	---	0007049 // c	0000777 // c	0005515 // p	---	---	This probe s	S000005939(11),YPL018W(11)
1770659_at	1836.38	P	177.44	P	10.3493	P	YPL017C	S. cerevisiae	YPL017C.S	chr16:51872	Microtubule	IRC15	chr16	YPL017C	---	Q02733	---	---	---	0006312 // m	0005737 // c	0004148 // d	IPR003953 ,	---	This probe s	S000005938(11),GENEFINDER00000000971(11),GENSCAN00000006157(11),YPL017C(11)
1774678_at	2294.5	P	956.14	P	2.3998	P	YPL015C	S. cerevisiae	YPL015C.S	chr16:52580	Cytoplasmic	HST2	chr16	YPL015C	---	P53686	---	---	---	0000183 // c	0005634 // n	0008270 // z	---	---	This probe s	S000005936(11),GENEFINDER00000000911(11),GENSCAN00000006108(11),YPL015C(11)
1772812_at	3797.5	P	1490.31	P	2.5481	P	YPL006W	S. cerevisiae	YPL006W.S	chr16:54462	Vacuolar me	NCR1	chr16	YPL006W	---	Q12200	---	---	---	0006629 // l	0000329 // f	0005515 // p	---	S000005927	This probe s	S000005927(11),GENSCAN00000005633(11),YPL006W(11)
1773010_at	537.7	P	253.52	P	2.1209	P	YPL005W	S. cerevisiae	YPL005W.S	chr16:54848	Peripheral m	AEP3	chr16	YPL005W	---	Q12089	---	---	---	0016071 // m	0005737 // c	---	---	---	This probe s	S000005926(11),GENSCAN00000005854(11),YPL005W(11)
1774149_at	2177.56	P	669.95	P	3.2503	P	YPL003W	S. cerevisiae	YPL003W.S	chr16:55201	Protein that	ULA1	chr16	YPL003W	---	Q12059	---	---	---	0008152 // m	---	0000166 // n	---	---	This probe s	S000005924(11),GENEFINDER00000001124(11),GENSCAN00000005776(11),YPL003W(11)
1771260_at	1067.99	P	404.8	P	2.6383	P	YPR005C	S. cerevisiae	YPR005C.S	chr16:56578	Cytoplasmic	HAL1	chr16	YPR005C	---	A0MNQ2 ///	---	---	---	0006950 // r	0005737 // c	---	IPR000049 ,	GENEFINDE	This probe s	S000006209(11),EF015596(9),GENEFINDER00000000925(9),GENSCAN00000005691(11),YPR005C(11)
1770437_at	523.68	P	84.32	P	6.2106	P	YPR015C	S. cerevisiae	YPR015C.S	chr16:59027	---	---	chr16	YPR015C	---	Q12531	---	---	---	---	0005622 // i	0003676 // n	---	---	This probe s	S000006219(11),YPR015C(11)
1777053_at	2165.16	P	359.31	P	6.0259	P	YPR026W	S. cerevisiae	YPR026W.S	chr16:61537	Acid trehala	ATH1	chr16	YPR026W	---	P48016	---	---	---	0005975 // c	0000324 // f	0003824 // c	---	S000006230	This probe s	S000006230(11),GENEFINDER00000000900(11),GENSCAN00000005960(11),YPR026W(11)
1776173_at	1201.86	P	261.76	P	4.5915	P	YPR030W	S. cerevisiae	YPR030W.S	chr16:62787	Nuclear prot	CSR2	chr16	YPR030W	---	Q12734	---	---	---	0006350 // t	0005634 // n	0005515 // p	---	---	This probe s	S000006234(11),GENSCAN00000006134(11),GENEFINDER00000001022(11),YPR030W(11)
1769541_at	1600.25	P	642.36	P	2.4912	P	YPR042C	S. cerevisiae	YPR042C.S	chr16:65042	Member of t	PUF2	chr16	YPR042C	---	Q12221	---	---	---	0000288 // n	0005737 // c	0000166 // n	IPR000504 ,	---	This probe s	S000006246(11),GENSCAN00000005949(11),YPR042C(11)
1775041_at	838.06	P	330.03	P	2.5393	P	YPR061C	S. cerevisiae	YPR061C.S	chr16:67597	Probable Hs	JID1	chr16	YPR061C	---	Q12350	---	---	---	---	0005739 // m	0031072 // h	---	---	This probe s	S000006265(11),YPR061C(11)
1778271_at	5628.72	P	1445.6	P	3.8937	P	YPR065W	S. cerevisiae	YPR065W.S	chr16:67968	Heme-deper	ROX1	chr16	YPR065W	---	P25042	---	---	---	0000122 // n	0000228 // n	0003677 // D	---	---	This probe s	S000006269(11),GENEFINDER00000000976(11),GENSCAN00000005545(11),YPR065W(11)
1778008_at	72.13	P	33.7	P	2.1404	P	YPR078C	S. cerevisiae	YPR078C.S	chr16:69714	---	---	chr16	YPR078C	---	Q06813	---	---	---	---	---	---	---	---	This probe s	S000006282(11),GENSCAN00000005802(11),YPR078C(11)
1771319_at	639.03	P	269.28	P	2.3731	P	YPR093C	S. cerevisiae	YPR093C.S	chr16:71955	Ubiquitin liga	ASR1	chr16	YPR093C	---	Q06834	---	---	---	0045471 // r	0005634 // n	0004842 // u	---	---	This probe s	S000006297(11),GENEFINDER00000001111(11),GENSCAN00000005573(11),YPR093C(11)
1769671_at	2659.79	P	1221.68	P	2.1772	P	YPR117W	S. cerevisiae	YPR117W.S	chr16:76002	---	---	chr16	YPR117W	---	Q06116	---	---	---	---	0016020 // m	---	---	S000006321	This probe s	S000006321(11),GENEFINDER00000001076(10),GENSCAN00000005831(11),YPR117W(11)
1780073_at	6447.12	P	1584.66	P	4.0685	P	YPR127W	S. cerevisiae	YPR127W.S	chr16:79007	---	---	chr16	YPR127W	---	Q06494	---	---	---	0055114 // o	0005634 // n	0016491 // o	---	---	This probe s	S000006331(11),GENEFINDER00000001102(11),GENSCAN00000006116(11),YPR127W(11)
1774742_at	6522.13	P	1986.81	P	3.2827	P	YPR157W	S. cerevisiae	YPR157W.S	chr16:84125	---	---	chr16	YPR157W	---	Q06466	---	---	---	0006810 // t	0016020 // m	---	---	---	This probe s	S000006361(11),GENEFINDER00000000992(11),GENSCAN00000005519(11),YPR157W(11)
1777476_at	2832.71	P	1023.74	P	2.767	P	YPR158W	S. cerevisiae	YPR158W.S	chr16:84325	Protein of ur	CUR1	chr16	YPR158W	---	Q06469	---	---	---	---	0005634 // n	---	---	---	This probe s	S000006362(11),GENSCAN00000006139(11),YPR158W(11)
1779850_at	9364.79	P	4090.58	P	2.2894	P	YPR160W	S. cerevisiae	YPR160W.S	chr16:86129	Non-essentia	GPH1	chr16	YPR160W	---	P06738	---	---	---	0005975 // c	0005737 // c	0004645 // p	---	---	This probe s	S000006364(11),GENEFINDER00000000983(11),GENSCAN00000006227(11),YPR160W(11)
1779892_at	1856.61	P	188.34	P	9.8578	P	YPR167C	S. cerevisiae	YPR167C.S	chr16:87684	3'-phosphoa	MET16	chr16	YPR167C	---	P18408	---	---	---	0000103 // s	0005622 // i	0004604 // p	---	---	This probe s	S000006371(11),GENEFINDER00000000938(11),GENSCAN00000005974(11),YPR167C(11)
1779981_at	284.69	P	135.64	P	2.0989	P	YPR168W	S. cerevisiae	YPR168W.S	chr16:87806	Subunit of th	NUT2	chr16	YPR168W	---	Q06213	---	---	---	0006350 // t	0000119 // m	0005515 // p	---	---	This probe s	S000006372(11),GENEFINDER00000000904(11),GENSCAN00000006088(11),YPR168W(11)
1769968_at	6123.15	P	1059.99	P	5.7766	P	YPR184W	S. cerevisiae	YPR184W.S	chr16:90203	Glycogen de	GDB1	chr16	YPR184W	---	Q06625	---	---	---	0005975 // c	0005737 // c	0003824 // c	---	---	This probe s	S000006388(11),GENEFINDER00000001131(11),GENSCAN00000006320(11),YPR184W(11)
1780248_at	311.06	P	80.81	P	3.8493	P	YPR192W	S. cerevisiae	YPR192W.S	chr16:92185	Spore-speci	AQY1	chr16	YPR192W	---	P53386 /// C	---	---	---	0006810 // t	0005783 // e	0005215 // t	---	S000006396	This probe s	S000006396(11),GENEFINDER00000001095(11),GENSCAN00000006173(11),YPR192W(11)
1776410_at	630.67	P	174.01	P	3.6243	P	YPR196W	S. cerevisiae	YPR196W.S	chr16:93136	---	---	chr16	YPR196W	---	Q06595	---	---	---	0006350 // t	0005634 // n	0003677 // D	---	---	This probe s	S000006400(11),GENSCAN00000006203(11),GENEFINDER00000000995(11),YPR196W(11)
1775803_at	8.45	P	4.18	A	2.0215	P	SPAC1A6.0	S. pombe S	SPAC1A6.0	chromosome	sequence or	SPAC1A6.0	---	---	2542601	Q13859	---	NP_593200	NM_001018	0007126 // m	0005634 // n	---	---	---	This probe s	NM_001018596(11)
1774834_at	359.1	P	161.17	A	2.2281	P	SPBC1683.(	S. pombe S	SPBC1683.(	chromosome	glycosyl hyd	SPBC1683.(	---	---	2540167	Q9P6J6	---	NP_595060	NM_001020	0005975 // c	0005737 // c	0004553 // h	---	---	This probe s	NM_001020966(11)
1775604_at	35.91	P	16.72	P	2.1477	P	SPBC1A4.0	S. pombe S	SPBC1A4.0	chromosome	U3 snoRNP-	SPBC1A4.0	---	---	2540796	O74340	---	NP_595809	NM_001021	0006364 // r	0005634 // n	0030515 // s	---	---	This probe s	NM_001021712(11)
1771563_at	22.97	P	11.35	A	2.0238	P	SPCC970.0	S. pombe S	SPCC970.0	chromosome	cytochrome	SPCC970.0	---	---	2539526	O74557	EC:1.6.2.2	NP_587852	NM_001022	0006886 // i	0005739 // m	0004128 // c	---	SPCC970.0	This probe s	NM_001022845(11)
1780239_at	24.42	P	9.68	A	2.5227	P	SPAC6B12.(	S. pombe S	SPAC6B12.(	chromosome	ubiquitin-pro	SPAC6B12.(	---	---	2543315	O14212	---	NP_593762	NM_001019	0016567 // p	0005737 // c	0004842 // u	---	---	This probe s	NM_001019192(11)
1779750_at	209.15	P	82.32	P	2.5407	P	SPCC1442.(	S. pombe S	SPCC1442.(	chromosome	ubiquitin/met	SPCC1442.(	---	---	2538751	O94580	---	NP_588321	NM_001023	0006464 // p	0005634 // n	0008237 // m	---	---	This probe s	NM_001023312(11)
1774911_at	48.51	P	5.19	A	9.3468	P	SPBC365.0	S. pombe S	SPBC365.0	chromosome	protoheme I	cox10	---	---	2540944	Q9Y7Y4	---	NP_596031	NM_001021	0006783 // h	0005739 // m	0004659 // p	---	SPBC365.0	This probe s	NM_001021941(11)
1770647_at	18.86	P	8.09	A	2.3313	P	SPAC144.1	S. pombe S	SPAC144.1	chromosome	nucleotide su	SPAC144.1	---	---	2542850	Q9UTK8	---	NP_594679	NM_001020	0006810 // t	0000139 // G	0005338 // n	IPR004853 ,	SPAC144.1	This probe s	NM_001020108(11)
1770723_at	81.76	P	39.28	P	2.0815	P	SPCC1450.(	S. pombe S	SPCC1450.(	chromosome	bromodoma	SPCC1450.(	---	---	2538955	Q9Y7N0	---	NP_588301	NM_001023	0006338 // c	0000812 // S	---	---	---	This probe s	NM_001023291(11)
1779249_at	1077.53	P	2508.37	P	0.4296	P	YAL059W	S. cerevisiae	YAL059W.S	chr1:36509-	Protein of ur	ECM1	chr1	YAL059W	---	P39715 /// C	---	---	---	0000055 // r	0005634 // n	---	---	---	This probe s	S000000055(11),GENEFINDER00000000089(11),GENSCAN00000003314(11),YAL059W(11)
1779169_at	268.57	P	1132.68	P	0.2371	P	YAL025C	S. cerevisiae	YAL025C.S	chr1:100226	Essential nu	MAK16	chr1	YAL025C	---	P10962	---	---	---	0000463 // m	0005634 // n	---	---	---	This probe s	S000000023(11),GENEFINDER00000000120(11),GENSCAN00000003268(11),YAL025C(11)
1779662_at	474.43	P	1033.45	P	0.4591	P	YAL019W	S. cerevisiae	YAL019W.S	chr1:114920	Protein who	FUN30	chr1	YAL019W	---	P31380	---	---	---	0051276 // c	0005634 // n	0000166 // n	IPR006935 ,	---	This probe s	S000000017(11),GENEFINDER00000000126(11),GENSCAN00000003278(11),YAL019W(11)
1771711_at	319.9	P	695.18	P	0.4602	P	YAR008W	S. cerevisiae	YAR008W.S	chr1:158965	Subunit of th	SEN34	chr1	YAR008W	---	P39707	---	---	---	0000379 // t	0000214 // t	0000213 // t	IPR006677 ,	---	This probe s	S000000066(11),GENSCAN00000003265(11),YAR008W(11)
1776525_at	450.67	P	1122.14	P	0.4016	P	YAR018C	S. cerevisiae	YAR018C.S	chr1:170390	Nonessentia	KIN3	chr1	YAR018C	---	P22209	---	---	---	0006468 // p	---	0000166 // n	IPR001245 ,	---	This probe s	S000000071(11),GENSCAN00000003286(11),YAR018C(11)
1775992_at	1343.6	P	3029.51	P	0.4435	P	YBL098W	S. cerevisiae	YBL098W.S	chr2:39136-	Kynurenine 3	BNA4	chr2	YBL098W	---	P38169	---	---	---	0008152 // m	0005739 // m	0004497 // m	IPR001327 ,	---	This probe s	S000000194(11),GENEFINDER00000001350(11),GENSCAN00000005493(11),YBL098W(11)
1772894_at	37.83	P	115.18	P	0.3284	P	YBL097W	S. cerevisiae	YBL097W.S	chr2:40822-	Subunit of th	BRN1	chr2	YBL097W	---	P38170	---	---	---	0000070 // m	0000799 // n	0005515 // p	---	---	This probe s	S000000193(11),GENEFINDER00000001467(11),GENSCAN00000005010(11),YBL097W(11)
1777016_at	650.77	P	1423.9	P	0.457	P	YBL081W	S. cerevisiae	YBL081W.S	chr2:71859-	---	---	chr2	YBL081W	---	P38180	---	---	---	---	---	0005515 // p	---	---	This probe s	S000000177(11),GENEFINDER00000001523(11),GENSCAN00000005258(11),YBL081W(11)
1775222_at	205.33	P	548.3	P	0.3745	P	YBL052C	S. cerevisiae	YBL052C.S	chr2:119340	Histone acet	SAS3	chr2	YBL052C	---	P34218	---	---	---	0006348 // c	0005634 // n	0004402 // h	---	---	This probe s	S000000148(11),GENEFINDER00000001409(11),GENSCAN00000005014(11),YBL052C(11)
1779256_at	1469.16	P	3973.32	P	0.3698	P	YBL042C	S. cerevisiae	YBL042C.S	chr2:138302	High affinity	FUI1	chr2	YBL042C	---	P38196	---	---	---	0006810 // t	0005886 // p	0005515 // p	---	S000000138	This probe s	S000000138(11),GENEFINDER00000001366(11),GENSCAN00000005456(11),YBL042C(11)
1769602_at	140.96	P	388.1	P	0.3632	P	YBL031W	S. cerevisiae	YBL031W.S	chr2:161660	Mitotic spind	SHE1	chr2	YBL031W	---	P38200	---	---	---	0000022 // m	0005737 // c	---	---	---	This probe s	S000000127(11),GENSCAN00000005457(11),YBL031W(11)
1776524_at	581.18	P	1646.6	P	0.353	P	YBL028C	S. cerevisiae	YBL028C.S	chr2:167479	---	---	chr2	YBL028C	---	P38202	---	---	---	---	0005634 // n	0005515 // p	---	---	This probe s	S000000124(11),GENSCAN00000005347(11),YBL028C(11)
1772084_at	692.33	P	1463.46	P	0.4731	P	YBL016W	S. cerevisiae	YBL016W.S	chr2:192412	Mitogen-acti	FUS3	chr2	YBL016W	---	P16892	---	---	---	0000746 // c	0005634 // n	0000166 // n	IPR001245 ,	---	This probe s	S000000112(11),GENEFINDER00000001440(11),GENSCAN00000005287(11),YBL016W(11)
1773826_at	232.75	P	750.2	P	0.3103	P	YBL009W	S. cerevisiae	YBL009W.S	chr2:207154	Protein kina	ALK2	chr2	YBL009W	---	P32789	---	---	---	0006468 // p	---	0000166 // n	---	---	This probe s	S000000105(11),GENSCAN00000004910(11),YBL009W(11)
1775235_at	2115	P	5240.78	P	0.4036	P	YBL003C	S. cerevisiae	YBL003C.S	chr4:915520	Histone H2A	HTA2	chr2	YBL003C	---	P04912	---	---	---	0006281 // D	0000786 // n	0003677 // D	IPR003958 ,	---	This probe s	S000000099(11),GENSCAN00000005117(11),YBL003C(11)
1777846_at	3430.32	P	7507.13	P	0.4569	P	YBL002W	S. cerevisiae	YBL002W.S	chr2:236453	Histone H2B	HTB2	chr2	YBL002W	---	P02294	---	---	---	0006333 // c	0000786 // n	0003677 // D	---	---	This probe s	S000000098(11),GENEFINDER00000001527(11),GENSCAN00000005437(11),YBL002W(11)
1770101_at	559.22	P	1120.52	P	0.4991	P	YBR028C	S. cerevisiae	YBR028C.S	chr2:294383	---	---	chr2	YBR028C	---	P38070	---	---	---	0006468 // p	0005737 // c	0000166 // n	IPR001245 ,	---	This probe s	S000000232(11),GENEFINDER00000001425(11),GENSCAN00000005397(11),YBR028C(11)
1779625_at	369.08	P	762.01	P	0.4844	P	YBR030W	S. cerevisiae	YBR030W.S	chr2:298250	Ribosomal N	RKM3	chr2	YBR030W	---	P38222	---	---	---	0018026 // p	0005634 // n	0008168 // m	---	---	This probe s	S000000234(11),GENEFINDER00000001380(11),GENSCAN00000005074(11),YBR030W(11)
1777567_at	1480.43	P	3311.2	P	0.4471	P	YBR034C	S. cerevisiae	YBR034C.S	chr2:304888	Nuclear SAM	HMT1	chr2	YBR034C	---	P38074	---	---	---	0006406 // m	0005634 // n	0005515 // p	IPR007848 ,	---	This probe s	S000000238(11),GENEFINDER00000001417(11),GENSCAN00000005205(11),YBR034C(11)
1778194_at	391.23	P	874.42	P	0.4474	P	YBR038W	S. cerevisiae	YBR038W.S	chr2:311855	Chitin syntha	CHS2	chr2	YBR038W	---	P14180	---	---	---	0000910 // c	0005935 // c	0004100 // c	---	S000000242	This probe s	S000000242(11),GENSCAN00000005379(11),YBR038W(11)
1772126_at	59.44	P	1288.15	P	0.0461	P	YBR040W	S. cerevisiae	YBR040W.S	chr2:316926	Integral mer	FIG1	chr2	YBR040W	---	P38224	---	---	---	0000753 // c	0009277 // f	0005515 // p	---	S000000244	This probe s	S000000244(11),YBR040W(11)
1776697_at	597.46	P	1319.92	P	0.4526	P	YBR061C	S. cerevisiae	YBR061C.S	chr2:364745	2'-O-ribose	TRM7	chr2	YBR061C	---	P38238	---	---	---	0006412 // t	0005737 // c	0003676 // n	---	---	This probe s	S000000265(11),GENSCAN00000005336(11),YBR061C(11)
1774691_at	1608.7	P	4290.93	P	0.3749	P	YBR088C	S. cerevisiae	YBR088C.S	chr2:424945	Proliferating	POL30	chr2	YBR088C	---	P15873	---	---	---	0000710 // m	0005634 // n	0003677 // D	---	---	This probe s	S000000292(11),GENEFINDER00000001397(11),GENSCAN00000005245(11),YBR088C(11)
1773910_at	569.17	P	1188.86	P	0.4788	P	YBR091C	S. cerevisiae	YBR091C.S	chr2:427110	Essential pro	TIM12	chr2	YBR091C	---	P32830	---	---	---	0006626 // p	0005739 // m	0008270 // z	---	---	This probe s	S000000295(11),YBR091C(11)
1774710_at	633.18	P	1317.82	P	0.4805	P	YBR104W	S. cerevisiae	YBR104W.S	chr2:449622	Mitochondria	YMC2	chr2	YBR104W	---	P38087	---	---	---	0006810 // t	0005739 // m	0005342 // o	---	---	This probe s	S000000308(11),GENSCAN00000005077(11),YBR104W(11)
1770892_at	149.63	P	316.22	P	0.4732	P	YBR138C	S. cerevisiae	YBR138C.S	chr2:513716	---	---	chr2	YBR138C	---	P38277	---	---	---	---	0005737 // c	---	---	---	This probe s	S000000342(11),GENSCAN00000005453(11),YBR138C(11)
1770074_at	323.33	P	792.65	P	0.4079	P	YBR141C	S. cerevisiae	YBR141C.S	chr2:526979	---	---	chr2	YBR141C	---	P38278	---	---	---	---	0005634 // n	---	---	---	This probe s	S000000345(11),GENSCAN00000005233(11),YBR141C(11)
1769852_at	1183.71	P	2606.93	P	0.4541	P	YBR142W	S. cerevisiae	YBR142W.S	chr2:528271	Essential nu	MAK5	chr2	YBR142W	---	P38112	---	---	---	0000027 // r	0005634 // n	0000166 // n	---	---	This probe s	S000000346(11),GENEFINDER00000001367(11),GENSCAN00000005131(11),YBR142W(11)
1777367_at	224.27	P	454.06	P	0.4939	P	YBR156C	S. cerevisiae	YBR156C.S	chr2:551058	Subunit of th	SLI15	chr2	YBR156C	---	P38283	---	---	---	0006468 // p	0000777 // c	0005515 // p	---	---	This probe s	S000000360(11),GENEFINDER00000001503(11),GENSCAN00000005440(11),YBR156C(11)
1775738_at	36.76	P	92.37	P	0.398	P	YBR184W	S. cerevisiae	YBR184W.S	chr2:597317	---	---	chr2	YBR184W	---	P38299	---	---	---	---	---	---	---	---	This probe s	S000000388(11),GENSCAN00000005478(11),YBR184W(11)
1774743_at	236.56	P	484.6	P	0.4882	P	YBR233W-A	S. cerevisiae	YBR233W-A	chr2:684931	Essential su	DAD3	chr2	YBR233W-A	---	P69850	---	---	---	0007049 // c	0000776 // k	0005515 // p	---	---	This probe s	S000007595(11),YBR233W-A(11)
1774721_at	707.89	P	1914.16	P	0.3698	P	YBR247C	S. cerevisiae	YBR247C.S	chr2:712958	Protein asso	ENP1	chr2	YBR247C	---	P38333	---	---	---	0000447 // e	0005634 // n	0005515 // p	---	---	This probe s	S000000451(11),GENEFINDER00000001343(11),GENSCAN00000005097(11),YBR247C(11)
1778279_at	1427.74	P	2978.04	P	0.4794	P	YBR265W	S. cerevisiae	YBR265W.S	chr2:738536	3-ketosphing	TSC10	chr2	YBR265W	---	P38342	---	---	---	0006666 // 3	0005737 // c	0003824 // c	NULL // NUI	S000000469	This probe s	S000000469(11),GENEFINDER00000001453(11),GENSCAN00000005033(11),YBR265W(11)
1771650_at	517.36	P	1593.2	P	0.3247	P	YBR267W	S. cerevisiae	YBR267W.S	chr2:739795	Cytoplasmic	REI1	chr2	YBR267W	---	P38344	---	---	---	0000278 // m	0005622 // i	0005515 // p	---	---	This probe s	S000000471(11),GENEFINDER00000001534(11),GENSCAN00000005168(11),YBR267W(11)
1774053_at	364.16	P	744.37	P	0.4892	P	YBR271W	S. cerevisiae	YBR271W.S	chr2:744804	---	---	chr2	YBR271W	---	P38347	---	---	---	---	0005737 // c	0005515 // p	---	---	This probe s	S000000475(11),GENSCAN00000005492(11),YBR271W(11)
1771324_at	1059.09	P	2144.48	P	0.4939	P	YCL059C	S. cerevisiae	YCL059C.S	chr3:22428-	Essential nu	KRR1	chr3	YCL059C	---	P25586	---	---	---	0000447 // e	0005634 // n	0003723 // R	IPR004088 ,	---	This probe s	S000000564(11),GENEFINDER00000000022(9),GENSCAN00000003690(11),YCL059C(11)
1770503_at	75.03	P	177.08	P	0.4237	P	YCL058C	S. cerevisiae	YCL058C.S	chr3:23522-	Protein of ur	FYV5	chr3	YCL058C	---	P25585	---	---	---	0007047 // c	0005576 // e	---	---	S000000563	This probe s	S000000563(11),YCL058C(11)
1778355_at	301.24	P	1183.7	P	0.2545	P	YCL055W	S. cerevisiae	YCL055W.S	chr3:27928-	Transcriptio	KAR4	chr3	YCL055W	---	P25583	---	---	---	0000080 // G	0005634 // n	0003700 // t	IPR007757 ,	---	This probe s	S000000560(11),GENSCAN00000003655(11),YCL055W(11)
1769709_at	1118.95	P	3119.51	P	0.3587	P	YCL054W	S. cerevisiae	YCL054W.S	chr3:31448-	AdoMet-der	SPB1	chr3	YCL054W	---	P25582	---	---	---	0000463 // m	0005634 // n	0003676 // n	---	---	This probe s	S000000559(11),GENEFINDER00000000063(11),GENSCAN00000003641(11),YCL054W(11)
1779541_at	406.69	P	1214.95	P	0.3347	P	YCL036W	S. cerevisiae	YCL036W.S	chr3:59025-	Protein of ur	GFD2	chr3	YCL036W	---	P25370	---	---	---	---	---	0003676 // n	---	---	This probe s	S000000541(11),GENEFINDER00000000074(11),GENSCAN00000003715(11),YCL036W(11)
1771752_at	637.68	P	2055.62	P	0.3102	P	YCL027W	S. cerevisiae	YCL027W.S	chr3:71802-	Membrane p	FUS1	chr3	YCL027W	---	P11710	---	---	---	0000747 // c	0005886 // p	0005515 // p	IPR001452 ,	S000000532	This probe s	S000000532(11),GENSCAN00000003741(11),GENEFINDER00000000039(11),YCL027W(11)
1776483_at	475.9	P	983.78	P	0.4837	P	YCL014W	S. cerevisiae	YCL014W.S	chr3:96279-	Protein invol	BUD3	chr3	YCL014W	---	P25558	---	---	---	0000282 // c	0000142 // c	0005089 // R	---	---	This probe s	S000000520(11),GENEFINDER00000000059(11),GENSCAN00000003693(11),YCL014W(11)
1774914_at	446.07	P	1246.71	P	0.3578	P	YCR016W	S. cerevisiae	YCR016W.S	chr3:143627	---	---	chr3	YCR016W	---	P25617	---	---	---	---	0005634 // n	0005515 // p	---	---	This probe s	S000000609(11),GENSCAN00000003649(11),YCR016W(11)
1769860_at	22.07	P	94.69	P	0.2331	P	YCR020W-B	S. cerevisiae	YCR020W-B	chr3:155313	Component	HTL1	chr3	YCR020W-B	---	Q9URQ5	---	---	---	0000086 // G	0005634 // n	0003682 // c	---	---	This probe s	S000006439(11),YCR020W-B(11)
1779254_at	420.89	P	1069.04	P	0.3937	P	YCR047C	S. cerevisiae	YCR047C.S	chr3:210709	Protein invol	BUD23	chr3	YCR047C	---	P25627	---	---	---	0000056 // r	0005634 // n	0008168 // m	---	---	This probe s	S000000643(11),GENEFINDER00000000081(11),GENSCAN00000003628(11),YCR047C(11)
1771819_at	576.72	P	1417.48	P	0.4069	P	YCR057C	S. cerevisiae	YCR057C.S	chr3:220448	Conserved 9	PWP2	chr3	YCR057C	---	P25635	---	---	---	0000447 // e	0005634 // n	0005515 // p	---	---	This probe s	S000000653(11),GENSCAN00000003620(11),YCR057C(11)
1772793_at	632.66	P	1456.24	P	0.4344	P	YCR065W	S. cerevisiae	YCR065W.S	chr3:229301	Forkhead tra	HCM1	chr3	YCR065W	---	P25364	---	---	---	0000115 // r	0000790 // n	0003677 // D	---	---	This probe s	S000000661(11),GENEFINDER00000000016(11),GENSCAN00000003716(11),YCR065W(11)
1779136_at	684.92	P	2178.32	P	0.3144	P	YCR072C	S. cerevisiae	YCR072C.S	chr3:240796	WD-repeat p	RSA4	chr3	YCR072C	---	P25382	---	---	---	0000027 // r	0005730 // n	---	---	---	This probe s	S000000668(11),GENSCAN00000003661(11),YCR072C(11)
1775095_at	759.46	P	1914.61	P	0.3967	P	YCR089W	S. cerevisiae	YCR089W.S	chr3:267426	Cell wall adh	FIG2	chr3	YCR089W	---	P25653	---	---	---	0000746 // c	0005576 // e	0005515 // p	---	---	This probe s	S000000685(11),GENSCAN00000003599(11),GENEFINDER00000000046(11),YCR089W(11)
1773646_at	279.27	P	725.8	P	0.3848	P	YDL240W	S. cerevisiae	YDL240W.S	chr4:22822-	Putative GTI	LRG1	chr4	YDL240W	---	P35688	---	---	---	0007165 // s	0005622 // i	0005096 // G	---	---	This probe s	S000002399(11),GENSCAN00000007906(11),GENEFINDER00000002834(11),YDL240W(11)
1778270_at	404.94	P	1220.38	P	0.3318	P	YDL227C	S. cerevisiae	YDL227C.S	chr4:46271-	Site-specific	HO	chr4	YDL227C	---	P09932	---	---	---	0000728 // c	0005634 // n	0003677 // D	IPR007868 ,	---	This probe s	S000002386(11),GENEFINDER00000002558(11),GENSCAN00000007869(11),YDL227C(11)
1778331_at	151.61	P	460.23	P	0.3294	P	YDL211C	S. cerevisiae	YDL211C.S	chr4:79294-	---	---	chr4	YDL211C	---	Q12121	---	---	---	---	0000324 // f	---	---	S000002370	This probe s	S000002370(11),GENSCAN00000007682(11),YDL211C(11)
1772827_at	938.94	P	1986.68	P	0.4726	P	YDL201W	S. cerevisiae	YDL201W.S	chr4:99561-	Subunit of a	TRM8	chr4	YDL201W	---	Q12009	---	---	---	0006400 // t	0005634 // n	0005515 // p	---	---	This probe s	S000002360(11),GENEFINDER00000002688(11),GENSCAN00000008102(11),YDL201W(11)
1769431_at	617.9	P	1417.26	P	0.436	P	YDL167C	S. cerevisiae	YDL167C.S	chr4:160995	Putative RNA	NRP1	chr4	YDL167C	---	P32770	---	---	---	---	0005622 // i	0000166 // n	---	---	This probe s	S000002326(11),GENSCAN00000008262(11),YDL167C(11)
1778315_at	851.76	P	1774.79	P	0.4799	P	YDL166C	S. cerevisiae	YDL166C.S	chr4:163449	Essential NT	FAP7	chr4	YDL166C	---	Q12055 /// C	---	---	---	0000056 // r	0005634 // n	0000166 // n	---	---	This probe s	S000002325(11),GENEFINDER00000002603(11),GENSCAN00000008028(11),YDL166C(11)
1774967_at	364.71	P	930.87	P	0.3918	P	YDL150W	S. cerevisiae	YDL150W.S	chr4:183343	RNA polyme	RPC53	chr4	YDL150W	---	P25441	---	---	---	0006350 // t	0005634 // n	0003677 // D	---	---	This probe s	S000002309(11),GENEFINDER00000002613(11),GENSCAN00000007700(11),YDL150W(11)
1770876_at	583.29	P	1516.65	P	0.3846	P	YDL148C	S. cerevisiae	YDL148C.S	chr4:188154	Nucleolar pr	NOP14	chr4	YDL148C	---	Q99207	---	---	---	0000447 // e	0005634 // n	0005515 // p	---	---	This probe s	S000002307(11),GENSCAN00000007855(11),YDL148C(11)
1773886_at	222.87	P	577.81	P	0.3857	P	YDL127W	S. cerevisiae	YDL127W.S	chr4:234926	G1 cyclin, a	PCL2	chr4	YDL127W	---	P25693	---	---	---	0000079 // r	0000131 // i	0005515 // p	---	---	This probe s	S000002285(11),YDL127W(11)
1770514_at	1078.18	P	2167.73	P	0.4974	P	YDL112W	S. cerevisiae	YDL112W.S	chr4:258913	2'-O-ribose	TRM3	chr4	YDL112W	---	Q07527	---	---	---	0006396 // R	0005737 // c	0003723 // R	---	---	This probe s	S000002270(11),GENSCAN00000008178(11),GENEFINDER00000002857(11),YDL112W(11)
1779349_at	343.82	P	722.44	P	0.4759	P	YDL102W	S. cerevisiae	YDL102W.S	chr4:276870	Catalytic sub	POL3	chr4	YDL102W	---	P15436	---	---	---	0006139 // n	0005634 // n	0000166 // n	---	---	This probe s	S000002260(11),GENEFINDER00000002877(11),GENSCAN00000007797(11),YDL102W(11)
1774694_at	1136.85	P	2464.72	P	0.4612	P	YDL063C	S. cerevisiae	YDL063C.S	chr4:338270	---	---	chr4	YDL063C	---	Q07395	---	---	---	---	0005634 // n	0005488 // b	---	---	This probe s	S000002221(11),GENEFINDER00000002771(11),GENSCAN00000008298(11),YDL063C(11)

1771012_at	968.22	P	2265.76	P	0.4273	P	YDL060W	S. cerevisiae	YDL060W S	chr4:341617	Protein regu	TSR1	chr4	YDL060W	---	Q07381	---	---	---	0006364 // r	0005634 // r	0005515 // r	---	---	This probe s	S000002218(11),GENEFINDER00000002743(9),GENSCAN00000008198(11),YDL060W(11)
1776844_at	250.35	P	715.83	P	0.3497	P	YDL038C	S. cerevisiae	YDL038C S	chr4:382325	---	---	---	YDL038C	---	---	---	---	---	---	---	---	---	---	This probe s	S000002196(11),GENEFINDER00000002898(11),GENSCAN00000008269(11),YDL038C(11)
1771133_at	1191.08	P	2717.83	P	0.4382	P	YDL031W	S. cerevisiae	YDL031W S	chr4:394212	Putative ATI	DBP10	chr4	YDL031W	---	Q12389	---	---	---	0000027 // r	0005634 // r	0000166 // r	---	---	This probe s	S000002189(11),GENEFINDER00000002665(11),GENSCAN00000007832(11),YDL031W(11)
1775495_at	432.44	P	1231.53	P	0.3511	P	YDL003W	S. cerevisiae	YDL003W S	chr4:444678	Essential su	MCD1	chr4	YDL003W	---	Q12158	---	---	---	0006473 // r	0000228 // r	0003682 // c	---	---	This probe s	S000002161(11),GENEFINDER00000002594(11),GENSCAN00000007960(11),YDL003W(11)
1776596_at	1852.25	P	6017.32	P	0.3078	P	YDR019C	S. cerevisiae	YDR019C S	chr4:484157	T subunit of	GCV1	chr4	YDR019C	---	P48015	---	---	---	0006544 // c	0005737 // c	0004047 // a	---	---	This probe s	S000002426(11),GENEFINDER00000002873(10),GENSCAN00000008224(11),YDR019C(11)
1776348_at	292.73	P	1044.71	P	0.2802	P	YDR021W	S. cerevisiae	YDR021W S	chr4:486798	Nucleolar pro	FAL1	chr4	YDR021W	---	Q12099	---	---	---	0006364 // r	0005634 // r	0000166 // r	---	---	This probe s	S000002428(11),GENSCAN00000007694(11),YDR021W(11)
1771710_at	1021.6	P	2118.06	P	0.4823	P	YDR083W	S. cerevisiae	YDR083W S	chr4:612066	Nucleolar pr	RRP8	chr4	YDR083W	---	P38961	---	---	---	0006364 // r	0000781 // c	0008168 // r	---	---	This probe s	S000002490(11),GENSCAN00000007787(11),YDR083W(11)
1776014_at	670.71	P	2106.02	P	0.3185	P	YDR087C	S. cerevisiae	YDR087C S	chr4:617462	Essential ev	RRP1	chr4	YDR087C	---	P35178	---	---	---	0006364 // r	0005634 // r	---	---	---	This probe s	S000002494(11),GENEFINDER00000002925(11),GENSCAN00000007885(11),YDR087C(11)
1778038_at	1833.23	P	3760.76	P	0.4875	P	YDR091C	S. cerevisiae	YDR091C.S	chr4:626700	Essential iro	RLI1	chr4	YDR091C	---	Q03195	---	---	---	0000054 // r	0005634 // r	0000166 // r	---	---	This probe s	S000002498(11),GENEFINDER00000002764(11),GENSCAN00000008092(11),YDR091C(11)
1773459_at	1131.87	P	3177.72	P	0.3562	P	YDR101C	S. cerevisiae	YDR101C.S	chr4:655678	Shuttling pre	ARX1	chr4	YDR101C	---	Q03862	---	---	---	0009987 // c	0005634 // r	0005515 // r	IPR000994	---	This probe s	S000002508(11),GENEFINDER00000002911(11),GENSCAN00000007946(11),YDR101C(11)
1775856_at	1228.6	P	2540.15	P	0.4837	P	YDR120C	S. cerevisiae	YDR120C S	chr4:691541	tRNA methy	TRM1	chr4	YDR120C	---	P15565 /// C	---	---	---	0008033 // t	0005634 // r	0003723 // F	---	---	This probe s	S000002527(11),GENEFINDER00000002692(11),GENSCAN00000008192(11),YDR120C(11)
1769406_at	780.09	P	1665.51	P	0.4684	P	YDR144C	S. cerevisiae	YDR144C S	chr4:744303	GPI-anchore	MKC7	chr4	YDR144C	---	P53379	---	---	---	0006508 // r	0005886 // r	0004190 // a	---	---	This probe s	S000002551(11),GENEFINDER00000002663(11),GENSCAN00000007809(11),YDR144C(11)
1770349_at	469.59	P	1199.12	P	0.3916	P	YDR146C	S. cerevisiae	YDR146C.S	chr4:748605	Transcriptio	SWI5	chr4	YDR146C	---	P08153 /// C	---	---	---	0000114 // r	0005622 // i	0003676 // r	IPR000637	---	This probe s	S000002553(11),GENEFINDER00000002630(11),GENSCAN00000007958(11),YDR146C(11)
1775506_at	927.32	P	2054.9	P	0.4513	P	YDR161W	S. cerevisiae	YDR161W S	chr4:779035	---	---	chr4	YDR161W	---	Q03771	---	---	---	---	0005634 // r	0005488 // b	---	---	This probe s	S000002568(11),GENEFINDER00000002939(11),GENSCAN00000007948(11),YDR161W(11)
1777710_at	23.08	P	83.93	P	0.275	P	YDR179C	S. cerevisiae	YDR179C.S	chr4:818700	Subunit of th	CSN9	chr4	YDR179C	---	Q03981	---	---	---	0000338 // r	0005634 // r	0005515 // r	---	---	This probe s	S000002586(11),GENSCAN00000007766(11),YDR179C(11)
1775866_at	356.39	P	1892.09	P	0.1884	P	YDR299W	S. cerevisiae	YDR299W.S	chr4:105961	Essential pro	BFR2	chr4	YDR299W	---	Q06631	---	---	---	0006364 // r	0005634 // r	0005515 // r	---	---	This probe s	S000002707(11),GENEFINDER00000002804(11),GENSCAN00000007835(11),YDR299W(11)
1774303_at	561.71	P	1192.11	P	0.4712	P	YDR303C	S. cerevisiae	YDR303C.S	chr4:106871	Component	RSC3	chr4	YDR303C	---	Q06639	---	---	---	0000082 // (	0005634 // r	0003677 // I	---	---	This probe s	S000002711(11),GENEFINDER00000002792(11),GENSCAN00000008223(11),YDR303C(11)
1774142_at	1645.59	P	3918.2	P	0.42	P	YDR309C	S. cerevisiae	YDR309C.S	chr4:107903	Redundant r	GIC2	chr4	YDR309C	---	Q06648	---	---	---	0007096 // r	0000131 // i	0005100 // F	---	---	This probe s	S000002717(11),GENSCAN00000007680(11),YDR309C(11)
1777100_at	185.56	P	528.56	P	0.3511	P	YDR312W	S. cerevisiae	YDR312W.S	chr4:108757	Protein regu	SSF2	chr4	YDR312W	---	Q12153	---	---	---	0000027 // r	0005634 // r	0019843 // r	---	---	This probe s	S000002720(11),GENEFINDER00000002775(11),GENSCAN00000008234(11),YDR312W(11)
1778980_at	492.9	P	1497.4	P	0.3292	P	YDR324C	S. cerevisiae	YDR324C.S	chr4:111442	Subunit of U	UTP4	chr4	YDR324C	---	Q06679	---	---	---	0000462 // r	0005634 // r	0005515 // r	IPR008386	---	This probe s	S000002732(11),GENEFINDER00000002784(10),GENSCAN00000007950(11),YDR324C(11)
1770928_at	194.42	P	519.87	P	0.374	P	YDR325W	S. cerevisiae	YDR325W.S	chr4:111711	Subunit of th	YCG1	chr4	YDR325W	---	Q06680	---	---	---	0000070 // r	0000799 // r	0005488 // b	---	---	This probe s	S000002733(11),GENEFINDER00000002713(11),GENSCAN00000008267(11),YDR325W(11)
1772967_at	165.67	P	367.75	P	0.4505	P	YDR356W	S. cerevisiae	YDR356W.S	chr4:118609	Inner plaque	SPC110	chr4	YDR356W	---	P32380	---	---	---	0000742 // k	0005634 // r	0005200 // s	---	---	This probe s	S000002764(11),GENSCAN00000008142(11),YDR356W(11)
1773397_at	1060.47	P	2383.65	P	0.4449	P	YDR398W	S. cerevisiae	YDR398W.S	chr4:126745	Subunit of U	UTP5	chr4	YDR398W	---	Q04177	---	---	---	0000462 // r	0005634 // r	0005515 // r	---	---	This probe s	S000002806(11),GENSCAN00000007773(11),YDR398W(11)
1772547_at	316.49	P	910.31	P	0.3477	P	YDR412W	S. cerevisiae	YDR412W.S	chr4:129468	Component	RRP17	chr4	YDR412W	---	Q04031	---	---	---	0006364 // r	0005634 // r	---	---	---	This probe s	S000002820(11),GENEFINDER00000002870(11),GENSCAN00000008154(11),YDR412W(11)
1778685_at	537.48	P	1713.17	P	0.3137	P	YDR449C	S. cerevisiae	YDR449C S	chr4:135756	Nucleolar pr	UTP6	chr4	YDR449C	---	Q02354	---	---	---	0000447 // e	0005622 // i	0030515 // s	---	---	This probe s	S000002857(11),GENEFINDER00000002933(10),GENSCAN00000007704(11),YDR449C(11)
1778368_at	351.17	P	978.48	P	0.3589	P	YDR451C	S. cerevisiae	YDR451C.S	chr4:136110	One of two I	YHP1	chr4	YDR451C	---	Q04116	---	---	---	0000122 // r	0000228 // r	0003677 // I	---	---	This probe s	S000002859(11),GENSCAN00000007687(11),YDR451C(11)
1778249_at	1377.55	P	3047.7	P	0.452	P	YDR492W	S. cerevisiae	YDR492W.S	chr4:143491	Membrane r	IZH1	chr4	YDR492W	---	Q03419	---	---	---	0006629 // I	0005783 // e	0005515 // r	---	S000002900	This probe s	S000002900(11),YDR492W(11)
1771126_at	1909.5	P	4015.99	P	0.4755	P	YDR496C	S. cerevisiae	YDR496C.S	chr4:144142	Pumilio-hom	PUF6	chr4	YDR496C	---	Q04373	---	---	---	0006417 // r	0005634 // r	0003723 // F	---	---	This probe s	S000002904(11),GENSCAN00000008163(11),YDR496C(11)
1775116_at	566.01	P	1423.95	P	0.3975	P	YDR507C	S. cerevisiae	YDR507C.S	chr4:146234	Protein kina	GIN4	chr4	YDR507C	---	Q12263	---	---	---	0000135 // s	0005737 // c	0000166 // r	IPR001245	---	This probe s	S000002915(11),GENEFINDER00000002914(11),GENSCAN00000007886(11),YDR507C(11)
1773520_at	430.81	P	900.55	P	0.4784	P	YDR514C	S. cerevisiae	YDR514C.S	chr4:147153	---	---	chr4	YDR514C	---	Q04408	---	---	---	---	0005634 // r	0003676 // r	---	---	This probe s	S000002922(11),GENSCAN00000008038(11),YDR514C(11)
1771495_at	227.35	P	655.44	P	0.3469	P	YDR528W	S. cerevisiae	YDR528W.S	chr4:149457	Protein invol	HLR1	chr4	YDR528W	---	Q04429	---	---	---	0007047 // c	0005737 // c	---	---	---	This probe s	S000002936(11),GENSCAN00000007681(11),YDR528W(11)
1770725_at	187.25	P	466.66	P	0.4013	P	YEL061C	S. cerevisiae	YEL061C.S	chr5:36534-	Kinesin moto	CIN8	chr5	YEL061C	---	P27895	---	---	---	0000070 // r	0000235 // a	0000166 // r	---	---	This probe s	S000000787(11),GENSCAN00000004160(11),YEL061C(11)
1778542_at	138.26	P	359.99	P	0.3841	P	YEL048C	S. cerevisiae	YEL048C.S	chr5:64708-	---	---	chr5	YEL048C	---	P32613 /// C	---	---	---	0006810 // t	0005622 // i	0005515 // r	IPR006722	---	This probe s	S000000774(11),YEL048C(11)
1779506_at	1569.01	P	4503.75	P	0.3484	P	YEL040W	S. cerevisiae	YEL040W.S	chr5:78052-	Cell wall pro	UTR2	chr5	YEL040W	---	P32623	---	---	---	0005975 // c	0000144 // c	0004553 // h	---	---	This probe s	S000000766(11),S66130(10),GENEFINDER00000000298(11),GENSCAN00000004024(11),YEL040W(11)
1774083_at	18.34	A	56.57	P	0.3242	P	YEL019C	S. cerevisiae	YEL019C.S	chr5:120497	SUMO ligas	MMS21	chr5	YEL019C	---	A6N4D5 /// I	---	---	---	0006281 // [	0005634 // r	0008270 // z	---	---	This probe s	S000000745(11),GENSCAN00000004090(11),YEL019C(11)
1774362_at	612.4	P	1470.26	P	0.4165	P	YER002W	S. cerevisiae	YER002W.S	chr5:156801	Constituent	NOP16	chr5	YER002W	---	P40007	---	---	---	0006364 // r	0005634 // r	0005515 // r	---	---	This probe s	S000000804(11),GENSCAN00000004104(11),GENEFINDER00000000300(9),YER002W(11)
1772331_at	410.84	P	822.39	P	0.4996	P	YER016W	S. cerevisiae	YER016W S	chr5:188275	Microtubule-	BIM1	chr5	YER016W	---	P40013	---	---	---	0007019 // r	0000776 // k	0005200 // s	IPR000873	---	This probe s	S000000818(11),GENEFINDER00000000244(11),GENSCAN00000004138(11),YER016W(11)
1777041_at	576.94	P	1460.7	P	0.395	P	YER032W	S. cerevisiae	YER032W.S	chr5:215061	Protein invol	FIR1	chr5	YER032W	---	P40020	---	---	---	0006378 // r	0005935 // c	---	---	---	This probe s	S000000834(11),GENSCAN00000004122(11),YER032W(11)
1770990_at	1052.18	P	2145.21	P	0.4905	P	YER064C	S. cerevisiae	YER064C.S	chr5:282702	---	---	chr5	YER064C	---	P40041	---	---	---	0045449 // r	0005634 // r	---	---	---	This probe s	S000000866(11),GENSCAN00000004082(11),YER064C(11)
1776623_at	350.68	P	701.53	P	0.4999	P	YER075C	S. cerevisiae	YER075C.S	chr5:308408	Phosphotyro	PTP3	chr5	YER075C	---	P40048	---	---	---	0000173 // i	0005634 // r	0004721 // r	---	---	This probe s	S000000877(11),GENSCAN00000004049(11),YER075C(11)
1777602_at	591.11	P	1374.69	P	0.43	P	YER082C	S. cerevisiae	YER082C.S	chr5:324267	Nucleolar pr	UTP7	chr5	YER082C	---	P40055	---	---	---	0000447 // e	0005634 // r	0005515 // r	---	---	This probe s	S000000884(11),GENEFINDER00000000282(11),GENSCAN00000004196(11),YER082C(11)
1779544_at	276.27	P	980.35	P	0.2818	P	YER127W	S. cerevisiae	YER127W.S	chr5:414476	Essential pro	LCP5	chr5	YER127W	---	P40079	---	---	---	0000154 // r	0005634 // r	0003723 // F	---	---	This probe s	S000000929(11),GENEFINDER00000000301(11),GENSCAN00000004233(11),YER127W(11)
1777853_at	1062.18	P	3281.16	P	0.3237	P	YER145C	S. cerevisiae	YER145C.S	chr5:460520	High affinity	FTR1	chr5	YER145C	---	P40088	---	---	---	0006810 // t	0005886 // r	0005381 // i	---	S000000947	This probe s	S000000947(11),GENEFINDER00000000245(11),GENSCAN00000004003(11),YER145C(11)
1772203_at	433.98	P	1116.32	P	0.3888	P	YFL023W	S. cerevisiae	YFL023W.S	chr6:90983-	Protein invol	BUD27	chr6	YFL023W	---	P43573	---	---	---	0000282 // c	0005737 // c	0005515 // r	---	---	This probe s	S000001871(11),GENEFINDER00000000222(11),GENSCAN00000003563(11),YFL023W(11)
1779003_at	404.27	P	1216.29	P	0.3324	P	YFR028C	S. cerevisiae	YFR028C.S	chr6:208400	Protein phos	CDC14	chr6	YFR028C	---	Q00684	---	---	---	0000278 // r	0005634 // r	0004721 // r	IPR000340	---	This probe s	S000001924(11),GENEFINDER00000000193(11),GENSCAN00000003484(11),YFR028C(11)
1778936_at	913.8	P	1917.2	P	0.4766	P	YGL244W	S. cerevisiae	YGL244W.S	chr7:41497-	Subunit of th	RTF1	chr7	YGL244W	---	P53064	---	---	---	0006350 // t	0005634 // r	0003677 // I	---	---	This probe s	S000003213(11),GENEFINDER00000002317(11),GENSCAN00000006211(11),YGL244W(11)
1776006_at	17.01	P	71.09	P	0.2393	P	YGL188C-A	S. cerevisiae	YGL188C-A	chr7:148827	---	---	chr7	YGL188C-A	---	Q8TGU1	---	---	---	---	---	---	---	---	This probe s	S000028635(11),YGL188C-A(11)
1771440_at	644.76	P	1558.11	P	0.4138	P	YGL171W	S. cerevisiae	YGL171W.S	chr7:182393	ATP-depend	ROK1	chr7	YGL171W	---	P45818	---	---	---	0006364 // r	0005634 // r	0000166 // r	---	---	This probe s	S000003139(11),GENEFINDER00000002447(10),GENSCAN00000006510(11),YGL171W(11)
1778649_at	63.65	P	254.27	P	0.2503	P	YGL170C	S. cerevisiae	YGL170C.S	chr7:184156	Component	SPO74	chr7	YGL170C	---	P45819 /// C	---	---	---	0030435 // s	0005634 // r	0005198 // s	---	---	This probe s	S000003138(11),GENSCAN00000005669(11),YGL170C(11)
1770680_at	507.74	P	1123.22	P	0.452	P	YGL169W	S. cerevisiae	YGL169W.S	chr7:186062	Single-stran	SUA5	chr7	YGL169W	---	P32579	---	---	---	0009060 // a	0005737 // c	---	---	---	This probe s	S000003137(11),GENSCAN00000005868(11),YGL169W(11)
1775352_at	1635.73	P	3300.26	P	0.4956	P	YGL120C	S. cerevisiae	YGL120C.S	chr7:281637	RNA helicas	PRP43	chr7	YGL120C	---	P53131	---	---	---	0000390 // s	0005634 // r	0000166 // r	---	---	This probe s	S000003088(11),GENEFINDER00000002410(11),GENSCAN00000006347(11),YGL120C(11)
1775262_at	251.3	P	551.48	P	0.4557	P	YGL116W	S. cerevisiae	YGL116W.S	chr7:289809	Cell-cycle re	CDC20	chr7	YGL116W	---	P26309	---	---	---	0000022 // r	0005634 // r	0004842 // u	IPR000644	GENEFINDE	This probe s	S000003084(11),GENEFINDER00000002341(11),GENSCAN00000005938(11),YGL116W(11)
1771159_at	960.92	P	2064.04	P	0.4656	P	YGL111W	S. cerevisiae	YGL111W.S	chr7:299978	Constituent	NSA1	chr7	YGL111W	---	P53136	---	---	---	0006364 // r	0005634 // r	---	---	---	This probe s	S000003079(11),GENSCAN00000006058(11),YGL111W(11)
1772505_at	395.66	P	954.48	P	0.4145	P	YGL101W	S. cerevisiae	YGL101W.S	chr7:312193	---	---	chr7	YGL101W	---	P53144	---	---	---	---	0005634 // r	0003824 // c	---	---	This probe s	S000003069(11),GENSCAN00000006023(11),YGL101W(11)
1778190_at	1093.67	P	2320.31	P	0.4713	P	YGL099W	S. cerevisiae	YGL099W.S	chr7:314631	Putative GTI	LSG1	chr7	YGL099W	---	P53145	---	---	---	0000054 // r	0005622 // i	0000166 // r	---	---	This probe s	S000003067(11),GENEFINDER00000002535(11),GENSCAN00000006506(11),YGL099W(11)
1774231_at	278.35	P	603.42	P	0.4613	P	YGL061C	S. cerevisiae	YGL061C.S	chr7:388968	Essential su	DUO1	chr7	YGL061C	---	P53168	---	---	---	0007049 // c	0000777 // c	0005200 // s	---	---	This probe s	S000003029(11),YGL061C(11)
1776992_at	1639.25	P	5710.58	P	0.2871	P	YGL032C	S. cerevisiae	YGL032C.S	chr7:436572	Adhesion su	AGA2	chr7	YGL032C	---	P32781	---	NP_011483	---	0000752 // a	0009277 // f	0005515 // r	---	---	This probe s	X62877(11)
1774719_at	232.02	P	698.62	P	0.3321	P	YGL029W	S. cerevisiae	YGL029W.S	chr7:440065	Protein invol	CGR1	chr7	YGL029W	---	P53188	---	---	---	0006364 // r	0005634 // r	0005515 // r	---	---	This probe s	S000002997(11),GENSCAN00000005712(11),YGL029W(11)
1776407_at	558.91	P	1355.81	P	0.4122	P	YGL021W	S. cerevisiae	YGL021W.S	chr7:454787	Protein kina	ALK1	chr7	YGL021W	---	P43633	---	---	---	0006468 // r	---	0000166 // r	---	---	This probe s	S000002989(11),GENSCAN00000006266(11),YGL021W(11)
1778806_at	1023.2	P	3227.7	P	0.317	P	YGR014W	S. cerevisiae	YGR014W.S	chr7:516943	Mucin family	MSB2	chr7	YGR014W	---	P32334	---	---	---	0001402 // s	0005887 // i	0005034 // c	---	S000003246	This probe s	S000003246(11),GENEFINDER00000002305(11),GENSCAN00000006549(11),YGR014W(11)
1770624_at	872.51	P	1911.77	P	0.4564	P	YGR078C	S. cerevisiae	YGR078C.S	chr7:639772	Part of the h	PAC10	chr7	YGR078C	---	P48363	---	---	---	0006457 // r	0005737 // c	0005515 // r	---	---	This probe s	S000003310(11),GENEFINDER00000002486(11),GENSCAN00000005979(11),YGR078C(11)
1774239_at	515.43	P	1725.27	P	0.2988	P	YGR079W	S. cerevisiae	YGR079W.S	chr7:640720	---	---	chr7	YGR079W	---	P53249	---	---	---	---	---	---	---	S000003311	This probe s	S000003311(11),GENEFINDER00000002458(10),GENSCAN00000005774(11),YGR079W(11)
1770106_at	443.96	P	1211.98	P	0.3663	P	YGR081C	S. cerevisiae	YGR081C.S	chr7:643148	Protein regu	SLX9	chr7	YGR081C	---	P53251	---	---	---	0000462 // r	0005634 // r	0005515 // r	---	---	This probe s	S000003313(11),GENSCAN00000006070(11),YGR081C(11)
1769973_at	970.71	P	3238.46	P	0.2997	P	YGR108W	S. cerevisiae	YGR108W.S	chr7:703636	B-type cyclin	CLB1	chr7	YGR108W	---	P24868	---	---	---	0000079 // r	0000307 // c	0005515 // r	---	---	This probe s	S000003340(11),GENEFINDER00000002311(11),GENSCAN00000006017(11),YGR108W(11)
1775052_at	680.54	P	1810.1	P	0.376	P	YGR128C	S. cerevisiae	YGR128C.S	chr7:747951	Nucleolar pr	UTP8	chr7	YGR128C	---	P53276	---	---	---	0000462 // r	0005634 // r	0005515 // r	---	---	This probe s	S000003360(11),GENSCAN00000005912(11),YGR128C(11)
1773994_at	964.91	P	2365.29	P	0.4079	P	YGR145W	S. cerevisiae	YGR145W.S	chr7:781768	Essential nu	ENP2	chr7	YGR145W	---	P48234	---	---	---	0006364 // r	0005634 // r	---	---	---	This probe s	S000003377(11),GENSCAN00000006062(11),GENEFINDER00000002429(11),YGR145W(11)
1780244_at	803.98	P	1719.25	P	0.4676	P	YGR173W	S. cerevisiae	YGR173W.S	chr7:843855	Protein with	RBG2	chr7	YGR173W	---	P53295	---	---	---	---	0005622 // i	0000166 // r	---	---	This probe s	S000003405(11),GENSCAN00000005752(11),YGR173W(11)
1771190_at	229.46	P	614.12	P	0.3736	P	YGR177C	S. cerevisiae	YGR177C.S	chr7:848830	Alcohol acet	ATF2	chr7	YGR177C	---	P53296 /// C	---	---	---	0006066 // c	0005737 // c	0004026 // a	---	---	This probe s	S000003409(11),GENSCAN00000005568(11),YGR177C(11)
1774982_at	810.89	P	1980.88	P	0.4094	P	YGR187C	S. cerevisiae	YGR187C.S	chr7:870233	Nonessentia	HGH1	chr7	YGR187C	---	P48362	---	---	---	0042254 // r	0005737 // c	0005488 // b	---	---	This probe s	S000003419(11),GENEFINDER00000002302(11),GENSCAN00000006057(11),YGR187C(11)
1778092_at	951.98	P	1954.9	P	0.487	P	YGR200C	S. cerevisiae	YGR200C.S	chr7:899905	Subunit of E	ELP2	chr7	YGR200C	---	P42935 /// C	---	---	---	0006350 // t	0005634 // r	0005515 // r	---	---	This probe s	S000003432(11),GENEFINDER00000002399(11),GENSCAN00000005627(11),YGR200C(11)
1771458_at	885.88	P	2299.96	P	0.3852	P	YGR208W	S. cerevisiae	YGR208W S	chr7:911884	Phosphoseri	SER2	chr7	YGR208W	---	P42941	---	---	---	0006564 // L	0005634 // r	0003824 // c	IPR006384	---	This probe s	S000003440(11),GENEFINDER00000002323(11),GENSCAN00000006036(11),YGR208W(11)
1779152_at	1193.12	P	2643.83	P	0.4513	P	YGR245C	S. cerevisiae	YGR245C.S	chr7:979767	Highly conse	SDA1	chr7	YGR245C	---	P53313	---	---	---	0000055 // r	0005634 // r	---	---	---	This probe s	S000003477(11),GENEFINDER00000002533(11),GENSCAN00000005808(11),YGR245C(11)
1775393_at	224.21	P	850.91	P	0.2635	P	YGR271C-A	S. cerevisiae	YGR271C-A	chr7:103780	Essential pro	EFG1	chr7	YGR271C-A	---	Q3E705	---	---	---	0000080 // (	0005634 // r	---	---	---	This probe s	S000007608(11),GENEFINDER00000002321(11),YGR271C-A(11)
1770541_at	165.06	P	686.25	P	0.2405	P	YGR272C	S. cerevisiae	YGR272C S	chr7:103804	---	---	---	YGR272C	---	---	---	---	---	---	---	---	---	---	This probe s	S000003504(11),YGR272C(11)
1773125_at	612.58	P	1383.33	P	0.4428	P	YGR283C	S. cerevisiae	YGR283C.S	chr7:105901	---	---	chr7	YGR283C	---	P53336	---	---	---	0042254 // r	0005730 // r	---	---	---	This probe s	S000003515(11),GENSCAN00000005872(11),YGR283C(11)
1778188_at	378.17	P	891.32	P	0.4243	P	YHL013C	S. cerevisiae	YHL013C.S	chr8:77425-	Protein of ur	OTU2	chr8	YHL013C	---	P38747	---	---	---	---	0005737 // c	---	---	---	This probe s	S000001005(11),GENEFINDER00000000619(10),GENSCAN00000003862(11),YHL013C(11)
1779279_at	290.38	P	669.66	P	0.4336	P	YHR023W	S. cerevisiae	YHR023W.S	chr8:151656	Type II myos	MYO1	chr8	YHR023W	---	P08964	---	---	---	0000910 // c	0000131 // i	0000166 // r	---	---	This probe s	S000001065(11),GENEFINDER00000000546(11),GENSCAN00000003908(11),YHR023W(11)
1778145_at	155.37	P	366.83	P	0.4235	P	YHR031C	S. cerevisiae	YHR031C.S	chr8:170789	DNA helicas	RRM3	chr8	YHR031C	---	P38766	---	---	---	0000002 // r	0005657 // r	0000166 // r	---	---	This probe s	S000001073(11),GENEFINDER00000000592(11),GENSCAN00000003873(11),YHR031C(11)
1777020_at	1274.76	P	3339.35	P	0.3817	P	YHR052W	S. cerevisiae	YHR052W.S	chr8:210839	Essential pro	CIC1	chr8	YHR052W	---	P38779	---	---	---	0006396 // F	0000502 // r	0003723 // F	---	---	This probe s	S000001094(11),GENEFINDER00000000565(11),GENSCAN00000003779(11),YHR052W(11)
1776125_at	361.3	P	842.29	P	0.4289	P	YHR061C	S. cerevisiae	YHR061C.S	chr8:221534	Protein of ur	GIC1	chr8	YHR061C	---	P38785	---	---	---	0007096 // r	0000131 // i	0005100 // F	---	---	This probe s	S000001103(11),GENEFINDER00000000584(11),GENSCAN00000003933(11),YHR061C(11)
1771367_at	565.02	P	1238.55	P	0.4562	P	YHR062C	S. cerevisiae	YHR062C S	chr8:222877	Subunit of b	RPP1	chr8	YHR062C	---	P38786	---	---	---	0006364 // r	0000172 // r	0000171 // r	---	---	This probe s	S000001104(11),GENSCAN00000003798(11),YHR062C(11)
1770047_at	751.46	P	2030.77	P	0.37	P	YHR065C	S. cerevisiae	YHR065C.S	chr8:227532	Protein invol	RRP3	chr8	YHR065C	---	P38712	---	---	---	0006364 // r	0005634 // r	0000166 // r	---	---	This probe s	S000001107(11),GENEFINDER00000000518(11),GENSCAN00000003835(11),YHR065C(11)
1774186_at	255.53	P	1233.75	P	0.2071	P	YHR066W	S. cerevisiae	YHR066W.S	chr8:229335	Constituent	SSF1	chr8	YHR066W	---	P38789	---	---	---	0000027 // r	0005634 // r	0005515 // r	---	---	This probe s	S000001108(11),GENEFINDER00000000594(11),GENSCAN00000003971(11),YHR066W(11)
1770963_at	664.52	P	1376.65	P	0.4827	P	YHR070W	S. cerevisiae	YHR070W.S	chr8:234881	tRNA(m(1)G	TRM5	chr8	YHR070W	---	P38793	---	---	---	0008033 // t	0005634 // r	0008168 // r	---	---	This probe s	S000001112(11),GENEFINDER00000000556(10),GENSCAN00000003767(11),YHR070W(11)
1776885_at	799.69	P	1763.41	P	0.4535	P	YHR084W	S. cerevisiae	YHR084W.S	chr8:274174	Transcriptio	STE12	chr8	YHR084W	---	P13574	---	---	---	0000747 // c	0005634 // r	0003677 // I	---	---	This probe s	S000001126(11),GENSCAN00000003903(11),GENEFINDER00000000519(11),YHR084W(11)
1771359_at	441.53	P	1223.04	P	0.361	P	YHR085W	S. cerevisiae	YHR085W.S	chr8:276764	Essential co	IPI1	chr8	YHR085W	---	P38803	---	---	---	0000027 // r	0005634 // r	---	---	---	This probe s	S000001127(11),GENEFINDER00000000525(10),GENSCAN00000003836(11),YHR085W(11)
1777405_at	619.93	P	1544.63	P	0.4013	P	YHR088W	S. cerevisiae	YHR088W.S	chr8:281495	Nucleolar pr	RPF1	chr8	YHR088W	---	P38805	---	---	---	0000027 // r	0005634 // r	0003723 // F	---	---	This probe s	S000001130(11),GENSCAN00000003846(11),YHR088W(11)
1775179_at	367.56	P	818.1	P	0.4493	P	YHR127W	S. cerevisiae	YHR127W.S	chr8:360914	---	---	chr8	YHR127W	---	P38833	---	---	---	0000022 // r	0005634 // r	---	---	---	This probe s	S000001169(11),GENSCAN00000003849(11),YHR127W(11)
1778791_at	443.94	P	930.41	P	0.4771	P	YHR144C	S. cerevisiae	YHR144C.S	chr8:387788	Deoxycytidir	DCD1	chr8	YHR144C	---	P06773	---	---	---	0006221 // r	0005737 // c	0003824 // c	---	---	This probe s	S000001187(11),GENSCAN00000004007(11),YHR144C(11)
1777403_at	841.55	P	2016.94	P	0.4172	P	YHR148W	S. cerevisiae	YHR148W.S	chr8:393534	Component	IMP3	chr8	YHR148W	---	P32899	---	---	---	0006364 // r	0005622 // i	0003723 // F	---	---	This probe s	S000001191(11),GENSCAN00000003900(11),YHR148W(11)
1770790_at	587.96	P	1241.82	P	0.4735	P	YHR149C	S. cerevisiae	YHR149C.S	chr8:394455	Integral mer	SKG6	chr8	YHR149C	---	P32900	---	---	---	---	0000131 // i	---	---	S000001192	This probe s	S000001192(11),GENEFINDER00000000614(11),GENSCAN00000003773(11),YHR149C(11)
1772981_at	447.11	P	979.68	P	0.4564	P	YHR154W	S. cerevisiae	YHR154W.S	chr8:402966	Protein impli	RTT107	chr8	YHR154W	---	P38850	---	---	---	0006260 // [	0005622 // i	0005515 // r	---	---	This probe s	S000001197(11),GENEFINDER00000000611(10),GENSCAN00000003872(11),YHR154W(11)
1770147_at	350.18	P	1119.11	P	0.3129	P	YHR169W	S. cerevisiae	YHR169W.S	chr8:442179	ATPase, put	DBP8	chr8	YHR169W	---	P38719	---	---	---	0000447 // e	0005634 // r	0000166 // r	---	---	This probe s	S000001212(11),GENEFINDER00000000540(11),GENSCAN00000003764(11),YHR169W(11)
1778275_at	786.65	P	2019.35	P	0.3896	P	YHR196W	S. cerevisiae	YHR196W.S	chr8:491926	Nucleolar pr	UTP9	chr8	YHR196W	---	P38882	---	---	---	0000462 // r	0005634 // r	0005515 // r	---	---	This probe s	S000001239(11),GENSCAN00000003952(11),YHR196W(11)
1769550_at	504.63	P	1557.53	P	0.324	P	YHR197W	S. cerevisiae	YHR197W.S	chr8:493891	Essential co	RIX1	chr8	YHR197W	---	P38883	---	---	---	0000027 // r	0005634 // r	0005515 // r	---	---	This probe s	S000001240(11),GENEFINDER00000000660(11),GENSCAN00000003819(11),YHR197W(11)
1776290_at	475.02	P	1244.11	P	0.3818	P	YIL158W	S. cerevisiae	YIL158W.S1	chr9:46200-	Putative pro	AIM20	chr9	YIL158W	---	P40451	---	---	---	---	0000324 // f	---	---	S000001420	This probe s	S000001420(11),GENEFINDER00000000393(11),GENSCAN00000003331(11),YIL158W(11)
1771084_at	55.13	P	123.96	P	0.4447	P	YIL150C	S. cerevisiae	YIL150C.S1	chr9:61012-	Essential ch	MCM10	chr9	YIL150C	---	P32354	---	---	---	0000084 // S	0005634 // r	0003682 // c	---	---	This probe s	S000001412(11),GENSCAN00000003415(11),YIL150C(11)
1777130_at	154.91	P	516.04	P	0.3002	P	YIL131C	S. cerevisiae	YIL131C.S1	chr9:100780	Forkhead fa	FKH1	chr9	YIL131C	---	P40466	---	---	---	0000117 // r	0005634 // r	0003677 // I	---	---	This probe s	S000001393(11),GENSCAN00000003580(11),YIL131C(11)
1773941_at	534.79	P	1285.97	P	0.4159	P	YIL127C	S. cerevisiae	YIL127C.S1	chr9:117023	Putative pro	RRT14	chr9	YIL127C	---	P40470 /// C	---	---	---	---	0005730 // r	0005515 // r	---	---	This probe s	S000001389(11),GENSCAN00000003332(11),YIL127C(11)
1772209_at	239.77	P	596.61	P	0.4019	P	YIL104C	S. cerevisiae	YIL104C.S1	chr9:169978	Protein regu	SHQ1	chr9	YIL104C	---	P40486	---	---	---	0000493 // b	0005634 // r	0005515 // r	IPR007052	---	This probe s	S000001366(11),GENEFINDER00000000483(11),GENSCAN00000003521(11),YIL104C(11)
1775775_at	709.81	P	1476.93	P	0.4806	P	YIL103W	S. cerevisiae	YIL103W.S1	chr9:171747	Protein regu	DPH1	chr9	YIL103W	---	P40487	---	---	---	0017183 // r	0005737 // c	0005515 // r	---	---	This probe s	S000001365(11),GENEFINDER00000000489(11),GENSCAN00000003353(11),YIL103W(11)
1770967_at	387.97	P	977.02	P	0.3971	P	YIL096C	S. cerevisiae	YIL096C.S1	chr9:182113	---	---	chr9	YIL096C	---	P40493	---	---	---	---	0005634 // r	---	---	---	This probe s	S000001358(11),GENSCAN00000003547(11),YIL096C(11)
1771835_at	677.74	P	1903.87	P	0.356	P	YIL091C	S. cerevisiae	YIL091C.S1	chr9:191026	---	---	chr9	YIL091C	---	P40498	---	---	---	---	0005634 // r	---	---	---	This probe s	S000001353(11),GENEFINDER00000000429(11),GENSCAN00000003579(11),YIL091C(11)
1773261_at	468.01	P	1132.82	P	0.4131	P	YIL079C	S. cerevisiae	YIL079C.S1	chr9:210919	RING finger	AIR1	chr9	YIL079C	---	P40507	---	---	---	0000292 // F	0005634 // r	0003676 // r	---	---	This probe s	S000001341(11),GENSCAN00000003395(11),YIL079C(11)
1774769_at	49.96	P	221	P	0.2261	P	YIL037C	S. cerevisiae	YIL037C.S1	chr9:283027	Pheromone-	PRM2	chr9	YIL037C	---	P40534	---	---	---	0000747 // c	0016020 // r	---	---	S000001299	This probe s	S000001299(11),GENEFINDER00000000448(11),GENSCAN00000003556(11),YIL037C(11)
1770008_at	281.26	P	780.83	P	0.3602	P	YIL019W	S. cerevisiae	YIL019W.S1	chr9:315090	Protein regu	FAF1	chr9	YIL019W	---	P40546	---	---	---	0000462 // r	0005634 // r	---	---	---	This probe s	S000001281(11),GENEFINDER00000000444(10),GENSCAN00000003390(11),YIL019W(11)
1778408_at	1535.52	P	4856.13	P	0.3162	P	YIL015W	S. cerevisiae	YIL015W.S1	chr9:322339	Aspartyl pro	BAR1	chr9	YIL015W	---	P12630	---	---	---	0000754 // a	0005576 // e	0004190 // a	---	S000001277	This probe s	S000001277(11),GENEFINDER00000000415(11),GENSCAN00000003625(11),YIL015W(11)
1772032_at	527.47	P	1067.86	P	0.494	P	YIR010W	S. cerevisiae	YIR010W.S	chr9:375427	Essential co	DSN1	chr9	YIR010W	---	P40568	---	---	---	0007049 // c	0000776 // k	0005515 // r	---	---	This probe s	S000001449(11),GENEFINDER00000000413(11),GENSCAN00000003364(11),YIR010W(11)
1771743_at	814.76	P	1721.95	P	0.4732	P	YJL208C	S. cerevisiae	YJL208C.S1	chr10:40193	Major mitocl	NUC1	chr10	YJL208C	---	P08466	---	---	---	0006308 // [	0005634 // r	0000287 // r	---	S000003744	This probe s	S000003744(11),GENSCAN00000004204(11),YJL208C(11)
1779227_at	244	P	521.28	P	0.4681	P	YJL194W	S. cerevisiae	YJL194W.S	chr10:69336	Essential AT	CDC6	chr10	YJL194W	---	P09119	---	---	---	0000082 // (	0005656 // r	0000166 // r	---	---	This probe s	S000003730(11),GENSCAN00000004419(11),YJL194W(11)
1777928_at	818.85	P	2833.43	P	0.289	P	YJL157C	S. cerevisiae	YJL157C.S1	chr10:12353	Cyclin-depen	FAR1	chr10	YJL157C	---	P21268	---	---	---	0000750 // r	0005634 // r	0004860 // r	---	---	This probe s	S000003693(11),GENEFINDER00000001217(11),GENSCAN00000004764(11),YJL157C(11)
1770485_at	1626.24	P	3646.23	P	0.446	P	YJL148W	S. cerevisiae	YJL148W.S	chr10:14013	RNA polyme	RPA34	chr10	YJL148W	---	P47006	---	---	---	0006350 // t	0005634 // r	0003899 // I	---	---	This probe s	S000003684(11),GENSCAN00000004746(11),YJL148W(11)
1770507_at	443.5	P	1135.14	P	0.3907	P	YJL125C	S. cerevisiae	YJL125C.S1	chr10:18522	Subunit of tF	GCD14	chr10	YJL125C	---	P46959	---	---	---	0008033 // t	0005634 // r	0005515 // r	---	---	This probe s	S000003661(11),GENEFINDER00000001250(11),GENSCAN00000004529(11),YJL125C(11)
1779514_at	722.25	P	2172.22	P	0.3325	P	YJL122W	S. cerevisiae	YJL122W.S	chr10:18941	Shuttling pre	ALB1	chr10	YJL122W	---	P47019	---	---	---	0006810 // t	0005634 // r	0005515 // r	---	---	This probe s	S000003658(11),YJL122W(11)
1778155_at	290.85	P	725.04	P	0.4012	P	YJL118W	S. cerevisiae	YJL118W.S	chr10:19133	---	---	chr10	YJL118W	---	P47022	---	---	---	---	0005840 // r	---	---	---	This probe s	S000003654(11),GENSCAN00000004575(11),YJL118W(11)
1775641_at	448.54	P	939.51	P	0.4774	P	YJL098W	S. cerevisiae	YJL098W.S	chr10:24177	Protein that	SAP185	chr10	YJL098W	---	P40856	---	---	---	0000082 // (	0005737 // c	0004722 // r	---	---	This probe s	S000003634(11),GENEFINDER00000001254(11),GENSCAN00000004156(11),YJL098W(11)
1779856_at	479.6	P	1066.07	P	0.4499	P	YJL074C	S. cerevisiae	YJL074C.S1	chr10:29885	Subunit of th	SMC3	chr10	YJL074C	---	P47037	---	---	---	0007049 // c	0000798 // r	0000166 // r	---	---	This probe s	S000003610(11),GENEFINDER00000001194(11),GENSCAN00000004424(11),YJL074C(11)
1779073_at	447.8	P	1556.82	P	0.2876	P	YJL069C	S. cerevisiae	YJL069C.S1	chr10:31061	Possible U3	UTP18	chr10	YJL069C	---	P40362	---	---	---	0000447 // e	0005634 // r	0005515 // r	---	---	This probe s	S000003605(11),GENEFINDER00000001194(11),GENSCAN00000004498(11),YJL069C(11)
1773231_at	429.39	P	1105.1	P	0.3886	P	YJL033W	S. cerevisiae	YJL033W.S	chr10:38353	Putative nuc	HCA4	chr10	YJL033W	---	P20448	---	---	---	0006364 // r	0005634 // r	0000166 // r	---	---	This probe s	S000003570(11),GENEFINDER00000001164(11),GENSCAN00000004450(11),YJL033W(11)
1769863_at	250.7	P	621.32	P	0.4035	P	YJL025W	S. cerevisiae	YJL025W.S	chr10:39366	Protein invol	RRN7	chr10	YJL025W	---	P40992	---	---	---	0006350 // t	0000120 // F	0003701 // F	---	---	This probe s	S000003562(11),GENSCAN00000004325(11),GENEFINDER00000001298(11),YJL025W(11)
1778892_at	309.76	P	783.61	P	0.3953	P	YJL011C	S. cerevisiae	YJL011C.S1	chr10:41397	RNA polyme	RPC17	chr10	YJL011C	---	P47076	---	---	---	0006350 // t	0005634 // r	0000166 // r	---	---	This probe s	S000003548(11),GENSCAN00000004490(11),YJL011C(11)
1777206_at	534.1	P	1242.5	P	0.4299	P	YJL010C	S. cerevisiae	YJL010C.S1	chr10:41725	Essential su	NOP9	chr10	YJL010C	---	P47077	---	---	---	0000056 // r	0005634 // r	0003723 // F	---	---	This probe s	S000003547(11),GENEFINDER00000001307(11),GENSCAN00000004590(11),YJL010C(11)
1772172_at	575.89	P	1188.5	P	0.4846	P	YJR002W	S. cerevisiae	YJR002W.S	chr10:43855	Component	MPP10	chr10	YJR002W	---	P47083	---	---	---	0000447 // e	0005634 // r	0005515 // r	---	GENEFINDE	This probe s	S000003762(11),GENEFINDER00000001169(11),GENSCAN00000004322(11),YJR002W(11)
1775023_at	390.48	P	914.84	P	0.4268	P	YJR003C	S. cerevisiae	YJR003C.S	chr10:44068	---	---	chr10	YJR003C	---	P47084	---	---	---	---	0005739 // r	---	---	---	This probe s	S000003763(11),GENSCAN00000004272(11),YJR003C(11)
1776092_at	422.6	P	845.81	P	0.4996	P	YJR031C	S. cerevisiae	YJR031C.S	chr10:48627	Guanine nuc	GEA1	chr10	YJR031C	---	P47102	---	---	---	0006888 // F	0000137 // (	0005085 // c	---	---	This probe s	S000003792(11),GENEFINDER00000001299(11),GENSCAN00000004455(11),YJR031C(11)
1776619_at	653.64	P	1436.02	P	0.4552	P	YJR041C	S. cerevisiae	YJR041C.S	chr10:50993	Nucleolar pr	URB2	chr10	YJR041C	---	P47108	---	---	---	0006364 // r	0005634 // r	---	---	---	This probe s	S000003802(11),GENSCAN00000004479(11),YJR041C(11)
1772491_at	27.41	P	55.63	P	0.4927	P	YJR053W	S. cerevisiae	YJR053W.S	chr10:53372	Component	BFA1	chr10	YJR053W	---	P47113	---	---	---	0007049 // c	0005816 // s	0005096 // (	---	---	This probe s	S000003814(11),GENSCAN00000004598(11),GENEFINDER00000001222(11),YJR053W(11)
1778487_at	1423.48	P	3194.09	P	0.4457	P	YJR063W	S. cerevisiae	YJR063W.S	chr10:55488	RNA polyme	RPA12	chr10	YJR063W	---	P32529	---	---	---	0006350 // t	0005634 // r	0003676 // r	---	---	This probe s	S000003824(11),GENSCAN00000004664(11),YJR063W(11)
1775342_at	1943.09	P	4049.49	P	0.4798	P	YJR070C	S. cerevisiae	YJR070C.S	chr10:56931	Deoxyhypus	LIA1	chr10	YJR070C	---	P47120	---	---	---	0000226 // r	0005634 // r	0004497 // r	---	---	This probe s	S000003831(11),GENEFINDER00000001141(11),GENSCAN00000004613(11),YJR070C(11)
1775640_at	402.61	P	1131.59	P	0.3558	P	YJR092W	S. cerevisiae	YJR092W.S	chr10:59842	Protein invol	BUD4	chr10	YJR092W	---	P47136	---	---	---	0000282 // c	0000142 // c	0005515 // r	---	---	This probe s	S000003852(11),GENEFINDER00000001296(11),GENSCAN00000004434(11),YJR092W(11)
1772275_at	135.65	P	483.3	P	0.2807	P	YJR097W	S. cerevisiae	YJR097W.S	chr10:61210	Protein of ur	JJJ3	chr10	YJR097W	---	P47138	---	---	---	0017183 // r	0005634 // r	0005515 // r	---	---	This probe s	S000003858(11),GENSCAN00000004166(11),YJR097W(11)
1778802_at	197.86	P	641.92	P	0.3082	P	YJR112W	S. cerevisiae	YJR112W.S	chr10:63672	Essential co	NNF1	chr10	YJR112W	---	P47149	---	---	---	0007049 // c	0000776 // k	0005515 // r	---	---	This probe s	S000003873(11),GENEFINDER00000001312(11),GENSCAN00000004756(11),YJR112W(11)
1772847_at	907.33	P	2771.83	P	0.3273	P	YKL172W	S. cerevisiae	YKL172W.S	chr11:12575	Essential pro	EBP2	chr11	YKL172W	---	P36049	---	---	---	0000280 // r	0005634 // r	0005515 // r	---	---	This probe s	S000001655(11),GENEFINDER00000001864(11),GENSCAN00000004418(11),YKL172W(11)
1771206_at	841.28	P	1779.57	P	0.4727	P	YKL165C	S. cerevisiae	YKL165C.S	chr11:13793	Protein invol	MCD4	chr11	YKL165C	---	P36051	---	---	---	0006506 // c	0000139 // (	0003824 // c	---	S000001648	This probe s	S000001648(11),GENSCAN00000004555(11),YKL165C(11)
1777032_at	450.55	P	946.57	P	0.476	P	YKL155C	S. cerevisiae	YKL155C.S	chr11:15945	Mitochondria	RSM22	chr11	YKL155C	---	P36056	---	---	---	0006412 // t	0005739 // r	0003735 // s	---	---	This probe s	S000001638(11),GENEFINDER00000001857(11),GENSCAN00000004779(11),YKL155C(11)
1774933_at	590.91	P	1651.15	P	0.3579	P	YKL143W	S. cerevisiae	YKL143W.S	chr11:17678	Component	LTV1	chr11	YKL143W	---	P34078	---	---	---	0000056 // r	0005634 // r	0005515 // r	IPR001245	GENEFINDE	This probe s	S000001626(11),GENEFINDER00000001598(10),GENSCAN00000004636(11),YKL143W(11)
1777971_at	1227.3	P	2609.44	P	0.4703	P	YKL127W	S. cerevisiae	YKL127W.S	chr11:20318	Phosphogluc	PGM1	chr11	YKL127W	---	P33401	---	---	---	0005975 // c	0005737 // c	0000287 // r	---	---	This probe s	S000001610(11),GENEFINDER00000001831(11),GENSCAN00000004689(11),YKL127W(11)
1771067_at	174.22	P	762.72	P	0.2284	P	YKL112W	S. cerevisiae	YKL112W.S	chr11:22621	DNA binding	ABF1	chr11	YKL112W	---	P14164	---	---	---	0000122 // r	0000113 // r	0003677 // I	---	---	This probe s	S000001595(11),GENEFINDER00000001637(11),GENSCAN00000004713(11),YKL112W(11)

1774503_at	398.26	P	1259.23	P	0.3163	P	YKL099C	S. cerevisiae	YKL099C S	chr11:25534	Subunit of U	UTP11	chr11	YKL099C	---	P34247	---	---	---	0000447 //	0005634 //	---	---	GENEFINDE	This probe s	S000001582(11),GENEFINDER00000001573(11),GENSCAN00000004453(11),YKL099C(11)
1777927_at	470.92	P	1464.99	P	0.3214	P	YKL078W	S. cerevisiae	YKL078W S	chr11:28848	Predominant	DHR2	chr11	YKL078W	---	P36009	---	---	---	0006364 //	0005634 //	0000166 //	IPR011545	---	This probe s	S000001561(11),GENSCAN00000004525(11),GENEFINDER00000001765(11),YKL078W(11)
1771914_at	105.56	P	213.47	P	0.4945	P	YKL072W	S. cerevisiae	YKL072W S	chr11:29922	Protein that	STB6	chr11	YKL072W	---	P36085	---	---	---	---	---	0005515 //	---	---	This probe s	S000001555(11),GENSCAN00000004271(11),YKL072W(11)
1779854_at	767.19	P	1564.93	P	0.4902	P	YKL057C	S. cerevisiae	YKL057C S	chr11:33049	Subunit of th	NUP120	chr11	YKL057C	---	P35729	---	---	---	0006406 //	0005634 //	0005198 //	---	---	This probe s	S000001540(11),GENSCAN00000004399(11),YKL057C(11)
1770941_at	217.77	P	594.59	P	0.3663	P	YKL048C	S. cerevisiae	YKL048C S	chr11:34685	Serine/threo	ELM1	chr11	YKL048C	---	P32801	---	---	---	0000135 //	0000142 //	0000166 //	IPR001245	---	This probe s	S000001531(11),GENSCAN00000004631(11),YKL048C(11)
1778963_at	506.12	P	1198.89	P	0.4222	P	YKL021C	S. cerevisiae	YKL021C S	chr11:39698	Protein invol	MAK11	chr11	YKL021C	---	P20484	---	---	---	0000027 //	0005624 //	---	---	---	This probe s	S000001504(11),GENEFINDER00000001863(11),GENSCAN00000004561(11),YKL021C(11)
1774349_at	2180.99	P	5032.97	P	0.4333	P	YKL009W	S. cerevisiae	YKL009W S	chr11:42587	Protein invol	MRT4	chr11	YKL009W	---	P33201	---	---	---	0006364 //	0005622 //	---	IPR001790	---	This probe s	S000001492(11),GENEFINDER00000001740(11),GENSCAN00000004698(11),YKL009W(11)
1778508_at	533.58	P	1638.49	P	0.3257	P	YKR024C	S. cerevisiae	YKR024C S	chr11:48478	Putative ATF	DBP7	chr11	YKR024C	---	P36120	---	---	---	0000027 //	0005634 //	0000166 //	---	---	This probe s	S000001732(11),GENEFINDER00000001563(11),GENSCAN00000004336(11),YKR024C(11)
1770407_at	891.23	P	2261.33	P	0.3941	P	YKR056W	S. cerevisiae	YKR056W S	chr11:54908	tRNA methy	TRM2	chr11	YKR056W	---	P33753	---	---	---	0006281 //	---	0000014 //	IPR002495	---	This probe s	S000001764(11),GENEFINDER00000001793(9),GENSCAN00000004541(11),YKR056W(11)
1773832_at	543.75	P	1528.76	P	0.3557	P	YKR060W	S. cerevisiae	YKR060W S	chr11:55615	Subunit of U	UTP30	chr11	YKR060W	---	P36144	---	---	---	0006364 //	0005634 //	0003723 //	---	---	This probe s	S000001768(11),GENSCAN00000004663(11),YKR060W(11)
1778491_at	477.29	P	1027.76	P	0.4644	P	YKR079C	S. cerevisiae	YKR079C S	chr11:58642	tRNA 3'-end	TRZ1	chr11	YKR079C	---	P36159	---	---	---	0008033 //	0005634 //	0004518 //	---	---	This probe s	S000001787(11),GENSCAN00000004703(11),YKR079C(11)
1771626_at	1265.27	P	3385.37	P	0.3737	P	YKR081C	S. cerevisiae	YKR081C S	chr11:59114	Essential pro	RPF2	chr11	YKR081C	---	P36160	---	---	---	0000027 //	0005634 //	0008097 //	---	---	This probe s	S000001789(11),GENEFINDER00000001645(10),GENSCAN00000004803(11),YKR081C(11)
1769945_at	1282.3	P	2799.24	P	0.4581	P	YKR092C	S. cerevisiae	YKR092C S	chr11:61229	Nucleolar, se	SRP40	chr11	YKR092C	---	P32583	---	---	---	0042254 //	0005730 //	0005515 //	---	---	This probe s	S000001800(11),GENSCAN00000004634(11),YKR092C(11)
1778653_at	610.44	P	1697.19	P	0.3597	P	YLL034C	S. cerevisiae	YLL034C S1	chr12:70631	Putative ATF	RIX7	chr12	YLL034C	---	Q07844	---	---	---	0000055 //	0005634 //	0000166 //	IPR011703	---	This probe s	S000003957(11),GENEFINDER00000003052(11),GENSCAN00000007614(11),YLL034C(11)
1777620_at	727.26	P	1466.34	P	0.496	P	YLL021W	S. cerevisiae	YLL021W S	chr12:10094	Component	SPA2	chr12	YLL021W	---	P23201	---	---	---	0007015 //	0000131 //	0005515 //	---	---	This probe s	S000003944(11),GENEFINDER00000003227(11),GENSCAN00000007617(11),YLL021W(11)
1774980_at	675.67	P	2211.92	P	0.3055	P	YLL011W	S. cerevisiae	YLL011W S	chr12:12752	Essential pro	SOF1	chr12	YLL011W	---	P33750	---	---	---	0000462 //	0005634 //	0005515 //	---	---	This probe s	S000003934(11),GENSCAN00000007190(11),GENEFINDER00000003147(11),YLL011W(11)
1773948_at	941.41	P	2268.39	P	0.415	P	YLL008W	S. cerevisiae	YLL008W S	chr12:13172	Nucleolar DE	DRS1	chr12	YLL008W	---	P32892	---	---	---	0000027 //	0005634 //	0000166 //	---	---	This probe s	S000003931(11),GENEFINDER00000003007(11),GENSCAN00000007136(11),YLL008W(11)
1774035_at	764.92	P	1809.6	P	0.4227	P	YLR002C	S. cerevisiae	YLR002C S	chr12:15434	Protein that	NOC3	chr12	YLR002C	---	Q07896	---	---	---	0006260 //	0005634 //	0003682 //	---	GENEFINDE	This probe s	S000003992(11),GENEFINDER00000002987(10),GENSCAN00000007003(11),YLR002C(11)
1772264_at	1895.19	P	3813.42	P	0.497	P	YLR009W	S. cerevisiae	YLR009W S	chr12:16653	Essential pro	RLP24	chr12	YLR009W	---	Q07915	---	---	---	0006412 //	0005622 //	0003735 //	---	---	This probe s	S000003999(11),GENEFINDER00000003039(11),YLR009W(11)
1777669_at	326.4	P	2081.03	P	0.1568	P	YLR042C	S. cerevisiae	YLR042C S	chr12:22996	---	---	chr12	YLR042C	---	Q07990	---	---	---	---	0005576 //	---	---	---	This probe s	S000004032(11),GENSCAN00000007569(11),YLR042C(11)
1775172_at	732.72	P	1613.15	P	0.4542	P	YLR051C	S. cerevisiae	YLR051C S	chr12:24632	Essential nu	FCF2	chr12	YLR051C	---	Q12035	---	---	---	0000447 //	0005634 //	---	---	---	This probe s	S000004041(11),GENEFINDER00000003050(11),GENSCAN00000007558(11),YLR051C(11)
1771069_at	334.75	P	970.1	P	0.3451	P	YLR063W	S. cerevisiae	YLR063W S	chr12:26415	---	---	chr12	YLR063W	---	Q12291	---	---	---	---	0005737 //	---	---	---	This probe s	S000004053(11),GENSCAN00000007509(11),YLR063W(11)
1777204_at	374.31	P	947.84	P	0.3949	P	YLR068W	S. cerevisiae	YLR068W S	chr12:27100	Essential pro	FYV7	chr12	YLR068W	---	Q12247	---	---	---	0000462 //	0005634 //	0005515 //	---	---	This probe s	S000004058(11),GENEFINDER00000003063(11),GENSCAN00000007517(11),YLR068W(11)
1775884_at	320.91	P	795.75	P	0.4033	P	YLR084C	S. cerevisiae	YLR084C S	chr12:29658	N-glycosylat	RAX2	chr12	YLR084C	---	Q12465	---	---	---	0000282 //	0005621 //	---	---	S00000407	This probe s	S000004074(11),GENEFINDER00000003085(10),GENSCAN00000007535(11),YLR084C(11)
1771269_at	202.8	P	528.38	P	0.3838	P	YLR103C	S. cerevisiae	YLR103C S	chr12:34398	DNA replicat	CDC45	chr12	YLR103C	---	Q08032	---	---	---	0000084 //	0005634 //	0003682 //	---	---	This probe s	S000004093(11),GENEFINDER00000003141(11),GENSCAN00000007133(11),YLR103C(11)
1777632_at	981.62	P	2297.4	P	0.4273	P	YLR129W	S. cerevisiae	YLR129W S	chr12:39965	Nucleolar pro	DIP2	chr12	YLR129W	---	Q12220	---	---	---	0000447 //	0005634 //	0005515 //	---	---	This probe s	S000004119(11),GENEFINDER00000003208(11),GENSCAN00000007462(11),YLR129W(11)
1777290_at	11.01	A	38.35	P	0.2871	P	YLR157W-C	S. cerevisiae	YLR157W-C	chr12:48187	---	---	chr12	YLR157W-E	---	Q12478	---	---	---	---	---	0005515 //	---	---	This probe s	S000028678(11),YLR157W-E(11)
1774947_at	325.72	P	671.26	P	0.4852	P	YLR183C	S. cerevisiae	YLR183C S	chr12:52054	Forkhead As	TOS4	chr12	YLR183C	---	Q06266	---	---	---	0034984 //	0000790 //	0003682 //	---	---	This probe s	S000004173(11),GENSCAN00000007035(11),YLR183C(11)
1774541_at	1386.1	P	2869.68	P	0.483	P	YLR196W	S. cerevisiae	YLR196W S	chr12:54396	Protein with	PWP1	chr12	YLR196W	---	P21304	---	---	---	0006364 //	0005634 //	0005515 //	---	---	This probe s	S000004186(11),GENEFINDER00000003064(11),GENSCAN00000007120(11),YLR196W(11)
1772730_at	60.82	P	130.44	P	0.4663	P	YLR213C	S. cerevisiae	YLR213C S	chr12:56665	Putative glyc	CRR1	chr12	YLR213C	---	Q05790	---	---	---	0005975 //	0005619 //	0004553 //	---	---	This probe s	S000004203(11),GENEFINDER00000003045(11),GENSCAN00000007472(11),YLR213C(11)
1771392_at	1680.07	P	3827.55	P	0.4389	P	YLR214W	S. cerevisiae	YLR214W S	chr12:56856	Ferric reduc	FRE1	chr12	YLR214W	---	P32791	---	---	---	0006810 //	0005624 //	0000293 //	---	S00000420	This probe s	S000004204(11),GENSCAN00000007570(11),YLR214W(11)
1771304_at	652.65	P	1795.3	P	0.3635	P	YLR222C	S. cerevisiae	YLR222C S	chr12:57931	Nucleolar pro	UTP13	chr12	YLR222C	---	Q05946	---	---	---	0000447 //	0005634 //	0005515 //	---	---	This probe s	S000004212(11),GENEFINDER00000003019(11),GENSCAN00000007377(11),YLR222C(11)
1774453_at	1763.76	P	3700.96	P	0.4766	P	YLR276C	S. cerevisiae	YLR276C S	chr12:69504	ATP-depend	DBP9	chr12	YLR276C	---	Q06218	---	---	---	0000027 //	0005634 //	0000166 //	---	---	This probe s	S000004266(11),GENEFINDER00000003055(11),GENSCAN00000007358(11),YLR276C(11)
1779963_at	339.16	P	777.79	P	0.4361	P	YLR287C	S. cerevisiae	YLR287C S	chr12:71099	---	---	chr12	YLR287C	---	Q05881	---	---	---	---	0005737 //	0005515 //	---	---	This probe s	S000004277(11),GENSCAN00000007481(11),YLR287C(11)
1770607_at	626.07	P	1384.93	P	0.4521	P	YLR336C	S. cerevisiae	YLR336C S	chr12:79969	Essential nu	SGD1	chr12	YLR336C	---	Q06132	---	---	---	0006972 //	0005634 //	0005488 //	---	---	This probe s	S000004328(11),GENSCAN00000007576(11),YLR336C(11)
1774282_at	653.14	P	1342.3	P	0.4866	P	YLR348C	S. cerevisiae	YLR348C S	chr12:82697	Mitochondria	DIC1	chr12	YLR348C	---	Q06143	---	---	---	0006810 //	0005739 //	0005310 //	---	GENEFINDE	This probe s	S000004340(11),GENEFINDER00000003196(10),GENSCAN00000007347(11),YLR348C(11)
1770954_at	703.85	P	1547.59	P	0.4548	P	YLR363W-A	S. cerevisiae	YLR363W-A	chr12:85345	---	---	chr12	YLR363W-A	---	Q3E747	---	---	---	---	0005634 //	---	---	---	This probe s	S000007620(11),YLR363W-A(11)
1776118_at	1258.39	P	2783.02	P	0.4522	P	YLR409C	S. cerevisiae	YLR409C S	chr12:93440	Subunit of U	UTP21	chr12	YLR409C	---	Q06078	---	---	---	0006364 //	0005634 //	0005515 //	---	---	This probe s	S000004401(11),GENEFINDER00000003205(11),GENSCAN00000007561(11),YLR409C(11)
1779745_at	529.72	P	1678.02	P	0.3157	P	YLR435W	S. cerevisiae	YLR435W S	chr12:10063	Protein with	TSR2	chr12	YLR435W	---	Q06672	---	---	---	0000462 //	0005634 //	0005515 //	---	---	This probe s	S000004427(11),GENSCAN00000007654(11),GENEFINDER00000003115(11),YLR435W(11)
1778277_at	788.57	P	1822.25	P	0.4327	P	YLR452C	S. cerevisiae	YLR452C S	chr12:10392	GTPase-act	SST2	chr12	YLR452C	---	P11972	---	---	---	0000754 //	0005886 //	0004871 //	---	---	This probe s	S000004444(11),GENEFINDER00000003044(11),GENSCAN00000007266(11),YLR452C(11)
1770297_at	919.45	P	2196.52	P	0.4186	P	YML093W	S. cerevisiae	YML093W S	chr13:83089	Subunit of U	UTP14	chr13	YML093W	---	Q04500	---	---	---	0000447 //	0005634 //	0000166 //	---	---	This probe s	S000004558(11),GENEFINDER00000001903(11),GENSCAN00000006418(11),YML093W(11)
1773118_at	303.89	P	1015.79	P	0.2992	P	YML080W	S. cerevisiae	YML080W S	chr13:10880	Dihydrouridi	DUS1	chr13	YML080W	---	P53759	---	---	---	0006400 //	0005634 //	0003824 //	---	---	This probe s	S000004545(11),GENSCAN00000006224(11),GENEFINDER00000001715(11),YML080W(11)
1770816_at	368.75	P	749.24	P	0.4922	P	YML065W	S. cerevisiae	YML065W S	chr13:14220	Largest subu	ORC1	chr13	YML065W	---	P54784	---	---	---	0006260 //	0005634 //	0000166 //	---	---	This probe s	S000004530(11),GENEFINDER00000001835(11),GENSCAN00000006158(11),YML065W(11)
1772101_at	257.21	P	655.95	P	0.3921	P	YML060W	S. cerevisiae	YML060W S	chr13:15187	Mitochondria	OGG1	chr13	YML060W	---	P53397	---	---	---	0006281 //	0005634 //	0003677 //	---	---	This probe s	S000004525(11),GENSCAN00000006167(11),YML060W(11)
1777384_at	25.85	P	548.03	P	0.0472	P	YML047C	S. cerevisiae	YML047C S	chr13:18001	Pheromone-	PRM6	chr13	YML047C	---	Q04705	---	---	---	0000747 //	0016020 //	0005515 //	---	S00000451	This probe s	S000004510(11),YML047C(11)
1774092_at	151.76	P	699.91	P	0.2168	P	YML043C	S. cerevisiae	YML043C S	chr13:19024	Protein regu	RRN11	chr13	YML043C	---	Q04712	---	---	---	0006350 //	0000120 //	0003701 //	---	---	This probe s	S000004507(11),GENSCAN00000006678(11),YML043C(11)
1775720_at	511.81	P	1490.75	P	0.3433	P	YML027W	S. cerevisiae	YML027W S	chr13:22140	Homeodoma	YOX1	chr13	YML027W	---	P34161	---	---	---	0000122 //	0000790 //	0003677 //	---	---	This probe s	S000004489(11),GENEFINDER00000001939(11),GENSCAN00000006267(11),YML027W(11)
1776115_at	389.75	P	848.41	P	0.4594	P	YML014W	S. cerevisiae	YML014W S	chr13:24322	tRNA methy	TRM9	chr13	YML014W	---	P49957	---	---	---	0006950 //	0005634 //	0005515 //	---	---	This probe s	S000004476(11),GENEFINDER00000001933(9),GENSCAN00000006403(11),YML014W(11)
1775593_at	425.02	P	1174.72	P	0.3618	P	YMR001C	S. cerevisiae	YMR001C S	chr13:26901	Polo-like kin	CDC5	chr13	YMR001C	---	P32562	---	---	---	0000712 //	0000922 //	0000166 //	---	---	This probe s	S000004603(11),GENSCAN00000006721(11),YMR001C(11)
1778753_at	75.56	P	271.45	P	0.2784	P	YMR001C-A	S. cerevisiae	YMR001C-A	chr13:27134	---	---	chr13	YMR001C-A	---	Q8TGS9	---	---	---	---	---	---	---	---	This probe s	S000028691(11),YMR001C-A(11)
1769576_at	292.2	P	937.54	P	0.3117	P	YMR003W	S. cerevisiae	YMR003W S	chr13:27311	Protein of ur	AIM34	chr13	YMR003W	---	Q03673	---	---	---	---	0005739 //	0003676 //	---	---	This probe s	S000004605(11),YMR003W(11)
1772828_at	193.63	P	666.54	P	0.2905	P	YMR014W	S. cerevisiae	YMR014W S	chr13:29886	Protein invol	BUD22	chr13	YMR014W	---	Q04347	---	---	---	0000282 //	0005634 //	---	---	---	This probe s	S000004616(11),GENEFINDER00000001557(11),GENSCAN00000006245(11),YMR014W(11)
1779961_at	791.91	P	3596.35	P	0.2202	P	YMR015C	S. cerevisiae	YMR015C S	chr13:30086	C-22 sterol	ERG5	chr13	YMR015C	---	P54781 /// C	---	---	---	0006694 //	---	0004497 //	---	---	This probe s	S000004617(11),GENEFINDER00000001744(11),GENSCAN00000006542(11),YMR015C(11)
1777701_at	234.24	P	694.99	P	0.337	P	YMR032W	S. cerevisiae	YMR032W S	chr13:33529	Bud neck-loc	HOF1	chr13	YMR032W	---	Q05080	---	---	---	0000910 //	0000142 //	0005515 //	IPR001452	---	This probe s	S000004635(11),GENEFINDER00000001739(11),GENSCAN00000006681(11),YMR032W(11)
1775937_at	271.74	P	683.35	P	0.3977	P	YMR030W-A	S. cerevisiae	YMR030W-A	chr13:33731	---	---	chr13	YMR030W-A	---	Q3E760	---	---	---	---	0016020 //	---	---	S00002857	This probe s	S000028574(11),YMR030W-A(11)
1769865_at	113.29	P	257.12	P	0.4406	P	YMR048W	S. cerevisiae	YMR048W S	chr13:36697	Protein regu	CSM3	chr13	YMR048W	---	Q04659	---	---	---	0000076 //	0005634 //	0005515 //	---	---	This probe s	S000004651(11),GENEFINDER00000001962(11),GENSCAN00000006280(11),YMR048W(11)
1769597_at	1430.79	P	3110.31	P	0.46	P	YMR049C	S. cerevisiae	YMR049C S	chr13:36809	Protein regu	ERB1	chr13	YMR049C	---	Q04660	---	---	---	0006364 //	0005634 //	---	---	---	This probe s	S000004652(11),GENEFINDER00000001931(11),GENSCAN00000006288(11),YMR049C(11)
1773089_at	2490.95	P	5467.21	P	0.4556	P	YMR058W	S. cerevisiae	YMR058W S	chr13:38882	Ferro-O2-ox	FET3	chr13	YMR058W	---	P38993	---	---	---	0006810 //	0005886 //	0004322 //	---	S00000466	This probe s	S000004662(11),GENEFINDER00000001967(11),GENSCAN00000006199(11),YMR058W(11)
1769420_at	546.59	P	1137.23	P	0.4806	P	YMR076C	S. cerevisiae	YMR076C S	chr13:41619	Protein regu	PDS5	chr13	YMR076C	---	Q04264	---	---	---	0007049 //	0000794 //	0005488 //	---	---	This probe s	S000004681(11),GENSCAN00000006796(11),YMR076C(11)
1771500_at	213.52	P	584.72	P	0.3652	P	YMR078C	S. cerevisiae	YMR078C S	chr13:42250	Subunit of a	CTF18	chr13	YMR078C	---	P49956	---	---	---	0000724 //	0005634 //	0000166 //	---	---	This probe s	S000004683(11),GENSCAN00000006217(11),YMR078C(11)
1776498_at	1293.95	P	3158.45	P	0.4097	P	YMR093W	S. cerevisiae	YMR093W S	chr13:45401	Nucleolar pro	UTP15	chr13	YMR093W	---	Q04305	---	---	---	0000462 //	0005634 //	0005515 //	---	---	This probe s	S000004699(11),GENEFINDER00000001584(11),GENSCAN00000006634(11),YMR093W(11)
1773187_at	145.36	P	293.95	P	0.4945	P	YMR106C	S. cerevisiae	YMR106C S	chr13:47829	Subunit of th	YKU80	chr13	YMR106C	---	Q04437	---	---	---	0000723 //	0000781 //	0003677 //	---	---	This probe s	S000004712(11),GENEFINDER00000001943(11),GENSCAN00000006093(11),YMR106C(11)
1778193_at	18.48	P	58.85	P	0.314	P	YMR117C	S. cerevisiae	YMR117C S	chr13:50124	Component	SPC24	chr13	YMR117C	---	Q04477	---	---	---	0007020 //	0000777 //	0005200 //	---	---	This probe s	S000004723(11),GENSCAN00000006066(11),YMR117C(11)
1769890_at	1911.78	P	3881.37	P	0.4926	P	YMR131C	S. cerevisiae	YMR131C S	chr13:53316	Essential nu	RRB1	chr13	YMR131C	---	Q04225	---	---	---	0006364 //	0005634 //	---	---	---	This probe s	S000004738(11),GENEFINDER00000001945(11),GENSCAN00000006803(11),YMR131C(11)
1773256_at	189.13	P	437.58	P	0.4322	P	YMR144W	S. cerevisiae	YMR144W S	chr13:55336	---	---	chr13	YMR144W	---	P40214	---	---	---	---	0005634 //	---	---	---	This probe s	S000004752(11),GENEFINDER00000001602(11),GENSCAN00000006607(11),YMR144W(11)
1779922_at	1575.13	P	6127.72	P	0.257	P	YMR189W	S. cerevisiae	YMR189W S	chr13:63749	P subunit of	GCV2	chr13	YMR189W	---	P49095	---	---	---	0006544 //	0005739 //	0004375 //	---	---	This probe s	S000004801(11),GENEFINDER00000001956(11),GENSCAN00000006716(11),YMR189W(11)
1775458_at	109.11	P	369.33	P	0.2954	P	YMR198W	S. cerevisiae	YMR198W S	chr13:65974	Kinesin-asso	CIK1	chr13	YMR198W	---	Q01649	---	---	---	0000070 //	0005634 //	0003777 //	---	---	This probe s	S000004811(11),GENEFINDER00000001592(11),GENSCAN00000006826(11),YMR198W(11)
1773591_at	605.02	P	1459.02	P	0.4147	P	YMR199W	S. cerevisiae	YMR199W S	chr13:66264	G1 cyclin inv	CLN1	chr13	YMR199W	---	P20437	---	---	---	0007049 //	0005634 //	0005515 //	---	---	This probe s	S000004812(11),GENSCAN00000006112(11),YMR199W(11)
1773766_at	1887.91	P	4534.08	P	0.4164	P	YMR215W	S. cerevisiae	YMR215W S	chr13:69679	Putative 1,3-	GAS3	chr13	YMR215W	---	Q03655	---	---	---	0005975 //	0005624 //	0003824 //	---	---	This probe s	S000004828(11),GENEFINDER00000001595(11),GENSCAN00000006257(11),YMR215W(11)
1777228_at	170.01	P	346.46	P	0.4907	P	YMR230W-A	S. cerevisiae	YMR230W-A	chr13:73326	---	---	chr13	YMR230W-A	---	Q3E7B5	---	---	---	---	---	---	---	---	This probe s	S000028576(11),YMR230W-A(11)
1770943_at	375.53	P	1063.44	P	0.3531	P	YMR239C	S. cerevisiae	YMR239C S	chr13:74826	RNAase III;	RNT1	chr13	YMR239C	---	Q02555	---	---	---	0006325 //	0005622 //	0003723 //	---	---	This probe s	S000004852(11),GENEFINDER00000001613(11),GENSCAN00000006491(11),YMR239C(11)
1776254_at	167.29	P	349.7	P	0.4784	P	YMR268C	S. cerevisiae	YMR268C S	chr13:80288	Splicing fact	PRP24	chr13	YMR268C	---	P49960 /// C	---	---	---	0000244 //	0005634 //	0000166 //	---	---	This probe s	S000004881(11),GENSCAN00000006729(11),YMR268C(11)
1775431_at	280.88	P	693.25	P	0.4052	P	YMR269W	S. cerevisiae	YMR269W S	chr13:80445	Nucleolar pro	TMA23	chr13	YMR269W	---	Q03525	---	---	---	0042274 //	0005634 //	0005515 //	---	---	This probe s	S000004882(11),GENSCAN00000006873(11),YMR269W(11)
1775736_at	1814.4	P	4465.96	P	0.4063	P	YMR300C	S. cerevisiae	YMR300C S	chr13:86555	Phosphoribo	ADE4	chr13	YMR300C	---	P04046	---	---	---	0006164 //	0005737 //	0000287 //	---	---	This probe s	S000004915(11),GENEFINDER00000001911(11),GENSCAN00000006466(11),YMR300C(11)
1779680_at	729.4	P	1862.81	P	0.3916	P	YMR310C	S. cerevisiae	YMR310C S	chr13:89571	---	---	chr13	YMR310C	---	Q04867	---	---	---	---	0005634 //	---	---	---	This probe s	S000004927(11),GENSCAN00000006947(11),YMR310C(11)
1770257_at	427.76	P	1159.91	P	0.3688	P	YNL313C	S. cerevisiae	YNL313C S	chr14:45306	---	---	chr14	YNL313C	---	P42842	---	---	---	---	0005634 //	0005515 //	IPR001440	---	This probe s	S000005257(11),GENEFINDER00000000870(11),GENSCAN00000004929(11),YNL313C(11)
1774693_at	1466	P	2973.76	P	0.493	P	YNL308C	S. cerevisiae	YNL308C S	chr14:54120	Essential nu	KRI1	chr14	YNL308C	---	P42846	---	---	---	0000447 //	0005634 //	0005515 //	---	---	This probe s	S000005252(11),GENEFINDER00000000779(11),GENSCAN00000005042(11),YNL308C(11)
1777922_at	2565.13	P	5575.39	P	0.4601	P	YNL300W	S. cerevisiae	YNL300W S	chr14:65742	Glycosylpho	TOS6	chr14	YNL300W	---	P48560	---	---	---	---	0009277 //	---	---	S00000524	This probe s	S000005244(11),GENEFINDER00000000717(11),YNL300W(11)
1779804_at	238.93	P	703.09	P	0.3398	P	YNL299W	S. cerevisiae	YNL299W S	chr14:66515	Poly (A) poly	TRF5	chr14	YNL299W	---	P48561	---	---	---	0006281 //	0005634 //	0003887 //	---	---	This probe s	S000005243(11),GENEFINDER00000000747(11),GENSCAN00000005363(11),YNL299W(11)
1769484_at	619.67	P	1266.48	P	0.4893	P	YNL292W	S. cerevisiae	YNL292W S	chr14:82803	Pseudouridi	PUS4	chr14	YNL292W	---	P48567	---	---	---	0001522 //	0005634 //	0005515 //	---	---	This probe s	S000005236(11),GENEFINDER00000000697(11),GENSCAN00000005084(11),YNL292W(11)
1778319_at	352.67	P	1165.77	P	0.3025	P	YNL289W	S. cerevisiae	YNL289W S	chr14:87894	Pho85 cyclin	PCL1	chr14	YNL289W	---	P24867	---	---	---	0000079 //	0000131 //	0005515 //	---	---	This probe s	S000005233(11),GENSCAN00000004990(11),YNL289W(11)
1775220_at	96.26	P	553.44	P	0.1739	P	YNL279W	S. cerevisiae	YNL279W S	chr14:11091	Pheromone-	PRM1	chr14	YNL279W	---	P53835	---	---	---	0000746 //	0005886 //	---	---	S00000522	This probe s	S000005223(11),GENEFINDER00000000882(11),GENSCAN00000004882(11),YNL279W(11)
1779813_at	1426.96	P	3186.78	P	0.4478	P	YNL248C	S. cerevisiae	YNL248C S	chr14:18135	RNA polyme	RPA49	chr14	YNL248C	---	Q01080	---	---	---	0006350 //	0005634 //	0003677 //	---	---	This probe s	S000005192(11),GENEFINDER00000000723(11),GENSCAN00000005373(11),YNL248C(11)
1777481_at	191.29	P	407.84	P	0.469	P	YNL227C	S. cerevisiae	YNL227C S	chr14:22065	Co-chapero	JJJ1	chr14	YNL227C	---	P53863	---	---	---	0006897 //	0005622 //	0001671 //	---	---	This probe s	S000005171(11),GENSCAN00000004974(11),YNL227C(11)
1776399_at	137.14	P	379.26	P	0.3616	P	YNL218W	S. cerevisiae	YNL218W S	chr14:23823	Protein with	MGS1	chr14	YNL218W	---	P40151	---	---	---	0006281 //	0005634 //	0000166 //	IPR003959	---	This probe s	S000005162(11),GENEFINDER00000000806(11),GENSCAN00000005279(11),YNL218W(11)
1775669_at	621.86	P	1328.57	P	0.4681	P	YNL207W	S. cerevisiae	YNL207W S	chr14:25534	Essential se	RIO2	chr14	YNL207W	---	P40160	---	---	---	0000462 //	0005634 //	0000166 //	---	---	This probe s	S000005151(11),GENEFINDER00000000727(11),GENSCAN00000005291(11),YNL207W(11)
1780099_at	499.73	P	1085.2	P	0.4605	P	YNL197C	S. cerevisiae	YNL197C S	chr14:26760	RNA binding	WHI3	chr14	YNL197C	---	P34761	---	---	---	0001403 //	0005737 //	0000166 //	---	---	This probe s	S000005141(11),GENEFINDER00000000778(11),GENSCAN00000004953(11),YNL197C(11)
1779372_at	135.69	P	293.17	P	0.4628	P	YNL188W	S. cerevisiae	YNL188W S	chr14:28630	Essential pro	KAR1	chr14	YNL188W	---	P11927 /// C	---	---	---	0000742 //	0005825 //	0005515 //	---	S00000513	This probe s	S000005132(11),GENEFINDER00000000691(11),GENSCAN00000004890(11),YNL188W(11)
1772349_at	244.92	P	881.43	P	0.2779	P	YNL182C	S. cerevisiae	YNL182C S	chr14:29595	Essential co	IPI3	chr14	YNL182C	---	P53877	---	---	---	0000027 //	0005634 //	0005515 //	---	---	This probe s	S000005126(11),GENSCAN00000004866(11),YNL182C(11)
1773605_at	761.76	P	2328.46	P	0.3272	P	YNL175C	S. cerevisiae	YNL175C S	chr14:30739	Nucleolar pro	NOP13	chr14	YNL175C	---	P53883	---	---	---	---	0005634 //	0000166 //	---	---	This probe s	S000005119(11),GENEFINDER00000000863(11),GENSCAN00000004877(11),YNL175C(11)
1772105_at	432.71	P	938.12	P	0.4613	P	YNL166C	S. cerevisiae	YNL166C S	chr14:32221	Protein invol	BNI5	chr14	YNL166C	---	P53890	---	---	---	0000910 //	0000144 //	0042802 //	---	---	This probe s	S000005110(11),GENEFINDER00000000852(11),GENSCAN00000005361(11),YNL166C(11)
1777214_at	122.2	P	345.23	P	0.354	P	YNL162W-A	S. cerevisiae	YNL162W-A	chr14:33032	---	---	chr14	YNL162W-A	---	Q3E7A8	---	---	---	---	0005634 //	---	---	---	This probe s	S000007624(11),YNL162W-A(11)
1777944_at	1020.41	P	2094.03	P	0.4873	P	YNL151C	S. cerevisiae	YNL151C S	chr14:34776	RNA polyme	RPC31	chr14	YNL151C	---	P17890	---	---	---	0006350 //	0005634 //	0003677 //	---	---	This probe s	S000005095(11),GENEFINDER00000000798(11),GENSCAN00000004962(11),YNL151C(11)
1779946_at	2411.67	P	5197.45	P	0.464	P	YNL141W	S. cerevisiae	YNL141W S	chr14:35959	Adenine dea	AAH1	chr14	YNL141W	---	P53909	---	---	---	0006146 //	0005634 //	0000034 //	---	---	This probe s	S000005085(11),GENSCAN00000005149(11),YNL141W(11)
1769336_at	138.9	P	332.92	P	0.4172	P	YNL126W	S. cerevisiae	YNL126W S	chr14:38722	Component	SPC98	chr14	YNL126W	---	P53540	---	---	---	0000226 //	0000922 //	0005200 //	---	---	This probe s	S000005070(11),GENSCAN00000005102(11),YNL126W(11)
1773892_at	517.19	P	1276.38	P	0.4052	P	YNL119W	S. cerevisiae	YNL119W S	chr14:40103	Protein regu	NCS2	chr14	YNL119W	---	P53923	---	---	---	0001403 //	0005737 //	0005515 //	---	---	This probe s	S000005063(11),GENSCAN00000004983(11),YNL119W(11)
1771542_at	1137.62	P	3014.74	P	0.3774	P	YNL113W	S. cerevisiae	YNL113W S	chr14:41276	RNA polyme	RPC19	chr14	YNL113W	---	P28000	---	---	---	0006350 //	0005634 //	0003677 //	---	---	This probe s	S000005057(11),GENEFINDER00000000826(11),GENSCAN00000005281(11),YNL113W(11)
1771499_at	680.94	P	2164.33	P	0.3146	P	YNL111C	S. cerevisiae	YNL111C S	chr14:41693	Cytochrome	CYB5	chr14	YNL111C	---	P40312	---	---	---	0006810 //	0005783 //	0005506 //	---	S00000505	This probe s	S000005055(11),GENEFINDER00000000789(10),GENSCAN00000005289(11),YNL111C(11)
1778347_at	1218.36	P	3206.65	P	0.3799	P	YNL110C	S. cerevisiae	YNL110C S	chr14:41782	Constituent	NOP15	chr14	YNL110C	---	P53927	---	---	---	0000915 //	0005634 //	0000166 //	---	GENEFINDE	This probe s	S000005054(11),GENEFINDER00000000789(11),GENSCAN00000005087(11),YNL110C(11)
1776526_at	797.39	P	1712.57	P	0.4656	P	YNL102W	S. cerevisiae	YNL102W S	chr14:43008	Catalytic sub	POL1	chr14	YNL102W	---	P13382 /// C	---	---	---	0000731 //	0005634 //	0000166 //	---	---	This probe s	S000005046(11),GENSCAN00000004966(11),YNL102W(11)
1777850_at	21.1	P	43.32	P	0.4871	P	YNL097C-A	S. cerevisiae	YNL097C-A	chr14:44079	---	---	chr14	YNL097C-B	---	P0C271	---	---	---	---	---	---	---	---	This probe s	S000028699(11),YNL097C-B(11)
1779535_at	752.72	P	2117.29	P	0.3555	P	YNL075W	S. cerevisiae	YNL075W S	chr14:48560	Component	IMP4	chr14	YNL075W	---	P53941	---	---	---	0006364 //	0005634 //	0030515 //	IPR001305	---	This probe s	S000005019(11),GENEFINDER00000000684(9),GENSCAN00000005236(11),YNL075W(11)
1775076_at	203.27	P	499.62	P	0.4068	P	YNL072W	S. cerevisiae	YNL072W S	chr14:49031	Ribonucleas	RNH201	chr14	YNL072W	---	P53942	---	---	---	0016070 //	0005634 //	0003676 //	---	---	This probe s	S000005016(11),GENEFINDER00000000800(11),GENSCAN00000005387(11),YNL072W(11)
1772723_at	778.39	P	2147.04	P	0.3625	P	YNL062C	S. cerevisiae	YNL062C S	chr14:50877	Subunit of tR	GCD10	chr14	YNL062C	---	P41814	---	---	---	0006413 //	0005634 //	0000049 //	---	---	This probe s	S000005006(11),GENSCAN00000005220(11),YNL062C(11)
1776088_at	263.21	P	1030.94	P	0.2553	P	YNR009W	S. cerevisiae	YNR009W S	chr14:64268	Transcriptio	NRM1	chr14	YNR009W	---	P53718	---	---	---	0006350 //	0005634 //	0016564 //	---	GENEFINDE	This probe s	S000005292(11),GENEFINDER00000000838(11),GENSCAN00000005046(11),YNR009W(11)
1772925_at	151.13	P	307.69	P	0.4912	P	YNR011C	S. cerevisiae	YNR011C S	chr14:64431	RNA-depend	PRP2	chr14	YNR011C	---	P20095	---	---	---	0000349 //	0005634 //	0000166 //	---	---	This probe s	S000005294(11),GENSCAN00000004932(11),YNR011C(11)
1778591_at	1149.67	P	5970.89	P	0.1925	P	YNR044W	S. cerevisiae	YNR044W S	chr14:70369	Anchorage s	AGA1	chr14	YNR044W	---	P32323	---	---	---	0000752 //	0005576 //	0005515 //	---	---	This probe s	S000005327(11),YNR044W(11)
1779287_at	1890.63	P	3859.91	P	0.4898	P	YNR053C	S. cerevisiae	YNR053C S	chr14:72111	Putative GTI	NOG2	chr14	YNR053C	---	P53742	---	---	---	0000055 //	0005622 //	0000166 //	---	---	This probe s	S000005336(11),YNR053C(11)
1774175_at	349.65	P	840.84	P	0.4158	P	YNR054C	S. cerevisiae	YNR054C S	chr14:72335	Essential nu	ESF2	chr14	YNR054C	---	P53743	---	---	---	0000447 //	0005634 //	0001671 //	---	---	This probe s	S000005337(11),GENEFINDER00000000836(11),GENSCAN00000004867(11),YNR054C(11)
1779790_at	32.69	P	69.23	P	0.4722	P	YNR063W	S. cerevisiae	YNR063W S	chr14:74693	---	---	chr14	YNR063W	---	P53749	---	---	---	0006350 //	0005634 //	0003677 //	IPR007219	---	This probe s	S000005346(11),GENSCAN00000004920(11),YNR063W(11)
1772699_at	382.42	P	1216.98	P	0.3142	P	YOL152W	S. cerevisiae	YOL152W S	chr15:40746	Putative ferr	FRE7	chr15	YOL152W	---	Q12333	---	---	---	0006810 //	0005886 //	0000293 //	---	S00000551	This probe s	S000005512(11),GENSCAN00000007279(11),YOL152W(11)
1770056_at	342.38	P	707.9	P	0.4837	P	YOL145C	S. cerevisiae	YOL145C S	chr15:49552	Component	CTR9	chr15	YOL145C	---	P89105	---	---	---	0006350 //	0005634 //	0003677 //	---	---	This probe s	S000005505(11),GENEFINDER00000002187(11),GENSCAN00000006592(11),YOL145C(11)
1771518_at	437.48	P	1304.73	P	0.3353	P	YOL144W	S. cerevisiae	YOL144W S	chr15:53095	Nucleolar pro	NOP8	chr15	YOL144W	---	Q08287	---	---	---	0042254 //	0005634 //	0000166 //	---	---	This probe s	S000005504(11),GENSCAN00000007151(11),GENEFINDER00000002186(11),YOL144W(11)
1778364_at	38.69	P	84.15	P	0.4598	P	YOL141W	S. cerevisiae	YOL141W S	chr15:56449	AdoMet-dep	PPM2	chr15	YOL141W	---	Q08282	---	---	---	0008033 //	0005737 //	0008168 //	---	---	This probe s	S000005501(11),GENEFINDER00000002086(11),GENSCAN00000007079(9),YOL141W(11)
1778811_at	706.4	P	1808.89	P	0.3905	P	YOL124C	S. cerevisiae	YOL124C S	chr15:85454	Catalytic sub	TRM11	chr15	YOL124C	---	Q12463	---	---	---	0008033 //	0005737 //	0003676 //	---	---	This probe s	S000005484(11),GENEFINDER00000002130(11),GENSCAN00000006555(11),YOL124C(11)
1778073_at	350.54	P	758.45	P	0.4622	P	YOL093W	S. cerevisiae	YOL093W S	chr15:14281	tRNA methy	TRM10	chr15	YOL093W	---	Q12400	---	---	---	0008033 //	0005634 //	0008168 //	---	---	This probe s	S000005453(11),GENEFINDER00000002055(11),GENSCAN00000006608(11),YOL093W(11)
1773490_at	326.21	P	1150.51	P	0.2835	P	YOL080C	S. cerevisiae	YOL080C S	chr15:18055	Putative RNA	REX4	chr15	YOL080C	---	Q08237	---	---	---	0000027 //	0005622 //	0003676 //	---	---	This probe s	S000005440(11),GENSCAN00000007172(11),YOL080C(11)
1773840_at	217.99	P	575.01	P	0.3791	P	YOL076W	S. cerevisiae	YOL076W S	chr15:18702	Non-catalyti	MDM20	chr15	YOL076W	---	Q12387	---	---	---	0000001 //	0005737 //	0004596 //	---	---	This probe s	S000005436(11),GENSCAN00000007258(11),YOL076W(11)
1773340_at	1040.82	P	2807.26	P	0.3708	P	YOL041C	S. cerevisiae	YOL041C S	chr15:25126	Nucleolar pro	NOP12	chr15	YOL041C	---	Q08208	---	---	---	0000463 //	0005634 //	0000166 //	---	---	This probe s	S000005401(11),GENEFINDER00000002082(11),GENSCAN00000006582(11),YOL041C(11)
1773154_at	417.64	P	1409.33	P	0.2963	P	YOL010W	S. cerevisiae	YOL010W S	chr15:30793	Subunit of U	RCL1	chr15	YOL010W	---	Q08096	---	---	---	0000447 //	0005634 //	0003824 //	---	---	This probe s	S000005370(11),GENEFINDER00000002228(11),GENSCAN00000007367(11),YOL010W(11)
1778296_at	610.95	P	1718.91	P	0.3554	P	YOL007C	S. cerevisiae	YOL007C S	chr15:31134	Protein of ur	CSI2	chr15	YOL007C	---	Q08054	---	---	---	---	0000324 //	---	---	S00000536	This probe s	S000005367(11),GENEFINDER00000002127(11),GENSCAN00000006750(11),YOL007C(11)
1773669_at	1030.57	P	3122.4	P	0.3301	P	YOR004W	S. cerevisiae	YOR004W S	chr15:33359	Essential nu	UTP23	chr15	YOR004W	---	Q12339	---	---	---	0000447 //	0005634 //	0003723 //	---	---	This probe s	S000005530(11),GENEFINDER00000002185(10),GENSCAN00000006703(11),YOR004W(11)
1778569_at	9.3	P	19.17	P	0.4851	P	YOR008C-A	S. cerevisiae	YOR008C-A	chr15:34285	---	---	chr15	YOR008C-A	---	Q3E7B9	---	---	---	---	0016020 //	---	---	S00000643	This probe s	S000006431(11),YOR008C-A(11)
1770015_at	8.42	P	18.16	P	0.4637	P	YOR011W-A	S. cerevisiae	YOR011W-A	chr15:35565	---	---	chr15	YOR011W-A	---	Q3E807	---	---	---	---	---	---	---	---	This probe s	S000028581(11),YOR011W-A(11)
1769834_at	259.96	P	738	P	0.3522	P	YOR025W	S. cerevisiae	YOR025W S	chr15:37821	Member of t	HST3	chr15	YOR025W	---	P53687	---	---	---	0006342 //	0005634 //	0004407 //	---	---	This probe s	S000005551(11),GENSCAN00000007376(11),YOR025W(11)
1770458_at	141.04	P	345.7	P	0.408	P	YOR032C	S. cerevisiae	YOR032C S	chr15:38976	Basic helix-l	HMS1	chr15	YOR032C	---	Q12398	---	---	---	0006350 //	0005634 //	0003677 //	---	---	This probe s	S000005558(11),GENEFINDER00000002124(11),GENSCAN00000007073(11),YOR032C(11)
1772781_at	623.33	P	1340.56	P	0.465	P	YOR056C	S. cerevisiae	YOR056C S	chr15:43024	Essential nu	NOB1	chr15	YOR056C	---	Q08444	---	---	---	0000462 //	0005634 //	0003723 //	---	---	This probe s	S000005582(11),GENEFINDER00000002144(11),GENSCAN00000006741(11),YOR056C(11)
1772165_at	492.3	P	1188.65	P	0.4142	P	YOR073W	S. cerevisiae	YOR073W S	chr15:46477	Component	SGO1	chr15	YOR073W	---	Q08490	---	---	---	0000070 //	0000775 //	---	---	---	This probe s	S000005599(11),GENSCAN00000007393(11),YOR073W(11)
1770448_at	315.25	P	665.09	P	0.474	P	YOR074C	S. cerevisiae	YOR074C S	chr15:46667	Thymidylate	CDC21	chr15	YOR074C	---	P06785	---	---	---	0006231 //	0005634 //	0004799 //	---	---	This probe s	S000005600(11),GENEFINDER00000002208(11),GENSCAN00000006807(11),YOR074C(11)
1777978_at	553.6	P	1273.79	P	0.4346	P	YOR078W	S. cerevisiae	YOR078W S	chr15:47272	Component	BUD21	chr15	YOR078W	---	Q08492	---	---	---	0000447 //	0005634 //	0005515 //	IPR001611	---	This probe s	S000005604(11),GENEFINDER00000002242(11),GENSCAN00000007113(11),YOR078W(11)
1779175_at	583.87	P	1644.32	P	0.3551	P	YOR091W	S. cerevisiae	YOR091W S	chr15:49326	Protein of ur	TMA46	chr15	YOR091W	---	Q12000	---	---	---	---	0005737 //	0003676 //	---	---	This probe s	S000005617(11),GENEFINDER00000002002(11),GENSCAN00000006642(11),YOR091W(11)
1771764_at	756.21	P	2183.97	P	0.3463	P	YOR101W	S. cerevisiae	YOR101W S	chr15:51524	GTPase invo	RAS1	chr15	YOR101W	---	P01119	---	---	---	0001302 //	0005622 //	0000166 //	---	---	This probe s	S000005627(11),GENEFINDER00000002220(11),GENSCAN00000007403(11),YOR101W(11)
1770878_at	247.16	P	503.08	P	0.4913	P	YOR144C	S. cerevisiae	YOR144C S	chr15:60271	Protein regu	ELG1	chr15	YOR144C	---	Q12050	---	---	---	0000723 //	0000781 //	0005515 //	---	---	This probe s	S000005670(11),GENSCAN00000007372(11),YOR144C(11)
1773706_at	1332.51	P	2720.11	P	0.4899	P	YOR145C	S. cerevisiae	YOR145C S	chr15:60534	Essential nu	PNO1	chr15	YOR145C	---	Q99216	---	---	---	0000447 //	0005634 //	0003723 //	---	---	This probe s	S000005671(11),GENSCAN00000007214(11),YOR145C(11)
1776391_at	304.35	P	744.09	P	0.409	P	YOR188W	S. cerevisiae	YOR188W S	chr15:68576	Protein invol	MSB1	chr15	YOR188W	---	P21339	---	---	---	0030010 //	0005739 //	---	---	---	This probe s	S000005714(11),GENSCAN00000006805(11),GENEFINDER00000002226(11),YOR188W(11)
1772485_at	317.78	P	734.38	P	0.4327	P	YOR195W	S. cerevisiae	YOR195W S	chr15:71286	Kinetochore-	SLK19	chr15	YOR195W	---	Q08581	---	---	---	0007049 //	0000775 //	0005515 //	---	---	This probe s	S000005721(11),GENEFINDER00000002134(11),GENSCAN00000007265(11),YOR195W(11)
1773719_at	978.49	P	2330.36	P	0.4199	P	YOR206W	S. cerevisiae	YOR206W S	chr15:72751	Protein that	NOC2	chr15	YOR206W	---	P39744	---	---	---	0000054 //	0005634 //	0005515 //	---	---	This probe s	S000005732(11),GENSCAN00000007313(11),YOR206W(11)
1775330_at	443.6	P	889.93	P	0.4985	P	YOR233W	S. cerevisiae	YOR233W S	chr15:77584	Serine/threo	KIN4	chr15	YOR233W	---	Q01919	---	---	---	0006468 //	0005816 //	0000166 //	IPR001245	---	This probe s	S000005759(11),GENEFINDER00000002032(11),GENSCAN00000006980(11),YOR233W(11)

1779549_at	775.41	P	1730.54	P	0.4481	P	YOR236W	S. cerevisiae	YOR236W S	chr15:78090	Dihydrofolat	DFR1	chr15	YOR236W	---	P07807	---	---	---	0006545 // c	0005829 // c	0004146 // c	---	---	This probe s	S000005762(11),GENSCAN00000007064(11),YOR236W(11)
1778358_at	33.95	P	68.54	P	0.4953	P	YOR237W	S. cerevisiae	YOR237W S	chr15:78199	Protein impli	HES1	chr15	YOR237W	---	P35843	---	---	---	0006694 // s	---	0005498 // s	---	---	This probe s	S000005763(11),GENSCAN00000007287(11),YOR237W(11)
1770589_at	517.6	P	1079.73	P	0.4794	P	YOR252W	S. cerevisiae	YOR252W S	chr15:80377	Protein of ur	TMA16	chr15	YOR252W	---	Q08687	---	---	---	---	0005634 // r	---	---	---	This probe s	S000005778(11),GENSCAN00000007090(11),YOR252W(11)
1770200_at	1181.19	P	3261.56	P	0.3622	P	YOR272W	S. cerevisiae	YOR272W S	chr15:83280	Constituent c	YTM1	chr15	YOR272W	---	Q12024	---	---	---	0006364 // r	0005634 // r	0005515 // r	---	---	This probe s	S000005798(11),GENEFINDER00000002001(11),GENSCAN00000007267(11),YOR272W(11)
1774820_at	178.02	P	685.65	P	0.2596	P	YOR287C	S. cerevisiae	YOR287C S	chr15:85093	---	---	chr15	YOR287C	---	Q12481	---	---	---	0000462 // r	---	0005515 // r	---	---	This probe s	S000005813(11),GENEFINDER00000002261(11),GENSCAN00000007232(11),YOR287C(11)
1778229_at	32.2	A	65.47	P	0.4918	P	YOR293C-A	S. cerevisiae	YOR293C-A	chr15:86799	---	---	chr15	YOR293C-A	---	Q3E7Y7	---	---	---	---	---	---	---	---	This probe s	S000028858(11),YOR293C-A(11)
1779516_at	901.09	P	2011.22	P	0.448	P	YOR294W	S. cerevisiae	YOR294W S	chr15:86833	Essential pro	RRS1	chr15	YOR294W	---	Q08746	---	---	---	0006364 // r	0005634 // r	0005515 // r	---	---	This probe s	S000005820(11),GENEFINDER00000002162(11),GENSCAN00000006654(11),YOR294W(11)
1774592_at	54.09	P	175.1	P	0.3089	P	YOR313C	S. cerevisiae	YOR313C S	chr15:90185	Protein whos	SPS4	chr15	YOR313C	---	P09937	---	---	---	0007126 // r	---	0005515 // r	---	---	This probe s	S000005840(11),GENSCAN00000007292(11),YOR313C(11)
1778739_at	328.11	P	1866.65	P	0.1758	P	YOR315W	S. cerevisiae	YOR315W S	chr15:90475	Nuclear prot	SFG1	chr15	YOR315W	---	Q12507	---	---	---	0006350 // t	0005634 // r	0003704 // s	---	---	This probe s	S000005842(11),GENSCAN00000006632(11),YOR315W(11)
1776091_at	1227.05	P	2518.7	P	0.4872	P	YOR340C	S. cerevisiae	YOR340C S	chr15:95919	RNA polyme	RPA43	chr15	YOR340C	---	P46669	---	---	---	0006350 // t	0005634 // r	0003899 // D	---	---	This probe s	S000005867(11),GENSCAN00000006854(11),YOR340C(11)
1773158_at	386.17	P	1341.75	P	0.2878	P	YOR359W	S. cerevisiae	YOR359W S	chr15:10111	Post-transcr	VTS1	chr15	YOR359W	---	Q08831	---	---	---	0000289 // r	0000932 // c	0000166 // r	---	---	This probe s	S000005886(11),GENEFINDER00000002128(11),GENSCAN00000007130(11),YOR359W(11)
1775835_at	155.21	P	407.48	P	0.3809	P	YPL267W	S. cerevisiae	YPL267W S	chr16:38168	Pseudosubs	ACM1	chr16	YPL267W	---	Q08981	---	---	---	0006350 // t	0005634 // r	0055105 // u	---	---	This probe s	S000006188(11),GENSCAN00000005761(11),YPL267W(11)
1772333_at	663.4	P	1415.5	P	0.4687	P	YPL263C	S. cerevisiae	YPL263C S	chr16:44550	Cytoplasmic	KEL3	chr16	YPL263C	---	Q08979	---	---	---	---	0005737 // c	0005515 // r	---	---	This probe s	S000006184(11),GENEFINDER00000001094(11),GENSCAN00000005520(11),YPL263C(11)
1776763_at	789.15	P	2179.04	P	0.3622	P	YPL256C	S. cerevisiae	YPL256C S	chr16:64976	G1 cyclin inv	CLN2	chr16	YPL256C	---	P20438 /// C	---	---	---	0000321 // r	0000307 // c	0005515 // r	---	---	This probe s	S000006177(11),GENSCAN00000005641(11),YPL256C(11)
1769350_at	159.7	P	491.35	P	0.325	P	YPL253C	S. cerevisiae	YPL253C S	chr16:71062	Protein that	VIK1	chr16	YPL253C	---	Q12045	---	---	---	0007017 // r	0005634 // r	0005515 // r	---	---	This probe s	S000006174(11),GENSCAN00000006110(11),YPL253C(11)
1779402_at	693.64	P	1705.06	P	0.4068	P	YPL227C	S. cerevisiae	YPL227C S	chr16:12016	UDP-glucose	ALG5	chr16	YPL227C	---	P40350	---	---	---	0006487 // r	0005783 // e	0004581 // c	---	S000006148	This probe s	S000006148(11),GENSCAN00000005655(11),YPL227C(11)
1772225_at	796.71	P	1593.51	P	0.5	P	YPL212C	S. cerevisiae	YPL212C S	chr16:15151	tRNA:pseud	PUS1	chr16	YPL212C	---	Q12211	---	---	---	0001522 // r	0005634 // r	0005515 // r	---	---	This probe s	S000006133(11),GENEFINDER00000001044(11),GENSCAN00000005572(11),YPL212C(11)
1773687_at	948.27	P	1920.81	P	0.4937	P	YPL207W	S. cerevisiae	YPL207W S	chr16:15990	Protein regu	TYW1	chr16	YPL207W	---	Q08960	---	---	---	0008033 // t	0005783 // e	0000166 // r	---	S000006128	This probe s	S000006128(11),GENSCAN00000006213(11),YPL207W(11)
1771534_at	173.49	P	485.02	P	0.3577	P	YPL193W	S. cerevisiae	YPL193W S	chr16:18140	Protein invol	RSA1	chr16	YPL193W	---	Q08932	---	---	---	0000027 // r	0005634 // r	0005515 // r	---	---	This probe s	S000006114(11),GENSCAN00000005747(11),YPL193W(11)
1770864_at	75.33	P	195.77	P	0.3848	P	YPL153C	S. cerevisiae	YPL153C S	chr16:26172	Protein kinas	RAD53	chr16	YPL153C	---	P22216	---	---	---	0006139 // r	0005634 // r	0000166 // r	---	---	This probe s	S000006074(11),GENEFINDER00000000977(9),GENSCAN00000006005(11),YPL153C(11)
1775962_at	769.51	P	1745.06	P	0.441	P	YPL146C	S. cerevisiae	YPL146C S	chr16:27616	Nucleolar pro	NOP53	chr16	YPL146C	---	Q12080	---	---	---	0000055 // r	0005634 // r	0019843 // r	---	---	This probe s	S000006067(11),GENEFINDER00000001107(11),GENSCAN00000005515(11),YPL146C(11)
1771561_at	192.19	P	556.6	P	0.3453	P	YPL141C	S. cerevisiae	YPL141C S	chr16:28346	---	---	chr16	YPL141C	---	Q03002	---	---	---	0006468 // r	0005737 // c	0000166 // r	IPR001245 ,	---	This probe s	S000006062(11),GENEFINDER00000001108(11),GENSCAN00000006038(11),YPL141C(11)
1779808_at	648.81	P	1504.18	P	0.4313	P	YPL126W	S. cerevisiae	YPL126W S	chr16:31020	U3 snoRNP	NAN1	chr16	YPL126W	---	Q02931	---	---	---	0000462 // r	0005634 // r	0004252 // s	---	---	This probe s	S000006047(11),GENSCAN00000006268(11),YPL126W(11)
1774670_at	133.38	P	410.33	P	0.3251	P	YPL108W	S. cerevisiae	YPL108W S	chr16:34844	---	---	chr16	YPL108W	---	Q02872	---	---	---	---	0005737 // c	0005515 // r	---	---	This probe s	S000006029(11),GENSCAN00000005932(11),YPL108W(11)
1778063_at	1778.56	P	3764.34	P	0.4725	P	YPL093W	S. cerevisiae	YPL093W S	chr16:37097	Putative GTP	NOG1	chr16	YPL093W	---	Q02892	---	---	---	0000054 // r	0005634 // r	0000166 // r	---	---	This probe s	S000006014(11),GENEFINDER00000000961(11),GENSCAN00000005530(11),YPL093W(11)
1777051_at	1321.14	P	2715.34	P	0.4865	P	YPL086C	S. cerevisiae	YPL086C S	chr16:38476	Subunit of E	ELP3	chr16	YPL086C	---	Q02908	---	---	---	0006350 // t	0005634 // r	0003824 // c	---	---	This probe s	S000006007(11),GENEFINDER00000000902(11),GENSCAN00000005595(11),YPL086C(11)
1778325_at	1259.85	P	4999.15	P	0.252	P	YPL061W	S. cerevisiae	YPL061W S	chr16:43258	Cytosolic ald	ALD6	chr16	YPL061W	---	A9LR77 /// P	---	NP_015264	---	0008152 // r	0005737 // c	0000287 // r	---	---	This probe s	EU255273(11)
1771992_at	1210.41	P	3168.55	P	0.382	P	YPL043W	S. cerevisiae	YPL043W S	chr16:46993	Nucleolar pro	NOP4	chr16	YPL043W	---	P37838	---	---	---	0006364 // r	0005634 // r	0000166 // r	---	---	This probe s	S000005964(11),GENEFINDER00000001009(11),GENSCAN00000005844(11),YPL043W(11)
1773627_at	607.44	P	1225.75	P	0.4956	P	YPL030W	S. cerevisiae	YPL030W S	chr16:49353	tRNA(Ser) U	TRM44	chr16	YPL030W	---	Q02648	---	---	---	0030488 // t	0005737 // c	0008168 // r	---	---	This probe s	S000005951(11),GENSCAN00000006293(11),YPL030W(11)
1774757_at	1361.52	P	2889.74	P	0.4712	P	YPL012W	S. cerevisiae	YPL012W S	chr16:52971	Protein regu	RRP12	chr16	YPL012W	---	Q12754	---	---	---	0000462 // r	0005634 // r	0003723 // R	---	---	This probe s	S000005933(11),GENEFINDER00000001075(11),GENSCAN00000005772(11),YPL012W(11)
1776685_at	169.44	P	397.48	P	0.4263	P	YPR018W	S. cerevisiae	YPR018W S	chr16:59447	Largest subu	RLF2	chr16	YPR018W	---	Q12495	---	---	---	0006334 // r	0000775 // c	0005515 // r	---	---	This probe s	S000006222(11),GENEFINDER00000001119(11),GENSCAN00000005819(11),YPR018W(11)
1777863_at	537.35	P	1075.58	P	0.4996	P	YPR034W	S. cerevisiae	YPR034W S	chr16:63951	Component	ARP7	chr16	YPR034W	---	Q12406	---	---	---	0006325 // e	0005634 // r	0005198 // s	---	---	This probe s	S000006238(11),GENEFINDER00000001083(10),GENSCAN00000006002(11),YPR034W(11)
1773575_at	163.1	P	383	P	0.4258	P	YPR104C	S. cerevisiae	YPR104C S	chr16:73361	Transcriptio	FHL1	chr16	YPR104C	---	P39521	---	---	---	0006350 // t	0000790 // r	0003677 // D	---	---	This probe s	S000006308(11),GENEFINDER00000000958(11),GENSCAN00000005878(11),YPR104C(11)
1777153_at	613.22	P	1787.92	P	0.343	P	YPR112C	S. cerevisiae	YPR112C S	chr16:74925	Essential co	MRD1	chr16	YPR112C	---	Q06106	---	---	---	0000447 // e	0005634 // r	0000166 // r	---	---	This probe s	S000006316(11),GENEFINDER00000000897(11),GENSCAN00000005688(11),YPR112C(11)
1779118_at	36.05	P	90.98	P	0.3962	P	YPR116W	S. cerevisiae	YPR116W S	chr16:75864	---	---	chr16	YPR116W	---	Q06109	---	---	---	---	0005739 // r	---	---	---	This probe s	S000006320(11),GENSCAN00000005584(11),YPR116W(11)
1776774_at	218.86	P	710.36	P	0.3081	P	YPR119W	S. cerevisiae	YPR119W S	chr16:77164	B-type cyclin	CLB2	chr16	YPR119W	---	P24869	---	---	---	0000086 // c	0005634 // r	0005515 // r	---	---	This probe s	S000006323(11),GENSCAN00000006165(11),YPR119W(11)
1778323_at	612.98	P	1787.75	P	0.3429	P	YPR137W	S. cerevisiae	YPR137W S	chr16:80235	Protein invol	RRP9	chr16	YPR137W	---	Q06506	---	---	---	0000154 // r	0005634 // r	0005515 // r	---	---	This probe s	S000006341(11),GENEFINDER00000000979(11),GENSCAN00000005936(11),YPR137W(11)
1771931_at	223.56	P	670.4	P	0.3335	P	YPR141C	S. cerevisiae	YPR141C S	chr16:81572	Minus-end-d	KAR3	chr16	YPR141C	---	P17119	---	NP_015467	---	0000741 // k	0005634 // r	0000166 // r	---	---	This probe s	M31719(11)
1779324_at	1005.19	P	2331.94	P	0.4311	P	YPR143W	S. cerevisiae	YPR143W S	chr16:81831	Nucleolar pro	RRP15	chr16	YPR143W	---	Q06511	---	---	---	0000463 // r	0005634 // r	---	---	---	This probe s	S000006347(11),GENEFINDER00000000969(11),GENSCAN00000005544(11),YPR143W(11)
1774976_at	784.06	P	2040.61	P	0.3842	P	YPR144C	S. cerevisiae	YPR144C S	chr16:81975	Nucleolar pro	NOC4	chr16	YPR144C	---	Q06512	---	---	---	0000447 // e	0005634 // r	0005515 // r	---	---	This probe s	S000006348(11),GENSCAN00000005695(11),YPR144C(11)
1774467_at	262.57	P	595.31	P	0.4411	P	YPR186C	S. cerevisiae	YPR186C S	chr16:90972	Transcriptio	PZF1	chr16	YPR186C	---	P39933	---	---	---	0006350 // t	0005622 // i	0003676 // r	---	---	This probe s	S000006390(11),GENSCAN00000005580(11),YPR186C(11)
1773138_at	730.82	P	1573.05	P	0.4646	P	YPR190C	S. cerevisiae	YPR190C S	chr16:91707	RNA polyme	RPC82	chr16	YPR190C	---	P32349	---	---	---	0006350 // t	0005634 // r	0003677 // D	---	---	This probe s	S000006394(11),GENEFINDER00000001096(11),GENSCAN00000005820(11),YPR190C(11)
1773740_at	10.1	A	20.21	P	0.4998	P	SPAC22G7.	S. pombe Sl	SPAC22G7.	chromosome	sequence or	SPAC22G7.	---	---	2541683	Q09797	---	NP_593052	NM_001018	---	0005634 // r	---	---	---	This probe s	NM_001018450(11)
1771603_s_at	5.7	A	15.54	P	0.3668	P	SPCC794.0	S. pombe Sl	SPCC794.0	chromosome	wtf element	SPCC794.0	---	---	2539070	Q8TF80	---	NP_587750	NM_001022	---	0005783 // e	---	---	SPCC794.0	This probe s	NM_001022744(11)
1775735_x_at	46.39	P	151.72	P	0.3058	P	SPBC1105.	S. pombe Sl	SPBC1105.	chromosome	histone H4 h	h4.3	---	---	2539991	P09322	---	NP_596468	NM_001022	0006333 // c	0000786 // r	0003677 // D	---	---	This probe s	NM_001022387(11)
1773797_s_at	1.12	A	2.61	P	0.4291	P	SPBC1711.0	S. pombe Sl	SPBC1711.0	chromosome	mating-type	matmc	---	---	2539619 ///	P10840	---	NP_595867	NM_001021	0000747 // c	0005634 // r	0003677 // D	---	---	This probe s	SPBC1711.02(11),SPBC23G7.09(11),NM_001021773(11),NM_001021781(11)
1772822_at	3.52	A	7.93	P	0.4439	P	SPAC22A12	S. pombe Sl	SPAC22A12	chromosome	sequence or	SPAC22A12	---	---	2541510	Q13894	---	NP_593232	NM_001018	0007126 // r	0005634 // r	---	---	---	This probe s	NM_001018629(11)
1776051_at	10.52	A	26.93	P	0.3906	P	SPAC6F6.0	S. pombe Sl	SPAC6F6.0	chromosome	VIC sodium	SPAC6F6.0	---	---	2542812	Q14234	---	NP_593894	NM_001019	0006810 // t	0005783 // e	0005216 // i	---	SPAC6F6.0	This probe s	NM_001019324(11)
